# Boston Birth Cohort profile: rationale and study design

**DOI:** 10.1097/PN9.0000000000000011

**Published:** 2022-08-18

**Authors:** Colleen Pearson, Tami Bartell, Guoying Wang, Xiumei Hong, Serena A. Rusk, LingLing Fu, Sandra Cerda, Blandine Bustamante-Helfrich, Wendy Kuohung, Christina Yarrington, William G. Adams, Xiaobin Wang

**Affiliations:** 1 Department of Pediatrics, Boston University School of Medicine and Boston Medical Center, Boston, MA, USA; 2 Patrick M. Magoon Institute for Healthy Communities, Ann & Robert H. Lurie Children’s Hospital of Chicago, Chicago, IL, USA; 3 Center on the Early Life Origins of Disease, Department of Population, Family and Reproductive Health, Johns Hopkins Bloomberg School of Public Health, Baltimore, MD, USA; 4 Department of Pathology, Boston University School of Medicine and Boston Medical Center, Boston, MA, USA; 5 University of the Incarnate Word School of Osteopathic Medicine, San Antonio, TX, USA; 6 Department of Obstetrics and Gynecology, Boston University School of Medicine and Boston Medical Center, Boston, MA, USA; 7 Department of Pediatrics, Johns Hopkins School of Medicine, Baltimore, MD, USA.

**Keywords:** Boston Birth Cohort, Longitudinal study, Genetics, Environment, Early life origins of chronic diseases

## Abstract

In1998, the Boston Birth Cohort (BBC) was initiated at Boston Medical Center (BMC) in response to persistently high rates of preterm birth (PTB, defined as birth before 37 weeks of gestation) in the US population and the longstanding profound PTB disparity among Black, Indigenous, and people of color (BIPOC). The BBC encompasses two linked study protocols: The PTB Study serves as the baseline recruitment in the BBC. It aims to address fundamental questions about the causes and consequences of PTB. The study oversamples preterm babies using a case/control study design, in which cases are defined as mothers who deliver a preterm and/or low birthweight baby (<2500 grams regardless of gestational age). Controls are enrolled at a 2:1 control/case ratio and matched by maternal age (±5 years), self-reported race and ethnicity, and date of delivery (± 7 days for case delivery). From inception, it was designed as a comprehensive gene-environmental study of PTB. As a natural extension, the Children’s Health Study, under a separate but linked Institutional Review Board protocol, is a longitudinal follow-up study of the participants who were recruited at birth in the PTB Study and who continue pediatric care at BMC. This linked model allows for investigation of early life origins of pediatric and chronic disease in a prospective cohort design. The BBC is one of the largest and longest National Institutes of Health–funded prospective birth cohort studies in the United States, consisting of 8733 mother-child dyads enrolled in the PTB Study at birth, and of those, 3592 children have been enrolled in the Children’s Health Study, with a median follow-up of 14.5 years. The BBC mirrors the urban, underresourced, and underrepresented BIPOC population served by BMC. A high proportion of BBC children were born prematurely and had chronic health conditions (e.g., asthma, obesity, and elevated blood pressure) in childhood. The BBC’s long-term goal has been to build a large, comprehensive database (epidemiological, clinical, and multiomics) and biospecimen repository to elucidate early life origins of pediatric and chronic diseases and identify modifiable upstream factors (e.g., psychosocial, environmental, and nutritional) to improve health across the life course for BIPOC mothers and children.

## Introduction

This article focuses on the rationale, study design, and scope of data collection of the Boston Birth Cohort (BBC). In a subsequent article, we will highlight major scientific discoveries in the BBC as the result of the past 20+ years of research efforts across multiple organs/systems and scientific disciplines.

### Why was the BBC cohort established?

The BBC was initiated in 1998 at Boston Medical Center (BMC) in response to persistently high rates of preterm birth (PTB; defined as birth before 37 weeks of gestation) in the overall US population and the longstanding profound PTB disparity among Black, Indigenous, and people of color (BIPOC).

PTB is a major cause of neonatal and infant morbidity and mortality with lifetime health consequences for children both in the US and worldwide. In 1998, when the BBC was initiated, the rate of PTB in the US rose to 11.6%, following a rise from 11.0% to 11.4% from 1996 to 1997.^[1]^ In each year since, nearly a half-million babies have been born preterm.^[2]^ Despite intensive research and intervention efforts, the PTB rate has remained high and, at present, affects one in 10 babies in the US and one in seven BIPOC women.^[3]^ Worldwide, 11% of infants are born preterm, and 1 million infants die annually due to PTB.^[1,4]^

PTB represents a longstanding and profound health disparity in the US. In 1998 and still today, 20 years later, the rate of PTB among BIPOC women (14%) has been about 50% higher than the rate among White women (9%).^[5]^ In 2021, for the first time in 6 years, the US PTB rate declined slightly from 10.2% in 2019 to 10.1% in 2020, except among BIPOC mothers whose rate increased from 14.25% to 14.36%.^[6]^

One major obstacle in preventing PTB is our incomplete understanding of its causes and underlying mechanisms. This knowledge gap has been partly explained by the fact that PTB is a complex trait determined by multiple environmental and genetic factors,^[7]^ and because of the significant heterogeneity and complexity of biological pathways that could lead to PTB.^[8,9]^

Early studies on PTB were largely focused on identifying sociodemographic, environmental, and clinical risk factors, of which a number have been recognized, including maternal race and ethnicity,^[10–12]^ age,^[13–15]^ education,^[16,17]^ income,^[18,19]^ nativity,^[20]^ malnutrition,^[21,22]^ stress,^[23–25]^ social support,^[26,27]^ smoking,^[28–30]^ and air pollution.^[31–34]^ A history of prior PTB^[35,36]^ and a woman having been born prematurely herself appear to be the strongest risk factors identified to date.^[37]^ An early study showed that the combination of these two factors significantly increased the risk of PTB or low birthweight (LBW).^[38]^ However, these risk factors still only explain a small fraction of PTB in the overall population and do not appear to drive the PTB disparity among BIPOC women.

While the importance of genetic susceptibility in PTB has been recognized and multiple genetic variants have been linked to risk of PTB, neither candidate genes nor genomewide association studies (GWAS) have been able to pinpoint genetic loci that could help to explain a substantial portion of PTB.^[39–41]^ However, lack of representation of BIPOC in genetic studies can lead to misclassification of benign *vs.* pathologic genetic variants and create or exacerbate health disparities in historically underserved populations.^[42]^ From a health equity perspective, even though BIPOC women have the highest rates of PTB, the majority of US birth cohorts that have included GWAS have primarily been conducted in White European populations.^[43–45]^ Meanwhile, the realization that traditional genetic factors cannot explain the development of heterogeneous, multifactorial health outcomes like PTB has shifted interest toward gene-environment interactions (G×E). Despite their promise, there is a lack of adequately powered studies on the role of G×E interactions in PTB among all populations. Studies conducted in the BBC have provided strong evidence to support this direction.^[12,38,46–50]^

A more profound realization is that the lack of birth cohort studies conducted among historically underrepresented and understudied BIPOC may largely explain our limited understanding of the causes and health disparity of PTB. The BBC’s focus on BIPOC women and children and the interconnections between environmental exposures and genetics are crucial steps toward understanding the etiology of PTB and addressing the persistent disparity of PTB.

### What did the BBC initially aim to investigate?

**Figure 1.**
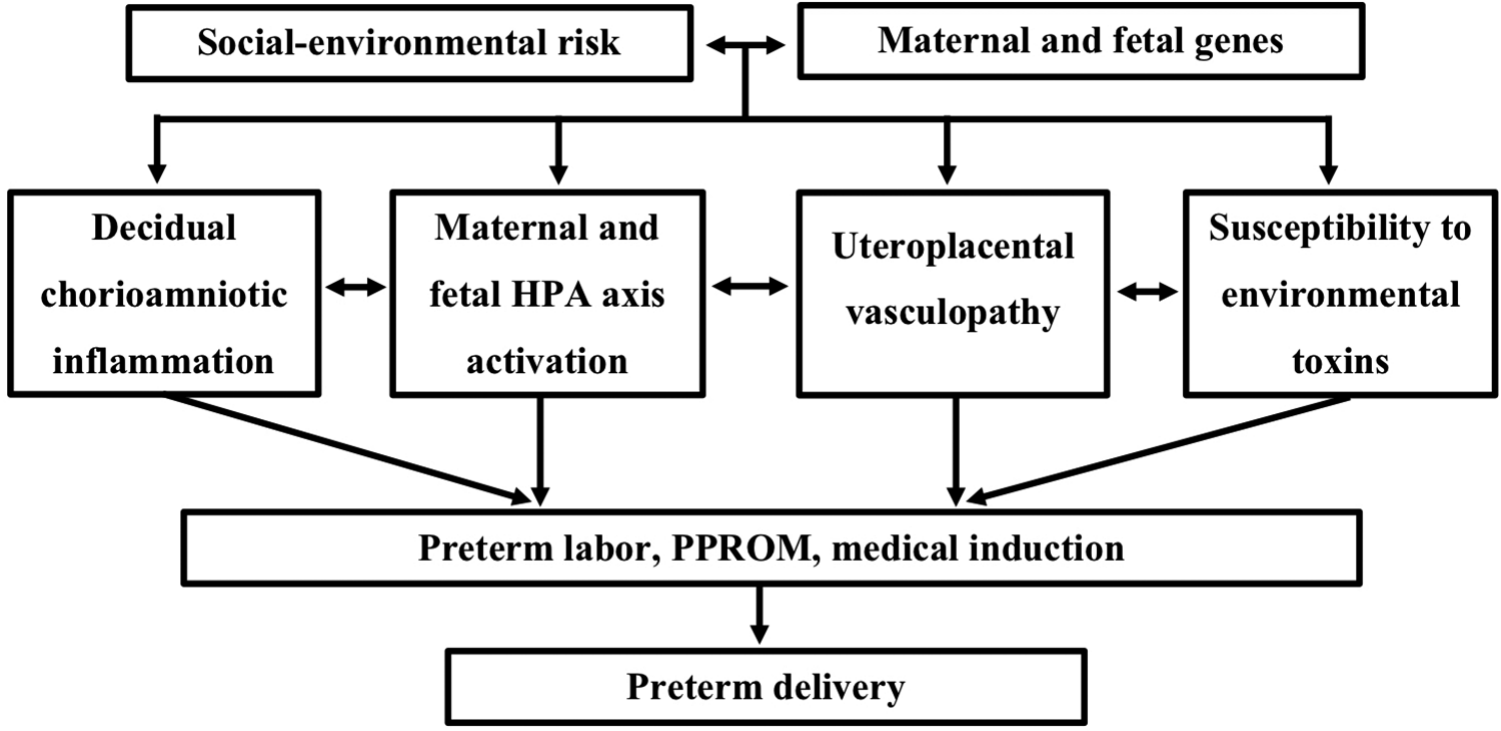
Early conceptual framework for studying preterm birth pathways in the BBC. Adapted from Wang et al ^[48]^. BBC, Boston Birth Cohort; HPA, hypothalamus pituitary adrenal gland; PROM, premature rupture of membranes.

As illustrated in Figure 1, from when it was first conceptualized in 1998, the BBC was designed to address some fundamental questions about the causes of PTB. By considering and collecting data on both genetic and environmental risk factors (including those now also referred to as social determinants of health [SDOH]), the BBC planned and allowed for the analyses of the impact of multilevel, multidimensional biopsychosocial-environmental risk factors and their complex interplay on PTB.

### Health disparities framework underlying research in the BBC

In 2015, the BBC adapted the research framework of the National Institute on Minority Health and Health Disparities ^[51]^ to more thoroughly address the complex and multifaceted nature of minority health and health disparities in BIPOC through the examination of contributions across different domains of influence within a life course perspective (biological, behavioral, nutritional, environmental, and healthcare) and at different levels of influence (individual, family/interpersonal, and community/societal) within those domains. Table 1 highlights the domains examined in the BBC, which have been updated, expanded, and adjusted over time.

**Table 1:** Overview of the scope of data collection in the Boston Birth Cohort.

Domains of influence	Levels of influence		
	Individual	Family/interpersonal	Community/society
Biological	Genomewide genotypes; metabolome, RNA, biomarkers of folate, B12, vitamin D	Maternal and child health	Immunization, prenatal screening (AFP, hCG, and estriol)
Behavioral	Smoking, alcohol, opioids, marijuana, cocaine, stress, and mental health	Marital status, household composition, support of partner, family, and friends; smokers in the household; and mental health and sleep/wake patterns	Witness of violence, rate of crime, smoking, alcohol, drug use, and incarceration
Nutritional	Dietary patterns, beverage consumption, receipt of WIC, and food stamps	Cultural food preference	Grocery stores, farmer’s market access, fast food restaurants, health statistics by zip codes, rates of obesity and diabetes
Environment	Exposure to ambient and indoor air pollution, fuel type, old housing, indoor pests, household pets, carpet, and molds	Family income, country of origin, immigration, ability to speak English, receipt of public assistance, WIC, employment, and access to technology	Disinvested and marginalized neighborhoods, race and ethnicity (self-reported), green space, built environment, public schools, parks, highways, transportation availability, and neighborhood crime rates
Health care	Prenatal and delivery care; lab tests, BMI, BP, diagnoses, medications, and services	Type of insurance, type and frequency of clinical visits, age, parity, and child with special health care needs	Type and completeness of recommended preventive care, and zip code level morbidity and mortality
Sources of data	Questionnaires, medical records, study visits, lab tests, and biosample/biomarkers	Questionnaires, medical records, lab tests, study visits, and biosample/biomarkers	Census data, vital statistics, police crime reports, GPS, and air monitors

WIC, women, infant and children’s supplemental nutrition program; AFP, alpha-fetoprotein; hCG, human chorionic gonadotropin; GPS, global positioning system; RNA, ribonucleic acid; BMI, body mass index; BP blood pressure.

### What are the long-term goals of the BBC?

A long-term goal for the BBC has been to build an adequately powered, comprehensive database, and biospecimen repository that allows for investigation of the causes and long-term health consequences of PTB, and for elucidation of early life origins of pediatric and chronic diseases and the health disparity among BIPOC mother-child dyads enrolled in the BBC. Ultimately, the findings from the BBC will inform clinical and public health practice and policies, including evidence-based primary prevention of PTB and early interventions for children born preterm to mitigate adverse long-term health consequences.

### How was the BBC designed?

**Figure 2.**
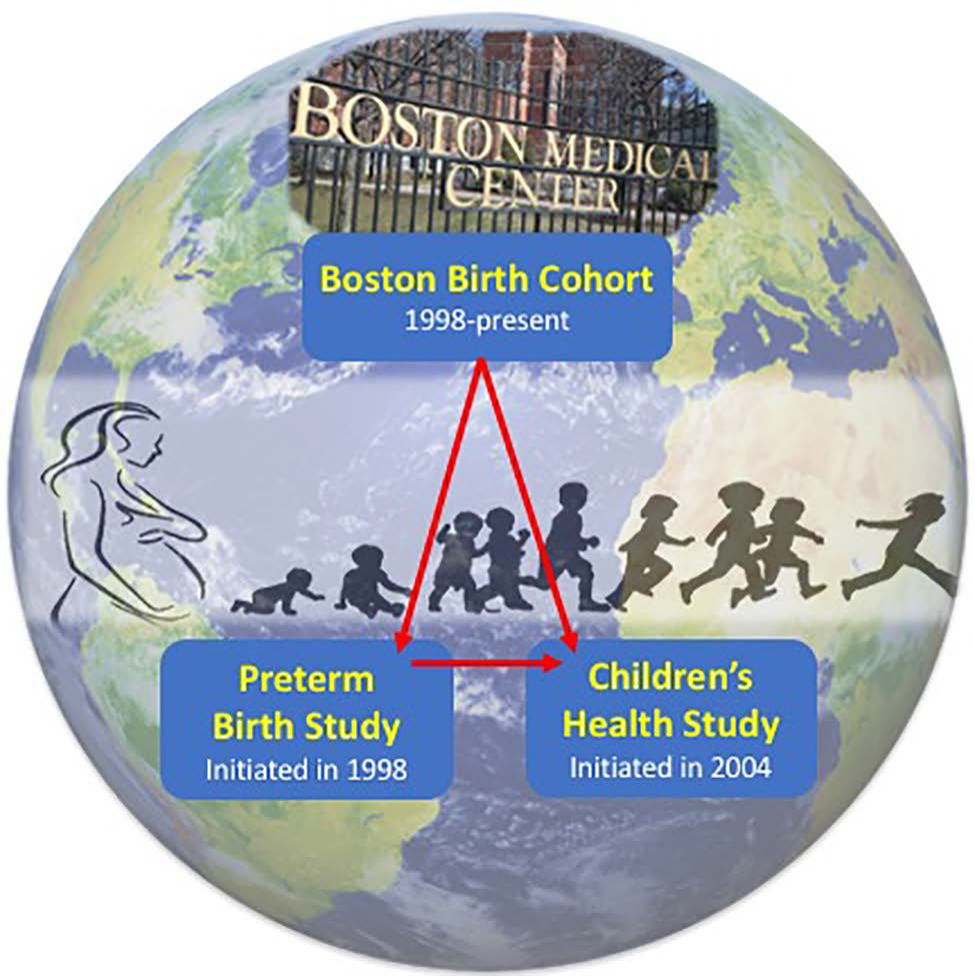
Illustration of the Boston Birth Cohort with two linked IRB-approved protocols at the Boston Medical Center. IRB, Institutional Review Board.

As our understanding of PTB and its impact on life-long health evolved along with the emergence of cutting-edge biomedical advances in science and technology, so did our vision for the cohort. As a result, the BBC encompasses two linked study protocols: the PTB Study and the Children’s Health Study, as illustrated in Figure 2.

The Preterm Birth Study Study serves as the first time point of recruitment and represents baseline recruitment in the BBC. We use a case/control study design, in which cases are defined as mothers who deliver a preterm (<37 weeks gestation) and/or LBW baby (<2500 grams regardless of gestational age). Controls are enrolled at a 2:1 control/case ratio and matched by maternal age (±5 years), self-reported race and ethnicity, and date of delivery (± 7 days for case delivery).

The PTB Study was designed to perform a comprehensive genetic-epidemiologic analysis of PTB and to dissect biological pathways leading to PTB by examining the role of maternal versus fetal genes in each pathway to increase our understanding of genetic and environmental determinants of PTB.^[46,48–50,52–57]^ Over time, we have expanded our research from maternal/fetal genomics to epigenomics^[58–60]^ and metabolomics,^[61,62]^ and have identified deoxyribonucleic acid methylation alterations and metabolites associated with PTB.

### The Children’s Health Study

As a natural evolution of the BBC PTB Study, the Children’s Health Study, initiated under a separate but linked Institutional Review Board (IRB) protocol, aims to conduct a longitudinal follow-up study of the mother-child pairs who were recruited at birth in the PTB Study and who continue pediatric care at BMC. Early studies produced evidence that while PTB manifests at birth, its effects continue across the life course.^[63–66]^ These findings are in line with the theory developed by David Barker that chronic degenerative conditions of adulthood may be triggered by circumstances decades earlier including PTB and LBW.^[67,68]^ That theory, now named the Developmental Origins of Health and Disease ^[69]^ and otherwise known as early life epigenetic programming, points to exposures in utero or during early childhood that program dysfunctions that appear later in life.^[70–73]^ These observations motivated further expansion of the BBC in 2004 to conduct postnatal follow-up of the BBC study population as the Children’s Health Study. This allows for investigation of early life origins of pediatric and chronic disease in a prospective cohort design.^[63–65,74–89]^

### Who are the BBC participants?

All participants in the BBC are recruited at BMC in Boston, MA, the largest safety-net hospital in New England. BMC serves approximately 65% of Boston residents who are concentrated in neighborhoods with the highest level of health disparities. More than half of BMC patients reside in disinvested, economically depleted neighborhoods and receive health care coverage through government payers such as Medicaid, the Health Safety Net, and Medicare.^[90]^

### What are the study procedures of the BBC?

#### Recruitment to the Preterm Birth Study

We identify potential participants for the PTB Study by tracking daily births and admissions to the postpartum floor at BMC. The inclusion and exclusion criteria include the following: any woman admitted to the postpartum floor who delivers a singleton live infant and meets our case (gestational age <37 weeks or lowbirthweight if ≤ 2500 grams and non lowbirthweight is >2500 grams) or control (full-term ≥37 weeks with birthweight ≥2500 grams) definition. A small percentage of women (<1%) with pregnancies that involve in vitro fertilization, multiple gestation, chromosomal abnormalities, or major birth defects; or women who delivered as the result of major trauma are excluded from the study. Women who do not understand English, Spanish, or Haitian Creole, the three primary languages of the BMC patient population for which we have language-specific IRB-approved consent forms, are also excluded.

Multilingual and culturally diverse Research Assistants (RAs) meet with eligible mothers at the maternal bedside during her postpartum hospital admission 24 hours after a vaginal delivery and 48–72 hours after Cesarean section to assess interest in participation. This timing was chosen to allow for maternal recuperation from labor and delivery (L&D). After determining eligibility, the RA explains the study in-depth and obtains written informed consent. Once consent is obtained, the RA administers a questionnaire interview and collects a maternal blood sample. The RA also retrieves cord blood and placenta that were obtained by the L&D nurses at delivery and stored in a designated refrigerator in L&D for study collection.

#### Recruitment to the Children’s Health Study

Beginning when a PTB Study child is at least 6 months of age, we invite the mother-child pair to expand their study participation to the Children’s Health Study to be longitudinally followed from birth (through linkage) to age 21. We delay recruitment until the infant is at least 6 months old to allow the mothers time to establish pediatric care and adjust to the demands of caring for a newborn. The inclusion/exclusion criteria include the following: past participation in the PTB Study and biological mother. Biological mothers who have lost permanent legal custody of their children are excluded because they are unable to provide consent for their children.

RAs approach mothers at BMC during check-in to their own or their child’s medical appointments to assess interest in participation and determine eligibility. After an in-depth discussion of the study protocol, written informed consent is obtained from the mother at recruitment and the child at age-determined intervals as required by the BMC IRB for minimal risk studies involving children. Biospecimen collection and protocol measurements also are completed at age-determined intervals. When procedural changes are made, all participants are reconsented. In 2020, the IRB eliminated child assent and replaced it with Parental Permission Consent.

#### How long and how often have BBC participants been followed?

Recruitment into the PTB Study is ongoing. Enrollment in the Children’s Health Study is dynamic; we are both actively recruiting and prospectively following children born over the past 2 decades. Due to the rolling enrollment in the Children’s Health Study, the length of follow-up varies, with a median length of 14.5 years. Many mothers who participated in the PTB Study but did not initially establish pediatric care at BMC in infancy return to BMC for care in childhood and are eligible for recruitment.

Relevant to both the PTB Study and the Children’s Health Study, a growing number of children who were recruited during our earliest enrollment period are now aged 18+ years (i.e., reproductive age range). Beginning in June 2019 through an IRB amendment, we query if a newly recruited mother-infant dyad in the PTB Study is a third-generation BBC participant. These mothers may “opt-in” to allow yearly electronic health record (EHR) surveillance for herself and her newborn until age 18. We also received IRB approval to reconsent the mother and the age 18+ child to continue participation over their lifetime, with an emphasis on yearly health data collection from the EHR. We hope to follow the children into adulthood and the mothers into middle and late adulthood.

As of June 30, 2021, 8733 mother-newborn pairs have been enrolled in the PTB Study, including 4134 (47.34%) BIPOC; 2479 (28.30%) Hispanic; 1065 (12.20%) non-hispanic white; 214 (2.45%) Asian American Pacific Islander; and 841 (9.63%) who did not identify with a race or ethnicity category. Race and ethnicity were self-reported by the mothers during the questionnaire interview. To enhance statistical power to study PTB, the BBC oversampled infants who were born preterm (3106 [35.57%] of the overall BBC). Of the 8733 mother-infant dyads, 3592 children from the PTB Study have been enrolled into the Children’s Health Study. Key characteristics of the mother-infant dyads recruited at birth and subsequently recruited into the Children’s Health Study are presented in Table 2.

**Table 2:** Characteristics of mother-child dyads in the Boston Birth Cohort by the two linked study protocols: Preterm Birth Study and Children’s Health Study.

	Preterm Birth Study	Children’s Health Study
N*	8623 mother/infant dyads	3394 children
Maternal characteristics at delivery		
Maternal age, years	28 (23–33)	28 (23–33)
Null parity, %	43	43
Asian American Pacific Islander,%*	2.2	1.7
African American and non-Hispanic Black, %*	32.5	39
Cabo Verdean, %*	4.3	4.9
Haitian, %*	14.9	19.8
Hispanic, %*	28.3	22
Multiethnic, %*	0.6	0.3
Non-hispanic White %*	11.9	7.4
Did not identify race or ethnicity, %*	5.3	4.9
Education (college or above), %	36	36
Married, %	35	33
US born, %	38	39
High stress during pregnancy, %	19	19
Smoking during pregnancy, %	19	18
Alcohol consumption, %	9	8
Prepregnancy overweight or obesity, %	48	52
Pre- or gestational diabetes, %	11	12
Cesarean section, %	33	36
Age at menarche, years	13 (12–14)	13 (12–14)
Child characteristics		
Child age, years	–	14 (12–18)
Boy, %	50	50
Preterm birth (<37 weeks), %	27	29
Low birthweight (<2500 g), %	26	28

Data are presented as % or median (interquartile range).

*N represents the complete cleaned dataset used for current analysis and not total accrual.

*Self-reported maternal race and ethnicity were defined as a socially constructed identity, not a biological or genetic category.

The BBC has received initial and annual continuation review and approval by the IRBs of BMC and Johns Hopkins University. Because we ask several sensitive questions during the maternal interview, in addition to standard Health Insurance Portability and Accountability Act

 protections, we obtained additional protections of confidentiality. Initially, we obtained a Certificate of Protection under Massachusetts General Law, C11, Section 24A: Reduction of Morbidity and Mortality within the Commonwealth; this protection has since been replaced with a Certificate of Confidentiality granted by the National Institutes of Health CC-HO-11-15. RAs emphasize these added protections to assure mothers of the high level of confidentiality protections to minimize concerns about disclosing sensitive information for research purposes.

### What variables have been measured in the BBC?

#### Data collection in the BBC

The BBC has a rich data repository that is unprecedented in studies conducted with participants who have been historically underrepresented in research.

Our data contain key phenotypes, genetic, microbial, and biological samples collected at multiple time points from birth through early adulthood. Current data collection in the Children’s Health Study focuses on the children’s transition from mid-childhood to adolescence and young adulthood and on the mothers’ transition to middle age. A summary of the data collection scope and timing intervals is shown below in Table 3 for the PTB Study and in Table 4 for the Children’s Health Study. A complete list of questionnaires, medical record abstractions, and variable measurements is available in the Supplemental Data, http://links.lww.com/PN9/A7.

**Table 3:** Data collection and measurements in the Preterm Birth Study on mother and newborn.

Biospecimens	Measurements
Maternal venous blood	Plasma leptin, adiponectin, plasma folate and B12, plasma metabolome, Genome-wide DNA methylation, Genome-wide genotype, RBC metal levels (Pb, Hg, Cd, Mn, Se), archived DNA, RNA, and plasma samples
Cord blood	Plasma leptin, adiponectin, plasma folate and B12, inflammatory markers, plasma metabolome, Genome-wide DNA methylation, Genome-wide genotype, plasma vitamin D, archived DNA, RNA, and plasma samples
Placenta maternal surface	Gross findings, placental weight, and histopathologic findings
Maternal oral swabs	Archived swabs for future oral microbiome
Placenta fetal surface and distal section of umbilical cord	Histopathologic findings
Newborn meconium	Archived for future gut microbiome analysis
Electronic health records	Data collection
Mother (1 year prior to pregnancy and throughout pregnancy until discharge from hospital care)	Height, weight, BMI, BP, labs, blood type and Rh, number of prenatal visits, ultrasounds with biometry, prenatal care site, clinical presentation at delivery, presence of pregnancy complications (DM, GDM, HELLP, Preeclampsia, Eclampsia, Abruption, GHTN, CHTN, placenta previa, and incompetent cervix), mode of delivery, induction of labor, gravidity, parity, number SAB’s and TAB’s, infertility and treatment, urine cultures and dips, presence of sexually transmitted disease, fetal fibronectin, 24-hour urine protein levels, amniocentesis, preterm labor and management, chronic health conditions, length of rupture of membranes to delivery, medications, placenta pathology reports, cigarette smoking, alcohol and illicit drug use including toxicology screening, food and drug allergies, address, zip code, and date of birth
Newborn (until discharge from hospital care)	Length, head circumference, date and time of delivery, *sex assigned at birth, labs, blood type and Rh, weight, gestational age by LMP and early US and/or Dubowitz score, New Ballard Score, Apgar scores, head ultrasounds, presence of birth defects, TORCH infections, length of hospitalization, and medical complications (NEC, IVH, and PDA)
**Maternal questionnaire interview**	**Data collection**
In-person interview at bedside during hospitalization	Fagerström Test for nicotine dependence, Perceived Stress Scale (PSS), epidemiology questionnaires; home environment, prenatal care site, number of prenatal visits attended and missed, food frequency questionnaire, maternal and paternal allergies and asthma history, medications and vitamin and herbal supplements, chronic health conditions, reproductive history, previous birth outcomes and pregnancy complications, age of menarche, smoking, alcohol and illicit drug use history and during pregnancy, maternal and paternal height and weight, maternal and maternal parents nativity, years living in the US, physical activity, employment, self-identified race and ethnicity, highest level of education, social support, income, and public assistance

*Sex assigned at birth is defined as a biologic construct.

BMI, body mass index; BP, blood pressure; Cd, cadmium; CHTN, chronic hypertension; DM, diabetes mellitus; DNA, deoxyribonucleic acid; GDM, gestational diabetes mellitus; GHTN, gestational hypertension; HELLP, Hemolysis, Elevated Liver enzymes and Low Platelets; Hg, hemoglobin; IVH, intraventricular hemorrhage; LMP, last menstrual period; Mn, manganese; NEC, necrotizing enterocolitis; Pb, lead; PDA, patent ductus arteriosus; RBC, red blood cell; RNA, ribonucleic acid; SAB, spontaneous abortion; Se, selenium; TAB, therapeutic abortion; TORCH, toxoplasmosis, others (syphilis, hepatitis B), rubella, cytomegalovirus, herpes simplex.

**Table 4:** Data collection, measurements, and timing in the Children’s Health Study*.

Type of data collection	Yearly	1–2 year	3–5 year	6–9 year	10–14 year	15–21 year
Epidemiology questionnaires; home environment	✓					
Geocodes and EPA air monitoring data	✓					
Covid-19 hardship questionnaires (2020)						
Food frequency questionnaire	✓					
Child clinical EHR data abstraction	✓					
	From birth onward					
Mother clinical EHR data abstraction	✓					
THRIVE survey (social determinants of health)	✓					
M-Chat		✓				
SCQ, SRS-2		✓	✓	✓	✓	✓
ADOS-2 (on children who failed the SCQ at 11>)			✓	✓		
Child asthma, allergy questionnaire interview	✓					
Children’s Sleep Habits Questionnaire (modified)	✓					
Child pubertal staging questionnaire, self-administered				✓	✓	✓
Mother waist circumference and body composition	✓					
Child waist circumference and body composition				✓	✓	✓
Child radioallergosorbent test		✓	✓	✓	✓	✓
Child urine			✓	✓	✓	✓
Child IOS and PFTs			✓	✓	✓	✓
Child height, weight, BMI, and BP at each well-child visit	✓					
Child age of menarche, menstrual disorders in girls				✓	✓	✓
Child pubertal progression in boys and girls questionnaire, self-administered				✓	✓	✓
Physician diagnosis of pubertal disorders				✓	✓	✓
Child blood sample collection and archive		✓	✓	✓	✓	✓

*Data from the Children’s Health Study is linked to data from the Preterm Birth Study (Table 3).

ADOS-2, Autism Diagnostic Observation Schedule-second addition; BMI, body mass index; BP, blood pressure; Covid-19, coronavirus disease 2019; EPA, Environmental Protection Agency; IOS, impulse oscillometry; M-Chat, Modified Checklist for Autism in Toddlers; PFTs Pulmonary Function Tests; SCQ, Social Communication Questionnaire; SRS, Social Responsiveness Scale; WC, waist circumference.

##### The Preterm Birth Study.

In-person maternal questionnaires administered 24–72 hours after delivery, collect baseline information on maternal environmental exposures pre- and post-conception and the mother’s previous birth outcomes. Newborn gestational age is calculated based on the last menstrual period or early ultrasound (<20 weeks gestation). Maternal health is assessed through maternal self-report and EHR abstraction before and during pregnancy for the presence of hypertensive disorders (chronic/gestational hypertension, preeclampsia, eclampsia, and hemolysis elevated liver enzymes and low platelets syndrome); diabetes (diabetes mellitus types 1&2 and gestational diabetes); intrauterine infection, genitourinary infection, past reproductive history and outcomes, and use of illicit substances (blood and urine toxicology screening during pregnancy and at delivery on both mother and neonate) including alcohol use (ever, during pregnancy) and smoking (ever and in each trimester of pregnancy). A modified food frequency questionnaire that focuses on consumption of fruits, vegetables, fiber-rich foods, dairy, pasta, breads, cereal, meat, eggs, nuts, seeds, tofu, shellfish, fish, coffee, teas, juices, and soda is administered to assess average daily intake during pregnancy. To assess stress in pregnancy, we administer the Perceived Stress Scale-10, a 10-question classic stress assessment used because of its brevity, reliability, and validity across language groups.^[91]^

 We follow the mother and infant’s postpartum course through EHR review until both are discharged from hospital care. The RA abstracts clinical variables from the EHR.

##### The Children’s Health Study.

The RA administers yearly questionnaires to assess child health with an emphasis on nutrition, asthma and allergies, development, and pubertal timing. We administer three developmental screening questionnaires: the Modified Checklist for Autism in Toddlers is administered beginning when the child is age 16–30 months. The Social Responsiveness Scale (SRS)-2 (Pre-school version) is administered at age 30–47 months followed by the SRS-2 (School Age) at age ≥48 months. The Social Communication Questionnaire (SCQ) is administered at age ≥30 months. The Autism Diagnostic Observation Schedule, Second Addition (ADOS-2), is a semi-structured, standardized assessment instrument that includes a number of play-based activities and social situations and is designed to obtain information in the areas of communication, reciprocal social interactions, and restricted and repetitive behaviors associated with a diagnosis of autism spectrum disorder. The ADOS-2 is completed on children who failed the SCQ with a research score of 11 or greater and is administered and scored by an ADOS-trained Developmental and Behavioral pediatrician. A self-administered Pubertal Development questionnaire is administered at age 6 years and is conducted yearly. We measure the body composition of both mother and child using the Tanita Body Composition Analyzer BF-350 scale (Tanita Corporation of America, Arlington Heights, IL). The RA also measures maternal and child waist circumference. Waist circumference measurements are taken with a soft cloth tape measure in cm just above the hip bone in a horizontal plane around the abdomen at the level of the highest point of the iliac crest. Before reading the tape measure, the RA ensures that the tape is snug but does not compress the skin and is parallel to the floor. The measurement is made at the end of a normal expiration. Measurements are recorded at 2 separate intervals after asking the mother and/or child to take a breath and exhale. Spirometry and impulse oscillometry (IOS) and pulmonary function tests (PFTs) are performed on a subset of children beginning at age 3 and 6 years, respectively. Due to funding limitations, we eliminated PFT and IOS administration in 2015. We also performed Radioallergosorbent (RAST) testing. Diagnostic testing for specific immunoglobulin E (IgE) to eight common food allergens (milk, peanut, egg, walnut, shellfish, fish, soy, and wheat) was completed by Quest Diagnostics, a Clinical Laboratory Improvement Amendments Certified Lab (Chantilly, VA). RAST results with specific IgE >0.35 kU/L were reported to both pediatric primary care physicians and mothers and entered into the EHR. Due to reduced funding, we eliminated RAST testing in 2016. We schedule childhood study blood draws to align with physician-ordered blood draws and piggyback on our study blood collection by providing additional study tubes to the pediatric phlebotomist for research analysis. Children provide a yearly urine specimen when able.

Maternal and child EHRs are reviewed each year to assess clinical variables including weight, height, blood pressure, lab work, sick visits, hospitalizations, diagnostic tests, medications, and International Classification of Disease (ICD) codes. In 2017, BMC incorporated an SDOH screening tool called THRIVE. Responses are entered in the EHR dashboard that automatically assigns ICD-10 codes. We can access responses to questions about homelessness, food and housing insecurity, inability to afford medications, lack of transportation, educational aspirations, utility bill concerns, and caregiving issues, as documented by providers during routine outpatient healthcare visits outside of the study encounters. With the integration of community health care data into Epic’s EpicCare (Care Everywhere), we can access BMC-affiliated community health center healthcare utilization outside of BMC to complete collection of clinical data, thereby reducing any missing outside healthcare utilization from our rolling recruitment strategy.

#### Biospecimen acquisition and processing in the BBC

Trained RAs draw maternal venous blood at the bedside. We collect a maximum of 10 mL ethylenediaminetetraacetic acid maternal and cord blood. L&D nursing staff collect study cord blood at delivery from all newborns through venous umbilical cord milking. For ribonucleic acid (RNA) analysis and stabilization, we use PAXgene Blood RNA tubes and collect an additional 2.5 mL of maternal whole blood and cord blood on a subset of mothers and newborns (incubated for at least 2 hours at room temperature). Maternal venous, cord, and childhood blood is centrifuged (1430 g at 0°C for 13 minutes) and fractionated into plasma, white blood cells, and red blood cells. Fresh placentas are sectioned in 2 × 2 cm on both maternal and fetal sides containing decidua basalis tissue, membranes, and a small piece of the distal umbilical cord, and stored in −20° freezers. For any recruited participant whose placenta is examined by the hospital pathologist for gross and histopathological review, we code the pathological findings into predominantly eight categories, in line with the classification proposed by Redline.^[92]^ Using Fisher Scientific Sterile Transport Swabs, the maternal oral cavity is swabbed in six locations (buccal, tongue dorsum and lateral, hard palate, sublingual, and gingiva). Meconium is collected from infant waste.

In childhood, we collect venous blood (K2EDTA) at five time points: age 9–12 months, 2–3 years, 4–5 years, 6–7 years, and 10–15 years. RAST blood is collected in 6-mL red-top (plain, nonserum separator) tubes. To ensure maximum efficiency of sample use, each fractionated sample is divided into small aliquots and biobanked. Children provide a yearly urine sample of at least 10 mL collected in bisphenol A-free sterile specimen containers as a sensitive and noninvasive source for measurement of a range of environmental pollutants and renal function. All biospecimens are stored in −80° freezers pending analysis.

#### What health conditions were examined in the BBC?

We have examined maternal and child health outcomes along the following organs/systems. This list will expand with ongoing investigations in the BBC.

- Prenatal, perinatal, and birth outcomes
- Allergy, asthma, and upper and lower airway conditions
- Cardiometabolic outcomes
- Neurodevelopmental outcomes
- Pubertal developmental outcomes

A more detailed description of phenotypes and a list of the BBC publications by organs/systems will be summarized in a subsequent publication.

#### Research infrastructure of the BBC

We intentionally chose BMC as a single recruitment site for a multitude of reasons. BMC is the medical home to our participants anchored within a large academic medical research center. This allows for optimization of resources and uniform and comprehensive repeated clinical data collection within a single medical system and EHR platform.

The conduct of clinical studies also requires robust research infrastructure support, which we can leverage through additional clinical resources, specifically the presence of a study office centrally located in the pediatric clinic along with a continuous EHR query system that began in 2002. Our foremost strategy has been to eliminate, as much as possible, the logistical barriers for mothers to participate in the study. We align study visits with regularly scheduled medical visits across multiple clinical outpatient departments, thereby reducing maternal and child burden by eliminating the need to travel to BMC specifically for study visits.

Along with primary care clinics, the multiple specialty care clinics and patient-focused programs at BMC increase the number and frequency of outpatient visits attended overall. This allows for multiple opportunities to engage with participants outside of yearly well-child visits and offers flexibility for the mother and child to complete the protocol across multiple encounters, further reducing their time burden while maximizing data collection. With a single recruitment site and central location for comprehensive pediatric health care services including inpatient, emergency department, primary care, and subspecialty clinics, we can be nimble and pivot our research team to a specific clinic where participants have the greatest number of appointments, thereby focusing our resources to maximize initial recruitment and engage and retain recruited participants.

We have determined that the most important retention strategy is to be flexible and cognizant of maternal time constraints to complete the study procedures in a single session. We include an option for “deferment of procedures” in the informed consent to allow participants to complete questionnaires and measurements over several encounters. The RA can assess in real-time if a specific clinical care visit has been long or if the mother is rushed and defer approaching her for study activities until the next visit. This approach accounts for our considerably small withdrawal rate and highlights the advantages of our integrated study presence in the pediatric clinic.

#### EHR and Clinical Data Warehouse

BMC established an EHR query system in 2002. With IRB approval and in collaboration with BMC Information Technology, we developed a weekly computerized query report using patient appointment scheduling to identify participants eligible for or recruited into the Children’s Health Study. By providing the medical records of recruited PTB Study mothers and children, a query can be run each week to output those participants with a scheduled appointment in the pediatric or other ancillary clinics. The data manager further refines the appointment list against the recruitment log file to provide a weekly recruitment list. Additionally, within the EHR system, we conduct real-time monitoring for canceled appointments and, conversely, for when a participant has “checked in” for their appointment.

Clinical variables are obtained from the Clinical Data Warehouse (CDW), a repository of BMC’s patient EHR data including legacy (data from various data systems that are no longer in active clinical use) and community health center data. We provide the medical record numbers of recruited participants in the PTB Study and Children’s Health Study to the CDW Senior Data Manager with updates on recruitment at 6-month intervals.

#### Engagement with participating mothers, children, and providers

Due to concerns about low literacy among BMC mothers, most questionnaires (except for pubertal self-report unless requested by the mother) are read aloud by the RAs. This reduces time and missing data. Since our transition to the research electronic data capture (REDCap) system, we can simplify the presentation of the questionnaires by using the program’s branching logic and voice recording the questions allowing a playback feature for mothers, if needed.

Our study office is equipped with a PlayStation, a television, children’s movies, handheld electronics, and I-pads. In general, performs child measurements while the second RA completes maternal questionnaires and measurements. The RAs are also familiar to providers and administrative staff and able to assess wait times, allowing some study procedures to begin while a participant is waiting for their medical visit to start.

We also periodically conduct information sessions with clinical providers about the Children’s Health Study’s research goals, as we understand clinical staff are instrumental in their assistance as trusted sources of information about research participation.

A study information board hangs in the pediatric clinic hallway with information about the study, pictures of the Pulmonary Function testing and Bioelectric Impedance Analysis equipment along with photographs of RAs, their names, what languages they speak, and a brief biography. As a result, many study mothers and children come to the study office to ask if they are due for any measurements since they are in the clinic and waiting for or finishing their appointment. This is also helpful information for clinicians who rotate through the pediatric outpatient clinics.

We provide a small gift card incentive after protocol completion in the PTB Study and at each yearly completed study visit in the Children’s Health Study. Taxi vouchers are provided if a participant arrives before their medical appointment to complete study procedures specifically for study purposes.

#### Impact of COVID-19 on the BBC

In March 2020, all in-person non-COVID-19 and safety-related research activities were suspended at BMC. With IRB approval, we administered questionnaires to BBC participants electronically using REDCap or over the telephone, adding two newly developed COVID-19 hardship questionnaires. The first COVID-19 questionnaire was designed to assess the acute impact experienced within the first 6 months of the stay-at-home order issued by the City of Boston on March 10, 2020. This questionnaire assessed COVID-19 positivity rates and testing rates, household composition, missed healthcare appointments, experiences of food insecurity, and significant events (job loss, school closures, death, or hospitalization of family members due to COVID-19 positivity). As COVID-19 pandemic restrictions continued in Boston, we administered a second COVID-19 questionnaire to assess the longer-term impact of the pandemic, especially regarding health care visits, school disruptions, caregiver stress, use of telemedicine, loss of employment, housing and food insecurity, and attitudes toward COVID-19 vaccines. Additionally, through weekly EHR surveillance, we queried the incident risk of COVID-19 positive mothers and children in the BBC and followed their health outcomes and symptomology.^[74]^

In essence, COVID-19 not only changed the way the study team interacted with the cohort but also impacted the cohort’s health outcomes, which glaringly highlighted the consequences of health inequities among populations living in low-resource, marginalized communities who make up a large proportion of the essential workforce. The BBC is well-positioned to examine the intersectionality and joint impact of biological factors, SDOH, and COVID-19 on the long-term physical, social, and emotional health of the mothers and children in the BBC, which, in turn, will inform clinical and public health preparedness and policies to minimize adverse consequences due to current and future pandemics.^[74]^

### What are the strengths and limitations of the BBC?

#### Unique strengths of the BBC

In addition to the common strengths associated with prospective birth cohort design, such as temporal, dose-response relationships and the ability to study comorbidities, control for time-dependent covariables, and conduct longitudinal data analyses, the BBC has the following unique strengths:

- Largest, longitudinal birth cohort study of US urban BIPOC women and their children from low-resource communities who bear a disproportionally high burden of social adversities and disparate health outcomes including PTB, but who have been severely underrepresented in PTB research.
- Participants were and continue to be recruited from a single-site medical center using uniform systems including a long-standing, single hospital EHR across time and essential clinical, laboratory, and computational infrastructures to ensure that clinical data are comprehensive and complete.
- By utilizing a wide range of informatics, a diverse study staff, and a study design that embeds study visits alongside clinical visits, through the two linked studies of the BBC, we have successfully recruited and retained participants from birth to age 21 and are approved to reconsent for lifetime follow-up.
- We have a comprehensive collection and archive of extensive epidemiological and clinical data including SDOH and maternal, placental, newborn, and childhood biospecimens.
- Application of cutting-edge biomedical advances in science and technology in the fields of genetics, epigenetics, and metabolomics while also addressing SDOH among BIPOC women and their children living in urban and low-resourced communities. While the collection of genetic, epigenetic, and metabolomic data is not unique, it is rare for such data to be obtained in an understudied and underrepresented BIPOC population. In doing so, the BBC helps to ensure that all women and their children can equally benefit from scientific and technological advancements.

**Figure 3.**
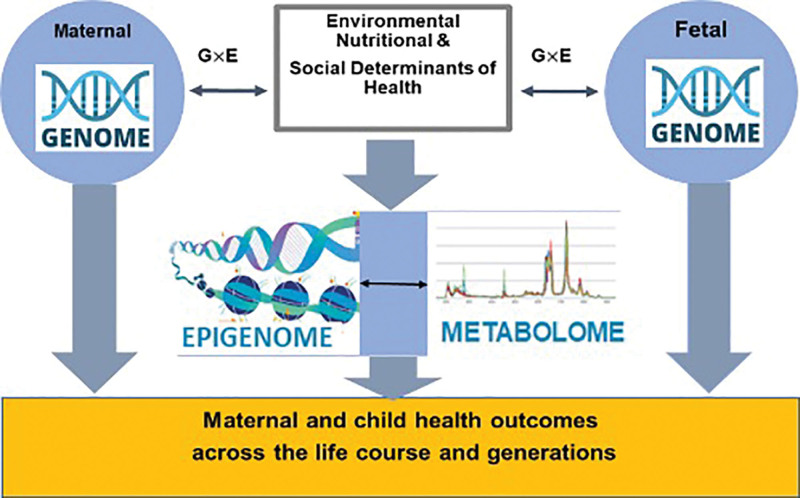
Updated conceptual framework for studying PTB pathways in the BBC. BBC, Boston Birth Cohort; PTB, preterm birth.

Due to exponential advances made in science and technology since 1998, we now use a life-course-biopsychosocial framework,^[93,94]^ which can for the first-time integrate key contributors of PTB including genome, epigenome, metabolome, health inequities, and environmental factors with maternal, placental, and fetal triads in a single study. This positions the BBC to apply current and future biomarkers, identified in the populations most likely to benefit from them, to prevent PTB and improve health outcomes. Figure 3 displays the current investigational model for applying the BBC data collection.

### Limitations, challenges, and opportunities of the BBC

Both a strength and a limitation of the BBC are that it is conducted in a predominately BIPOC population; while findings from the BBC may not be generalizable to other populations, the findings apply to those most impacted by PTB and its long-term consequences. A potential limitation is that, although mothers have preferred having questionnaires read aloud to them, reading questions aloud may have inadvertently produced social desirability bias in their responses.

Furthermore, the unique features of the BBC stated above overcome many of the limitations of most existing birth cohort studies, which struggle with limited sample sizes, fragmented data, longitudinal retention, and lack of multilevel multidimensional data to bridge biology with SDOH. Missing data and attrition/loss to follow-up are still threats to this and any longitudinal studies. There is also the challenge of describing such a dynamic long-term study like the BBC, largely due to the complexity that underlies its seamless operation. This includes significant ongoing IRB policy changes related to retention and tracking that have impacted the cohort in important ways.

The BBC is also uniquely positioned to address the lack of BIPOC representation in biomedical research, and even more so in research that uses advanced biotechnology such as multi- omics. The BBC, with its extensive biorepository, along with extensive and longitudinal collection of epidemiological and clinical data, offers an unprecedented opportunity to gain a deeper understanding of the underlying causes and mechanisms of the persistent disparity in PTB; to provide critically needed evidence to inform public policy, social reform, health services organization and delivery, and clinical and public health programs; and, ultimately, to improve individual and population health for BIPOC and for all society.

## Acknowledgments

We are indebted to the BBC mothers, children, and families for their ongoing participation. We would like to thank over 100 investigators ranging from faculty and postdoctoral fellows to students and research assistants who worked with us over the past 20+ years and brought tremendous energy, diversity, ideas, and talent that was so vital to our recruitment and retention success. We are indebted to the L&D nursing staff for cord blood collection, and the support of the clinical departments in Pediatrics, Family Medicine, and OB/GYN. We appreciate timely EHR data retrieval by Linda Rosen at the BMC CDW. The CDW service is supported by the Boston University Clinical and Translational Institute and the NIH Clinical and Translational Science Award (grant UL1TR001430).

## Ethical Conduct of Research

The Institutional Review Boards (IRBs) of Boston Medical Center and Johns Hopkins Bloomberg School of Public Health approved both study protocols of the BBC. We continually assess RA training, informed consent administration observations, retrain periodically, and perform numerous data quality checks.

## Availability of supporting data

Our goal is to make the BBC data available to investigators, while at the same time respecting the confidentiality of our participants and ensuring the integrity of the data. A subset of the data including maternal and child genotypes and phenotypes from the Preterm Birth Study is available under controlled access request at: Genome-Wide Association Study of Preterm Birth—NCBI (https://www.ncbi.nlm.nih.gov/). Investigators interested in learning more about the BBC and how to obtain BBC data can contact us at BBCcontact@bmc.org. Our team will review specific data requests during monthly team meetings. For general inquiries, we will respond as quickly as possible.

## Clinicaltrials.gov registration

The BBC is registered under clinicaltrials.gov NCT03228875.

## Funding

The Boston Birth Cohort has been supported in part by the March of Dimes PERI grants (20-FY02-56, #21-FY07-605); the Health Resources and Services Administration (HRSA) of the U.S. Department of Health and Human Services (HHS) grants (R40MC27443, UJ2MC31074); and the National Institutes of Health (NIH) grants (R21ES011666, R01HD041702, 2R01HD041702, 2R01HD041702-12, 2R01HD041702-17, R21HD066471, U01AI090727, R21AI079872, R01HD086013, R01HD098232, R21AI154233, R01ES031272, and R01ES031521).

## Author contributions

XW designed and initiated the BBC and has been the principal investigator of the BBC since its inception; CP, GW, XH, SR, LF, SC, BB, WK, CY, WGA, and XW conducted research; and CP, TB, and XW wrote the article. All authors have contributed to and reviewed the article and approved the final article for submission. CP, WGA, and XW had primary responsibility for final content.

## Conflict of interest

None declared.

## Supplementary Material


