# Supplementary material for: Boston Birth Cohort profile: rationale and study design
Source: Precis Nutr. 2022 Aug 18;1(2):e00011. doi: 10.1097/PN9.0000000000000011 (PMC9844822; doi:10.1097/PN9.0000000000000011)
Supplement: Supplementary file 1 [file pn9-1-e00011-s001.pdf]

0301635277

BMC Supplimental Questionnaire

ID [ ][ ][ ][ ][ ][ ][ ][ ][ ][ ][ ] Follow up # [ ][ ]

Date [ ][ ] / [ ][ ] / [ ][ ]

BIA Data

weight [ ][ ][ ].[ ] kg Body Mass Index [ ][ ].[ ] fat percent [ ][ ].[ ] %

Basal Metabolic Rate [ ][ ][ ][ ] kJ impedence [ ][ ][ ] Fat Mass [ ][ ].[ ] kg

Fat Free Mass [ ][ ].[ ] kg Total Body Water [ ][ ].[ ] kg

mother's weight [ ][ ][ ].[ ] kg mother's height [ ][ ][ ].[ ] cm

mother's body mass index [ ][ ].[ ]

Asthma Questions

1. Did a doctor ever tell you that your child had wheezing in the first 3yrs of life?

   ○ Yes ○ No ○ Don't know

   If yes, did it happen more than once?

   ○ Yes ○ No ○ Don't know [ ][ ] # times

2. Has a doctor or nurse EVER told you that your child has asthma?

   ○ Yes ○ No ○ Not Sure

   If **yes**,

3. Who told you? ○ Doctor ○ Nurse

   other [ ][ ][ ][ ][ ][ ][ ][ ][ ][ ][ ][ ][ ][ ][ ]

4. How old was your child at that time? [ ][ ] yrs [ ][ ] mos

Does your child EVER:

5. Wheeze(have whistling in the chest?) ○ Yes ○ No ○ Not Sure

6. Have a cough that will not go away? ○ Yes ○ No ○ Not Sure

7. Cough at night when the child does not have a cold? ○ Yes ○ No ○ Not Sure

8. Have breathing problems when the air temperature changes?

   ○ Yes ○ No ○ Not Sure

5827635275

If child is > 6 yrs.

9. Has your child had wheezing within the last year? ○ Yes ○ No

If yes,

10. Was the wheezing heard by a doctor? ○ Yes ○ No

11. What is your child's current asthma status? ○ Current ○ Outgrown ○ Never

If NEVER end questionnaire

Was this diagnosed by a doctor for the first time since the last visit? ○ Yes ○ No

age of diagnosis ☐☐ *yr* ☐☐ mo

12. In the past 14 days, how many DAYS did your child have any of the following symptoms: wheezing, chest tightness, cough or shortness of breath?

○ Don't Know ☐☐ days

13. In the past 14 days, how many NIGHTS did your child wake up because of any of the following symptoms: wheezing, chest tightness, cough or shortness of breath?

○ Don't Know ☐☐ nights

14. During the past 3 months,when in school, how many DAYS did your child miss school because of wheezing, chest tightness, cough or shortness of breath?

○ Don't Know ☐☐ days

15. During the past 12 months, did your child have to be admitted to the hospital and stay overnight due to asthma?

○ Yes ○ No ○ Don't Know

If yes,

☐☐ # of times

16. During the past 12 months, did your child have a severe asthma episode that required him/her to go to the Emergency Department?

○ Yes ○ No ○ Don't Know

If yes,

☐☐ # of times

17. Not Counting any hospitalizations or emergency visits we just discussed, during the past 12 months, did your child have a severe asthma episode or asthma attack that required him/her to get same day medical care at your doctor's office?

○ Yes ○ No ○ Don't Know

If yes,

☐☐ # of times

18a. Does your child use any inhalers, pumps or puffers?

○ Yes ○ No ○ Don't Know

8159635276

18b. During the past 12 months, have your child used any of these inhalers or pumps?

- ○ Aerobid
- ○ Albuterol
- ○ Alupent
- ○ Atrovent
- ○ Azmacort
- ○ Beclovent
- ○ Combivent
- ○ Flovent
- ○ Foradil
- ○ Intal
- ○ Asmanex
- ○ Xopenex
- ○ Maxair
- ○ Proventil
- ○ Pulmicort
- ○ Qvar
- ○ Serevent (inhaler)
- ○ Tilade
- ○ Vanceril
- ○ Ventolin
- ○ Advair
- ○ Serevent (diskus)

19. Has your child used an inhaler in the past 14 days?

○ Yes ○ No

20a. If YES, please list the inhalers that your child has used.

☐☐☐☐☐☐☐☐☐☐ In the past 14 days, how many days did your child use this inhaler?

○ Don't Know ☐☐ days

☐☐☐☐☐☐☐☐☐☐ In the past 14 days, how many days did your child use this inhaler?

○ Don't Know ☐☐ days

☐☐☐☐☐☐☐☐☐☐ In the past 14 days, how many days did your child use this inhaler?

○ Don't Know ☐☐ days

☐☐☐☐☐☐☐☐☐☐ In the past 14 days, how many days did your child use this inhaler?

○ Don't Know ☐☐ days

☐☐☐☐☐☐☐☐☐☐ In the past 14 days, how many days did your child use this inhaler?

○ Don't Know ☐☐ days

20b. Does your child use other asthma medicines? (daily or $\geq$ 3 days per week)

○ Yes ○ No ○ Don't Know

Singulair ○ Yes ○ No

In the past 14 days, how many days did your child use this medicine? ☐☐ days

Prednisone / Orapred or other oral steroid ○ Yes ○ No

In the past 14 days, how many days did your child use this medicine? ☐☐ days

Other ☐☐☐☐☐☐☐☐☐☐

In the past 14 days, how many days did your child use this medicine? ☐☐ days

21. Does your child have a SPACER such as an Aerochamber to use with his/her inhaler(s) ○ Yes ○ No ○ Don't Know

Baseline

Family ID	______________
Visit ID (Baseline)	______________ (IN0)
Interview Date	______________
Interviewer's Name	______________ (First and last name)
Location of Interview	______________
Child's home zipcode<br><br>¿Qúe es su código poste?	______________

SCREENING: FOR INTERVIEWS
Eligibility

Are you this child's biological mother?<br><br>¿Usted es la madre biológica de..., verdad?	◯ Yes<br>◯ No<br>(IF NO STOP)
Are you this child's legal guardian?<br><br>¿Tiene usted custodia legal de...?	◯ Yes<br>◯ No<br>(IF NO STOP)
Mother's Name Matches Query	◯ Yes<br>◯ No
Child's Name Matches Query	◯ Yes<br>◯ No

IF NO STOP

Section I. Family Pedigree

Can I ask you a few questions about your child's biological father's medical history?<br><br>¿Puedo preguntar sobre el historial médico del padre?	◯ Yes<br>◯ No
Father's Birth Month<br><br>¿Cuál es su fecha de nacimiento?	______________ (Month)

Father's Birth Year<br><br>¿Cuál es su fecha de nacimiento?	________________________<br>(Year)
Father's Medical History<br><br>Usted sabe si el padre de ...tiene algunas enfermedades como<br>Alergias alimentarias<br>Eccema<br>Asma<br>Alergias estacionales<br>Alergias a medicinas<br>Otros<br>Reflujo de ácido	☐ Food Allergy<br>☐ Eczema<br>☐ Asthma<br>☐ Hay Fever<br>☐ Drug Allergy<br>☐ Other Allergies<br>☐ EE<br>☐ GERD
Do you have any other children with her/his father? (Full Sibling)<br><br>¿Tiene ud. otros hijos con el padre de (index kid)?	◯ Yes<br>◯ No
Full Sibling 1. Gender	◯ Male<br>◯ Female
Full Sibling 1 Birth Month<br><br>¿Cuál es la fecha de nacimiento de el/ella?	________________________<br>(Month)
Full Sibling 1 Birth Year<br><br>¿Cuál es la fecha de nacimiento de el/ella?	________________________<br>(Year)
Full Sibling 1 Medical History<br><br>Alergias alimentarias<br>Eccema<br>Asma<br>Alergias estacionales<br>Alergias a medicinas<br>Otros<br>Reflujo de ácido	☐ Food Allergy<br>☐ Eczema<br>☐ Asthma<br>☐ Hay Fever<br>☐ Drug Allergy<br>☐ Other Allergies<br>☐ EE<br>☐ GERD
Full Sibling 2 Gender	◯ Male<br>◯ Female
Full Sibling 2 Birth Month	________________________<br>(Month)
Full Sibling 2 Birth Year	________________________<br>(year)

Full Sibling 2 Medical History	☐ Food Allergy<br>☐ Eczema<br>☐ Asthma<br>☐ Hay Fever<br>☐ Drug Allergy<br>☐ Other Allergies<br>☐ EE<br>☐ GERD
Full Sibling 3 Gender	◯ Male<br>◯ Female
Full Sibling 3 Birth Month	______________________<br>(Month)
Full Sibling 3 Birth Year	______________________<br>(Year)
Full Sibling 3 Medical History	☐ Food Allergy<br>☐ Eczema<br>☐ Asthma<br>☐ Hay Fever<br>☐ Drug Allergy<br>☐ Other Allergies<br>☐ EE<br>☐ GERD
Full Sibling 4 Gender	◯ Male<br>◯ Female
Full Sibling 4 Birth Month	______________________<br>(Month)
Full Sibling 4 Birth Year	______________________<br>(Year)
Full Sibling 4 Medical History	☐ Food Allergy<br>☐ Eczema<br>☐ Asthma<br>☐ Hay Fever<br>☐ Drug Allergy<br>☐ Other Allergies<br>☐ EE<br>☐ GERD
Full Sibling 5 Gender	◯ Male<br>◯ Female
Full Sibling 5 Birth Month	______________________<br>(Month)

Full Sibling 5 Birth Year

____
(Year)

Full Sibling 5 Medical History

- ☐ Food Allergy
- ☐ Eczema
- ☐ Asthma
- ☐ Hay Fever
- ☐ Drug Allergy
- ☐ Other Allergies
- ☐ EE
- ☐ GERD

1. Since birth and/or up until your child reached one year old, has your child ever had any of the following illnesses? (DURING THE FIRST YEAR OF LIFE ONLY)

¿Desde ha nacido o durante el primer año de... tenía algunas enfermedades como:?

	Yes	No	Unsure
Common Cold / Gripe	○	○	○
Ear Infection / Infección de oreja	○	○	○
Pneumonia / Pulmonía	○	○	○
Skin Infection / Infección de piel	○	○	○
Urinary Tract Infection / Infección urinaria	○	○	○
Gastric/intestinal infection / Infección intestinal	○	○	○
Conjunctivitis / Conjuntivitis	○	○	○
Parasite Infection / Infección de parasito	○	○	○
Bone Infection (osteomyelitis) / Infección de hueso	○	○	○
Meningitis / Meningitis	○	○	○
Bacteremia/Sepsis (Blood Infection) / Infección de sangre	○	○	○
RSV/Bronchiolitis / Bronquiolitis	○	○	○
If yes, hospitalized	○	○	○
Sinus Infection / Infección de sino	○	○	○
Bronchitis / Bronquitis	○	○	○
Has your child been diagnosed with any other illnesses within the last year?	○	○	○

Other illness specify

____

Other illness specify

____

If yes, how many times?
Cold ____________________

If yes, how many times?
Ear Infection ____________________

If yes, how many times?
Pneumonia ____________________

If yes, how many times?
Skin Infection ____________________

If yes, how many times?
Urinary Tract Infection ____________________

If yes, how many times?
Gastric/Intestinal Infection ____________________

If yes, how many times?
Conjunctivitis ____________________

2. During the first year of life (or since birth IF THE CHILD IS UNDER 1 YEAR OLD), has your child taken any antibiotics? Antibiotics are medicine that your doctor prescribes for illnesses caused by infections. Examples of some names of commonly prescribed antibiotics are amoxicillin and penicillin?

Desde...ha nacido en el último ano ¿tomo alguno antibiótico?

◯ Yes
◯ No

If yes, how many times was your child prescribed an antibiotic medicine since birth (IF UNDER 1 YEAR OLD) or in the first year of life?

¿Cuántas veces fue ... recetado un antibiótico?

____
(times)

3. During the first year of life (or since birth if the child is under 1 years old), has your child ever lived in a farming environment?

¿Ha vivido en una granja?

◯ Yes
◯ No

4. Were pets present in the home during your child's first year of life (or since birth if child is under 1 year old)?

Desde... ha nacido o en el último año, ha tenido Ud. algunas mascotas o animales en la casa?

◯ Yes
◯ No

	Yes	No
Cats / Gatos	◯	◯
Dogs / Pero	◯	◯

Fish / Pez	◯	◯
Birds / Pajaro	◯	◯
Reptiles / Reptiles	◯	◯
Rabbit / Conejos	◯	◯
Guinea Pig / conejillo de Indias	◯	◯
Others	◯	◯

If others, specify

____

Number of cats present during child's first year?

____

Number of dogs present during child's first year?

____

Number of fish present during child's first year?

____

Number of birds present in child's first year of life?

____

5. How long has your child lived in your current home?
Years

¿Cuántos años ha vivido ... en su casa actual?

____
(Years)

5. How long has your child lived in your current home?
Months

¿Cuántos años ha vivido ... en su casa actual?

____
(Months)

6. Before the age of 5, did someone help in caring for your child for even part of the day? (nanny, daycare, preschool, relative)

Antes de la edad de cinco, ¿Alguien diferente del padres de... cuidaba de...? (Como una niñera guardería, preescolar, otra pariente)

◯ Yes
◯ No

If yes, child's age in years when childcare 1 began
Years

¿Desde qué edad?

____
(Years)

Child's age in months when childcare 1 began
Months

¿Desde qué edad?

____
(months)

Child's age in days when childcare 1 began
Days

¿Desde qué edad?

____
(days)

Child's age in years when childcare 1 ended
Years

¿A qué edad?

______________________ (Years)

Child's age in months that childcare 1 ended

¿A qué edad?

______________________ (Months)

Child's age in days when childcare 1 ended
days

¿A qué edad?

______________________ (days)

# of days/week @ Childcare 1

¿Cuánto días por semana?

______________________ (days/week)

# of other children @ Childcare 1

¿Cuantos otros niños? (en su clase o en el cuidado de niñera/otro pariente)

______________________ (# of other children)

If yes, what age in years when childcare 2 began
Years

______________________ (Years)

Child's age in months when childcare 2 began
Months

______________________ (months)

Child's age in days when childcare 2 began
Days

______________________ (days)

Child's age in years when childcare 2 ended
Years

______________________ (Years)

Child's age in months that childcare 2 ended
Months

______________________ (Months)

Child's age in days when childcare 2 ended
days

______________________ (days)

# of days/week @ Childcare 2

______________________ (days/week)

# of other children @ Childcare 2

______________________ (# of other children)

7. Before your child reached the age of 5, did/do you take care of other children in your home (at least twice a week)?

Antes de la edad de cinco, ¿Ud. cuida de otros niños en su casa?

◯ Yes
◯ No

# of days/week

________________ (# of days/week)

# of other children

________________ (# of other children)

8. Did you breast feed or formula feed your child?

¿Daba el pecho? ¿O alimentaba con formula? ¿Ambos?

◯ Formula Only
◯ Breast Only
◯ Both

9. If breast fed, how long did you exclusively breast feed for (no formula)?
Months

¿Cuánto tiempo le dio pecho exclusivamente? (No formula)

________________ (Months)

9. If breast fed, how long did you exclusively breast feed for (no formula)?
Weeks

¿Cuánto tiempo le dio pecho exclusivamente? (No formula)

________________ (weeks)

9. If breast fed, how long did you exclusively breast feed for (no formula)?
Days

¿Cuánto tiempo le dio pecho exclusivamente? (No formula)

________________ (Days)

10. At what age did you introduce the following formula/milk to your child?

¿A qué edad le dio... formula por la primera vez?

10. At what age did you introduce the following formula/milk to your child?
Cow's milk formula (Enfamil, Similac)?

◯ never
◯ not yet
◯ unsure

Cow's milk formula introduced at:
Years

________________ (Years)

Cow's milk formula introduced at:
Months

________________ (Months)

Cow's milk formula introduced at:
Days

____
(Days)

---

10. At what age did you introduce the following formula/milk to your child?
Whey hydrolyzed formula (Goodstart)?

¿A qué edad le dio... formula por la primera vez?

○ never
○ not yet
○ unsure

Whey hydrolyzed formula introduced at:
Years

____
(Years)

Whey hydrolyzed formula introduced at:
Months

____
(Months)

Whey hydrolyzed formula introduced at:
Days

____
(Days)

---

10. At what age did you introduce the following formula/milk to your child?
Casein Hydrolysate formula?

○ never
○ not yet
○ unsure

Casein Hydrolysate formula introduced at:
Years

____
(Years)

Casein Hydrolysate formula introduced at:
Months

____
(Months)

Casein Hydrolysate formula introduced at:
Days

____
(Days)

---

10. At what age did you introduce the following formula/milk to your child?
Elemental formula (Neocate, Elecare, EO28)?

○ never
○ not yet
○ unsure

Elemental formula introduced at:
Years

____
(Years)

Elemental formula introduced at:
Months

____
(Months)

Elemental formula introduced at:
Days

____
(Days)

---

10. At what age did you introduce the following formula/milk to your child?
Whole cow's milk?

○ never
○ not yet
○ unsure

Whole cow's milk introduced at:
Years

________________ (Years)

Whole cow's milk introduced at:
Months

________________ (Months)

Whole cow's milk introduced at:
Days

________________ (Days)

10. At what age did you introduce the following formula/milk to your child?
Soy formula (Isomil, Prosobee, Alsoy)?

- ◯ never
- ◯ not yet
- ◯ unsure

Soy formula introduced at:
Years

________________ (Years)

Soy formula introduced at:
Months

________________ (Months)

Soy formula introduced at:
Days

________________ (Days)

10. At what age did you introduce the following formula/milk to your child?
Soy milk?

- ◯ never
- ◯ not yet
- ◯ unsure

Soy milk introduced at:
Years

________________ (years)

Soy milk introduced at:
Months

________________ (Months)

Soy milk formula introduced at:
Days

________________ (Days)

11. In a typical week during your pregnancy with this child, on average, how often did you (THE MOTHER) eat the following foods (Only ask those : if cases, ID< 2141, if control ID< 4248)

¿Mientras estaba embrazada de..., con que frecuencia Ud. come estas comidas?

	None	< 1 day	1-2 days	3-5 days	6-7 days	Unsure

Peanut (Including peanut butter) / Maní (o cacahuete)	◯	◯	◯	◯	◯	◯
Tree Nuts (ie almond, cashew, filbert/hazel, walnut, brazil, macadamia, pecan, pine, pistachio) / Otros nueces	◯	◯	◯	◯	◯	◯
Shellfish (ie shrimp, crab, lobster, clam, oyster, mussels) / Mariscos	◯	◯	◯	◯	◯	◯
Wheat (ie pasta, bread, cereal) / Trigo (pan/cereal/pasta)	◯	◯	◯	◯	◯	◯
Soy/Tofu / Soya/Tofu	◯	◯	◯	◯	◯	◯
Seeds (ie sesame, sunflower, pumpkin) / Semillas	◯	◯	◯	◯	◯	◯
Orange veggies (carrots, squash, etc) / Verdura naranjas	◯	◯	◯	◯	◯	◯

12. In a typical week during the period of breast feeding, how often did you (THE MOTHER) eat the following foods?

◯ Not applicable

12. In a typical week during the period of breast feeding, how often did you (THE MOTHER) eat the following foods?

¿Normalmente, mientras estaba amamantado, con qué frecuencia come las siguientes comidas? ¿Cuántas días por semana?

	None	< 1 days	1-2 days	3-5 days	6-7 days	Unsure
Cow's milk/Dairy Products/Cheese / Productos Lácteos	◯	◯	◯	◯	◯	◯
Egg Whites / Huevos	◯	◯	◯	◯	◯	◯
Peanut (including peanut butter) / Maní (incluyendo crema/mantequilla de maní)	◯	◯	◯	◯	◯	◯
Tree Nuts (ie almond, cashew, filbert/hazel, walnut, brazil, macadamia, pecan, pine, pistachio) / Otros nueces	◯	◯	◯	◯	◯	◯
Fish (ie salmon, tuna, catfish, cod, flounder, halibut, trout, bass) / Pescado	◯	◯	◯	◯	◯	◯
Shellfish (shrimp, crab, lobster, clam, oyster, mussels) / Mariscos	◯	◯	◯	◯	◯	◯

Wheat (ie pasta, bread, cereal) / Productos de Trigo	◯	◯	◯	◯	◯	◯
Soy/Tofu / Soja/Tofu	◯	◯	◯	◯	◯	◯
Seeds (ie sesame, sunflower, pumpkin) / Semillas	◯	◯	◯	◯	◯	◯
Green vegetables / Verduras Verdes	◯	◯	◯	◯	◯	◯
Orange veggies (carrots, squash, etc) / Verduras Naranjas	◯	◯	◯	◯	◯	◯
Fruits / Frutas	◯	◯	◯	◯	◯	◯
Meats / Carnes	◯	◯	◯	◯	◯	◯
Beans / Frijoles	◯	◯	◯	◯	◯	◯
Rice / Arroz	◯	◯	◯	◯	◯	◯
Orange Juice / Jugo de naranja	◯	◯	◯	◯	◯	◯

---

13. During breast feeding did you take medications for gastrointestinal upset?

¿Cuándo estaba dando el pecho, tomaba alguna medicina para dolor de estómago?

◯ No
◯ Yes
◯ Unsure
◯ Not Applicable

If YES, which one of the following medications did you take?

☐ Antacids (Mylanta, Rolaids, TUMS, Pepto-Bismol)
☐ H2 Blockers (Pepcid AC, Zantac)
☐ Proton Pump inhibitors (Aciphex, Prilosec, Preveacid, Nexium)
☐ Prokinetic agents (Urecholine, Regland, Erythromycin)
☐ Unsure
☐ Other

If Others, specify:

____
(Other GI medications taken during breast feeding)

---

14. During pregnancy did you take medications for gastrointestinal upset?

¿Tomaba Ud. alguna medicina para el dolor de estómago cuando estaba embarazada...?

◯ No
◯ Yes
◯ Unsure

If YES, which one of the following medications did you take?

☐ Antacids (Mylanta, Rolaids, TUMS, Pepto-Bismol)
☐ H2 Blockers (Pepcid AC, Zantac)
☐ Proton Pump inhibitors (Aciphex, Prilosec, Preveacid, Nexium)
☐ Prokinetic agents (Urecholine, Regland, Erythromycin)
☐ Unsure
☐ Other

If Others, specify:

____

15. In a typical week while you were breast feeding, what brands of skin oil or lotions did you (THE MOTHER) apply to the breast area?

¿Cuándo Ud. daba el pecho a ..., usaba crema o loción en el pecho? ¿Qué tipo o marca?

◯ None
◯ Yes, I remember
◯ Yes, but I don't remember
◯ Unsure
◯ Not Applicable

They are:
Lotion 1

____
(Lotion 1 applied to breast area while breast feeding)

They are:
Lotion 2

____
(Lotion 2 applied to breast area while breast feeding)

They are:
Lotion 3

____
(Lotion 3 applied to breast area while breast feeding)

They are:
Lotion 4

____
(Lotion 4 applied to breast area while breast feeding)

---

16. At what age did you first introduce solid food to your child?

¿Qué edad tenía cuando comió comidas solidas por la primera vez?

◯ Not yet
◯ Never
◯ Unsure

---

16. At what age did you first introduce solid food to your child?
Years

____
(Child's age in years at solid food introduction)

---

16. At what age did you first introduce solid food to your child?
Months

____
(Child's age in months at solid food introduction)

---

17. At what age did you first introduce the following foods to your child?

Ahora, le diré una lista de comidas, y Ud. me dirá que edad tenía... cuando le día estas siguientes comida por la primera vez?

17. At what age did you first introduce the following foods to your child?
Jar Vegetables (baby food)

Verduras para bebes

◯ never
◯ not yet
◯ unsure

---

17. At what age did you first introduce the following foods to your child?
Jar vegetables (baby food)
Years

____
(Child's age in years at introduction of jar vegetables)

17. At what age did you first introduce the following foods to your child?<br>Jar Vegetables (baby food)<br>Months	______________________________<br>(Child's age in months at introduction of jar vegetables)
17. At what age did you first introduce the following foods to your child?<br>Green Vegetables<br><br>Verduras verdes	◯ never<br>◯ not yet<br>◯ unsure
17. At what age did you first introduce the following foods to your child?<br>Green vegetables<br>Years	______________________________<br>(Child's age in years at introduction of green vegetables)
17. At what age did you first introduce the following foods to your child?<br>Green vegetables<br>Months	______________________________<br>(Child's age in months at introduction of green vegetables)
17. At what age did you first introduce the following foods to your child?<br>Orange Vegetables<br><br>Verduras naranjas	◯ never<br>◯ not yet<br>◯ unsure
17. At what age did you first introduce the following foods to your child?<br>Orange vegetables<br>Years	______________________________<br>(Child's age in years at introduction of orange vegetables)
17. At what age did you first introduce the following foods to your child?<br>Orange vegetables<br>Months	______________________________<br>(Child's age in months at introduction of orange vegetables)
17. At what age did you first introduce the following foods to your child?<br>Jar Fruits<br><br>Frutas para bebes	◯ never<br>◯ not yet<br>◯ unsure
17. At what age did you first introduce the following foods to your child?<br>Jar Fruits<br>Years	______________________________<br>(Child's age in years at introduction of jar fruits)
17. At what age did you first introduce the following foods to your child?<br>Jar Fruits<br>Months	______________________________<br>(Child's age in months at introduction of jar fruits)
17. At what age did you first introduce the following foods to your child?<br>Fresh Fruits<br><br>Frutas solida	◯ never<br>◯ not yet<br>◯ unsure

17. At what age did you first introduce the following foods to your child?<br>Fresh Fruits<br>Years	__________________________________<br>(Child's age in years at introduction of fresh fruits)
17. At what age did you first introduce the following foods to your child?<br>Fresh Fruits<br>Months	__________________________________<br>(Child's age in months at introduction of fresh fruits)
17. At what age did you first introduce the following foods to your child?<br>Rice Cereal<br><br>Cereal de arroz	◯ never<br>◯ not yet<br>◯ unsure
17. At what age did you first introduce the following foods to your child?<br>Rice Cereal<br>Years	__________________________________<br>(Child's age in years at introduction of rice cereal)
17. At what age did you first introduce the following foods to your child?<br>Rice Cereal<br>Months	__________________________________<br>(Child's age in months at introduction of rice cereal)
17. At what age did you first introduce the following foods to your child?<br>Cow's Milk/Dairy Products/Cheese<br><br>Productos lácteos	◯ never<br>◯ not yet<br>◯ unsure
17. At what age did you first introduce the following foods to your child?<br>Cow's Milk/Dairy Products/Cheese<br>Years	__________________________________<br>(Child's age in years at introduction of Cow's Milk/Dairy Products/Cheese)
17. At what age did you first introduce the following foods to your child?<br>Cow's Milk/Dairy Products/Cheese<br>Months	__________________________________<br>(Child's age in months at introduction of Cow's Milk/Dairy Products/Cheese)
17. At what age did you first introduce the following foods to your child?<br>Egg<br><br>Huevos	◯ never<br>◯ not yet<br>◯ unsure
17. At what age did you first introduce the following foods to your child?<br>Egg<br>Years	__________________________________<br>(Child's age in years at introduction of egg)
17. At what age did you first introduce the following foods to your child?<br>Egg<br>Months	__________________________________<br>(Child's age in months at introduction of egg)

17. At what age did you first introduce the following foods to your child?
Meat

Carne

◯ never
◯ not yet
◯ unsure

---

17. At what age did you first introduce the following foods to your child?
meat
Years

____
(Child's age in years at introduction of meat)

---

17. At what age did you first introduce the following foods to your child?
Meat
Months

____
(Child's age in months at introduction of meat)

---

17. At what age did you first introduce the following foods to your child?
Fruit Juice

Jugo de fruta

◯ never
◯ not yet
◯ unsure

---

17. At what age did you first introduce the following foods to your child?
Fruit Juice
Years

____
(Child's age in years at introduction of fruit juice)

---

17. At what age did you first introduce the following foods to your child?
Fruit Juice
Months

____
(Child's age in months at introduction of fruit juice)

---

17. At what age did you first introduce the following foods to your child?
Peanut (incl. peanut butter)

Maní (incluyendo mantequilla de maní)

◯ never
◯ not yet
◯ unsure

---

17. At what age did you first introduce the following foods to your child?
Peanut (incl. peanut butter)
Years

____
(Child's age in years at introduction of Peanut (incl. peanut butter))

---

17. At what age did you first introduce the following foods to your child?
Peanut (incl. peanut butter)
Months

____
(Child's age in months at introduction of Peanut (incl. peanut butter))

---

17. At what age did you first introduce the following foods to your child?
Tree Nuts (ie almond, cashew, filbert/hazel, macadamia, pecan, pine, pistachio)

Nueces

◯ never
◯ not yet
◯ unsure

17. At what age did you first introduce the following foods to your child?<br>Tree Nuts (ie almond, cashew, filbert/hazel, macadamia, pecan, pine, pistachio)<br>Years	__________________________________<br>(Child's age in years at introduction of tree nuts)
17. At what age did you first introduce the following foods to your child?<br>Tree Nuts (ie almond, cashew, filbert/hazel, macadamia, pecan, pine, pistachio)<br>Months	__________________________________<br>(Child's age in months at introduction of tree nuts)
17. At what age did you first introduce the following foods to your child?<br>Fish<br><br>Pez	◯ never<br>◯ not yet<br>◯ unsure
17. At what age did you first introduce the following foods to your child?<br>Fish<br>Years	__________________________________<br>(Child's age in years at introduction of fish)
17. At what age did you first introduce the following foods to your child?<br>Fish<br>Months	__________________________________<br>(Child's age in months at introduction of fish)
17. At what age did you first introduce the following foods to your child?<br>Shellfish<br><br>Mariscos	◯ never<br>◯ not yet<br>◯ unsure
17. At what age did you first introduce the following foods to your child?<br>Shell Fish<br>Years	__________________________________<br>(Child's age in years at introduction of shell fish)
17. At what age did you first introduce the following foods to your child?<br>Shellfish<br>Months	__________________________________<br>(Child's age in months at introduction of shellfish)
17. At what age did you first introduce the following foods to your child?<br>Wheat (ie pasta, bread, cereal)<br><br>Trigo	◯ never<br>◯ not yet<br>◯ unsure
17. At what age did you first introduce the following foods to your child?<br>Wheat (ie pasta, bread, cereal)<br>Years	__________________________________<br>(Child's age in years at introduction of wheat)
17. At what age did you first introduce the following foods to your child?<br>Wheat (ie pasta, bread, cereal)<br>Months	__________________________________<br>(Child's age in months at introduction of wheat)

17. At what age did you first introduce the following foods to your child?
Soy/Tofu

Soja/Tofu

◯ never
◯ not yet
◯ unsure

17. At what age did you first introduce the following foods to your child?
Soy/Tofu
Years

____
(Child's age in years at introduction of soy)

17. At what age did you first introduce the following foods to your child?
Soy/Tofu
Months

____
(Child's age in months at introduction of soy)

17. At what age did you first introduce the following foods to your child?
Seeds (ie sesame, sunflower, pumpkin)

Semillas

◯ never
◯ not yet
◯ unsure

17. At what age did you first introduce the following foods to your child?
Seeds (ie sesame, sunflower, pumpkin)
Years

____
(Child's age in years at introduction of seeds)

17. At what age did you first introduce the following foods to your child?
Seeds (ie sesame, sunflower, pumpkin)
Months

____
(Child's age in months at introduction of seeds)

18. During the first year of life or since birth if the child is less than 1 year old, what brands of skin oil or lotion (NOT SOAP) did you use on your child's skin?

Desde...ha nacido hasta su primer año, que tipo de crema o loción usaba Ud. Por su piel su ...?

◯ None
◯ Yes, I remember
◯ Yes, but I don't remember
◯ Unsure

18. They are
Skin Oil/Lotion #1

____

18. They are
Skin Oil/Lotion #2

____

18. They are
Skin Oil/Lotion #3

____

18. They are
Skin Oil/Lotion #4

____

19. At present, does your child take any nutritional supplements or vitamins?

¿Toma... algunas vitaminas o suplementos?

◯ Yes
◯ No

19. If YES, on average how many days per week does your child take a nutritional supplement or vitamin?

¿Cuántas días por semana toma la vitamina?

	None	1-2 days	3-4 days	5-6 days	Everyday	Unsure
Multivitamin/polyvisol / Multivitamínica	◯	◯	◯	◯	◯	◯
Multivitamin with iron (polyvisol with iron) / Multivitamínica con hierro	◯	◯	◯	◯	◯	◯
Trivisol	◯	◯	◯	◯	◯	◯
Calcium / Calcio	◯	◯	◯	◯	◯	◯
Pediasure/Ensure	◯	◯	◯	◯	◯	◯
Other	◯	◯	◯	◯	◯	◯

Other specify: ________________________

20a. At present, how often does your child eat the following foods per week?

Ahora, le diré una lista de comidas, y Ud. me dirá cuántas días por semana...los come?

	None	< 1 day	1-2 days	3-5 days	6-7 days	Unsure
Cow's milk/Dairy Products/Cheese / Productos Lácteos	◯	◯	◯	◯	◯	◯
Egg Whites / Huevos	◯	◯	◯	◯	◯	◯
Peanut (Including peanut butter) / Maní (incluyendo crema/mantequilla de maní)	◯	◯	◯	◯	◯	◯
Tree Nuts (ie almond, cashew, filbert/hazel, walnut, brazil, macadamia, pecan, pine, pistachio) / Otros nueces	◯	◯	◯	◯	◯	◯
Fish (ie salmon, tuna, catfish, cod, flounder, halibut, trout, bass) /Pescado	◯	◯	◯	◯	◯	◯
Shellfish (ie shrimp, crab, lobster, clam, oyster, mussels) / Mariscos	◯	◯	◯	◯	◯	◯
Wheat (ie pasta, bread, cereal) / Productos de Trigo	◯	◯	◯	◯	◯	◯
Soy/Tofu / Soja/Tofu	◯	◯	◯	◯	◯	◯

Seeds (ie sesame, sunflower, pumpkin) / Semillas	○	○	○	○	○	○
Green vegetables /Verduras Verdes	○	○	○	○	○	○
Orange veggies (carrots, squash, etc) / Verduras Naranjas	○	○	○	○	○	○
Fruits / Frutas	○	○	○	○	○	○
Meats / Carnes	○	○	○	○	○	○
Beans / Frijoles	○	○	○	○	○	○
Rice / Arroz	○	○	○	○	○	○
Calcium-fortified Juice / Jugo de naranja con calcio	○	○	○	○	○	○

20b. At present, how often does your child eat breakfast per week?

¿Come...el desayuno todos los días?

- ○ None
- ○ < 1 day
- ○ 1-2 days
- ○ 3-5 days
- ○ 6-7 days
- ○ Unsure

---

21. Does your child have Eczema?

¿Ha tenido eccema?

- ○ No
- ○ Yes, he/she has it now
- ○ Yes, only when she/he was a baby, but outgrew
- ○ Unsure

By what age did your child outgrow his/her Eczema? Years

____
(child's age in YEARS when eczema was outgrown)

By what age did your child outgrow his/her Eczema? Months

____
(child's age in MONTHS when eczema was outgrown)

If YES, was your child's eczema diagnosed by a doctor?

¿Fue diagnosticado por un doctor?

- ○ No
- ○ Yes
- ○ Unsure

How old was your child when first diagnosed by a doctor?

¿Cuándo?

- ○ Yes, I remember
- ○ Unsure

Age IN YEARS when eczema first diagnosed by a doctor

____
(age in years)

Age IN MONTHS when eczema first diagnosed by a doctor

____
(age in months)

22. Have you ever used a steroid cream (like hydrocortisone cream or triamcinolone cream, including creams, lotions, and ointments containing steroids) on your child's skin?<br><br>¿Ha usado...una crema que tiene esteroides (como hidrocortisona) en su piel?	◯ No<br>◯ Yes<br>◯ Unsure
23. Does your child have asthma?<br><br>¿Ha tenido ... asma?	◯ No<br>◯ Yes, he/she has it now<br>◯ Yes, only when she/he was a baby, but outgrew<br>◯ Unsure
By what age did your child outgrow his/her asthma?<br>Years	________________________<br>(child's age in YEARS when asthma was outgrown)
By what age did your child outgrow his/her asthma?<br>Months	________________________<br>(child's age in MONTHS when asthma was outgrown)
If YES, was your child's asthma diagnosed by a doctor?<br><br>¿Fue diagnosticado por un doctor?	◯ No<br>◯ Yes<br>◯ Unsure
How old was your child when first diagnosed by a doctor?<br><br>¿Cuándo?	◯ Yes, I remember<br>◯ Unsure
How old was your child when first diagnosed by a doctor?<br>Years	________________________<br>(child's age in YEARS when asthma was first diagnosed)
How old was your child when first diagnosed by a doctor?<br>Months	________________________<br>(child's age in MONTHS when asthma was first diagnosed)
24. Has your child ever used an inhaler or a nebulizer?<br><br>¿Ha usado.. un inhalador o nebulizador?	◯ Yes<br>◯ No<br>◯ Unsure
25. Does your child have hay fever or seasonal allergies?<br><br>¿Tiene alergias estacionales?	◯ No<br>◯ Yes, he/she has it now<br>◯ Yes, only when she/he was a baby, but outgrew<br>◯ Unsure
By what age did your child outgrow his/her hay fever or seasonal allergies?<br>Years	________________________<br>(child's age in YEARS when hayfever or seasonal allergies was outgrown)

By what age did your child outgrow his/her hay fever or seasonal allergies?<br>Months	__________________________________<br>(child's age in MONTHS when hayfever or seasonal allergies was outgrown)
If YES, was your child's hay fever ever diagnosed by a doctor?<br><br>¿Fue diagnosticado por un doctor?	◯ Yes<br>◯ No<br>◯ Unsure
How old was your child when first diagnosed by a doctor?<br><br>¿Cuándo?	◯ Yes, I remember<br>◯ Unsure
How old was your child when first diagnosed by a doctor?<br>Years	__________________________________<br>(child's age in YEARS when hayfever or seasonal allergies was first diagnosed)
How old was your child when first diagnosed by a doctor?<br>Months	__________________________________<br>(child's age in MONTHS when hayfever or seasonal allergies was first diagnosed)
Which season does your child have seasonal allergies? (select all that apply)<br><br>Primavera<br>Verano<br>Otoño<br>Invierno<br>Todo el año	☐ Spring<br>☐ Summer<br>☐ Autumn<br>☐ Winter<br>☐ Year round<br>☐ Unsure
26. Does your child have pet allergies?<br><br>¿Tiene ... alergias a algunas animales?	◯ No<br>◯ Yes, he/she has it now<br>◯ Yes, only when she/he was a baby, but outgrew<br>◯ Unsure
At what age did your child outgrow his/her pet allergies?<br>Years	__________________________________<br>(child's age in YEARS when pet allergies were outgrown)
At what age did your child outgrow his/her pet allergies?<br>Months	__________________________________<br>(child's age in MONTHS when pet allergies were outgrown)
If YES, what type of pet allergy? (select all that apply)	☐ Cat<br>☐ Dog<br>☐ Other<br>☐ Unsure
If OTHER, specify:	__________________________________<br>(name of other type of pet that child is allergic to)

If YES, was your child's pet allergy diagnosed by a doctor?<br><br>¿Fue diagnosticado por un doctor?	◯ No<br>◯ Yes<br>◯ Unsure
How old was your child when first diagnosed by a doctor?<br><br>¿Cuándo?	◯ Yes, I remember<br>◯ Unsure
How old was your child when first diagnosed by a doctor?<br>Years	______________________<br>(child's age in YEARS when pet allergies were first diagnosed)
How old was your child when first diagnosed by a doctor?<br>Months	______________________<br>(child's age in MONTHS when pet allergies were first diagnosed)
27. Has your child ever used anti-allergy medication? (ie Benadryl, Zyrtec, Claritin, Atarax, Dimetapp)<br><br>¿Ha usado...medicina anti alergia?	◯ Yes<br>◯ No<br>◯ Unsure
28. Does your child have any drug allergies?<br><br>¿Ha tiene alergia a medicina o drogas?	◯ Yes<br>◯ No<br>◯ Unsure
If yes, specify the drug (use "," to separate):	______________________
If YES, was your child's drug allergy diagnosed by a doctor?<br><br>¿Fue diagnosticado por un doctor?	◯ Yes<br>◯ No<br>◯ Unsure
How old was your child when first diagnosed by a doctor?<br><br>¿Cuándo?	◯ Yes, I remember<br>◯ Unsure
How old was your child when first diagnosed by a doctor?<br>Years	______________________<br>(child's age in YEARS when drug allergy was first diagnosed)
How old was your child when first diagnosed by a doctor?<br>Months	______________________<br>(child's age in MONTHS when drug allergy was first diagnosed)
29. Is your child G6PD deficient?<br><br>¿Tiene...una deficiencia de G6PD?	◯ Yes<br>◯ No<br>◯ Unsure

30. Is your child allergic to insect stings?<br><br>¿Ha sido...picado por una abeja o una avispa?<br>¿Tuvo una reacción alérgica?	◯ Yes<br>◯ No<br>◯ Don't know/Child has never been stung
If yes, 1) what type of insect?<br><br>¿Cuál tupo de insecto?	◯ Bee<br>◯ Wasp<br>◯ Yellow Jacket
If yes, 2) Is it a severe allergy (that is, difficulty breathing, need epi pen or to go to the hospital)?<br><br>¿Es una alergia muy grave?	◯ Yes<br>◯ No<br>◯ Unsure
31. Has your child ever used medications for gastrointestinal upset?<br><br>¿Ha usado...alguna medicina por el dolor de estómago?	◯ Yes<br>◯ No<br>◯ Unsure
if YES, which of the following medications did he/she take?	☐ Antacids (Mylants, Rolaids, TUMS, Pepto-Bismol)<br>☐ H2 Blockers<br>☐ Proton Pump inhibitors (Aciphex, Prilosec, Preveacid, Nexium)<br>☐ Prokinetic agents (Urecholine, Reglin, Erythromycin)<br>☐ Unsure<br>☐ Other
If Others, specify:	__________________________________
32. Is your child allergic to any food(s) at present?<br><br>¿Está...actualmente alérgico(a) a algunas comidas?	◯ Yes<br>◯ No
33. Has your child ever been allergic to any foods in the past that they have since outgrown?<br><br>¿Ha tenido...alguna alergia en el pasado?	◯ Yes<br>◯ No
Allergy to Dairy products / Cheese / Milk<br>Current, Outgrown, Never?	◯ Never<br>◯ Current<br>◯ Outgrown
If Current/Outgrown, how old was your child when you first noticed his/her food allergy (to Dairy products/Cheese/Milk)?<br>Years<br><br>¿Cuándo notó por primera vez la alergia?	__________________________________<br>(child's age in years when parent first noticed milk FA)
If Current/Outgrown, how old was your child when you first noticed his/her food allergy (to Dairy products/Cheese/Milk)?<br>Months<br><br>¿Cuándo notó por primera vez la alergia?	__________________________________<br>(child's age in months when parent first noticed milk FA)

If Outgrown, at what age?<br>Years<br><br>¿Cuándo superó la alergia?	__________________________________<br>(child's age in years when he/she outgrew milk FA)
If Outgrown, at what age?<br>Months<br><br>¿Cuándo superó la alergia?	__________________________________<br>(child's age in months when he/she outgrew milk FA)
Allergy to Egg<br>Current, Outgrown, Never?	◯ Never<br>◯ Current<br>◯ Outgrown
If Current/Outgrown, how old was your child when you first noticed his/her food allergy (to Egg)?<br>Years	__________________________________<br>(child's age in years when parent first noticed egg FA)
If Current/Outgrown, how old was your child when you first noticed his/her food allergy (to Egg)?<br>Months	__________________________________<br>(child's age in months when parent first noticed egg FA)
If Outgrown, at what age?<br>Years	__________________________________<br>(child's age in years when he/she outgrew egg FA)
If Outgrown, at what age?<br>Months	__________________________________<br>(child's age in months when he/she outgrew egg FA)
Allergy to Peanuts<br>Current, Outgrown, Never?	◯ Never<br>◯ Current<br>◯ Outgrown
If Current/Outgrown, how old was your child when you first noticed his/her food allergy (to Peanuts)?<br>Years	__________________________________<br>(child's age in years when parent first noticed peanut FA)
If Current/Outgrown, how old was your child when you first noticed his/her food allergy (to Peanuts)?<br>Months	__________________________________<br>(child's age in months when parent first noticed peanut FA)
If Outgrown, at what age?<br>Years	__________________________________<br>(child's age in years when he/she outgrew peanut FA)
If Outgrown, at what age?<br>Months	__________________________________<br>(child's age in months when he/she outgrew peanut FA)

Allergy to Tree Nuts<br>Current, Outgrown, Never?	◯ Never<br>◯ Current<br>◯ Outgrown
If Current/Outgrown, how old was your child when you first noticed his/her food allergy (to Tree Nuts)?<br>Years	______________________<br>(child's age in years when parent first noticed tree nut FA)
If Current/Outgrown, how old was your child when you first noticed his/her food allergy (to Tree Nuts)?<br>Months	______________________<br>(child's age in months when parent first noticed tree nut FA)
If CURRENT, please choose the specific type (select all that apply):	☐ Almond<br>☐ Cashew<br>☐ Filbert/hazel<br>☐ Walnut<br>☐ Brazil<br>☐ Macadamia<br>☐ Pecan<br>☐ Pine<br>☐ Pistachio<br>☐ Other
If OUTGROWN, at what age?<br>Years	______________________<br>(child's age in years when he/she outgrew treenut FA)
If OUTGROWN, at what age?<br>Months	______________________<br>(child's age in months when he/she outgrew treenut FA)
If OUTGROWN, please choose the specific type (select all that apply):	☐ Almond<br>☐ Cashew<br>☐ Filbert/hazel<br>☐ Walnut<br>☐ Brazil<br>☐ Macadamia<br>☐ Pecan<br>☐ Pine<br>☐ Pistachio<br>☐ Other
Allergy to Fish<br>Current, Outgrown, Never?	◯ Never<br>◯ Current<br>◯ Outgrown
If Current/Outgrown, how old was your child when you first noticed his/her food allergy (to Fish)?<br>Years	______________________<br>(child's age in years when parent first noticed fish FA)
If Current/Outgrown, how old was your child when you first noticed his/her food allergy (to Fish)?<br>Months	______________________<br>(child's age in months when parent first noticed fish FA)

If CURRENT, please choose the specific type (select all that apply)	☐ Salmon<br>☐ Tuna<br>☐ Catfish<br>☐ Cod<br>☐ Flounder<br>☐ Halibut<br>☐ Trout<br>☐ Bass
If CURRENT, other type of fish child is allergic to?	__________________________________
If OUTGROWN, at what age?<br>Years	__________________________________<br>(child's age in years when he/she outgrew fish FA)
If OUTGROWN, at what age?<br>Months	__________________________________<br>(child's age in months when he/she outgrew fish FA)
If OUTGROWN, please choose the specific type (select all that apply)	☐ Salmon<br>☐ Tuna<br>☐ Catfish<br>☐ Cod<br>☐ Flounder<br>☐ Halibut<br>☐ Trout<br>☐ Bass
If OUTGROWN, other type of fish that child was allergic to?	__________________________________
Allergy to Shellfish<br>Current, Outgrown, Never?	◯ Never<br>◯ Current<br>◯ Outgrown
If Current/Outgrown, how old was your child when you first noticed his/her food allergy (to Shellfish)?<br>Years	__________________________________<br>(child's age in years when parent first noticed shellfish FA)
If Current/Outgrown, how old was your child when you first noticed his/her food allergy (to Shellfish)?<br>Months	__________________________________<br>(child's age in months when parent first noticed shellfish FA)
If CURRENT, please choose the specific type (select all that apply)	☐ Shrimp<br>☐ Crab<br>☐ Lobster<br>☐ Clam<br>☐ Oyster<br>☐ Mussels
If OUTGROWN, at what age?<br>Years	__________________________________<br>(child's age in years when he/she outgrew shellfish FA)

If OUTGROWN, at what age?<br>Months	__________________________________<br>(child's age in months when he/she outgrew shellfish FA)
If OUTGROWN, please choose the specific type (select all that apply)	☐ Shrimp<br>☐ Crab<br>☐ Lobster<br>☐ Clam<br>☐ Oyster<br>☐ Mussels
Allergy to Wheat<br>Current, Outgrown, Never?	◯ Never<br>◯ Current<br>◯ Outgrown
If Current/Outgrown, how old was your child when you first noticed his/her food allergy (to Wheat)?<br>Years	__________________________________<br>(child's age in years when parent first noticed wheat FA)
If Current/Outgrown, how old was your child when you first noticed his/her food allergy (to Wheat)?<br>Months	__________________________________<br>(child's age in months when parent first noticed wheat FA)
If OUTGROWN, at what age?<br>Years	__________________________________<br>(child's age in years when he/she outgrew wheat FA)
If OUTGROWN, at what age?<br>Months	__________________________________<br>(child's age in months when he/she outgrew wheat FA)
Allergy to Soy/Tofu<br>Current, Outgrown, Never?	◯ Never<br>◯ Current<br>◯ Outgrown
If Current/Outgrown, how old was your child when you first noticed his/her food allergy (to Soy/Tofu)?<br>Years	__________________________________<br>(child's age in years when parent first noticed soy/tofu FA)
If Current/Outgrown, how old was your child when you first noticed his/her food allergy (to Soy/Tofu)?<br>Months	__________________________________<br>(child's age in months when parent first noticed soy/tofu FA)
If OUTGROWN, at what age?<br>Years	__________________________________<br>(child's age in years when he/she outgrew soy FA)
If OUTGROWN, at what age?<br>Months	__________________________________<br>(child's age in months when he/she outgrew soy FA)

Allergy to Seeds<br>Current, Outgrown, Never?	◯ Never<br>◯ Current<br>◯ Outgrown
If Current/Outgrown, how old was your child when you first noticed his/her food allergy (to Seeds)?<br>Years	______________________________<br>(child's age in years when parent first noticed seed FA)
If Current/Outgrown, how old was your child when you first noticed his/her food allergy (to Seeds)?<br>Months	______________________________<br>(child's age in months when parent first noticed seed FA)
If CURRENT, please choose the specific type (select all that apply)	◯ Sesame<br>◯ Sunflower<br>◯ Pumpkin
If CURRENT, name of other type of seed that child is allergic to?	______________________________
If OUTGROWN, at what age?<br>Years	______________________________<br>(child's age in years when he/she outgrew seed FA)
If OUTGROWN, at what age?<br>Months	______________________________<br>(child's age in months when he/she outgrew seed FA)
If OUTGROWN, please choose the specific type (select all that apply):	☐ Sesame<br>☐ Sunflower<br>☐ Pumpkin
If OUTGROWN, other type of seed child is allergic to?	______________________________
Specify Other Food Allergy #1:	______________________________<br>(name of other food #1 child is allergic to)
Other Food Allergy #1<br>Current or Outgrown?	◯ Current<br>◯ Outgrown
Other Food Allergy #1<br>How old was your child when you first noticed his/her food allergy?<br>Years	______________________________<br>(child's age in years when parent first noticed food #1 FA)
Other Food Allergy #1<br>How old was your child when you first noticed his/her food allergy?<br>Months	______________________________<br>(child's age in months when parent first noticed food #1 FA)
Other Food Allergy #1<br>If OUTGROWN, at what age?<br>Years	______________________________<br>(child's age in years when he/she outgrew food #1 FA)

Other Food Allergy #1<br>If OUTGROWN, at what age?<br>Months	__________________________________<br>(child's age in months when he/she outgrew food #1 FA)
Specify Other Food Allergy #2:	__________________________________<br>(name of other food #2 child is allergic to)
Other Food Allergy #2<br>Current or Outgrown?	◯ Current<br>◯ Outgrown
Other Food Allergy #2<br>How old was your child when you first noticed his/her food allergy?<br>Years	__________________________________<br>(child's age in years when parent first noticed food #2 FA)
Other Food Allergy #2<br>How old was your child when you first noticed his/her food allergy?<br>Months	__________________________________<br>(child's age in months when parent first noticed food #2 FA)
If OUTGROWN, at what age?<br>Years	__________________________________<br>(child's age in years when he/she outgrew food #2 FA)
If OUTGROWN, at what age?<br>Months	__________________________________<br>(child's age in months when he/she outgrew food #2 FA)
Specify Other Food Allergy #3:	__________________________________<br>(name of other food #3 child is allergic to)
Other Food Allergy #3<br>Current or Outgrown?	◯ Current<br>◯ Outgrown
Other Food Allergy #3<br>How old was your child when you first noticed his/her food allergy?<br>Years	__________________________________<br>(child's age in years when parent first noticed food #3 FA)
Other Food Allergy #3<br>How old was your child when you first noticed his/her food allergy?<br>Months	__________________________________<br>(child's age in months when parent first noticed food #3 FA)
Other Food Allergy #3<br>If Outgrown, at what age?<br>Years	__________________________________<br>(child's age in years when he/she outgrew food #3 FA)
Other Food Allergy #3<br>If Outgrown, at what age?<br>Months	__________________________________<br>(child's age in months when he/she outgrew food #3 FA)

Specify Other Food Allergy #4:	______________________<br>(name of other food #4 child is allergic to)
Other Food Allergy #4<br>Current or Outgrown?	◯ Current<br>◯ Outgrown
Other Food Allergy #4<br>How old was your child when you first noticed his/her food allergy?<br>Years	______________________<br>(child's age in years when parent first noticed food #4 FA)
Other Food Allergy #4<br>How old was your child when you first noticed his/her food allergy?<br>Months	______________________<br>(child's age in months when parent first noticed food #4 FA)
Other Food Allergy #4<br>If Outgrown, at what age?<br>Years	______________________<br>(child's age in years when he/she outgrew food #4 FA)
Other Food Allergy #4<br>If Outgrown, at what age?<br>Months	______________________<br>(child's age in years when he/she outgrew food #4 FA)
Specify Other Food Allergy #5:	______________________<br>(name of other food #5 child is allergic to)
Other Food Allergy #5<br>Current or Outgrown?	◯ Current<br>◯ Outgrown
Other Food Allergy #5<br>How old was your child when you first noticed his/her food allergy?<br>Years	______________________<br>(child's age in years when parent first noticed food #5 FA)
Other Food Allergy #5<br>How old was your child when you first noticed his/her food allergy?<br>Months	______________________<br>(child's age in months when parent first noticed food #5 FA)
Other Food Allergy #5<br>If Outgrown, at what age?<br>Years	______________________<br>(child's age in years when he/she outgrew food #5 FA)
Other Food Allergy #5<br>If Outgrown, at what age?<br>Months	______________________<br>(child's age in months when he/she outgrew food #5 FA)
Specify Other Food Allergy #6:	______________________<br>(name of other food #6 child is allergic to)

Other Food Allergy #6<br>Current or Outgrown?	◯ Current<br>◯ Outgrown
Other Food Allergy #6<br>How old was your child when you first noticed his/her food allergy?<br>Years	__________________________________<br>(child's age in years when parent first noticed food #6 FA)
Other Food Allergy #6<br>How old was your child when you first noticed his/her food allergy?<br>Months	__________________________________<br>(child's age in months when parent first noticed food #6 FA)
Other Food Allergy #6<br>If Outgrown, at what age?<br>Years	__________________________________<br>(child's age in years when he/she outgrew food #6 FA)
Other Food Allergy #6<br>If Outgrown, at what age?<br>Months	__________________________________<br>(child's age in months when he/she outgrew food #6 FA)

34. Specific symptoms of food allergy (through ingestion):

Sintomas Alergicas

a. MOUTH

Cow's Milk/Dairy Products/Cheese

	Check box if yes
Lips Itching/Tingling / Picazón de labios	☐
Lips Swelling / Labios hinchados	☐
Tongue Itching/Tingling / Picazón la lengua	☐
Tongue Swelling / Lengua hinchada	☐

34. Specific symptoms of food allergy (through ingestion):

a. MOUTH

Egg

	Check box if yes
Lips Itching/Tingling	☐
Lips Swelling	☐
Tongue Itching/Tingling	☐
Tongue Swelling	☐

34. Specific symptoms of food allergy (through ingestion):
a. MOUTH
Peanut

	Check box if yes
Lips Itching/Tinging	☐
Lips Swelling	☐
Tongue Itching/Tingling	☐
Tongue Swelling	☐

34. Specific symptoms of food allergy (through ingestion):
a. MOUTH
Tree Nuts

	Check box if yes
Lips Itching/Tingling	☐
Lips Swelling	☐
Tongue Itching/Tingling	☐
Tongue Swelling	☐

34. Specific symptoms of food allergy (through ingestion):
a. MOUTH
Fish

	Check box if yes
Lips Itching/Tingling	☐
Lips Swelling	☐
Tongue Itching/Tingling	☐
Tongue Swelling	☐

34. Specific symptoms of food allergy (through ingestion):
a. MOUTH
Shellfish

	Check box if yes
Lips Itching/Tingling	☐
Lips Swelling	☐
Tongue Itching/Tingling	☐
Tongue Swelling	☐

34. Specific symptoms of food allergy (through ingestion):
a. MOUTH
Wheat

	Check box if yes
Lips Itching/Tingling	☐
Lips Swelling	☐

Tongue Itching/Tingling	☐
Tongue Swelling	☐

34. Specific symptoms of food allergy (through ingestion):
a. MOUTH
Soy/Tofu

	Check box if yes
Lips Itching/Tingling	☐
Lips Swelling	☐
Tongue Itching/Tingling	☐
Tongue Swelling	☐

34. Specific symptoms of food allergy (through ingestion):
a. MOUTH
Seeds

	Check box if yes
Lips Itching/Tingling	☐
Lips Swelling	☐
Tongue Itching/Tingling	☐
Tongue Swelling	☐

34a. Name of Other Food Allergy #1 ________________________________

34. Specific symptoms of food allergy (through ingestion):
a. MOUTH
Other Food Allergy #1

	Check box if yes
Lips Itching/Tingling	☐
Lips Swelling	☐
Tongue Itching/Tingling	☐
Tongue Swelling	☐

34a. Name of Other Food Allergy #2 ________________________________

34. Specific symptoms of food allergy (through ingestion):
a. MOUTH
Other Food Allergy #2

	Check box if yes
Lips Itching/Tingling	☐
Lips Swelling	☐

Tongue Itching/Tingling ☐
Tongue Swelling ☐

34a. Name of Other Food Allergy #3 ______________________

34. Specific symptoms of food allergy (through ingestion):
a. MOUTH
Other Food Allergy #3

Check box if yes

Lips Itching/Tingling ○
Lips Swelling ○
Tongue Itching/Tingling ○
Tongue Swelling ○

34a. Name of Other Food Allergy #4 ______________________

34. Specific symptoms of food allergy (through ingestion):
a. MOUTH
Other Food Allergy #4

Check box if yes

Lips Itching/Tingling ☐
Lips Swelling ☐
Tongue Itching/Tingling ☐
Tongue Swelling ☐

34a. Name of Other Food Allergy #5 ______________________

34. Specific symptoms of food allergy (through ingestion):
a. MOUTH
Other Food Allergy #5

Check box if yes

Lips Itching/Tingling ☐
Lips Swelling ☐
Tongue Itching/Tingling ☐
Toungue Swelling ☐

34a. Name of Other Food Allergy #6 ______________________

34. Specific symptoms of food allergy (through ingestion):
a. MOUTH
Other Food Allergy #6

	Check box if yes
Lips Itching/Tingling	○
Lips Swelling	○
Tongue Itching/Tingling	○
Tongue Swelling	○

34. Specific symptoms of food allergy (through ingestion):
b/c. EYE/NOSE / Síntomas de ojos/ nariz
Cow's Milk/Dairy Products/Cheese

	Check box if yes
Red/Watery/Itchy Eye / Ojo rojo/picazón	☐
Swollen Eye / Ojo hinchado	☐
Stuffy/Runny Nose / Congestión nasal	☐
Sneezing / Estornudo	☐
Itchy Nose / Picazón en la nariz	☐

34. Specific symptoms of food allergy (through ingestion):
b/c. EYE/NOSE
Egg

	Check box if yes
Red/Watery/Itchy Eye	☐
Swollen Eye	☐
Stuffy/Runny Nose	☐
Sneezing	☐
Itchy Nose	☐

34. Specific symptoms of food allergy (through ingestion):
b/c. EYE/NOSE
Peanut

	Check box if yes
Red/Watery/Itchy Eye	☐
Swollen Eye	☐
Stuffy/Runny Nose	☐
Sneezing	☐
Itchy Nose	☐

34. Specific symptoms of food allergy (through ingestion):
b/c. EYE/NOSE
Tree Nuts

	Check box if yes
Red/Watery/Itchy Eye	☐
Swollen Eye	☐
Stuffy/Runny Nose	☐
Sneezing	☐
Itchy Nose	☐

34. Specific symptoms of food allergy (through ingestion):
b/c. EYE/NOSE
Fish

	Check box if yes
Red/Watery/Itchy Eye	☐
Swollen Eye	☐
Stuffy/Runny Nose	☐
Sneezing	☐
Itchy Nose	☐

34. Specific symptoms of food allergy (through ingestion):
b/c. EYE/NOSE
Shellfish

	Check box if yes
Red/Watery/Itchy Eye	☐
Swollen Eye	☐
Stuffy/Runny Nose	☐
Sneezing	☐
Itchy Nose	☐

34. Specific symptoms of food allergy (through ingestion):
b/c. EYE/NOSE
Wheat

	Check box if yes
Red/Watery/Itchy Eye	☐
Swollen Eye	☐
Stuffy/Runny Nose	☐
Sneezing	☐
Itchy Nose	☐

34. Specific symptoms of food allergy (through ingestion):
b/c. EYE/NOSE
Soy/Tofu

	Check box if yes
Red/Watery/Itchy Eye	☐
Swollen Eye	☐
Stuffy/Runny Nose	☐
Sneezing	☐
Itchy Nose	☐

34. Specific symptoms of food allergy (through ingestion):
b/c. EYE/NOSE
Seeds

	Check box if yes
Red/Watery/Itchy Eye	☐
Swollen Eye	☐
Stuffy/Runny Nose	☐
Sneezing	☐
Itchy Nose	☐

34b/c. Name of Other Food Allergy #1 ______________________

34. Specific symptoms of food allergy (through ingestion):
b/c. EYE/NOSE
Other Food Allergy #1

	Check box if yes
Red/Watery/Itchy Eye	☐
Swollen Eye	☐
Stuffy/Runny Nose	☐
Sneezing	☐
Itchy Nose	☐

34b/c. Name of Other Food Allergy #2 ______________________

34. Specific symptoms of food allergy (through ingestion):
b/c. EYE/NOSE
Other Food Allergy #2

	Check box if yes
Red/Watery/Itchy Eye	☐

Swollen Eye	☐
Stuffy/Runny Nose	☐
Sneezing	☐
Itchy Nose	☐

34b/c. Name of Other Food Allergy #3 __________________________________

34. Specific symptoms of food allergy (through ingestion):
b/c. EYE/NOSE
Other Food Allergy #3

	Check box if yes
Red/Watery/Itchy Eye	◯
Swollen Eye	◯
Stuffy/Runny Nose	◯
Sneezing	◯
Itchy Nose	◯

34b/c. Name of Other Food Allergy #4 __________________________________

34. Specific symptoms of food allergy (through ingestion):
b/c. EYE/NOSE
Other Food Allergy #4

	Check box if yes
Red/Watery/Itchy Eye	☐
Swollen Eye	☐
Stuffy/Runny Nose	☐
Sneezing	☐
Itchy Nose	☐

34b/c. Name of Other Food Allergy #5 __________________________________

34. Specific symptoms of food allergy (through ingestion):
b/c. EYE/NOSE
Other Food Allergy #5

	Check box if yes
Red/Watery/Itchy Eye	◯
Swollen Eye	◯
Stuffy/Runny Nose	◯

Sneezing ◯
Itchy Nose ◯

34b/c. Name of Other Food Allergy #6

____

34. Specific symptoms of food allergy (through ingestion):
b/c. EYE/NOSE
Other Food Allergy #6

Check box if yes

Red/Watery/Itchy Eye ☐
Swollen Eye ☐
Stuffy/Runny Nose ☐
Sneezing ☐
Itchy Nose ☐

34. Specific symptoms of food allergy (through ingestion):
d. THROAT / Síntomas de la garganta
Cow's Milk/Dairy Products/Cheese

Check box if yes

Itching and/or tightness in the throat / Picazón u opresión en la garganta ☐

Hoarseness/change of voice / Voz ronco ☐

Choking/Difficulty Swallowing / Dificultad para deglutir ☐

Throat Clearing / Limpiado de la garganta ☐

34. Specific symptoms of food allergy (through ingestion):
d. THROAT
Egg

Check box if yes

Itching and/or tightness in the throat ◯

Hoarseness/change of voice ◯
Choking/Difficulty Swallowing ◯
Throat Clearing ◯

34. Specific symptoms of food allergy (through ingestion):
d. THROAT
Peanut

	Check box if yes
Itching and/or tightness in the throat	☐
Hoarseness/change of voice	☐
Choking/Difficulty swallowing	☐
Throat Clearing	☐

34. Specific symptoms of food allergy (through ingestion):
d. THROAT
Tree Nuts

	Check box if yes
Itching and/or tightness in the throat	☐
Hoarseness/change of voice	☐
Choking/Difficulty Swallowing	☐
Throat Clearing	☐

34. Specific symptoms of food allergy (through ingestion):
d. THROAT
Fish

	Check box if yes
Itching and/or tightness in the throat	☐
Hoarseness/change of voice	☐
Choking/Difficulty Swallowing	☐
Throat Clearing	☐

34. Specific symptoms of food allergy (through ingestion):
d. THROAT
Shellfish

	Check box if yes
Itching and/or tightness in the throat	☐
Hoarseness/change of voice	☐
Choking/Difficulty Swallowing	☐
Throat Clearing	☐

34. Specific symptoms of food allergy (through ingestion):
d. THROAT
Wheat

	Check box if yes
Itching and/or tightness in the throat	☐
Hoarseness/change of voice	☐
Choking/Difficulty Swallowing	☐
Throat Clearing	☐

34. Specific symptoms of food allergy (through ingestion):
d. THROAT
Soy/Tofu

	Check box if yes
Itching and/or tightness in the throat	☐
Hoarseness/change of voice	☐
Choking/Difficulty Swallowing	☐
Throat Clearing	☐

34. Specific symptoms of food allergy (through ingestion):
d. THROAT
Seeds

	Check box if yes
Itching and/or tightness in the throat	☐
Hoarseness/change of voice	☐
Choking/Difficulty Swallowing	☐
Throat Clearing	☐

34d. Name of Other Food Allergy #1 ______________________________

34. Specific symptoms of food allergy (through ingestion):
d. THROAT
Other Food Allergy #1

	Check box if yes
Itching and/or tightness in the throat	☐
Hoarseness/change of voice	☐
Choking/Difficulty Swallowing	☐
Throat Clearing	☐

34d. Name of Other Food Allergy #2 ______________________________

34. Specific symptoms of food allergy (through ingestion):
d. THROAT
Other Food Allergy #2

	Check box if yes
Itching and/or tightness in the throat	○
Hoarseness/change of voice	○
Choking/Difficulty Swallowing	○
Throat Clearing	○

34d. Name of Other Food Allergy #3 __________________________________

34. Specific symptoms of food allergy (through ingestion):
d. THROAT
Other Food Allergy #3

	Check box if yes
Itching and/or tightness in the throat	○
Hoarseness/change of voice	○
Choking/Difficulty Swallowing	○
Throat Clearing	○

34d. Name of Other Food Allergy #4 __________________________________

34. Specific symptoms of food allergy (through ingestion):
d. THROAT
Other Food Allergy #4

	Check box if yes
Itching and/or tightness in the throat	☐
Hoarseness/change of voice	☐
Choking/Difficulty Swallowing	☐
Throat Clearing	☐

34d. Name of Other Food Allergy #5 __________________________________

34. Specific symptoms of food allergy (through ingestion):
d. THROAT
Other Food Allergy #5

	Check box if yes
Itching and/or tightness in the throat	☐
Hoarseness/change of voice	☐
Choking/Difficulty Swallowing	☐
Throat Clearing	☐

34d. Name of Other Food Allergy #6 ________________________________

34. Specific symptoms of food allergy (through ingestion):
d. THROAT
Other Food Allergy #6

	Check box if yes
Itching and/or tightness in the throat	☐
Hoarseness/change of voice	☐
Choking/Difficulty Swallowing	☐
Throat Clearing	☐

34. Specific symptoms of food allergy (through ingestion):
e. SKIN / Síntomas de piel
Cow's Milk/Dairy Products/Cheese

	Check box if yes
Itching / Picazón	☐
Hives /Urticaria	☐
Swelling of the face and/or extremeties / Hinchazón de la cara o extremidades	☐
Redness of the skin / Piel rojo	☐

34. Specific symptoms of food allergy (through ingestion):
e. SKIN
Egg

	Check box if yes
Itching	☐
Hives	☐
Swelling of the face and/or extremeties	☐

Redness of the skin ☐

34. Specific symptoms of food allergy (through ingestion):
e. SKIN
Peanut

	Check box if yes
Itching	☐
Hives	☐
Swelling of the face and/or extremeties	☐
Redness of the skin	☐

34. Specific symptoms of food allergy (through ingestion):
e. SKIN
Treenut

	Check box if yes
Itching	☐
Hives	☐
Swelling of the face and/or extremeties	☐
Redness of the skin	☐

34. Specific symptoms of food allergy (through ingestion):
e. SKIN
Fish

	Check box if yes
Itching	☐
Hives	☐
Swelling of the face and/or extremeties	☐
Redness of the skin	☐

34. Specific symptoms of food allergy (through ingestion):
e. SKIN
Shellfish

	Check box if yes
Itching	☐
Hives	☐
Swelling of the face and/or extremeties	☐
Redness of the skin	☐

34. Specific symptoms of food allergy (through ingestion):
e. SKIN
Wheat

	Check box if yes
Itching	☐
Hives	☐
Swelling of the face and/or extremeties	☐
Redness of the skin	☐

34. Specific symptoms of food allergy (through ingestion):
e. SKIN
Soy/Tofu

	Check box if yes
Itching	☐
Hives	☐
Swelling of the face and/or extremeties	☐
Redness of the skin	☐

34. Specific symptoms of food allergy (through ingestion):
e. SKIN
Seeds

	Check box if yes
Itching	☐
Hives	☐
Swelling of the face and/or extremeties	☐
Redness of the skin	☐

34e. Name of Other Food Allergy #1 ______________________________

34. Specific symptoms of food allergy (through ingestion):
e. SKIN
Other Food Allergy #1

	Check box if yes
Itching	☐
Hives	☐
Swelling of the face and/or extremeties	☐
Redness of the skin	☐

34e. Name of Other Food Allergy #2 ______________________________

34. Specific symptoms of food allergy (through ingestion):
e. SKIN
Other Food Allergy #2

	Check box if yes
Itching	☐
Hives	☐
Swelling of the face and/or extremeties	☐
Redness of the skin	☐

34e. Name of Other Food Allergy #3 ______________________________

34. Specific symptoms of food allergy (through ingestion):
e. SKIN
Other Food Allergy #3

	Check box if yes
Itching	☐
Hives	☐
Swelling of the face and/or extremities	☐
Redness of the skin	☐

34e. Name of Other Food Allergy #4 ______________________________

34. Specific symptoms of food allergy (through ingestion):
e. SKIN
Other Food Allergy #4

	Check box if yes
Itching	☐
Hives	☐
Swelling of the face and/or extremities	☐
Redness of the skin	☐

34e. Name of Other Food Allergy #5 ______________________________

34. Specific symptoms of food allergy (through ingestion):
e. SKIN
Other Food Allergy #5

	Check box if yes
Itching	☐
Hives	☐
Swelling of the face and/or extremities	☐
Redness of the skin	☐

34e. Name of Other Food Allergy #6 __________________________________

34. Specific symptoms of food allergy (through ingestion):
e. SKIN
Other Food Allergy #6

	Check box if yes
Itching	☐
Hives	☐
Swelling of the face and/or extremities	☐
Redness of the skin	☐

34. Specific symptoms of food allergy (through ingestion):
f. LUNG / Síntomas de Pulmón
Cow's Milk/Dairy Products/Cheese

	Check box if yes
Shortness of breath / Falta de aliento	☐
Repetitive coughing / Tos repetitiva	☐
Wheezing / Aliento ruidoso	☐
Chest Tightness / Opresión en el pecho	☐

34. Specific symptoms of food allergy (through ingestion):
f. LUNG
Egg

	Check box if yes
Shortness of breath	☐
Repetitive coughing	☐
Wheezing	☐

Chest Tightness	☐

34. Specific symptoms of food allergy (through ingestion):
f. LUNG
Peanut

	Check box if yes
Shortness of breath	☐
Repetitive coughing	☐
Wheezing	☐
Chest Tightness	☐

34. Specific symptoms of food allergy (through ingestion):
f. LUNG
Treenut

	Check box if yes
Shortness of breath	☐
Repetitive Coughing	☐
Wheezing	☐
Chest Tightness	☐

34. Specific symptoms of food allergy (through ingestion):
f. LUNG
Fish

	Check box if yes
Shortness of breath	☐
Repetitive coughing	☐
Wheezing	☐
Chest Tightness	☐

34. Specific symptoms of food allergy (through ingestion):
f. LUNG
Shellfish

	Check box if yes
Shortness of breath	☐
Repetitive coughing	☐
Wheezing	☐
Chest Tightness	☐

34. Specific symptoms of food allergy (through ingestion):
f. LUNG
Wheat

	Check box if yes
Shortness of breath	☐
Repetitive coughing	☐
Wheezing	☐
Chest Tightness	☐

34. Specific symptoms of food allergy (through ingestion):
f. LUNG
Soy/Tofu

	Check box if yes
Shortness of breath	☐
Repetitive coughing	☐
Wheezing	☐
Chest Tightness	☐

34. Specific symptoms of food allergy (through ingestion):
f. LUNG
Seeds

	Check box if yes
Shortness of breath	☐
Repetitive coughing	☐
Wheezing	☐
Chest Tightness	☐

34f. Name of Other Food Allergy #1 ______________________________

34. Specific symptoms of food allergy (through ingestion):
f. LUNG
Other Food Allergy #1

	Check box if yes
Shortness of breath	☐
Repetitive coughing	☐
Wheezing	☐
Chest Tightness	☐

34f. Name of Other Food Allergy #2 ______________________________

34. Specific symptoms of food allergy (through ingestion):
f. LUNG
Other Food Allergy #2

	Check box if yes
Shortness of breath	☐
Repetitive coughing	☐
Wheezing	☐
Chest Tighness	☐

34f. Name of Other Food Allergy #3 __________________________________

34. Specific symptoms of food allergy (through ingestion):
f. LUNG
Other Food Allergy #3

	Check box if yes
Shortness of breath	☐
Repetitive coughing	☐
Wheezing	☐
Chest Tightness	☐

34f. Name of Other Food Allergy #4 __________________________________

34. Specific symptoms of food allergy (through ingestion):
f. LUNG
Other Food Allergy #4

	Check box if yes
Shortness of breath	☐
Repetitive coughing	☐
Wheezing	☐
Chest Tightness	☐

34f. Name of Other Food Allergy #5 __________________________________

34. Specific symptoms of food allergy (through ingestion):
f. LUNG
Other Food Allergy #5

	Check box if yes
Shortness of breath	☐

Repetitive coughing	☐
Wheezing	☐
Chest Tightness	☐

34f. Name of Other Food Allergy #6 ____________________

34. Specific symptoms of food allergy (through ingestion):
f. LUNG
Other Food Allergy #6

	Check box if yes
Shortness of breath	☐
Repetitive coughing	☐
Wheezing	☐
Chest Tightness	☐

34. Specific symptoms of food allergy (through ingestion):
g. GUT / Síntomas de tripa / intestino
Cow's Milk/Dairy Products/Cheese

	Check box if yes
Stomach cramps/pain / Dolor de estómago	☐
Nausea / Náusea	☐
Vomiting / Vómito	☐
Diarrhea / Diarrea	☐
Bloating (swelling, gassy feeling) / Estómago hinchado	☐

34. Specific symptoms of food allergy (through ingestion):
g. GUT
Egg

	Check box if yes
Stomach cramps/pain	☐
Nausea	☐
Vomiting	☐
Diarrhea	☐
Bloating (swelling, gassy feeling)	☐

34. Specific symptoms of food allergy (through ingestion):
g. GUT
Peanut

	Check box if yes
Stomach cramps/pain	☐
Nausea	☐
Vomiting	☐
Diarrhea	☐
Bloating (swelling, gassy feeling)	☐

34. Specific symptoms of food allergy (through ingestion):
g. GUT
Tree Nuts

	Check box if yes
Stomach cramps/pain	☐
Nausea	☐
Vomiting	☐
Diarrhea	☐
Bloating (swelling, gassy feeling)	☐

34. Specific symptoms of food allergy (through ingestion):
g. GUT
Fish

	Check box if yes
Stomach cramps/pain	☐
Nausea	☐
Vomiting	☐
Diarrhea	☐
Bloating (swelling, gassy feeling)	☐

34. Specific symptoms of food allergy (through ingestion):
g. GUT
Shellfish

	Check box if yes
Stomach cramps/pain	☐
Nausea	☐
Vomiting	☐
Diarrhea	☐
Bloating (swelling, gassy feeling)	☐

34. Specific symptoms of food allergy (through ingestion):
g. GUT
Wheat

	Check box if yes
Stomach cramps/pain	☐
Nausea	☐
Vomiting	☐
Diarrhea	☐
Blaoting (swelling, gassy feeling)	☐

34. Specific symptoms of food allergy (through ingestion):
g. GUT
Soy/Tofu

	Check box if yes
Stomach cramps/pain	☐
Nausea	☐
Vomiting	☐
Diarrhea	☐
Bloating (swelling, gassy feeling)	☐

34. Specific symptoms of food allergy (through ingestion):
g. GUT
Seeds

	Check box if yes
Stomach cramps/pain	☐
Nausea	☐
Vomiting	☐
Diarrhea	☐
Bloating (swelling, gassy feeling)	☐

34g. Name of Other Food Allergy #1 ______________________

34. Specific symptoms of food allergy (through ingestion):
g. GUT
Other Food Allergy #1

	Check box if yes
Stomach cramps/pain	☐
Nausea	☐
Vomiting	☐
Diarrhea	☐

Bloating (swelling, gassy feeling) ☐

34g. Name of Other Food Allergy #2

____

34. Specific symptoms of food allergy (through ingestion):
g. GUT
Other Food Allergy #2

	Check box if yes
Stomach cramps/pain	☐
Nausea	☐
Vomiting	☐
Diarrhea	☐
Bloating (swelling, gassy feeling)	☐

34g. Name of Other Food Allergy #3

____

34. Specific symptoms of food allergy (through ingestion):
g. GUT
Other Food Allergy #3

	Check box if yes
Stomach cramps/pain	☐
Nausea	☐
Vomiting	☐
Diarrhea	☐
Bloating (swelling, gassy feeling)	☐

34g. Name of Other Food Allergy #4

____

34. Specific symptoms of food allergy (through ingestion):
g. GUT
Other Food Allergy #4

	Check box if yes
Stomach cramps/pain	☐
Nausea	☐
Vomiting	☐
Diarrhea	☐
Bloating (swelling, gassy feeling)	☐

34g. Name of Other Food Allergy #5

____

34. Specific symptoms of food allergy (through ingestion):
g. GUT
Other Food Allergy #5

	Check box if yes
Stomach cramps/pain	☐
Nausea	☐
Vomiting	☐
Diarrhea	☐
Bloating (swelling, gassy feeling)	☐

34g. Name of Other Food Allergy #6 __________________________________

34. Specific symptoms of food allergy (through ingestion):
g. GUT
Other Food Allergy #6

	Check box if yes
Stomach cramps/pain	☐
Nausea	☐
Vomiting	☐
Diarrhea	☐
Bloating (swelling, gassy feeling)	☐

34. Specific symptoms of food allergy (through ingestion):
h. CARDIOVACULAR / Síntomas de cardiovascular
Cow's Milk/Dairy Products/Cheese

	Check box if yes
Pale or turn blue / Piel pálida o azul	☐
Dizzy/Light-headed / Marceo	☐
Passing out/Fainting / Desmogo	☐

34. Specific symptoms of food allergy (through ingestion):
h. CARDIOVACULAR
Egg

	Check box if yes
Pale or turn blue	☐
Dizzy/Light-headed	☐
Passing out/Fainting	☐

34. Specific symptoms of food allergy (through ingestion):
h. CARDIOVACULAR
Peanut

	Check box if yes
Pale or turn blue	☐
Dizzy/Light-headed	☐
Passing out/Fainting	☐

34. Specific symptoms of food allergy (through ingestion):
h. CARDIOVACULAR
Treenut

	Check box if yes
Pale or turn blue	☐
Dizzy/Light-headed	☐
Passing out/Fainting	☐

34. Specific symptoms of food allergy (through ingestion):
h. CARDIOVACULAR
Fish

	Check box if yes
Pale or turn blue	☐
Dizzy/Light-headed	☐
Passing out/Fainting	☐

34. Specific symptoms of food allergy (through ingestion):
h. CARDIOVACULAR
Shellfish

	Check box if yes
Pale or turn blue	☐
Dizzy/Light-headed	☐
Passing out/Fainting	☐

34. Specific symptoms of food allergy (through ingestion):
h. CARDIOVACULAR
Wheat

	Check box if yes
Pale or turn blue	☐
Dizzy/Light-headed	☐
Passing out/Fainting	☐

34. Specific symptoms of food allergy (through ingestion):
h. CARDIOVACULAR
Soy/Tofu

	Check box if yes
Pale or turn blue	☐
Dizzy/Light-headed	☐
Passing out/Fainting	☐

34. Specific symptoms of food allergy (through ingestion):
h. CARDIOVACULAR
Seeds

	Check box if yes
Pale or turn blue	☐
Dizzy/Light-headed	☐
Passing out/Fainting	☐

34h. Name of Other Food Allergy #1 ______________________________

34. Specific symptoms of food allergy (through ingestion):
h. CARDIOVACULAR
Other Food Allergy #1

	Check box if yes
Pale or turn blue	☐
Dizzy/Light-headed	☐
Passing out/Fainting	☐

34h. Name of Other Food Allergy #2 ______________________________

34. Specific symptoms of food allergy (through ingestion):
h. CARDIOVACULAR
Other Food Allergy #2

	Check box if yes
Pale or turn blue	☐
Dizzy/Light-headed	☐
Passing out/Fainting	☐

34h. Name of Other Food Allergy #3 ______________________________

34. Specific symptoms of food allergy (through ingestion):
h. CARDIOVASCULAR
Other Food Allergy #3

	Check box if yes
Pale or turn blue	☐
Dizzy/Light-headed	☐
Passing out/Fainting	☐

34h. Name of Other Food Allergy #4 __________________________________

34. Specific symptoms of food allergy (through ingestion):
h. CARDIOVASCULAR
Other Food Allergy #4

	Check box if yes
Pale or turn blue	☐
Dizzy/Light-headed	☐
Passing out/Fainting	☐

34h. Name of Other Food Allergy #5 __________________________________

34. Specific symptoms of food allergy (through ingestion):
h. CARDIOVASCULAR
Other Food Allergy #5

	Check box if yes
Pale or turn blue	☐
Dizzy/Light-headed	☐
Passing out/Fainting	☐

34h. Name of Other Food Allergy #6 __________________________________

34. Specific symptoms of food allergy (through ingestion):
h. CARDIOVASCULAR
Other Food Allergy #6

	Check box if yes
Pale or turn blue	☐
Dizzy/Light-headed	☐
Passing out/Fainting	☐

35. Has your child ever experienced anaphylaxis (a life-threatening allergic reaction)?

¿Ha experimentado...anafilaxia? (Reacción alérgica que amenaza la vida)

◯ Yes
◯ No

35a. If yes, to what foods? (Select all that apply)

¿A qué tipo de comida?

	Yes	No
Cow's Milk/Dairy Products/Cheese	◯	◯
Egg	◯	◯
Peanut	◯	◯
Tree Nuts	◯	◯
Fish	◯	◯
Shellfish	◯	◯
Wheat	◯	◯
Soy/Tofu	◯	◯
Seeds	◯	◯
Other Food Allergy #1	◯	◯
Other Food Allergy #2	◯	◯
Other Food Allergy #3	◯	◯
Other Food Allergy #4	◯	◯
Other Food Allergy #5	◯	◯
Other Food Allergy #6	◯	◯

Cow's Milk/Dairy Products/Cheese
Number of episodes (lifetime) ________________________________

Cow's Milk/Dairy Products/Cheese
Number of episodes (in last year) ________________________________

Egg
Number of episodes (lifetime) ________________________________

Egg
Number of episodes (in last year) ________________________________

Peanut
Number of episodes (lifetime) ________________________________

Peanut
Number of episodes (in last year) ________________________________

Tree Nuts
Number of episodes (lifetime) ________________________________

Tree Nuts
Number of episodes (in last year) ________________________________

Fish
Number of episodes (lifetime) ________________________________

Fish
Number of episodes (in last year) ________________________________

Shellfish
Number of episdoes (lifetime) ______________________

Shellfish
Number of episdoes (in last year) ______________________

Wheat
Number of episdoes (lifetime) ______________________

Wheat
Number of episdoes (in last year) ______________________

Soy/Tofu
Number of episodes (lifetime) ______________________

Soy/Tofu
Number of episodes (in last year) ______________________

Seeds
Number of episodes (lifetime) ______________________

Seeds
Number of episodes (in last year) ______________________

Other Food Allergy #1 ______________________

Other Food Allergy #1
Number of episodes (lifetime) ______________________

Other Food Allergy #1
Number of episodes (in last year) ______________________

Other Food Allergy #2 ______________________

Other Food Allergy #2
Number of episodes (lifetime) ______________________

Other Food Allergy #2
Number of episodes (in last year) ______________________

Other Food Allergy #3 ______________________

Other Food Allergy #3
Number of episodes (lifetime) ______________________

Other Food Allergy #3
Number of episodes (in last year) ______________________

Other Food Allergy #4 ______________________

Other Food Allergy #4
Number of episodes (lifetime) ______________________________

Other Food Allergy #4
Number of episodes (in last year) ______________________________

Other Food Allergy #5 ______________________________

Other Food Allergy #5
Number of episodes (lifetime) ______________________________

Other Food Allergy #5
Number of episodes (in last year) ______________________________

Other Food Allergy #6 ______________________________

Other Food Allergy #6
Number of episodes (lifetime) ______________________________

Other Food Allergy #6
Number of episodes (in last year) ______________________________

36. How long does it usually take from eating the food to the onset of the allergic symptoms?

¿Después de comer la comida, cuánto tiempo toma para los síntomas a aparecer?

36. How long does it usually take from eating the food to the onset of the allergic symptoms for COW'S MILK/DAIRY PRODUCTS/CHEESE? (Time until onset in DAYS)
______________________________ (number of days)

36. How long does it usually take from eating the food to the onset of the allergic symptoms for COW'S MILK/DAIRY PRODUCTS/CHEESE? (Time until onset in HOURS)
______________________________ (number of hours)

36. How long does it usually take from eating the food to the onset of the allergic symptoms for COW'S MILK/DAIRY PRODUCTS/CHEESE? (Time until onset in MINUTES)
______________________________ (number of minutes)

36. How long does it usually take from eating the food to the onset of the allergic symptoms for EGGS? (Time until onset in DAYS)
______________________________ (number of days)

36. How long does it usually take from eating the food to the onset of the allergic symptoms for EGGS? (Time until onset in HOURS)
______________________________ (number of hours)

36. How long does it usually take from eating the food to the onset of the allergic symptoms for EGGS? (Time until onset in MINUTES)
______________________________ (number of minutes)

36. How long does it usually take from eating the food to the onset of the allergic symptoms for PEANUTS? (Time until onset in DAYS)

____
(number of days)

36. How long does it usually take from eating the food to the onset of the allergic symptoms for PEANUTS? (Time until onset in HOURS)

____
(number of hours)

36. How long does it usually take from eating the food to the onset of the allergic symptoms for PEANUTS? (Time until onset in MINUTES)

____
(number of minutes)

36. How long does it usually take from eating the food to the onset of the allergic symptoms for TREE NUTS? (Time until onset in DAYS)

____
(number of days)

36. How long does it usually take from eating the food to the onset of the allergic symptoms for TREE NUTS? (Time until onset in HOURS)

____
(number of hours)

36. How long does it usually take from eating the food to the onset of the allergic symptoms for TREE NUTS? (Time until onset in MINUTES)

____
(number of minutes)

36. How long does it usually take from eating the food to the onset of the allergic symptoms for FISH? (Time until onset in DAYS)

____
(number of days)

36. How long does it usually take from eating the food to the onset of the allergic symptoms for FISH? (Time until onset in HOURS)

____
(number of hours)

36. How long does it usually take from eating the food to the onset of the allergic symptoms for FISH? (Time until onset in MINUTES)

____
(number of minutes)

36. How long does it usually take from eating the food to the onset of the allergic symptoms for SHELLFISH? (Time until onset in DAYS)

____
(number of days)

36. How long does it usually take from eating the food to the onset of the allergic symptoms for SHELLFISH? (Time until onset in HOURS)

____
(number of hours)

36. How long does it usually take from eating the food to the onset of the allergic symptoms for SHELLFISH? (Time until onset in MINUTES)

____
(number of minutes)

36. How long does it usually take from eating the food to the onset of the allergic symptoms for WHEAT? (Time until onset in DAYS)

____
(number of days)

36. How long does it usually take from eating the food to the onset of the allergic symptoms for WHEAT? (Time until onset in HOURS)

____
(number of hours)

36. How long does it usually take from eating the food to the onset of the allergic symptoms for WHEAT? (Time until onset in MINUTES)	______________________ (number of minutes)
36. How long does it usually take from eating the food to the onset of the allergic symptoms for SOY/TOFU? (Time until onset in DAYS)	______________________ (number of days)
36. How long does it usually take from eating the food to the onset of the allergic symptoms for SOY/TOFU? (Time until onset in HOURS)	______________________ (number of hours)
36. How long does it usually take from eating the food to the onset of the allergic symptoms for SOY/TOFU? (Time until onset in MINUTES)	______________________ (number of minutes)
36. How long does it usually take from eating the food to the onset of the allergic symptoms for SEEDS? (Time until onset in DAYS)	______________________ (number of days)
36. How long does it usually take from eating the food to the onset of the allergic symptoms for SEEDS? (Time until onset in HOURS)	______________________ (number of hours)
36. How long does it usually take from eating the food to the onset of the allergic symptoms for SEEDS? (Time until onset in MINUTES)	______________________ (number of minutes)
36. Name of Other Food Allergy #1	______________________
36. How long does it usually take from eating the food to the onset of the allergic symptoms for OTHER FOOD ALLERGY #1? (Time until onset in DAYS)	______________________ (number of days)
36. How long does it usually take from eating the food to the onset of the allergic symptoms for OTHER FOOD ALLERGY #1? (Time until onset in HOURS)	______________________ (number of hours)
36. How long does it usually take from eating the food to the onset of the allergic symptoms for OTHER FOOD ALLERGY #1? (Time until onset in MINUTES)	______________________ (number of minutes)
36. Name of Other Food Allergy #2	______________________
36. How long does it usually take from eating the food to the onset of the allergic symptoms for OTHER FOOD ALLERGY #2? (Time until onset in DAYS)	______________________ (number of days)

36. How long does it usually take from eating the food to the onset of the allergic symptoms for OTHER FOOD ALLERGY #2? (Time until onset in HOURS)	______________________ (number of hours)
36. How long does it usually take from eating the food to the onset of the allergic symptoms for OTHER FOOD ALLERGY #2? (Time until onset in MINUTES)	______________________ (number of minutes)
36. Name of Other Food Allergy #3	______________________
36. How long does it usually take from eating the food to the onset of the allergic symptoms for OTHER FOOD ALLERGY #3? (Time until onset in DAYS)	______________________ (number of days)
36. How long does it usually take from eating the food to the onset of the allergic symptoms for OTHER FOOD ALLERGY #3? (Time until onset in HOURS)	______________________ (number of hours)
36. How long does it usually take from eating the food to the onset of the allergic symptoms for OTHER FOOD ALLERGY #3? (Time until onset in MINUTES)	______________________ (number of minutes)
36. Name of Other Food Allergy #4	______________________
36. How long does it usually take from eating the food to the onset of the allergic symptoms for OTHER FOOD ALLERGY #4? (Time until onset in DAYS)	______________________ (number of days)
36. How long does it usually take from eating the food to the onset of the allergic symptoms for OTHER FOOD ALLERGY #4? (Time until onset in HOURS)	______________________ (number of hours)
36. How long does it usually take from eating the food to the onset of the allergic symptoms for OTHER FOOD ALLERGY #4? (Time until onset in MINUTES)	______________________ (number of minutes)
36. Name of Other Food Allergy #5	______________________
36. How long does it usually take from eating the food to the onset of the allergic symptoms for OTHER FOOD ALLERGY #5? (Time until onset in DAYS)	______________________ (number of days)
36. How long does it usually take from eating the food to the onset of the allergic symptoms for OTHER FOOD ALLERGY #5? (Time until onset in HOURS)	______________________ (number of hours)

36. How long does it usually take from eating the food to the onset of the allergic symptoms for OTHER FOOD ALLERGY #5? (Time until onset in MINUTES)	_______________ (number of minutes)
36. Name of Other Food Allergy #6	_______________
36. How long does it usually take from eating the food to the onset of the allergic symptoms for OTHER FOOD ALLERGY #6? (Time until onset in DAYS)	_______________ (number of days)
36. How long does it usually take from eating the food to the onset of the allergic symptoms for OTHER FOOD ALLERGY #6? (Time until onset in HOURS)	_______________ (number of hours)
36. How long does it usually take from eating the food to the onset of the allergic symptoms for OTHER FOOD ALLERGY #6? (Time until onset in MINUTES)	_______________ (number of minutes)

37. What treatment(s) has/have your child used for the most severe COW's MILK/DAIRY PRODUCTS/CHEESE allergic reactions? (Select all that apply)

¿Qué tipo de tratamiento se ha utilizado... para tratar las reacciones alérgicas?

	Check box if yes
Benadryl Only / Solamente Benadryl	☐
Epi Pen / Epipen	☐
Doctor's Office / Oficina del doctor	☐
ER / Sala de emergencia	☐
Hospital / Hospital	☐
ICU / UCI	☐

37. What treatment(s) has/have your child used for the most severe EGG allergic reactions? (Select all that apply)

	Check box if yes
Benadryl Only	☐
Epi Pen	☐
Doctor's Office	☐
ER	☐
Hospital	☐
ICU	☐

37. What treatment(s) has/have your child used for the most severe PEANUT allergic reactions? (Select all that apply)

	Check box if yes
Benadryl Only	☐
Epi Pen	☐
Doctor's Office	☐
ER	☐
Hospital	☐
ICU	☐

37. What treatment(s) has/have your child used for the most severe TREE NUTS allergic reactions? (Select all that apply)

	Check box if yes
Benadryl Only	☐
Epi Pen	☐
Doctor's Office	☐
ER	☐
Hospital	☐
ICU	☐

37. What treatment(s) has/have your child used for the most severe FISH allergic reactions? (Select all that apply)

	Check box if yes
Benadryl Only	☐
Epi Pen	☐
Doctor's Office	☐
ER	☐
Hospital	☐
ICU	☐

37. What treatment(s) has/have your child used for the most severe SHELLFISH allergic reactions? (Select all that apply)

	Check box if yes
Benadryl Only	☐
Epi Pen	☐
Doctor's Office	☐
ER	☐
Hospital	☐
ICU	☐

37. What treatment(s) has/have your child used for the most severe WHEAT allergic reactions? (Select all that apply)

	Check box if yes
Benadryl Only	☐
Epi Pen	☐
Doctor's Office	☐
ER	☐
Hospital	☐
ICU	☐

37. What treatment(s) has/have your child used for the most severe SOY/TOFU allergic reactions? (Select all that apply)

	Check box if yes
Benadryl Only	☐
Epi Pen	☐
Doctor's Office	☐
ER	☐
Hospital	☐
ICU	☐

37. What treatment(s) has/have your child used for the most severe SEEDS allergic reactions? (Select all that apply)

	Check box if yes
Benadryl Only	☐
Epi Pen	☐
Doctor's Office	☐
ER	☐
Hospital	☐
ICU	☐

37. Name of Other Food Allergy #1

____

37. What treatment(s) has/have your child used for the most severe OTHER FOOD ALLERGY #1 allergic reactions? (Select all that apply)

	Check box if yes
Benadryl Only	☐
Epi Pen	☐
Doctor's Office	☐
ER	☐
Hospital	☐

ICU ☐

37. Name of Other Food Allergy #2

____

37. What treatment(s) has/have your child used for the most severe OTHER FOOD ALLERGY #2 allergic reactions? (Select all that apply)

	Check box if yes
Benadryl Only	☐
Epi Pen	☐
Doctor's Office	☐
ER	☐
Hospital	☐
ICU	☐

37. Name of Other Food Allergy #3

____

37. What treatment(s) has/have your child used for the most severe OTHER FOOD ALLERGY #3 allergic reactions? (Select all that apply)

	Check box if yes
Benadryl Only	☐
Epi Pen	☐
Doctor's Office	☐
ER	☐
Hospital	☐
ICU	☐

37. Name of Other Food Allergy #4

____

37. What treatment(s) has/have your child used for the most severe OTHER FOOD ALLERGY #4 allergic reactions? (Select all that apply)

	Check box if yes
Benadryl Only	☐
Epi Pen	☐
Doctor's Office	☐
ER	☐
Hospital	☐
ICU	☐

37. Name of Other Food Allergy #5

____

37. What treatment(s) has/have your child used for the most severe OTHER FOOD ALLERGY #5 allergic reactions? (Select all that apply)

	Check box if yes
Benadryl Only	☐
Epi Pen	☐
Doctor's Office	☐
ER	☐
Hospital	☐
ICU	☐

37. Name of Other Food Allergy #6 ______________________

37. What treatment(s) has/have your child used for the most severe OTHER FOOD ALLERGY #6 allergic reactions? (Select all that apply)

	Check box if yes
Benadryl Only	☐
Epi Pen	☐
Doctor's Office	☐
ER	☐
Hospital	☐
ICU	☐

Section II. Family History

38. What is your present marital status?

¿Mamá, Ud. Está?
Casada
Viuda
Divorciada
Separada
Soltera

◯ Married
◯ Widowed
◯ Divorced
◯ Separated
◯ Single

39. What is the highest grade of school you have completed to date?

¿Qué grado de escuela Ud. terminó?

◯ No school
◯ Elementary school
◯ Some secondary school (9th grade and above)
◯ High school graduate or GED
◯ Some college
◯ College degree
◯ Graduate school degree
◯ Post Graduate (PhD/MD/Other)

40. Are you currently working for pay?

¿Ud. Está trabajando?

◯ Yes
◯ No

41. What is your occupation/job title?

¿Cuál es su ocupación?

____

What field does your occupation fall under?	◯ Not Applicable<br>◯ Management/Business/Administration<br>◯ Financial/Computer/Mathematical<br>◯ Architecture and Engineering<br>◯ Life, Physical, and Social Science<br>◯ Legal Occupations<br>◯ Education, Training, and Library<br>◯ Sales, Arts, Design, Entertainment, and Media<br>◯ Athletics (Sports, Dancing, etc)<br>◯ Healthcare<br>◯ Food Preparation and Serving<br>◯ Building and Grounds Cleaning and Maintenance<br>◯ Personal Care and Service<br>◯ Farming, Fishing, and Forestry<br>◯ Construction Trades<br>◯ Extraction Workers<br>◯ Installation, Maintenance, and Repair Workers<br>◯ Production Occupations<br>◯ Transportation and Material Moving<br>◯ Military Specific
42. What was your total household income last year, before taxes? (INCLUDES PUBLIC ASSISTANCE)<br><br>¿Por el último año, ¿Cuántos fueron su ingresos totales de hogar?	◯ < $5,000<br>◯ $5,000-9,999<br>◯ $10,000-14,999<br>◯ $15,000-19,999<br>◯ $20,000-24,999<br>◯ $25,000-29,999<br>◯ $30,000-34,999<br>◯ $35,000-39,999<br>◯ $40,000-49,999<br>◯ $50,000-59,999<br>◯ $60,000-79,999<br>◯ $80,000-99,999<br>◯ > $100,000<br>◯ Unsure
43. What is your current height in FEET?<br><br>¿Qué es su altura actual?	______________________
43. What is your current height in INCHES?<br><br>¿Qué es su altura actual?<br>Pulgudas	______________________
43. What is your current height in CENTIMETERS?<br><br>¿Qué es su altura actual?<br>Centimetros	______________________
44. What is your current weight (IN POUNDS)?<br><br>¿Su peso actual?	______________________<br>(pounds)
44. What is your current weight (IN KILOGRAMS)?	______________________<br>(kilograms)

45a. Can I ask what your child's biological father's height and weight is?<br><br>¿Puedo preguntar sobre el padre?	◯ Yes<br>◯ No
45b. What is the baby's father's current height (IN FEET)?<br><br>¿Altura de parde de...?	________________<br>(feet)
45b. What is the baby's father's current height (IN INCHES)?<br><br>¿Altura de parde de...?	________________<br>(inches)
45b. What is the baby's father's current height (IN CENTIMETERS)?<br><br>¿Altura de parde de...?	________________<br>(centimeters)
45b. Check box if mother is unsure of baby's father's current height	☐ Unsure
46. What is the baby's father's current weight (IN POUNDS)?<br><br>¿Peso de padre de...?	________________<br>(pounds)
46. What is the baby's father's current weight (IN KILOGRAMS)?<br><br>¿Peso de padre de...?	________________<br>(kilograms)
46. Check box if mother is unsure of baby's father's current weight	☐ Unsure
47. Do you have a personal history of asthma?<br><br>¿Ud. Tenido asma?	◯ No<br>◯ Yes I have it now<br>◯ Yes, only when I was a child, but I outgrew it<br>◯ Unsure
If asthma outgrown, at what age?<br>(YEARS)	________________<br>(age in years when mother outgrew asthma)
If asthma outgrown, at what age?<br>(MONTHS)	________________<br>(age in months when mother outgrew asthma)
If YES, was your asthma diagnosed by a doctor?<br><br>¿Fue diagnosticado por un doctor?	◯ Yes<br>◯ No<br>◯ Unsure
How old were you when your asthma was first diagnosed by a doctor?<br><br>¿Cuándo?	◯ Yes, I remember<br>◯ Unsure

How old were you when your asthma was first diagnosed by a doctor? (AGE IN YEARS)	__________________ (years)
How old were you when your asthma was first diagnosed by a doctor? (AGE IN MONTHS)	__________________ (months)
48. Have you ever used an inhaler or a nebulizer?<br><br>¿Ha usado Ud. un inhalador?	◯ Yes<br>◯ No<br>◯ Unsure
49. Do you have Eczema?<br><br>¿Ha tenido Ud. Eccema?	◯ Yes, I have it now<br>◯ Yes, only when I was a child, but I outgrew it<br>◯ No<br>◯ Unsure
If yes, only when I was a baby, but outgrew by:<br>Years	__________________ (Years)
If yes, only when I was a baby, but outgrew by:<br>Months	__________________ (Months)
If YES, was your eczema diagnosed by a doctor?<br><br>¿Fue diagnosticado por un doctor?	◯ Yes<br>◯ No<br>◯ Unsure
How old were you when your eczema was first diagnosed by a doctor?<br><br>¿Cuándo?	◯ Yes, I remember<br>◯ Unsure
How old were you when your eczema was first diagnosed by a doctor? (AGE IN YEARS)	__________________ (years)
How old were you when your eczema was first diagnosed by a doctor? (AGE IN MONTHS)	__________________ (months)
50. Have you ever used a steroid cream (like hydrocortisone cream or triamcinolone cream), including creams, lotions, and oitments containing steroids?<br><br>¿Ha usado ud. alguna crema que contiene esteroides (como hidrocortisona)?	◯ Yes<br>◯ No<br>◯ Unsure
51. Do you have hay fever or seasonal allergies?<br><br>¿Tiene Ud. alergias estacionales?	◯ Yes, I have it now<br>◯ Yes, only when I was a child, but I outgrew it<br>◯ No<br>◯ Unsure
How old were you when you outgrew your hay fever or seasonal allergies?<br>Years	__________________ (Years)

How old were you when you outgrew your hay fever or seasonal allergies?<br>Months	__________________________________<br>(Months)
If YES, was your hay fever diagnosed by a doctor?<br><br>¿Fue diagnosticado por un doctor?	◯ Yes<br>◯ No<br>◯ Unsure
How old were you when your hay fever was first diagnosed by a doctor?<br><br>¿Cuándo?	◯ Yes, I remember<br>◯ Unsure
How old were you when your hay fever was first diagnosed by a doctor? (AGE IN YEARS)	__________________________________<br>(years)
How old were you when your hay fever was first diagnosed by a doctor? (AGE IN MONTHS)	__________________________________<br>(months)
Which season(s) do you have seasonal allergies? (select all that apply)<br><br>Primavera<br>Verano<br>Otoño<br>Invierno<br>Todo el año	☐ Spring<br>☐ Summer<br>☐ Autumn<br>☐ Winter<br>☐ Year round<br>☐ Unsure
52. Do you have drug allergies?<br><br>¿Tiene Ud. alergias a algunas medicinas?	◯ Yes<br>◯ No<br>◯ Unsure
If YES, specify the drug(s)	__________________________________<br>(use "," to separate)
If YES, was your drug allergy diagnosed by a doctor?<br><br>¿Fue diagnosticado por un doctor?	◯ Yes<br>◯ No
How old were you when your drug allergy was first diagnosed by a doctor?<br><br>¿Cuándo?	◯ Yes, I remember<br>◯ Unsure
How old were you (AGE IN YEARS) when first diagnosed by a doctor with a drug allergy?	__________________________________<br>(years)
How old were you (AGE IN MONTHS) when first diagnosed by a doctor with a drug allergy?	__________________________________<br>(months)

53. Have you ever used anti-allergy medications? (ie Benadryl, Zyrtex, Claritin, Atarax, Dimetapp)<br><br>¿Ha usado Ud. Medicina anti-alergia?	◯ Yes<br>◯ No<br>◯ Unsure
54. Do you have any allergies triggered by the environment that was diagnosed by your doctor?<br><br>¿Tiene Ud. otras alergias diagnosticas por un doctor?	◯ Yes<br>◯ No<br>◯ Unsure
If YES, what type? (select all that apply)<br><br>Gato<br>Pero<br>Cucaracha<br>Moho<br>Polen<br>Polvo	☐ Cat<br>☐ Cockroach<br>☐ Dog<br>☐ Dust Mite<br>☐ Mold<br>☐ Pollen<br>☐ Other<br>☐ Unsure
If OTHER, specify	______________________________
55. Are you allergic to insect stings?<br><br>¿Ha sido Ud. picado por un abeja o avispa/ avispón? ¿Tuvo Ud. una reacción alérgica a la picadura?	◯ Yes<br>◯ No<br>◯ Don't know/Never been stung
If YES, 1) What type of insect?<br><br>¿Qué tipo?	◯ Bee<br>◯ Wasp<br>◯ Yellow Jacket
If YES, 2) Is it a severe allergy (that is, difficulty breathing, need epi pen or to go to the hospital)?<br><br>¿Es una alergia muy grave?	◯ Yes<br>◯ No<br>◯ Unsure
56. Do you have food allergies?<br><br>¿Tiene Ud. alergias alimentales?	◯ Yes, I have it now<br>◯ Yes, only when I was a child, but outgrew<br>◯ Unsure<br>◯ No
If OUTGREW, by what age (IN YEARS)?	______________________________
If OUTGREW, by what age (IN MONTHS)?	______________________________
If YES, was your food allergy diagnosed by a doctor?<br><br>¿Fue diagnosticado por un doctor?	◯ Yes<br>◯ No<br>◯ Unsure
How old were you when first diagnosed by a doctor?<br><br>¿Cuándo?	◯ Yes, I remember<br>◯ Unsure
How old were you (AGE IN YEARS) when first diagnosed by a doctor?	______________________________<br>(years)

How old were you (AGE IN MONTHS) when first diagnosed by a doctor?

____
(months)

57. If you ever had a food allergy, what type of food(s) were you allergic to?

¿A qué tipo de comida tiene Ud. alergias?

	Yes	No
Cow's milk/dairy products/cheese	○	○
Egg Whites	○	○
Peanut	○	○
Tree Nuts	○	○
Fish	○	○
Shellfish	○	○
Wheat	○	○
Soy/Tofu	○	○
Seeds	○	○
Other Foods	○	○

If you have ever had an allergy to TREE NUTS, please choose the specific type (select all that apply)

¿Qué tipo de nueces?

- ☐ Almond
- ☐ Cashew
- ☐ Filbert/Hazel
- ☐ Walnut
- ☐ Brazil
- ☐ Macadamia
- ☐ Pecan
- ☐ Pine
- ☐ Pistachio
- ☐ Other

If other tree nuts, specify:

____

If you have ever had an allergy to FISH, please choose the specific type (select all that apply)

¿Qué tipo de pescado?

- ☐ Salmon
- ☐ Tuna
- ☐ Catfish
- ☐ Cod
- ☐ Flounder
- ☐ Halibut
- ☐ Trout
- ☐ Bass
- ☐ Other

If other fish, specify:

____

If you have ever had an allergy to SHELLFISH, please choose the specific type (select all that apply)

¿Qué tipo de mariscos?

- ☐ Shrimp
- ☐ Crab
- ☐ Lobster
- ☐ Clam
- ☐ Oyster
- ☐ Mussels
- ☐ Other

If other shellfish, specify: ______________________

If you have ever had an allergy to SEEDS, please choose the specific type (select all that apply)

¿Qué tipo de semillas?

- [ ] Sesame
- [ ] Sunflower
- [ ] Pumpkin
- [ ] Other

If other seeds, specify: ______________________

If other foods not listed, specify: ______________________

Section III. Home Environment

58. Here are some questions about your current home:

Estas preguntas son sobre su hogar actual

a) How long have you lived in your current home? (TIME IN YEARS) ______________________

¿Cuántos años ha vivido Ud. en su Casa?

a) How long have you lived in your current home? (TIME IN MONTHS) ______________________

¿Cuántos años ha vivido Ud. en su Casa?

b) What type of housing is your home?

¿Qué tipo de casa? ¿Casa o apartamento?

- ◯ Single family
- ◯ Duplex
- ◯ Row House
- ◯ Condo/Apartment
- ◯ Trailer Home
- ◯ Shelter
- ◯ Other

If Others, specify: ______________________

c) # of bedrooms ______________________

¿Cuántas habitaciones tiene en la casa?

d) # of bathrooms ______________________

¿Cuántos baños?

e) # of people who permanently live in your home ______________________

¿Cuántas personas viven allí?

f) What type of fuel do you use for heating your home?<br><br>¿Qué usa Ud. para calentar la casa?<br>Aceite<br>Electricidad<br>Gas	◯ Gas<br>◯ Electricity<br>◯ Oil<br>◯ Other<br>◯ Unsure
If Others, specify:	______________________________<br>(other type of fuel used for heating the home)
g) What type of stove do you use for cooking?<br><br>¿Y para cocinar?<br>Gas<br>Electricidad	◯ Gas<br>◯ Electricity<br>◯ Other<br>◯ Unsure
If Others, specify:	______________________________<br>(other type of fuel used for cooking)
h) Do you have any wall to wall carpet in your home?<br><br>¿Hay alfombra de pared a pared en alguna parte de la casa?	◯ Yes<br>◯ No<br>◯ Unsure
If yes, specify location:<br><br>Sala<br>Sala de estar<br>Comendar<br>Cocina<br>Habitaciones<br>Sótano<br>Baño	☐ Living room<br>☐ Family room<br>☐ Dining room<br>☐ Kitchen<br>☐ Bedroom (master) parents<br>☐ Bedroom index child<br>☐ Bedroom Sib#1<br>☐ Bedroom Sib#2<br>☐ Basement<br>☐ Bathroom
i) Approximately how old is the building/apartment/home you live in?<br><br>¿Cuántos años tiene desde su casa ha sido consumado?	◯ 10 years or less<br>◯ 11-25 years<br>◯ 26-50 years<br>◯ 51-75 years<br>◯ Greater than 75 years old<br>◯ Don't know
59. Have you (mother of the child) ever smoked cigarettes, cigars, or pipes?<br><br>¿Ud. fuma? (¿Ha fumado?)<br>Nunca<br>¿Ha dejado fumar?<br>¿Cuándo dejó, antes o después de queda embarazada con...?	◯ No, I never smoked<br>◯ Yes, I currently smoke<br>◯ I used to smoke but I quit before becoming pregnant with index child<br>◯ I used to smoke but quit after becoming pregnant with index child
If yes, what do/did you smoke?<br><br>Cigarrillos<br>Cigarros<br>Pipa	◯ Cigarettes<br>◯ Cigars<br>◯ Pipes

60. If yes to Q 59,<br>Do you smoke inside the home?<br><br>¿Fuma en la casa?	◯ Yes<br>◯ No
How many (cigarettes, cigars, pipes) do you smoke PER DAY (Regardless of indoor or outdoor)<br><br>¿Cuántos cigarrillos fuman por día?	__________________________________
OR, How many (cigarettes, cigars, pipes) do you smoke PER WEEK (Regardless of indoor or outdoor)<br><br>¿Cuántos cigarrillos fuman por semana?	__________________________________
61. Can I ask you about your child's biological father's smoking status?<br><br>¿Puedo preguntar sobre el padre?	◯ Yes<br>◯ No
61a. Has the father of the child ever smoked cigarettes, cigars, or pipes?<br><br>¿Y el padre de ... ha fumado?	◯ No, he never smoked<br>◯ Yes, he currently smokes<br>◯ He used to smoke but he quit before I became pregnant with index child<br>◯ He used to smoke but he quit after I became pregnant with index child
If yes, what does/did he smoke?<br><br>Cigarrillos<br>Cigarros<br>Pipa	◯ Cigarettes<br>◯ Cigars<br>◯ Pipes
62. If yes to Q 61,<br>Does he smoke inside the home?<br><br>¿Fuma él en la casa?	◯ Yes<br>◯ No
How many (cigarettes, cigars, pipes) does he smoke PER DAY (Regardless of indoor or outdoor)?<br><br>¿Cuántos cigarrillos fuman por día?	__________________________________<br>(per day)
OR, How many (cigarettes, cigars, pipes) does he smoke PER WEEK (Regardless of indoor or outdoor)?<br><br>¿Cuántos cigarrillos fuman por semana?	__________________________________<br>(per week)
63. How many other people who live in your home smoke cigarettes (not including the mother and father of the child)?<br><br>¿Hay otras personas en la casa que fuman?	__________________________________
64. How many of them smoke inside the home?<br><br>¿Cuántas personas fuman en la casa?	__________________________________

65. Total numbers of cigarettes smoked inside your home per day (NOT INCLUDING AMOUNT SMOKED by yourself and the father of your child)? __________________________________

66. Do you currently have any pets in your home?

¿Tiene Ud. mascotas o animales en la casa?

◯ Yes
◯ No

If yes, specify type of pet and how many of each type:

	Yes	No
Cat / Gato	◯	◯
Dog / Pero	◯	◯
Reptiles / Reptiles	◯	◯
Rabbit / Conejo	◯	◯
Fish / Pez	◯	◯
Guinea Pig / conejillo de indias	◯	◯
Birds / Pájaro	◯	◯
Others	◯	◯

How many cats? __________________________________

How many dogs? __________________________________

How many reptiles? __________________________________

How many rabbits? __________________________________

How many fish? __________________________________

How many guinea pigs? __________________________________

How many birds? __________________________________

If others, specify: __________________________________

How many others? __________________________________

67. Does the house you live in have any cockroaches?

¿Hay cucarachas en la casa?

◯ Yes
◯ No
◯ Unsure

68. Does the house you live in have any mice/rats?<br><br>¿Hay ratones o ratas en las casa?	◯ Yes<br>◯ No<br>◯ Unsure
69. Does the house you live in have any visible mold, mildew, water damage, leakage or seepage?<br><br>¿Hay moho o daños por agua en la casa?	◯ Yes<br>◯ No<br>◯ Unsure
70. Do you currently live in a farming environment?<br><br>¿Ud. no vive en una granja, verdad?	◯ Yes<br>◯ No<br>◯ Unsure

Supplemental Table 3

BBC Variable Collection

Self-reported questionnaire measurements Preterm Study

Demographics: Age, marital status, occupation, income, highest level of educational attainment, zipcode, number of children (if any), ethnicity and race of mother, biologic father of baby and mother's parents, insurance
Home Environment number of bedrooms, number of bathrooms, neighborhood violence, living outside of the US during pregnancy, length of time in home, homelessness in pregnancy, living in a shelter, number of people in household, presence of pets and pests (mice, rats, cockroaches), type of heating and cooking fuel, presence of carpet, mold, water leakage and seepage, age of home
Life course characteristics: Retrospective report of mother's own birthweight and gestational age, mother's place of birth, mother and biological father of pregnancy, maternal parental place of birth, public health assistance, native language, ability to speak English, if foreign born, years living in U.S.
Health-related behaviors: Diet in pregnancy, smoking of cigarettes, cigars, e-cigarettes, pipes, chewing tobacco age began smoking, duration (ever, 3 months prior to conception and in each trimester until delivery), amount smoked daily, alcohol consumption, caffeine intake, physical activity, illicit drug use (ever and in pregnancy), vitamin intake, herbal supplement use, weeks at prenatal visit initiation, number of prenatal appointments attended and missed, daily physical activity
Mental health- desired or undesired pregnancy, Perceived Stress Scale, level of support during pregnancy from family and partner, reported job stress
Reproductive history- gravidity, parity, number of pregnancies (including their outcomes and pregnancy complications), contraception use, condom use in pregnancy, menstrual patterns, genital and urinary tract infections
Cardiometabolic health own history of diagnosis of hypertension and diabetes
Respiratory health (repeatedly asked about diagnoses and symptoms of asthma and bronchitis)
Medication use prescribed and over the counter use and frequency and duration in pregnancy
Asthma and Allergy own history of food and medication allergy in mother and biological father of baby

Obstetric data abstracted from medical records

Mother Repeated measurements of weight and blood pressure beginning one year prior to pregnancy through postpartum
Derived (from repeated blood pressure and proteinuria measurements, presence and location of edema (hands, legs, face, sudden onset weight gain, presence of blurred vision, severe headache, decreased urinary output and physician diagnosis) hypertensive disorders of pregnancy (pre-existing hypertension, gestational hypertension, pre-eclampsia, pre-eclampsia superimposed on pre-existing hypertension and eclampsia

Medical record recorded diagnoses of pre-existing diabetes, gestational diabetes or failed 3 hour glucose tolerance test, placenta previa, HELLP syndrome, placental abruption, oligohydramnios, polyhydramnios, and other complications of pregnancy

Medical History hyperthyroidism, hypothyroidism, endometriosis, uterine myoma, uterine malformation, pelvic inflammatory disease, anemia, polycystic ovarian disease, malignant tumors, allergies, eczema, auto-immune disease, infertility, seizure disorder, abdominal operations

Blood group and Rh, urine dips for presence of glucose and/or protein, GBS status, complete blood counts one year prior to and during pregnancy, rubella and varicella immunity, genital tract infections and treatment, tuberculosis, amniocentesis or chorionic villus sampling

Fetal Fibronectin

Triple Screen (AFP, hCG, Estriol**)**

Hospital admissions

Ultrasound results (fetal growth, weight, biometry, abnormal fetal and cord findings)

Preterm contractions and management if present

Mode of delivery

Initiation of labor (contractions, rupture of membranes, both contractions and rupture of membranes together, medical induction)

Number of prenatal visits

Location of prenatal care

Type of provider physician or nurse midwife

Length of time from membrane rupture to delivery

Placental weight and pathology reports

Postnatal health- length of stay, postpartum complications, transfusion of blood products

Geocodes

Newborn

Blood group and Rh, labs through discharge

Gender

Apgars 1, 5, and 10 minutes

Head ultrasounds

Birthweight

Gestational age (by LMP, early <20 week ultrasound, Dubowitz or New Ballard score)

Head circumference

Length

Date and time of delivery

Presence of birth defects

Length of hospitalization

Medical complications (ROP, IVH, NEC, PDA)

Newborn Infections TORCH, CMV, congenital syphilis

Children’s Health Study

Baseline Questionnaire

Demographics zip code, income, occupation of mother and father, educational attainment, current marital status
Family Composition Father’s age, birth month and year, biological siblings age, birth month and year
Home Environment length of time at current address, type of housing (single family, condo, apartment, trailer home, shelter or homeless), number of bedrooms and bathrooms, total number of people living in residence, children’s ages and gender, heating and cooking fuel, age of home, carpeting in home, mold, mildew, water damage or seepage in home, number of smokers living in in home, exposure to second hand smoke, pets (type and number) mother and father’s smoking status, childcare utilization, type of childcare (in-home, daycare center), number of other children in childcare setting
Breastfeeding and solid food introduction duration of exclusive breast feeding, age at formula introduction type of formula (nonfat milk and whey protein, soy, elemental formulas, casein hydrolysate), age of whole milk introduction, age of solid food introduction and first foods introduced, mother’s diet during exclusive breastfeeding
Allergy and Illness in family Paternal and sibling history of illness and food allergy, study child’s Illness type in first year of life, food allergies in study child and specific symptoms and severity (respiratory, dermal, gut, throat, cardiovascular) time from ingestion to symptomatic onset, treatment for allergic symptoms (Benadryl or other antihistamines, Epi-pen , doctor visit, emergency room visit, hospitalization) maternal history of food, seasonal, insect, and medication allergic reactions, maternal age at onset of allergies
Asthma and eczema mother and study child age of symptom onset and treatment, study child wheezing in first 3 years of life, physician diagnosis of asthma
Pubertal Development repeated self-administered questionnaire completed by child or with help from mother- female: age of first menstruation, menstrual bleeding duration, intervals in days, presence of dysmenorrhea, presence of pubic and underarm hair, and breast development stage; male pubertal development- facial, pubic and leg hair, penile development stage
Development M-Chat™, Social Communication Questionnaire, Social Responsiveness Scale™, ADOS-®2
Pandemic SarsCoV-2 Hardships and attitudes (2020) positive testing for Covid-19 in household, childcare disruption, job loss, food insecurity, healthcare utilization, number of children in the home and grades in school, children with IEPs and 504s, use of telemedicine, attitudes towards telemedicine, missed healthcare visits of children and mother, transportation use, household composition, living situation (own home, renter, temporary staying somewhere, shelter), housing insecurity, risk for housing eviction, smokers in home, stress levels, school closure and remote learning attitudes, physical activity, SarsCoV-2 vaccine receipt attitudes for self and children

Follow-up clinical assessments

Anthropometry: maternal report of paternal weight and height, maternal and study child repeated waist, height and weight measurements, body composition (weight, BMI, fat%, basal metabolic rate, impedance, fat mass, fat free mass, total body water)
Respiratory Health Pulmonary Function Testing and Incentive Oscillating Spirometry

Follow-up questionnaires (added variables administered to Baseline Questionnaire)

Nutritional and vitamin use and supplementation
Child dietary weekly intake
Repeated demographics
Television, computer and video game screen time
Physical activity, outside play, sports participation
Sleep Habits Questionnaire

Record linkage

Preterm Study with Children's Health Study
Death rates and cause of death (future linkage in development)
Community Health Center data
THRIVE: homelessness, food and housing insecurity, inability to afford medications, lack of transportation, educational aspirations, utility bill concerns, employment, caregiving needs

>130 Publications from Boston Birth Cohort by Organs/Systems

Prenatal, Peri-natal and Birth Outcomes
Allergy, Asthma, Upper and Lower Airway Conditions
Cardio-Metabolic Outcomes
Neurodevelopmental Outcomes
Opioids Epidemic: Risk factors and Consequences
Puberty and Antecedents
COVID-19 on Maternal and Child Health

**Pre, Peri-natal and Birth Outcomes**
1. Wang X, Zuckerman B, Kaufman G, Wise P, Hill M, Niu T, Ryan L, Wu D, Xu X. Molecular epidemiology of preterm delivery: methodology and challenges. *Paediatric and Perinatal Epidemiology* 2001; 15(suppl 2): 63-77.
2. Wang X, Zuckerman B, Pearson C, Kaufman G, Chen C, Wang G, Niu T, Wise P, Bauchner H, Xu X. Maternal cigarette smoking, metabolic gene polymorphism, and infant birth weight. *Journal of American Medical Association* 2002; 287:195-202. (See editorial comments).
3. Hao K, Xu X, Laird N, Wang X, Xu X. Power estimation of multiple SNP association test of case-control study and application. *Genetic Epidemiology* 2004; 26:22-30.
4. Hao K, Wang X, Niu T, Xu Xin, Li A, Chang W, Wang L, Li G, Laird N, Xu X. A candidate gene association study on preterm delivery: application of high-throughput genotyping technology and advanced statistical methods. *Human Molecular Genetics* 2004; 13:683-691.
5. Hao K, Wang X. Incorporating individual error rate into association test of unmatched case-control design. *Human Heredity* 2004; 58:154-63.
6. Wang L, Wang X, Laird N, Zuckerman B, Stubblefield P, Xu Xin. Polymorphism in maternal *LRP8* gene is associated with fetal growth. *American Journal of Human Genetics* 2006; 78(5):770-7.
7. Wang L, Feng Y, Zhang Y, Zhou H, Jiang J, Niu T, Wei LJ, Xu Xin, Xu X, Wang X. Prolylcarboxypeptidase gene, chronic hypertension, and risk of preeclampsia. *American Journal of Obstetrics and Gynecology* 2006; 195:162-71.
8. Gupta M, Mestan KK, Martin CR, Pearson C, Ortiz K, Fu L, Stubblefield P, Cerda S, Kasznica JM, Wang X. Impact of clinical and histologic correlates of maternal and fetal inflammatory response on gestational age in preterm births. *Journal of Maternal, Fetal, and Neonatal Medicine* 2007; 20(1):39-46.
9. Tsai H, Liu X, Mestan K, Yu Y, Zhang S, Fang Y, Pearson C, Ortiz K, Zuckerman B, Bauchner H, Cerda S, Stubblefield P, Xu X, Wang X. Maternal cigarette smoking, metabolic gene polymorphisms, and preterm delivery: new insights on GxE interactions and pathogenic pathways. *Human Genetics* 2008; 123:359-69.
10. Matoba N1, Yu Y, Mestan K, Pearson C, Ortiz K, Porta N, Thorsen P, Skogstrand K, Hougaard DM, Zuckerman B, Wang X. Differential patterns of 27 cord blood immune biomarkers across gestational age. *Pediatrics.* 2009 May;123(5):1320-8. doi: 10.1542/peds.2008-1222.
11. Mestan K, Yu Y, Thorsen P, Skogstrand K, Matoba N, Liu X, Kumar R, Hougaard DM, Gupta M, Pearson C, Ortiz K, Bauchner H, Wang X. Cord Blood Biomarkers of the Fetal Inflammatory Response. *Journal of Maternal-Fetal & Neonatal Medicine* 2009; 22(5):379-87.
12. Yu Y, Tsai H, Liu X, Mestan K, Zhang S, Pearson C, Ortiz K, Xu X, Zuckerman B, Wang X. The joint association between F5 gene polymorphisms and maternal smoking during pregnancy on preterm

delivery. *Human Genetics* 2009;124(6):659-68.

13. Tsai HJ, Yu Y, Zhang S, Pearson C, Ortiz K, Xu X, Bauchner H, Zuckerman B, Wang X. Association of genetic ancestry with preterm delivery and related traits among African American mothers. *American Journal of Obstetrics and Gynecology* 2009 July; 201(1):94.E1-10. Comments and reply: 2010; 202(6):e12-3.

14. Mestan K, Ouyang F, Matoba N, Pearson C, Ortiz K, Wang X. Maternal obesity, diabetes mellitus, and cord blood biomarkers in large-for-gestational age infants. *Journal of Pediatric Biochemistry* 2010; 1(3): 217–224.

15. Matoba N, Ouyang F, Mestan K, Porta, N, Pearson C, Ortiz K, Bauchner H, Zuckerman B, Wang X. Cord blood immune biomarkers in small for gestational age births. *Journal of Developmental Origins of Health and Disease* 2011; 2(2):89-98.

16. Kumar P, Venners S, Fu L, Pearson C, Ortiz K, Wang X. Association of antenatal steroid use with cord blood immune biomarkers in preterm births. *Early Human Development* 2011; 87(8):559-64.

17. Tsai H, Hong X, Chen J, Liu X, Pearson C, Ortiz K, Fu L, Hirsch E, Heffner L, Weeks DE, Zuckerman B, Wang X. Role of African Ancestry and Gene-Environment Interactions in Predicting Preterm Birth. *Obstetrics & Gynecology* 2011; 118(5):1081-9.

18. Liu X, Wang G, Hong X, Tsai H, Liu R, Zhang S, Wang H, Pearson C, Ortiz K, Wang D, Hirsch E, Zuckerman B, Wang X. Associations between Gene Polymorphisms in Fatty Acid Metabolism Pathway and Preterm Delivery in a U.S. Urban Black Population. *Human Genetics* 2012; 131(3):341-51.

19. Wang D, Liu X, Zhou Y, Xie H, Hong X, Tsai HJ, Wang G, Liu R, Wang X. Individual variation and longitudinal pattern of genome-wide DNA methylation from birth to the first two years of life. *Epigenetics* 2012; 7(6):594-605.

20. Ouyang F, Parker M, Cerda S, Pearson C, Fu L, Gillman MW, Zuckerman B, Wang X. Placental weight mediates the effects of prenatal factors on fetal growth: the extent differs by preterm status. *Obesity (Silver Spring)*. 2013; 21(3):609-20.

21. Yu Y, Zhang S, Wang G, Hong X, Mallow EB, Walker SO, Pearson C, Heffner L, Zuckerman B, Wang X. The Combined association of psychosocial stress and chronic hypertension with preeclampsia. *American Journal of Obstetrics and Gynecology* 2013; 209(5):438.e1-438.e12*.*

22. Parker MG, Ouyang F, Pearson C, Gillman MW, Belfort MB, Hong X, Wang G, Heffner L, Zuckerman B, Wang X. Prepregnancy BMI and risk of preterm birth: Association heterogeneity by preterm subgroups. *BMC Pregnancy and Childbirth* 2014; 14:153.

23. Chen Z, Myers R, Wei T, Bind E, Kassim P, Wang G, Ji Y, Hong X, Caruso D, Bartell T, Gong Y, Strickland P, Navas-Acien A, Guallar E, Wang X. Placental transfer and concentrations of cadmium, mercury, lead, and selenium in mothers, newborns, and young children. *J Expo Sci Environ Epidemiol.* 2014 Sep-Oct;24(5):537-44. doi: 10.1038/jes.2014.26. Epub 2014 Apr 23. PMID: 24756102; PMCID: PMC4329243.

24. Tyna Dao, Xiumei Hong, Xiaobin Wang and Wan-yee Tang. Aberrant 5'-CpG methylation of cord blood TNFα associated with maternal exposure to polybrominated diphenyl ethers. *Plos One* 2015 Sep 25;10(9):e0138815. doi: 10.1371/journal.pone.0138815. eCollection 2015.

25. Nachman RM, Mao G, Zhang X, Hong X, Chen Z, He H, Wang G, Caruso D, Pearson C, Biswal S, Zuckerman B, Wills-Karp M, Wang X. Intrauterine Inflammation and maternal exposure to ambient $pm_{2.5}$

during preconception and specific periods of pregnancy: The Boston Birth Cohort. *Environmental Health Perspectives* 2016 Oct;124(10):1608-1615. https://doi.org/10.1289/EHP243. PMCID: PMC5047781

26. Zhang B, Hong X, Ji H, Tang WY, Kimmel M, Ji Y, Wang G, Pearson C, Zuckerman B, Surkan PJ, Wang X. Maternal smoking during pregnancy and cord blood DNA methylation: new insight on sex differences and effect modification by maternal folate. *Epigenetics* 2018 Jun 27. doi: 10.1080/15592294.2018.1475978. PMID: 29945474

27. Hong X, Hao K, Ji H, Peng S, Sherwood B, Narzo AD, Tsai HJ, Liu X, Burd I, Wang G, Ji Y, Caruso D, Mao G, Bartell TR, Zhang Z, Pearson C, Heffner L, Sandra Cerda[9], Terri H. Beaty[10], M Daniele Fallin[11], Lee-Parritz A, Zuckerman B, Weeks DE, Wang X. Genome-wide association study of preterm birth identified a significant gene-maternal pre-pregnancy BMI interaction in the Boston Birth Cohort. *Nature Communication* 2017 Jun 9;8:15608. doi: 10.1038/ncomms15608. PMID: 28598419

28. Hong X, Sherwood B, Ladd-Acosta C, Peng S, Ji H, Hao K, Burd I, Bartell TR, Wang G, Tsai HJ, Liu X, Ji Y, Wahl A, Caruso D, Lee-Parritz A, Zuckerman B, Wang X. Genome-wide DNA methylation associations with spontaneous preterm birth in US blacks: findings in maternal and cord blood samples. *Epigenetics* 2017. DOI: 10.1080/15592294.2017.1287654 PMID 28165855

29. Tsai HJ, Surkan PJ, Yu, SM, Caruso D, Hong X, Bartell TR, Wahl A, Sampankanpanich C, Reily A, Zuckerman BS, Wang X. Differential effects of stress and African ancestry on preterm birth and related traits among US born and immigrant Black mothers. *Medicine* (Baltimore). 2017 Feb;96(5):e5899. doi: 10.1097/MD.0000000000005899. PMID: 28151865 [PubMed - in process]

30. Bustamante-helfrich B, Chilukuri N, He H, Cerda SR, Hong X, Wang G, Pearson C, Burd I, Wang X. Maternal vascular malperfusion of the placental bed associated with hypertensive disorders in the boston birth cohort. *Placenta* 2017 Apr;52:106-113 doi: 10.1016/j.placenta.2017.02.016 PMID: 28454692

31. Surkan PJ, Dong L, Ji Y, Hong X, Ji H, Wang X. Paternal involvement and support and risk of preterm birth: Findings from the Boston Birth Cohort. *J Psychosom Obs Gyn,* 2017 Nov 16:1-9. doi: 10.1080/0167482X.2017.1398725. PMID:29144191

32. Rappoport N, Toung J, Hadley D, Wong RJ, Fujioka K, Reuter J, Abbott CW, Oh S, Hu D, Eng C, Huntsman S, Bodian DL, Niederhuber JE, Hong X, Zhang G, Sikora-Wohfeld W, Gignoux CR, Wang H, Oehlert J, Jelliffe-Pawlowski LL, Gould JB, Darmstadt GL, Wang X, Bustamante CD, Snyder MP, Ziv E, Patsopoulos NA, Muglia LJ, Burchard E, Shaw GM, O'Brodovich HM, Stevenson DK, Butte AJ, Sirota M. A genome-wide association study identifies only two ancestry specific variants associated with spontaneous preterm birth. *Scientific Reports, 2018* Jan 9;8(1):226. *doi:10.1038.s41598-017-18246-5.* PMID: 29317701

33. Cheng TL, Mistry KB, Wang G, Zuckerman B, Wang X. Folate nutrition status in a sample of U.S. urban low-income mothers: A public health perspective. *Am J Public Health.* 2018 Apr 19:e1-e9. doi: 10.2105/AJPH.2018.304355. PMID: 29672150

34. Zheng Z, Bennett WL, Mueller NT, Appel LJ, Wang X. Gestational weight gain and pregnancy complications in a high-risk racially and ethnically diverse population. *J Women Health* 2018 Jun 19. doi: 10.1089/jwh.2017.6574. PMID: 29920144 [PubMed - as supplied by publisher]

35. Duis J, Cox OH, Ji Y, Seifuddin F, Lee RS, Wang X. Effect of Genotype and Maternal Affective Disorder on Intronic Methylation of FK506 Binding Protein 5 in Cord Blood DNA. *Front Genet*. 2018 Dec 17;9:648. doi: 10.3389/fgene.2018.00648. eCollection 2018. PMID: 30619472 [PubMed]

36. Surkan PJ, Hong X, Zhang B, Nawa N, Ji H, Tang WY, Ji Y, Kimmel MC, Wang G, Pearson C, Wang X.

	Can social support during pregnancy affect maternal DNA methylation? Findings from a cohort of African Americans. *Pediatr Res*. 2019 Jul 26. doi: 10.1038/s41390-019-0512-7. [Epub ahead of print] PMID: 31349361 [PubMed - as supplied by publisher]
37.	Li J, Hong X, Mesiano S, Muglia LJ, Wang X, Snyder M, Stevenson DK, Shaw GM. Natural Selection Has Differentiated the Progesterone Receptor among Human Populations. *Am J Hum Genet.* 2018 Jul 5;103(1):45-57. doi: 10.1016/j.ajhg.2018.05.009. Epub 2018 Jun 21. PMID: 29937092; PMCID: PMC6035283.
38.	Stevenson DK, Wong RJ, Aghaeepour N, Angst MS, Darmstadt GL, DiGiulio DB, Druzin ML, Gaudilliere B, Gibbs RS, B Gould J, Katz M, Li J, Moufarrej MN, Quaintance CC, Quake SR, Relman DA, Shaw GM, Snyder MP, Wang X, Wise PH. Understanding health disparities. *J Perinatol.* 2018 Dec 18. doi: 10.1038/s41372-018-0298-1.PMID: 30560947
39.	Olapeju B, Saifuddin A, Wang G, Ji Y, Hong X, Raghavan R, Summers A, Keiser A, Ji H, Zuckerman B, Heffner LJ, Cheng TL, Hao L, Surkan PJ, Wang X. Maternal folate status and preterm birth in a high-risk US population. *Public Health Nutrition* 2018 Nov 29:1-11. doi: 10.1017/S1368980018003221. PMID: 30486913
40.	Liu T, Zhang M, Guallar E, Wang G, Hong X, Wang X, Mueller NT. Trace Minerals, Heavy Metals and Preeclampsia: Findings from the Boston Birth Cohort. *JAHA* Aug 20, 2019 https://doi.org/10.1161/JAHA.119.012436Journal of the American Heart Association. 2019;8:e012436
41.	Zhang Z, Cheng H, Hong X, Di Narzo AF, Franzen O, Peng S, Ruusalepp A, Kovacic JC, Bjorkegren JLM, Wang X, Hao K. EnsembleCNV: an ensemble machine learning algorithm to identify and genotype copy number variation using SNP array data. *Nucleic Acids Res*. 2019 Apr 23;47(7):e39. doi: 10.1093/nar/gkz068. PMID: 30722045 [PubMed - indexed for MEDLINE] Free PMC Article
42.	Hong X, Surkan P, Zhang B, Keiser A, Ji Y, Ji H, Burd I, Bustamante-Helfrich B, Ogunwole S, Tang WY, Liu L, Pearson C, Cerda S, Zuckerman B, Hao L, Wang X. Genome-wide association study identifies a novel maternal gene × stress interaction associated with spontaneous preterm birth. *Pediatric Research* 2020, https://doi.org/10.1038/s41390-020-1093-1.
43.	Hong X, Bartell TR, Wang X. Gaining a deeper understanding of social determinants of preterm birth by integrating multi-omics data. *Pediatr Res*. 2021 Jan;89(2):336-343. doi: 10.1038/s41390-020-01266-9. Epub 2020 Nov 13. PMID: 33188285; PMCID: PMC7898277.
44.	Hong X, Zhang B, Liang L, Zhang Y, Ji Y, Wang G, Ji H, Clish C, Burd I, Pearson C, Zuckerman B, Hu F, Wang X. Postpartum plasma metabolomic profile among women with preeclampsia and preterm delivery: implications for long-term health. *BMC Medicine*, 2020 Oct 13;18(1):277. doi: 10.1186/s12916-020-01741-4. PubMed PMID: 33046083; PubMed Central PMCID: PMC7552364.
45.	Olapeju B, Saifuddin A, Hong X, Wang G, Summers A, Cheng TL, Irina Burd, Wang X. Maternal Preeclampsia Status during Pregnancy and Postpartum Plasma B-vitamin and Homocysteine Profiles in a High-Risk Multiethnic US Population. *J Women's Health* (Larchmt). 2020 Nov 16;. doi: 10.1089/jwh.2020.8420. [Epub ahead of print] PubMed PMID: 33252313.
46.	Boakye E, Sharma G, Ogunwole SM, Zakaria S, Vaught AJ, Kwapong YA, Hong X, Ji Y, Mehta L, Creanga AA, Blaha MJ, Blumenthal RS, Nasir K, Wang X. Relationship of Preeclampsia With Maternal Place of Birth and Duration of Residence Among Non-Hispanic Black Women in the United States. *Circ Cardiovasc Qual Outcomes.* 2021 Feb;14(2):e007546. doi: 10.1161/CIRCOUTCOMES.120.007546. Epub 2021 Feb 10. PMID: 33563008; PMCID: PMC7887058.
47.	Ogunwole SM, Mwinnyaa G, Wang X, Hong X, Henderson J, Bennett WL. Preeclampsia Across Pregnancies and Associated Risk Factors: Findings From a High-Risk US Birth Cohort. *J Am Heart*

Assoc. 2021 Sep 7;10(17):e019612. doi: 10.1161/JAHA.120.019612. Epub 2021 Aug 16. PMID: 34398644.
48. Xu, R., Hong X, Zhang B, Huang W, Hou W, Wang G, Wang X, Igusa T, Liang L, Ji H. DNA methylation mediates the effect of maternal smoking on offspring birthweight: a birth cohort study of multi-ethnic US mother-newborn pairs. *Clin Epigenetics.* 2021 Mar 4;13(1):47. doi: 10.1186/s13148-021-01032-6. PMID: 33663600; PMCID: PMC7931602.
49. Makker, K., Ji, Y., Hong, X. et al. Antenatal and neonatal factors contributing to extra uterine growth failure (EUGR) among preterm infants in Boston Birth Cohort (BBC). *J Perinatol*. 2021; https://doi.org/10.1038/s41372-021-00948-4.
50. Rhee DK, Ji Y, Hong X, Pearson C, Wang X, Caulfield LE. Mediterranean-Style Diet and Birth Outcomes in an Urban, Multiethnic, and Low-Income US Population. *Nutrients*. 2021 Apr 3;13(4):1188. doi: 10.3390/nu13041188. PMID: 33916686; PMCID: PMC8066173.
51. Wang X. Healthy diet during pregnancy-navigating the double-edged sword. *Am J Clin Nutr.* 2021 May 26:nqab168. doi: 10.1093/ajcn/nqab168. Epub ahead of print. PMID: 34038942.
52. Olapeju B, Hong X, Wang G, Summers A, Burd I, Cheng TL, Wang X. Birth outcomes across the spectrum of maternal age: Dissecting aging effect versus confounding by social and medical determinants. *BMC Pregnancy Childbirth* 21, 594 (2021). https://doi.org/10.1186/s12884-021-04077-w
53. Park S, Ji Y, Hong X, Zuckerman B, Wang X, Surkan PJ. Effects of Stress and Nativity on Maternal Antenatal Substance Use and Postnatal Mental Disorders. J Womens Health (Larchmt). 2021 Dec 17. doi: 10.1089/jwh.2021.0016. Epub ahead of print. PMID: 34935494.
54. Boakye E, Kwapong YA, Obisesan F, Ogunwole SM, Hays AG, Nasir K, Blumenthal RS, Douglas PS, Blaha MJ, Creanga AA, Wang X, Sharma G. Nativity-related Disparities in Preeclampsia and Cardiovascular Disease Risk Profile among Racially Diverse Women in the United States. *JAMA Network Open.* 2021 Dec 1;4(12):e2139564. doi: 10.1001/jamanetworkopen.2021.39564. PMID: 34928357.
55. Zhang M, Buckley JP, Liang L, Hong X, Wang G, Guallar E, Wang MC, Wills-Karp M, Wang X, Mueller N. A metabolome-wide association study of *in utero* metal and trace element exposures with cord blood metabolome profile: findings from the Boston Birth Cohort. *Environmental International 2022, Volume158. https://doi.org/10.1016/j.envint.2021.106976.* PMCID in progress
56. Anum S. Minhas, Ellen Boakye, Olufunmilayo H. Obisesan, Yaa A. Kwapong, Sammy Zakaria, Andreea A. Creanga, Arthur J. Vaught, Laxmi S. Mehta, Melinda B. Davis, Natalie A. Bello, Miguel Cainzos-Achirica, Khurram Nasir, Michael J. Blaha, Roger S. Blumenthal, Pamela S. Douglas, Xiaobin Wang, Garima Sharma. The Association of Preterm Birth With Maternal Nativity and Length of Residence Among Non-Hispanic Black Women. CJC Open,2021, ISSN 2589-790X,
57. Minhas AS, Hong X, Wang G, Rhee DK, Liu T, Zhang M, Michos ED, Wang X, Mueller NT. Mediterranean-Style Diet and Risk of Preeclampsia by Race in the Boston Birth Cohort. *JAHA* (in press)
58. Yaa A. Kwapong, Ellen Boakye, Lochan M Shah, S. Michelle Ogunwole, Allison G. Hays, Michael J Blaha, Khurram Nasir, Xiaobin Wang, Garima Sharma. Cardiovascular Risk Profile and Preterm Birth by Maternal Nativity in Racially Diverse, Urban, Low-Income Women in the United States. AJPM (in press)

59. Lochan M Shah, Yaa A. Kwapong, Ellen Boakye, S. Michelle Ogunwole, Wendy L. Bennett, Roger S Blumenthal, Allison G. Hays, Michael J Blaha, Khurram Nasir, Sammy Zakaria, Guoying Wang, Xiaobin Wang, Garima Sharma. Racial disparities in Gestational Diabetes by Maternal Nativity and Length of US Residence in an Urban Low-income Population in the United States. CJC Open (in press).

Allergy, Asthma, Upper and Lower Airway Conditions

60. Kumar R, Yu Y, Story RF, Pongracic JA, Gupta R, Pearson C, Ortiz K, Bauchner HC, Wang X. Prematurity, chorioamnionitis and the development of recurrent wheezing: A prospective birth cohort study. *Journal of Allergy & Clinical Immunology* 2008; 121(4):878-84.

61. Kumar R, Ouyang F, Story RE, Pongracic JA, Hong X, Wang G, Pearson C, Ortiz K, Bauchner H, Wang X. Gestational diabetes, atopic dermatitis and allergen sensitization in early childhood. *Journal of Allergy & Clinical Immunology* 2009; 124(5):1031-1038.

62. Kumar R, Story RE, Pongracic JA, Hong X, Arguelles L, Wang G, Kuptsova-Clarkson N, Pearson C, Ortiz K, Bonzagni A, Apollon S, Fu L, Bauchner H, Wang X. Maternal pre-pregnancy obesity and recurrent wheezing in early childhood. *Pediatric Allergy, Immunology, and Pulmonology* 2010; 23(3):183-190.

63. Hong X, Tsai HJ, Liu X, Arguelles L, Kumar R, Wang G, Kuptsova-Clarkson N, Pearson C, Ortiz K, Bonzagni A, Apollon S, Fu L, Pongracic JA, Schleimer R, Holt PG, Bauchner H, Wang X. Does genetic regulation of IgE begin in-utero? Evidence from TH1/TH2 gene polymorphisms and cord blood total IgE. *Journal of Allergy & Clinical Immunology* 2010; 126(5):1059-1067.

64. Hong X, Wang G, Liu X, Kumar R, Tsai H, Arguelles L, Hao K, Pearson C, Ortiz K, Bonzagni A, Apollon S, Fu L, Caruso D, Pongracic J, Schleimer R, Holt P, Bauchner H, Wang X. Gene polymorphisms, breastfeeding and development of food sensitization in early childhood. *Journal of Allergy & Clinical Immunology* 2011; 128(2):374-381.e2.

65. Kumar R, Tsai HJ, Hong X, Liu X, Wang G, Pearson C, Ortiz K, Fu L, Pongracic J, Bauchner H, Wang X. Race, ancestry and development of food allergen sensitization in early childhood. *Pediatrics* 2011; 128(4):e821-9.

66. Liu X, Wang GY, Hong XM, Wang D, Tsai HJ, Zhang SC, Arguelles L, Kumar R, Wang HJ, Liu R, Zhou Y, Pearson C, Ortiz K, Schleimer R, Holt R, Pongracic J, Price HE, Langman C, Wang X. Gene-Vitamin D interactions on food sensitization: A prospective birth cohort study. *Allergy* 2011; 66(11):1442-8.

67. Kumar R, Tsai H, Hong X, Pearson C, Fu M, Pongracic J, Bauchner H, Wang X. African ancestry, early life exposures, and respiratory morbidity in early childhood. *Clinical and Experimental Allergy* 2012; 42(2):265-74.

68. Robison RG, Kumar R, Arguelles LM, Hong X, Wang G, Apollon S, Bonzagni A, Ortiz K, Pearson C, Pongracic JA, Bauchner H, Wang X. Maternal smoking during pregnancy, prematurity and recurrent wheezing in early childhood. *Pediatric Pulmonology* 2012; 47(7):666-673.

69. Liu X, Arguelles L, Zhou Y, Wang G, Chen Q, Tsai H, Hong X, Liu R, Price H, Pearson C, Apollon S, Cruz N, Schleimer R, Langman C, Pongracic J, Wang X. The longitudinal trajectory of vitamin D status from birth to early childhood on the development of food sensitization. *Pediatric Research* 2013; 74(3):321-6.

70. Tripathi P, Hong X, Caruso D, Gao P, Wang X. Genetic determinants in the development of sensitization to environmental allergens in early childhood. *Immunity, Inflammation and Disease* 2014; 2(3):193-204.

71. Hong X, Hao K, Ladd-Acosta C, Hansen KD, Tsai HJ, Liu X, Xu X, Thornton TA, Caruso D, Keet CA, Sun Y, Wang G, et al. Genome-wide Association Study identifies peanut allergy-specific loci and evidence of epigenetic mediation in U.S. children. *Nat Commun.* 2015 Feb 24;6: 6304. doi: 10.1038/ncomms7304.
*NIAID news release and Hopkins BSPH news release.

72. He H, Butz A, Keet CA, Minkovitz CS, Hong X, Caruso DM, Pearson C, Cohen RT, Wills-Karp M, Zuckerman B, Hughes ME, Wang X. Preterm birth with childhood asthma: the role of degree of prematurity and asthma definitions. *American Journal of Respiratory and Critical Care Medicine.* 2015 Aug 15;192(4):520-3.

73. Hong X, Ladd-Acosta C, Hao K, Sherwood B, Ji H, Keet CA, Kumar R, Caruso D, Liu X, Wang G, Chen Z, Ji Y, Mao G, Walker SO, Bartell TR, Ji Z, Sun Y, Tsai HJ, Pongracic JA, Weeks DE, Wang X. Epigenome-wide association study links site-specific DNA methylation changes with cow's milk allergy. *Journal of Allergy & Clinical Immunology,* 2016 Sep;138(3):908-911.e9.

74. Tsai HJ, Wang G, Hong X**,** Yao TC, Y Ji Y, Radovick S, Cheng TL, Wang X. Early Life Weight Gain and Development of Childhood Asthma in a Prospective Birth Cohort. *Annals of the American Thoracic Society* 2018; doi.org/10.1513/AnnalsATS.201712-921OC. PubMed: 29979628

75. Hong X, Liang L, Sun Q, Keet C, Tsai HJ, Ji Y, Wang G, Ji H, Clish C, Pearson C, Wang Y, Wood R, Hu FB, Wang X. Maternal Triacylglycerol Signature and Offspring Risk of Food Allergy: Effect modification by timing of solid food introduction. *J Allergy Clin Immunol.* 2019 Apr 17. pii: S0091-6749(19)30522-6. doi: 10.1016/j.jaci.2019.03.033. [Epub ahead of print] PMID: 31004614 *selected as *JACI* Editor's Choice

76. Chen T, Hughes ME, Wang H, Wang G, Hong X, Liu Li, Ji Y, Pearson C, Li S, Hao L, Wang X. Prenatal, Perinatal, Early Childhood Factors Associated with Childhood Obstructive Sleep Apnea: Role of Cord and Early Childhood Plasma Leptin. *J Pediatr.* 2019 Jun 25. pii: S0022-3476(19)30670-5. doi: 10.1016/j.jpeds.2019.05.053. PMID: 31253409 [PubMed - as supplied by publisher]

77. McGowan EC, Hong X, Selhub J, Paul L, Wood RA, Matsui EC, Keet CA, Wang X. Association Between Folate Metabolites and the Development of Food Allergy in Children. *J Allergy Clin Immunol Pract.* 2019 Jun 25. pii: S2213-2198(19)30569-0

78. Gutierrez, M.J.; Nino, G.; Hong, X.; Wang, X. Epigenetic Dynamics of the Infant Immune System Reveals a Tumor Necrosis Factor Superfamily Signature in Early Human Life. Epigenomes 2020, 4, 12.

79. Gutierrez, M.J.; Nino, G.; Hong, X.; Wang, X. Epigenomics and early life human humoral immunity: novel paradigms and research opportunities. *Frontiers in Immunology, section B Cell Biology*. Mini Review. September 2020 https://doi.org/10.3389/fimmu.2020.01766

80. Gutierrez, M.J.; Nino, G.; Hong, X.; Wang, X. Maternal pre-pregnancy weight and early life lower respiratory tract infections in a low-income urban minority birth cohort. *Scientific Reports* 11, 9790 (2021). https://doi.org/10.1038/s41598-021-88360-y

81. Gutierrez, M.J.; Nino, Landeo-Gutierrez, J; Weiss, M; Preciado, DG; Hong, X.; Wang, X. Lower respiratory tract infections in early life are associated with OSA diagnosis during childhood in a large birth cohort. *Sleep*, 2021; zsab198, https://doi.org/10.1093/sleep/zsab198

82. Wang Y, Hong X, Yao TC, Tsai HJ, Wang X. Interaction of maternal asthma history and plasma folate levels on child asthma risk in the Boston Birth Cohort. *Pediatr Pulmonol.* 2021 Oct 4. doi: 10.1002/ppul.25680. PMID: 34607393.

83. Hong X, Liang L, Ji H, Larmin B, Guerrerio P, Wang G, Clish C, Pearson C, Hu FB, Wang X. Fetal lipidome and incident risk of food allergy: A prospective birth cohort study. *Pediatric Allergy and Immunology* First published: 17 December 2021 https://doi.org/10.1111/pai.13722

Cardio-Metabolic Outcomes

84. Liu X, Chen Q, Tsai H, Wang G, Hong X, Zhou Y, Zhang C, Liu C, Liu R, Wang H, Zhang S, Yu Y, Mestan K, Pearson C, Otlans P, Zuckerman B, Wang X. Maternal preconception body mass index and offspring cord blood dna methylation: exploration of early life origins of disease. *Environmental and Molecular Mutagenesis* 2014; 55(3):223-30.

85. Wang G, Divall S, Radovick S, Paige D, Ning Y, Chen Z, Ji Y, Hong X, Walker S, Caruso D, Pearson C, Wang MC, Zuckerman B, Cheng TL, Wang X. Preterm birth and random plasma insulin levels at birth and in early childhood. *JAMA* 2014; 311(6):587-596 (see editorial comments).
*Hopkins BSPH news release.

86. Wang G, Johnson S, Gong Y, Thornton R, Divall S, Radovick S, Paige D, Hong X, Pearson C, Wang MC, Cheng TL, Wang X. Weight Gain in the first 4 months of life and Development of Childhood Obesity among Term, Preterm, and Fetal Growth Restricted Babies in a High Risk U.S. Birth Cohort. *Science report* 2016 Jul 15;6:29867. doi: 10.1038/srep29867.

87. Wang G, Hu FB, Mistry KB, Zhang C, Ren F, Huo Y, Paige D, Bartell T, Hong X, Caruso D, Ji Z, Chen Z, Ji Y, Pearson C, Ji H, Zuckerman B, Cheng TL, Wang X. Maternal prepregnancy BMI, plasma folate level and child metabolic risk. *JAMA pediatrics* 2016 Aug 1;170(8):e160845.
*****NICHD news release and Hopkins BSPH news release.

88. Mao G, Nachman RM, Sun Q, Zhang X, Koehler K, Chen Z, Hong X, Wang G, Caruso D, Zong G, Pearson C, Ji H, Biswal S, Zuckerman B, Wills-Karp M, Wang X. Individual and joint effects of early-life ambient $pm_{2.5}$ exposure and maternal pre-pregnancy obesity on childhood overweight or obesity. Environ Health Perspect. Environ Health Perspect. 2017 Jun 14;125(6):067005. doi: 10.1289/EHP261. PMID: 28669938. PMCID: PMC5743454

89. Mueller N, Mao G, Bennet W, Hourigan S, Dominguez-Bello MG, Appel L, and Wang X. Does vaginal delivery mitigate or strengthen the intergenerational association of overweight and obesity? Findings from the Boston Birth Cohort. *International Journal of Obesity.* 2016 Nov 30. doi: 10.1038/ijo.2016.219. [Epub ahead of print] *****2017 most cited papers of IJO

90. Wang H, Mueller NT, Li J, Sun N, Huo Y, Ren F, Wang X. Joint association of maternal folate level and cardiometabolic risk factors on child risk of elevated blood pressure. *American Journal of Hypertension* 2017 Mar 6. doi: 10.1093/ajh/hpx003. PMID: 28338750

91. Wang H, Xu B, Xu R, Walker S, Wang G. Joint effect of maternal plasma homocysteine and prepregnancy obesity on child blood pressure. *International Journal of Obesity* 2017 May 3. doi: 10.1038/ijo.2017.109. [Epub ahead of print] PMID: 28454692 PMCID: PMC5412713

92. Wang H, Mueller N, Wang G, Hong X, Chen T, Ji Y, Pearson C, Appel LJ, Wang X. Does longer sleep in early childhood mitigate higher blood pressure associated with small for gestational age? *Science Reports* 2018 Jun 25;8(1):9632. doi: 10.1038/s41598-018-27815-1. PMID: 29941998 [PubMed - in process]

93. Zhang M, Mueller NT, Wang H, Hong X, Appel LJ, Wang X. Maternal exposure to ambient $pm_{2.5}$ during pregnancy and the risk for high blood pressure in childhood: findings from the Boston Birth Cohort. *Hypertension* 2018 Jul;72(1):194-201. doi: 10.1161/HYPERTENSIONAHA.117.10944. Epub 2018 May 14. PMCID: PMC6002908 See editorial comments and press release by the journal and AHA news. This paper received broad media attention.

94. Wang G, Liu X, Bartell TR, Pearson C, Cheng TL, Wang X. Vitamin D trajectories from birth to early childhood and elevated systolic blood pressure during childhood and adolescence. *Hypertension*. 2019 Jul 1:HYPERTENSIONAHA11913120. doi: 10.1161/HYPERTENSIONAHA.119.13120. [Epub ahead of print] PMID: 31256718 [PubMed - as supplied by publisher]

95. Wang G, Sun Q, Liang L, Clash C, Zhang C, Hong X, Ji Y, Radovick S, Pearson C, Bartell TR, Zuckerman B, Cheng TL, Hu FB, Wang X. Inter-generational link of obesity in term and preterm births: role of maternal plasma acylcarnitines. *Int J Obes* (Lond). 2019 Jul 22. doi: 10.1038/s41366-019-0417-x. [Epub ahead of print] PMID: 31332276 [PubMed - as supplied by publisher] PMCID: PMC6900290.

96. Wang G, DiBari J, Bind E, Steffens AM, Mukherjee J, Azuine RE, Singh GK, Hong X, Ji Y, Ji H, Pearson C, Zuckerman BS, Cheng TL, Wang X. Association Between Maternal Exposure to Lead, Maternal Folate Status, and Intergenerational Risk of Childhood Overweight and Obesity. *JAMA Netw Open.* 2019 Oct 2;2(10):e1912343. doi: 10.1001/jamanetworkopen.2019.12343. PMID: 31577354; PMCID: PMC6777254. (see editorial comments and NIH press release)

97. Wang G, DiBari J, Bind E, Steffens AW, Mukherjee J, Bartell TR, Bellinger DC, Hong X, Ji Y, Wang MC, Wills-Karp M, Cheng TL, Wang X. In utero exposure to mercury and childhood overweight or obesity: counteracting effect of maternal folate status. *BMC Med* 17, 216 (2019) doi:10.1186/s12916-019-1442-2. Published on November 2019. PMCID*:* PMC6882077.

98. Zhang M, Michos ED, Wang G, Wang X, Mueller NT. Associations of Cord Blood Vitamin D and Preeclampsia With Offspring Blood Pressure in Childhood and Adolescence. *JAMA Netw Open.* 2020 Oct 1;3(10):e2019046. doi: 10.1001/jamanetworkopen.2020.19046. PMID: 33017029; PMCID: PMC7536588.

99. Wang G, Tang WY, Wills-Karp M, Ji H, Bartell TR, Ji Y, Hong X, Pearson C, Cheng TL, Wang X. A Nonlinear Relation Between Maternal Red Blood Cell Manganese Concentrations and Child Blood Pressure at Age 6-12 y: A Prospective Birth Cohort Study. J Nutr. 2021 Mar 11;151(3):570-578. doi: 10.1093/jn/nxaa368. PMID: 33438012; PMCID: PMC7948198.

100. Long J, Zhang M, Wang G, Hong X, Ji Y, Bustamante-Helfrich B, Wang X, Mueller NT. Association of Placental Pathology with Childhood Blood Pressure Among Children Born Preterm. *American Journal of Hypertension*, Volume 34, Issue 11, November 2021, Pages 1154–1162, https://doi.org/10.1093/ajh/hpab097

101. Cao T, Zhao J, Hong X, Wang G, Hu F, Wang X, Liang L. Associations of cord blood plasma lipid metabolites with longitudinal BMI trajectory from birth to adolescence in US multi-ethnic birth cohort. *Metabolites* 2021, 11, 739. https://doi.org/10.3390/metabo11110739

102. Zhang M, Liu T, Wang G, Buckley JP, Guallar E, Hong X, Wang MC, Wills-Karp M, Wang X, Mueller NT. In-Utero Exposure to Heavy Metals and Trace Elements and Childhood Blood Pressure in a U.S. Urban, Low-Income, Minority Birth Cohort. *Environ Health Perspect.* 2021 Jun;129(6):67005. doi: 10.1289/EHP8325. Epub 2021 Jun 23. PMID: 34160246; PMCID: PMC8221032.

103. Minhas AS, Boakye E, Obisesan OH, Kwapong YA, Zakaria S, Creanga AA, Vaught AJ, Mehta LS, Davis MB, Bello NA, Cainzos-Achirica M, Nasir K, Blaha MJ, Blumenthal RS, Douglas PS, Wang X, Sharma G. The Association of Preterm Birth with Maternal Nativity and Length of Residence among Non-Hispanic Black Women, CJC Open, 2021, ISSN 2589-790X, https://doi.org/10.1016/j.cjco.2021.10.009.

104. Makker K, Zhang M, Wang G, Hong X, Zhang C, Wang X. Determinants of Plasma Insulin Levels and its Trajectory from Birth to Early Childhood among a High-Risk Minority Birth Cohort: Implications for Primordial Prevention of Diabetes. *Pediatric Research* (in press)

105. Hou W, Zhang M, Ji Y, Hong X, Wang G, Liang L, Ji H, Saria S, Wang X. In-utero exposure to cigarette smoking on child long-term risk of obesity: concordance of self-report and maternal and cord blood biomarkers of cigarette smoking. *International Journal of Obesity* (under review)

106. Huang W, Igusa T, Wang G, Buckley JP, Hong X, Bind E, Steffens A, Mukherjee J, Haltmeier D, Ji Y, Xu R, Hou W, Fan Z, Wang X. Effects of in-utero exposure to toxic metal mixture and micronutrients on the risk of overweight or obesity in children from US urban, low-income, underrepresented populations. *International Journal of Obesity* (under review)

107. Zhang M, Yu CH, Wang G, Buckley JP, Hong X, Pearson C, Adams W, Fan Z, Wang X. Longitudinal trajectories and determinants of plasma per- and polyfluoroalkyl substance (PFAS) levels from birth to early childhood and metabolomic associations: A pilot study in the Boston Birth Cohort. *Precision Nutrition.* 2022 (in press)

Neurodevelopmental Outcomes

108. Li M, Fallin MD, Riley A, Landa R, Walker SO, Silverstein M, Caruso D, Pearson C, Kiang S, Dahm JL, Hong X, Wang G, Wang MC, Zuckerman B, Wang X. The Association of maternal obesity and diabetes with autism and other developmental disabilities. *Pediatrics*. 2016 Feb;137(2):e20152206. doi: 10.1542/peds.2015-2206. Epub 2016 Jan 29 *Since publication, it has been cited ~200 times, replicated in diverse populations, and received broad media attention.

109. Brucato M, Ladd-Acosta C, Li M, Caruso D, Hong X, Kaczaniuk J, Stuart EA, Fallin MD, Wang X. Prenatal exposure to fever is associated with Autism Spectrum Disorder in the Boston Birth Cohort. *Autism Research.* First published: 11 August 2017. DOI: 10.1002/aur.1841 *Selected as 2017 top 20 publications by Interagency Autism Coordinating Committee, NIH

110. Raghavan R, Riley AW, Volk H, Caruso D, Hironaka L, Sices L, Hong X, Wang G, Ji Y, Brucato M, Wahl A; Stivers T, Pearson C, Zuckerman B, Stuart EA, Landa R, Fallin DM, Wang X. Maternal multivitamin intake, plasma folate and vitamin B12 levels and ASD risk in offspring. *Pediatrics and Perinatal Epidemiology.* 2017 Oct 6. doi: 10.1111/ppe.12414. [Epub ahead of print] *****Selected as 2017 top 20 publications by Interagency Autism Coordinating Committee, NIH; it is also the most cited paper of *Pediatrics and Perinatal Epidemiology*

111. Okano L, Ji Y, Riley AW, Wang X. Maternal psychosocial stress and children's ADHD diagnosis: a prospective birth cohort study. *J Psychosom Obstet Gynaecol*. 2019 Sep;40(3):217-225. doi: 10.1080/0167482X.2018.1468434. Epub 2018 May 23.
PMID: 29790815 PMCID: PMC6251762 (available on 2020-09-01)

112. Ji Y, Riley AW, Lee LC, Volk H, Hong X, Wang G, Angomas R, Stivers T, Wahl A, Ji H, Bartell TR, Burd I, Paige D, Fallin MD, Zuckerman B, Wang X. A prospective birth cohort study on maternal cholesterol levels and offspring attention deficit hyperactivity disorder: new insight on sex differences. Brain Sci*.* 2017 Dec 23;8(1). pii: E3. doi: 10.3390/brainsci8010003.

113. Ji Y, Hong X, Wang G, Chatterjee N, Riley AW, Lee LC, Surkan P, Bartell TR, Zuckerman B, Wang X. A prospective birth cohort study on early childhood lead levels and attention deficit hyperactivity disorder: new insight on sex differences. *J of Pediatrics*. 2018
DOI: https://doi.org/10.1016/j.jpeds.2018.03.076

114.	Ji Y, Riley AW, Lee LC, Hong X, Wang G, Tsai HJ, Pearson C, Panjwani A, Ji H, Bartell TR, Burd I, Fallin DM, Wang X. Maternal biomarkers of acetaminophen use and attention deficit hyperactivity disorder. *Brain Sci.* 2018 Jul 3;8(7). pii: E127. doi: 10.3390/brainsci8070127. PMID: 29970852
115.	Raghavan R, Zuckerman B, Hong X, Wang G, Ji Y, Paige D, Zhang C, DiBari J, Fallin DM, Wang X. Cord and early childhood plasma leptin, fetal and infancy growth pattern, and development of autism spectrum disorder in the Boston Birth Cohort. *Autism Research* 2018. Published online 2018 in Wiley Online Library (wileyonlinelibrary.com) DOI: 10.1002/aur.2011
116.	Raghavan R, Fallin DM, Hong X, Wang G, Ji Y, Stuart EA, Paige D, Wang X. Cord and early childhood plasma adiponectin levels and autism risk: A prospective birth Cohort study. *Journal of Autism and Developmental Disorders* 2018 Jul 24. doi: 10.1007/s10803-018-3688-5. PMID: 30043356
117.	Panjwani, A.A., Ji, Y., Fahey, J.W., Palmer, A., Wang G., Hong, X., Zuckerman, B., Wang, X. Maternal Obesity/Diabetes, Plasma Branched-Chain Amino Acids (BCAAs), and Offspring Autism Spectrum Disorder Risk: Evidence of Sex Difference. *Autism Research* 2019 Aug 9. doi: 10.1002/aur.2177. PMID: 31400063 [PubMed - as supplied by publisher]
118.	Panjwani, A.A., Ji Y, Fahey JW, Palmer A, Wang G, Hong X, Zuckerman B, Wang X. Maternal Dyslipidemia, Plasma Branched-Chain Amino Acids, and the Risk of Child Autism Spectrum Disorder: Evidence of Sex Difference. *J Autism Dev Disord*. 2020 Feb;50(2):540-550. doi: 10.1007/s10803-019-04264-x. PMID: 31686281.
119.	Raghavan, R., Helfrich B.B, Cerda S.R., Burd I., Wang G., Hong X., Ji Y., Fu L., Pearson C., Fallin D.M., Zuckerman B., Wang X. Preterm birth subtypes, placental pathology findings, and risk of neurodevelopmental disabilities during childhood. *Placenta* 2019 Aug;83:17-25. doi: 10.1016/j.placenta.2019.06.374. Epub 2019 Jun 14. PMID: 31477202 [PubMed - in process]
120.	Ji Y, Azuine RE, Zhang Y, Hou W, Hong X, Wang G, Riley A, Pearson C, Zuckerman B, Wang X. Association of Cord Plasma Biomarkers of In Utero Acetaminophen Exposure With Risk of Attention-Deficit/Hyperactivity Disorder and Autism Spectrum Disorder in Childhood. *JAMA Psychiatry*. 2020 Feb 1;77(2):180-189. doi: 10.1001/jamapsychiatry.2019.3259. PMID: 31664451; PMCID: PMC6822099.
121.	Raghavan R, Selhub J, Paul L, Ji Y, Wang G, Hong X, Zuckerman B, Fallin MD, Wang X. A prospective birth cohort study on cord blood folate subtypes and risk of autism spectrum disorder. *Am J Clin Nutr.* 2020 Nov 11;112(5):1304-1317. doi: 10.1093/ajcn/nqaa208. PMID: 32844208; PMCID: PMC7657337.
122.	Anand N, Ji Y, Wang G, Hong X, van der Rijn M, Riley A, Pearson C, Zuckerman B, Wang X. Maternal and Cord Plasma Branched Chain Amino Acids and Child Risk of Attention Deficit Hyperactivity Disorder: A Prospective Birth Cohort Study. *J Child Psychol Psychiatry.* 2020 Sep 22;. doi: 10.1111/jcpp.13332. [Epub ahead of print] PubMed PMID: 32960988.
123.	Calliope Holingue, Martha Brucato, Christine Ladd-Acosta, Xiumei Hong, Noel Mueller, Heather Volk Xiaobin Wang, Daniele Fallin. Interaction Between Maternal Immune Activation and Antibiotic Use during Pregnancy and Child Risk of Autism Spectrum Disorder. *Autism Research, 2020, Oct 16. Doi:10.1002/aur.2411*.
124.	Bo Y Park, Ruofan Yao, Elaine Tierney, Martha Brucato, Xiumei Hong, Guoying Wang, Yuelong Ji, Colleen Pearson, M. Daniele Fallin, Xiaobin Wang, Heather Volk. The association between maternal lipid profile after birth and offspring risk of Autism Spectrum Disorder. *Ann Epidemiol.* 2020 Sep 9;. doi: 10.1016/j.annepidem.2020.08.009. [Epub ahead of print] PubMed PMID: 32919032; NIHMSID:NIHMS1627783.
125.	Lee ASE, Ji Y, Raghavan R, Wang G, Hong X, Pearson C, Mirolli G, Bind E, Steffens A, Mukherjee J, Haltmeier D, Fan ZT, Wang X. Maternal prenatal selenium levels and child risk of

neurodevelopmental disorders: A prospective birth cohort study. Autism Res. 2021 Dec;14(12):2533-2543. doi: 10.1002/aur.2617. Epub 2021 Sep 24. PMID: 34558795; PMCID: PMC8665097.

126. Ji Y, Raghavan R, Wang X. Early Life Origins of ASD and ADHD. Oxford Research Encyclopedia of Global Public Health, May 2021 https://doi.org/10.1093/acrefore/9780190632366.013.181

127. Anand NS, Raghavan R, Wang G, Hong X, Azuine RE, Pearson C, Zuckerman B, Xie H, Wang X. Perinatal Acetaminophen Exposure and Childhood Attention-Deficit/Hyperactivity Disorder (ADHD): Exploring the Role of Umbilical Cord Plasma Metabolites in Oxidative Stress Pathways. *Brain Sciences*. 2021; 11(10):1302. https://doi.org/10.3390/brainsci11101302

128. Qu X, Lee LC, Ladd-Acosta C, Hong X, Ji Y, Kalb LG, Volk HE, Wang X. Association between atopic diseases and neurodevelopmental disabilities in a longitudinal birth cohort. *Autism Res.* 2022 Feb 2. doi: 10.1002/aur.2680. Epub ahead of print. PMID: 35112480.

129. Brucato M, Lance E, Lanzkron S, Wang X, Pecker L. Developmental disorders in children born to women with sickle cell disease: A report from the Boston Birth Cohort" eJHaem (In press).

Opioids Epidemic: Risk factors and consequences

130. Roy Adams, Yuelong Ji, Xiaobin Wang, Suchi Saria. Learning Models from Data with Measurement Error: Tackling Underreporting. *ICML* (https://www.researchgate.net/publication/330700929)

131. Azuine, E.R. Ji, Y., Chang, H.Y., Kim, Y., Ji, H., DiBari, J., Hong, X., Wang, G., Singh, K.G. Pearson, C., Zuckerman, B., Surkan, J.P., Wang, X. Prenatal Risk Factors, Peri- and Post-natal Outcomes associated with Maternal Opioids Exposure in a U.S. Urban Low-Income Multi-Ethnic Population. *JAMA Network Open*. 2019 Jun 5;2(6):e196405. doi: 10.1001/jamanetworkopen.2019.6405. (see editorial comments)

132. Garrison-Desany H, Nawa N, Kim Y, Ji Y, Chang HY, Hong X, Wang G, Pearson C, Zuckerman BS, Wang X, Surkan PJ. Associations between depressant and stimulant drug use in pregnancy and preterm birth in a sample of US urban low-income multiethnic population. *Public Health Reports*. 2020. May;135(3):383-392. PubMed PMID: 32311304; PubMed Central PMCID: PMC7238708; DOI: 10.1177/0033354920915437.

133. Garrison-Desany HM, Hong X, Mahor BS, Beaty TH, Wang G, Pearson C, Liang L, Wang X, Ladd-Acosta C. Individual and combined association between prenatal polysubstance exposure and childhood risk of attention deficit hyperactivity disorder. *JAMA Network Open* (in press).

134. Nawa N, Garrison-Desany H, Kim Y, Ji Y, Hong X, Wang G, Pearson C, Zuckerman BS, Wang X, Surkan, PJ. Joint effects of maternal marijuana use and tobacco smoking during pregnancy on preterm birth. *Pediatric and Perinatal Epidemiology*. 2020 June 30. PubMed PMID: 32602574; DOI: 10.1111/ppe.12686.

Puberty and Early Life Risk Factors

135. Chen L, Wang G, Bennett WL, Ji Y, Pearson C, Radovick S, Wang X. Longitudinal Trajectory of BMI from Ages 2 to 7 Years and Age at Peak Height Velocity in Boys and Girls of the Boston Birth Cohort. *Journal of Pediatrics* November 26, 2020. DOI:https://doi.org/10.1016/j.jpeds.2020.11.047

136. Wang G, Tang WY, Ji H, Wang X. Prenatal exposure to mercury and precocious puberty: a prospective birth cohort study. *Hum Reprod.* 2021 Feb 18;36(3):712-720. doi: 10.1093/humrep/deaa315. PMID: 33367618; PMCID: PMC7891807.

Impact of COVID-19 on Maternal and Child Health
137. Wang G, Foney DM, DiBari J, Hong X, Showell N, Kim KS, Ji H, Pearson C, Mirolli G, Rusk S, Sharfstein J, Cheng TL, Zuckerman B, Wang X. A prospective cohort study on the intersectionality of obesity, chronic disease, social factors, and incident risk of COVID-19 in US low-income minority middle-age mothers. *International Journal of Obesity* (Lond). 2021 Aug 19:1–8. doi: 10.1038/s41366-021-00943-x. Epub ahead of print. PMID: 34413468; PMCID: PMC8374030.
138. Rusk SA, DiBari J, Foney DM, Li M, Hong X, Wang G, Kim KS, Pearson C, Mirolli G, Cheng TL, Kogan M, Zuckerman B, Wang X. A longitudinal study of psychiatric clinical encounters before and during the COVID-19 pandemic among low-income minority children in Boston, MA. *J Child Psychol Psychiatry* (in submission)

CHILDREN'S SLEEP HABITS QUESTIONNAIRE (ABBREVIATED)

The following statements are about your child's sleep habits and possible difficulties with sleep. Think about the past week in your life when you answer the questions. If last week was unusual for a specific reason, choose the most recent typical week. Unless noted, check Always if something occurs every night, Usually if it occurs 5 or 6 times a week, Sometimes if it occurs 2 to 4 times a week, Rarely if it occurs once a week, and Never if it occurs less than once a week.

BEDTIME

Write in your child's usual bedtime: Weeknights _____:_____ am/pm

Weekends _____:_____ am/pm

	7<br>Always	5-6<br>Usually	2-4<br>Sometimes	1<br>Rarely	0<br>Never
1. Child goes to bed at the same time at night.	( )	( )	( )	( )	( )
2. Child falls asleep within 20 minutes after going to bed.	( )	( )	( )	( )	( )
3. Child falls asleep alone in own bed.	( )	( )	( )	( )	( )
4. Child falls asleep in parent's or sibling's bed.	( )	( )	( )	( )	( )
5. Child falls asleep with rocking or rhythmic movements.	( )	( )	( )	( )	( )
6. Child needs special object to fall asleep (doll, special blanket, stuffed animal, etc.).	( )	( )	( )	( )	( )
7. Child needs parent in the room to fall asleep.	( )	( )	( )	( )	( )
8. Child resists going to bed at bedtime.	( )	( )	( )	( )	( )
9. Child is afraid of sleeping in the dark.	( )	( )	( )	( )	( )

SLEEP BEHAVIOR

Write in your child's usual amount of sleep each day
(combining nighttime sleep and naps): _____ hours and _____ minutes

	7<br>Always	5-6<br>Usually	2-4<br>Sometimes	1<br>Rarely	0<br>Never
10. Child sleeps about the same amount each day.	( )	( )	( )	( )	( )
11. Child is restless and moves a lot during sleep.	( )	( )	( )	( )	( )

	7<br>Always	5-6<br>Usually	2-4<br>Sometimes	1<br>Rarely	0<br>Never
12. Child moves to someone else's bed during the night (parent, sibling, etc.).	( )	( )	( )	( )	( )
13. Child grinds teeth during sleep (your dentist may have told you this).	( )	( )	( )	( )	( )
14. Child snores loudly.	( )	( )	( )	( )	( )
15. Child awakens during the night and is sweating, screaming, and inconsolable.	( )	( )	( )	( )	( )
16. Child naps during the day.	( )	( )	( )	( )	( )
Write in the number of minutes the nap usually lasts: _____ minutes					

WAKING DURING THE NIGHT

	7<br>Always	5-6<br>Usually	2-4<br>Sometimes	1<br>Rarely	0<br>Never
17. Child wakes up once during the night.	( )	( )	( )	( )	( )
18. Child wakes up more than once during the night.	( )	( )	( )	( )	( )

MORNING WAKE UP

Write in the time child usually wakes up in the morning: Weekdays _____:_____ am/pm

Weekends _____:_____ am/pm

	7<br>Always	5-6<br>Usually	2-4<br>Sometimes	1<br>Rarely	0<br>Never
19. Child wakes up by him/herself.	( )	( )	( )	( )	( )
20. Child wakes up very early in the morning (or, earlier than necessary or desired).	( )	( )	( )	( )	( )
21. Child seems tired during the daytime.	( )	( )	( )	( )	( )
22. Child falls asleep while involved in activities.	( )	( )	( )	( )	( )

Covid19 School Housing And Telemedicine Questionnaire

Thank you for participating in the BMC Children's Health Study. This questionnaire is about you and your family's experiences during the Coronavirus (Covid19) Pandemic beginning in March of 2020. You may have answered a questionnaire about Covid Health and Resources earlier in the year, but this is a new questionnaire to ask about additional hardships since the Covid 19 pandemic has continued to impact communities.

These questions ask about access to healthcare, school, housing, physical activity and other resources and hardships. Most research studies cannot be in-person at BMC yet so we are asking our participants to complete our questionnaires using a secure system called REDCap. The survey will take about 10-15 minutes to complete. The Visit ID and Interviewer sections will be filled in for you, so please just fill in the date you are answering the questionnaire and the remainder of the questions. Once you have answered all the questions, please press submit.

Visit ID ______________________________

Response method

◯ Self administered by participant
◯ Administered over the phone
◯ Done in person

This questionnaire asks about some of the ways the Coronavirus (Covid 19) pandemic has affected our daily lives including school, housing, healthcare, and access to food. Please answer the questions based on the events since the beginning of the Coronavirus (Covid 19) pandemic in March 2020. If you have any questions or prefer to answer the survey over the phone please contact Gabie Mirolli at gabrielle.mirolli@bmc.org or (857) 505-4471. If you need help with resources because of hardships you or your family may be experiencing during COVID-19, please visit: BMC Community Resource Guide Este cuestionario pregunta sobre algunas de las maneras en que la pandemia del coronavirus (Covid 19) ha afectado nuestra vida diaria, incluso la escuela, la vivienda, la atención médica y el acceso a la comida. Por favor contesta las preguntas sobre los eventos desde el principio de la pandemia del coronavirus (Covid 19) en marzo de 2020. Si tiene alguna pregunta o prefiere responder al cuestionario por teléfono, por favor contacte con Gabie Mirolli en gabrielle.mirolli@bmc.org o (857) 505-4471. Si usted o su familia necesita ayuda con dificultades durante la pandemia por favor hace clic aqui: BMC Community Resource Guide

This section of the survey is about the changes to schooling that have occurred because of the Covid 19 Pandemic. If you do not have any children or your children are not in grades K-12 please select "none" for the first question in this section.

Esta sección del cuestionario es sobre los cambios a la enseñaza causada por la pandemia de Covid 19. Si no tiene hijos o sus hijos no están en los grados K-12 por favor elegir "none (0)" para la primera pregunta en esta sección.

How many children in grades K-12 do you have?

Cuantos de sus hijos están en los grados K-12?

◯ One (1)
◯ Two (2)
◯ Three (3)
◯ Four (4)
◯ Five (5)
◯ Six (6)
◯ Seven (7)
◯ None (0)
◯ Prefer not to answer (Prefiero no responder

What type of schooling are your child(ren) currently receiving? (Check all that apply)<br><br>¿Qué tipo de aprendizaje reciben sus hijos actualmente? (marque todos que apliquen)<br><br>1. Híbrido (mezcla de remoto y en persona)<br>2. Remoto a tiempo completo<br>3. En persona a tiempo completo	☐ Hybrid (part time remote part time in person)<br>☐ Remote learning full time<br>☐ In person full time
If your child(ren) is/are on the hybrid model, how often are they going into school?<br><br>Si sus hijos usan el modelo híbrido, ¿con qué frecuencia van a la escuela?<br><br>1. Cada dos semanas<br>2. Cada dos días<br>3. Dos días a la semana<br>4. Otro	◯ Every other week ◯ Every other day<br>◯ 2 days a week ◯ Other
If other, please describe how often your child goes into school<br><br>Si es otro, con qué frecuencia van a la escuela	__________________________________
If your child(ren) is/are engaging in remote learning, is there an adult who is able to be at home with them while they are in school?<br><br>Si sus hijos están participando en el aprendizaje remoto, ¿hay un adulto en casa cuando están en clase?<br><br>1. Sí<br>2. No	◯ Yes<br>◯ No
What types of device(s) do your children use to participate in remote learning? (Check all that apply)<br><br>¿Qué tipo de aparato(s) usan sus hijos para participar en el aprendizaje remoto? (Marque todos que apliquen)<br>1. computadora de escritorio<br>2. portátil o Chromebook<br>3. tableta o iPad<br>4. teléfono inteligente<br>5. otro	☐ Desktop computer<br>☐ Laptop computer or Chromebook<br>☐ Tablet or iPad<br>☐ Smartphone<br>☐ Other
If other, please specify what type of device is used<br><br>Si es otro, ¿qué tipo de aparato usan?	__________________________________

If your child(ren) is/are engaged in remote learning do you have enough devices for them to each participate in the programming scheduled by their school(s)?<br><br>Si sus hijos participan en el aprendizaje remoto, tienen suficientes aparatos para que cada hijo puede participar en las lecciones programadas por su escuela?<br><br>1. Sí<br>2. No<br>3. Prefiero no responder	◯ Yes ◯ No ◯ Prefer not to answer
Do any of your children have an IEP (Individualized Education Plan) and/or a 504 Plan at school?<br><br>¿Tiene alguno de sus hijos un programa educativo individualizado (IEP) y/o un plan 504 en escuela?<br>1. sí, un IEP<br>2. sí, un plan 504<br>3, sí, los dos<br>4. no<br>5. prefiero no responder	◯ Yes, an IEP<br>◯ Yes, a 504 Plan<br>◯ Yes, both<br>◯ No<br>◯ Prefer not to answer
What grade is your first child in?<br><br>¿En qué grado está su primer hijo/a?	◯ Kindergarten<br>◯ First grade (primer)<br>◯ Second grade (segundo)<br>◯ Third grade (tercer)<br>◯ Fourth grade (cuarto)<br>◯ Fifth grade (quinto)<br>◯ Sixth grade (sexto)<br>◯ Seventh grade (séptimo)<br>◯ Eighth grade (octavo)<br>◯ Ninth grade (noveno)<br>◯ Tenth grade (décimo)<br>◯ Eleventh grade (undécimo)<br>◯ Twelfth grade (duodécimo)<br>(Child 1)
What grade is your second child in?<br><br>¿En qué grado está su segundo hijo?	◯ Kindergarten<br>◯ First grade (primer)<br>◯ Second grade (segundo)<br>◯ Third grade (tercer)<br>◯ Fourth grade (cuarto)<br>◯ Fifth grade (quinto)<br>◯ Sixth grade (sexto)<br>◯ Seventh grade (séptimo)<br>◯ Eighth grade (octavo)<br>◯ Ninth grade (noveno)<br>◯ Tenth grade (décimo)<br>◯ Eleventh grade (undécimo)<br>◯ Twelfth grade (duodécimo)<br>(Child 2)

What grade is your third child in?<br><br>¿En qué grado está su tercer hijo?	◯ Kindergarten<br>◯ First grade (primer)<br>◯ Second grade (segundo)<br>◯ Third grade (tercer)<br>◯ Fourth grade (cuarto)<br>◯ Fifth grade (quinto)<br>◯ Sixth grade (sexto)<br>◯ Seventh grade (séptimo)<br>◯ Eighth grade (octavo)<br>◯ Ninth grade (noveno)<br>◯ Tenth grade (décimo)<br>◯ Eleventh grade (undécimo)<br>◯ Twelfth grade (duodécimo)<br>(Child 3)
What grade is your fourth child in?<br><br>¿En qué grado está su cuarto hijo?	◯ Kindergarten<br>◯ First grade (primer)<br>◯ Second grade (segundo)<br>◯ Third grade (tercer)<br>◯ Fourth grade (cuarto)<br>◯ Fifth grade (quinto)<br>◯ Sixth grade (sexto)<br>◯ Seventh grade (séptimo)<br>◯ Eighth grade (octavo)<br>◯ Ninth grade (noveno)<br>◯ Tenth grade (décimo)<br>◯ Eleventh grade (undécimo)<br>◯ Twelfth grade (duodécimo)<br>(Child 4)
What grade is your fifth child in?<br><br>¿En qué grado está su quinto hijo?	◯ Kindergarten<br>◯ First grade (primer)<br>◯ Second grade (segundo)<br>◯ Third grade (tercer)<br>◯ Fourth grade (cuarto)<br>◯ Fifth grade (quinto)<br>◯ Sixth grade (sexto)<br>◯ Seventh grade (séptimo)<br>◯ Eighth grade (octavo)<br>◯ Ninth grade (noveno)<br>◯ Tenth grade (décimo)<br>◯ Eleventh grade (undécimo)<br>◯ Twelfth grade (duodécimo)<br>(Child 5)
What grade is your sixth child in?<br><br>¿En qué grado está su sexto hijo?	◯ Kindergarten<br>◯ First grade (primer)<br>◯ Second grade (segundo)<br>◯ Third grade (tercer)<br>◯ Fourth grade (cuarto)<br>◯ Fifth grade (quinto)<br>◯ Sixth grade (sexto)<br>◯ Seventh grade (séptimo)<br>◯ Eighth grade (octavo)<br>◯ Ninth grade (noveno)<br>◯ Tenth grade (décimo)<br>◯ Eleventh grade (undécimo)<br>◯ Twelfth grade (duodécimo)<br>(Child 6)

What grade is your seventh child in?<br><br>¿En qué grado está su séptimo hijo?	◯ Kindergarten<br>◯ First grade (primer)<br>◯ Second grade (segundo)<br>◯ Third grade (tercer)<br>◯ Fourth grade (cuarto)<br>◯ Fifth grade (quinto)<br>◯ Sixth grade (sexto)<br>◯ Seventh grade (séptimo)<br>◯ Eighth grade (octavo)<br>◯ Ninth grade (noveno)<br>◯ Tenth grade (décimo)<br>◯ Eleventh grade (undécimo)<br>◯ Twelfth grade (duodécimo)<br>(Child 7)
Have any of your children ever been held back a grade at school?<br><br>¿Alguno de sus hijos repitió un grado en escuela?<br>1. Sí<br>2. No<br>3. Prefiero no responder	◯ Yes ◯ No ◯ Prefer not to answer
Are there any aspects of in person school that your child is not receiving due to remote learning? (check all that apply)<br><br>¿Hay algunos aspectos de escuela que ahora falta su hijo/a debido al aprendizaje remoto? (Marque todos que apliquen)<br>1. Servicios de educación especial<br>2. Consejería escolar<br>3. Ayuda despues de la escuela<br>4. La socialización con los compañeros<br>5. Programa de cuidado infantil<br>6. Oportunidades por estudio de trabajo<br>7. Ninguno de los arriba<br>8. Otro<br>9. Prefiero no responder	☐ Special education services<br>☐ School counseling<br>☐ After school help<br>☐ Socialization with peers<br>☐ After school childcare program<br>☐ Work study opportunities<br>☐ None of the above<br>☐ Other<br>☐ Prefer not to answer
If other, please specify<br><br>Si es otro, por favor especifique	__________________________________
How have your child(ren) been staying active during the Covid 19 pandemic? (check all that apply)<br><br>¿Cómo mantenerse activo sus hijos durante la pandemia de Covid 19?<br>1. Practicar deportes afuera<br>2. Jugar afuera con los hermanos o amigos<br>3. Ir a caminar<br>4. Hacer ejercicios adentro<br>5. Ninguna actividad física<br>6. Otro<br>7. Ninguno de los arriba	☐ Playing outside sports<br>☐ Playing outside with siblings or friends<br>☐ Going for walks<br>☐ Exercising indoors<br>☐ No physical activity<br>☐ Other<br>☐ None of the above
If other, how has your child been staying active?<br><br>Si es otro, ¿cómo mantenerse activo sus hijos?	__________________________________

Many schools have created a free lunch program that can be picked up at the school, have you utilized this program at your child's school?

Muchas escuelas han creado un programa de almuerzo gratis que puede recoger en la escuela, ¿ha utilizado este programa en la escuela de su hijo?
1. Sí
2. No
3. La escuela de mi hijo no tiene este programa
4. Prefiero no responder

◯ Yes
◯ No
◯ My child's school does not have this program
◯ Prefer not to answer

Have you used any other food assistance programs during the Covid 19 Pandemic? (Check all that apply)

¿Ha utilizado un otro programa de asistencia alimentaria durante la pandemia de Covid 19? (Marque todos que apliquen)
1. Despensa de alimentos patrocinado de BMC
2. Despensa de alimentos patrocinado de una iglesia
3. Despensa de alimentos patrocinado de la comunidad
4. Programa de entrega de comestibles
5. Programa de comidas por los estudiantes durante la fin de semana
6. Ninguno de los arriba
7. Otro
8. Prefiero no responder

☐ BMC sponsored food pantry
☐ Church sponsored food pantry
☐ Community sponsored food pantry
☐ Grocery delivery program
☐ Weekend meal program for students
☐ None of the above
☐ Other
☐ Prefer not to answer

If other, please specify what type of food assistance program

Si es otro, por favor especifique que tipo de asistencia alimentaria usa

____

This section of the survey inquires about your current housing situation. We are trying to understand the hardships that the Covid 19 Pandemic might have brought upon our BMC patients.

Esta sección del cuestionario pregunta sobre su situación de vivienda actual. Queremos entender los dificultades de nuestros pacientes durante la pandemia de Covid 19.

Including yourself, how many people do you live with?

Incluyendo usted misma, ¿con cuantas personas vive usted?

____

What type of home are you currently living in?

¿En qué tipo de hogar vive usted actualmente?
1. Casa unifamiliar
2. Casa multifamiliar
3. Apartamento
4. Condominio
5. Refugio para personas sin hogar
6. Otro
7. Prefiero no responder

◯ Single family house
◯ Multiple family house
◯ Apartment
◯ Condo
◯ Shelter
◯ Other
◯ Prefer not to answer

If other, please specify<br><br>Si es otro, por favor especifique	______________________
How long have you lived in your current home? (answer in YEARS, if less than 1 year please answer below)<br><br>¿Por cuánto tiempo vive en su vivienda actual? (responda en años, si es menos de un año, responda abajo)	______________________<br>(Years)
How long have you lived in your current home (in MONTHS if less than 1 year)<br><br>Por cuánto tiempo vive en su vivienda actual? (responda en meses si es menos de un año)	______________________<br>(Months)
Do you own or rent the home that you currently live in?<br><br>¿Es propietaria o se alquila su vivienda actual?<br>1. Soy propietaria<br>2. Alquilo<br>3. Ninguno de los arriba<br>4. Prefiero no responder	◯ Own<br>◯ Rent<br>◯ None of the above<br>◯ Prefer not to answer
How many bedrooms are in your home?<br><br>¿Cuántas habitaciones hay en su hogar?	______________________
How many bathrooms are in your home?<br><br>¿Cuántos baños hay en su hogar?	______________________
Do you have your own room in the shelter you are staying in?<br><br>¿Tiene su propia habitación en el refugio donde queda?<br>1. Sí<br>2. No	◯ Yes ◯ No
Do you have your own bathroom in the shelter you are staying in?<br><br>¿Tiene su propio baño en el refugio donde queda?<br>1. Sí<br>2. No	◯ Yes ◯ No
What type of fuel do you use to heat your home?<br><br>¿Qué tipo de combustible utiliza para calentar su hogar?<br>1. Petróleo<br>2. Electricidad<br>3. Gas<br>4. Otro<br>5. No sé	◯ Oil ◯ Electricity ◯ Gas<br>◯ Other ◯ Don't know

If other, please specify<br><br>Si es otro, por favor especifique	__________________________________
What type of stove do you use for cooking?<br><br>¿Qué tipo de estufa utiliza para cocinar?<br>1. Gas<br>2. Eléctrica<br>3. Otro<br>4. No sé	◯ Gas ◯ Electric ◯ Other<br>◯ Don't know
If other, please specify<br><br>Si es otro, por favor especifique	__________________________________
Does anyone living in your home smoke cigarettes?<br><br>¿Alguien que vive en su casa fuma cigarrillos?<br>1. Sí<br>2. No<br>3. Prefiero no responder	◯ Yes ◯ No ◯ Prefer not to answer
How many people in your home smoke cigarettes?<br><br>¿Cuantas personas en su casa fuma cigarrillos?	__________________________________
Where are the cigarettes smoked?<br><br>¿Dónde fuma los cigarrillos?<br>1. Dentro de la casa<br>2. Fuera de la casa<br>3. Dentro y fuera de la casa<br>4. Prefiero no responder	◯ Inside the home<br>◯ Outside the home<br>◯ Both inside and outside the home<br>◯ Prefer not to answer
Does anyone living in your home vape?<br><br>¿Alguien que vive en su casa fuma cigarrillos electrónicos?	◯ Yes ◯ No ◯ Prefer not to answer
Many of our families are struggling to keep up with rent. Are you behind on rent?<br><br>Muchas de nuestras familias están luchando para pagar el alquiler. Está atrasado en el alquiler?<br>1. Sí<br>2. No<br>3. Prefiero no responder	◯ Yes ◯ No ◯ Prefer not to answer
Are you at risk of eviction?<br><br>Está en riesgo de ser desolojado?<br>1. Sí<br>2. No<br>3. Prefiero no responder	◯ Yes ◯ No ◯ Prefer not to answer

Many of our families are struggling to keep up with housing payments. Are you behind on paying your mortgage?

Muchas de nuestras familias están luchando para pagar su hipoteca. ¿Está atrasado en el hipoteca?
1. Sí
2. No
3. Prefiero no responder

○ Yes ○ No ○ Prefer not to answer

Are you at risk of foreclosure?

¿Está en riesgo de ejeción hipotecaria?
1. Sí
2. No
3. Prefiero no responder

○ Yes ○ No ○ Prefer not to answer

Because of the Covid 19 Pandemic we have seen an increase in doctors and patients participating in healthcare over the phone or on video calls. This section of the questionnaire is focused on understanding if and how our participants have interacted with telehealth during the Covid 19 Pandemic.

Durante la pandemia de Covid 19 hemos visto un aumento de médicos y pacientes que participan en la atención médica por teléfono o en videollamadas. Esta sección del cuestionario es enfocada a entender como nuestros participantes han interactuado con la telesalud durante la pandemia de Covid 19.

How do you normally get to your healthcare appointments?

¿Normalmente, cómo llega a sus citas médicas?
1. Bus
2. Tren/ Metro
3. Conducir
4. Un amigo o miembro de mi familia me lleva
5. Tomar un Uber o Lyft
6. Caminar
7. Ir en bici
8. Otro

☐ Bus
☐ Train
☐ Drive myself
☐ Get a ride with a family member or friend
☐ Take an Uber or Lyft
☐ Walk
☐ Bike
☐ Other

If other, please specify

Si es otro, por favor especifique

____

Since the onset of the Covid 19 Pandemic in March 2020 have you or your children participated in phone or video calls for health purposes?

¿Ha participado usted o su hijo(s) en una cita médica por llamada o video llamada desde el principio de la pandemia de Covid 19 en marzo 2020?
1. Sí- yo
2. Sí- mi hijo/a
3. Sí- mi hijo/a y yo
4. No
5. Prefiero no responder

○ Yes-me
○ Yes- my child
○ Yes- both me and my child
○ No
○ Prefer not to answer

What device(s) do you typically use for telehealth visits? (Check all that apply)<br><br>¿Qué tipo de aparato(s) usa para participar en las citas de la telesalud? (Marque todos que apliquen)<br>1. computadora de escritorio<br>2. portátil o Chromebook<br>3. tableta o iPad<br>4. teléfono inteligente<br>5. otro<br>6. Prefiero no responder	☐ Desktop computer<br>☐ Laptop computer or chromebook<br>☐ Tablet or iPad<br>☐ Smartphone<br>☐ Other<br>☐ Prefer not to answer
If other, what type of device do you use for telehealth appointments?<br><br>Si es otro, ¿qué tipo de apartato usa para sus citas de telesalud?	__________________________________
What type(s) of visits did you have over the phone or video? (Check all that apply)<br><br>¿Qué tipo(s) de cita tiene por llamada o videollamada? (Marque todos que apliquen)<br>1. atención primaria para adultos<br>2. atención primaria pediátrica<br>3. pediatría del desarrollo<br>4. la neurología<br>5. la psiquiatría o la psicología<br>6. la endocrinología<br>7. la oftalmóloga<br>8. gastrointestinal<br>9. la hematología<br>10. la cardiología<br>11. tocoginecología<br>12. la dermatología<br>13. la odontología<br>14. otro<br>15. Prefiero no responder	☐ Adult Primary Care<br>☐ Pediatric Primary Care<br>☐ Developmental/Behavioral Pediatrics<br>☐ Neurology<br>☐ Psychiatry/Psychology<br>☐ Endocrinology<br>☐ Eye appointment<br>☐ GI<br>☐ Hematology<br>☐ Cardiology<br>☐ OB/GYN<br>☐ Dermatology<br>☐ Dental Clinic<br>☐ Other<br>☐ Prefer not to answer
If other, what type of visit did you have on the phone or through video?<br><br>Si es otro, ¿qué tipo de cita tuve por una llamada o videollamada?	__________________________________
When compared to previous in-person appointments, I feel that telehealth meets all of my healthcare needs.<br><br>Comparado con mis citas anteriores (en persona), siento como la telesalud satisfacer mis necesidades médicas.<br>1. Totalmente de acuerdo<br>2. De acuerdo<br>3. Neutral<br>4. En desacuerdo<br>5. Totalmente en desacuerdo<br>6. Prefiero no responder	◯ Strongly agree<br>◯ Agree<br>◯ Neutral<br>◯ Disagree<br>◯ Strongly disagree<br>◯ Prefer not to answer

What were your specific concerns related to why telehealth did not meet your needs?

¿Cuáles son sus preocupaciones específicas relacionado con por qué la telesalud no satisfacer sus necesidades?

____

Please check off any advantages of having a doctors appointment over the phone or on video (Check all that apply)

Por favor marque alguna ventaja de tener una cita medica por llamada o videollamada (Marque todos que apliquen)
1. Ahorro tiempo porque no viajar
2. No necesito arreglar el transporte
3. Reducir el riesgo de enfermarse
4. Es fácil para programar
5. No necesito buscar el cuidado de los niños
6. Comodidad aumentado al hablar sobre problemas de salud
7. Otro
8. Ninguno de los arriba
9. Prefiero no responder

- ☐ Saving time by not traveling
- ☐ Not having to arrange transportation
- ☐ Reducing the risk of getting sick by staying home
- ☐ Ease of scheduling
- ☐ Not having to find childcare
- ☐ Increased comfort talking about health problems
- ☐ Other
- ☐ None of the above
- ☐ Prefer not to answer

Please list any other advantages about having telehealth appointments

Por favor desriba cualquier otra ventaja de las citas de telesalud

____

Please also check off any disadvantages you saw while having telehealth appointments (Check all that apply)

Por favor marque alguna desventaja de las citas de telesalud (Marque todos que apliquen)
1. Falta de examen físico
2. No tengo un lugar tranquilo para hablar en privado con mi médico
3. Problemas tecnológicos durante mi cita
4. Comodidad reducida hablando sobre problemas de salud
5. Otro
6. Ninguno de los arriba
7. Prefiero no responder

- ☐ Lack of physical examination
- ☐ I don't have a quiet area to speak privately with my doctor
- ☐ Technological issues during my appointment
- ☐ Reduced comfort talking about health problems
- ☐ Other
- ☐ None of the above
- ☐ Prefer not to answer

Please list any other disadvantages about having telehealth appointments

Por favor desriba cualquier otra desventaja de las citas de telesalud

____

How likely are you to continue to schedule telehealth appointments after the Covid 19 pandemic restrictions are over?

¿Cuál es la probabilidad de continua hacer citas de telesalud después de las restricciones de la pandemia de Covid 19?
1. Muy probable
2. Probable
3. Neutral
4. Improbable
5. Muy improbable
6. Prefiero no responder

- ◯ Very likely
- ◯ Somewhat likely
- ◯ Indifferent
- ◯ Somewhat unlikely
- ◯ Very unlikely
- ◯ Prefer not to answer

What type of visits would you continue to schedule as telehealth after the Covid 19 pandemic restrictions? (Check all that apply)

¿Qué tipo de cita continuaría de hacer como telesalud después de las restricciones de la pandemia de Covid 19? (Marque todos que apliquen)
1. atención primaria para adultos
2. atención primaria pediátrica
3. pediatría del desarrollo
4. la neurología
5. la psiquiatría o la psicología
6. la endocrinología
7. la oftalmóloga
8. gastrointestinal
9. la hematología
10. la cardiología
11. tocoginecología
12. la dermatología
13. la odontología
14. otro
15. Prefiero no responder

- ☐ Adult Primary Care
- ☐ Pediatric Primary Care
- ☐ Developmental/Behavioral Pediatrics
- ☐ Neurology
- ☐ Psychiatry/Psychology
- ☐ Endocrinology
- ☐ Eye appointment
- ☐ GI
- ☐ Hematology
- ☐ Cardiology
- ☐ OB/GYN
- ☐ Dermatology
- ☐ Dental Clinic
- ☐ Other
- ☐ Prefer not to answer

If other, what type of appointment would you continue as telehealth after the Covid 19 pandemic restrictions?

Si es otro, ¿qué tipo de cita continuaría como telesalud después de las restricciones de la pandemia de Covid 19?

____

This section of the questionnaire is about resources and your access to food before and during the Covid 19 Pandemic, beginning in March 2020. We are looking to better understand the hardships that were created by the Covid 19 Pandemic.

Esta sección del cuestionario es sobre sus recursos y su acceso a la comida antes de y durante la pandemia de Covid 19, hasta el principio en Marzo 2020. Queremos entender los dificultades creado por la pandemia de Covid 19.

In the last 12 months, did you ever run out of food before you were able to purchase more?<br><br>Durante los últimos 12 meses ha queda sin comida antes de podía comprar más?<br><br>1. Sí, ANTES de la pandemia<br>2. Sí, DURANTE la pandemia<br>3. Sí antes de Y durante la pandemia<br>4. No<br>5. Prefiero no responder	◯ Yes, BEFORE the pandemic<br>◯ Yes, DURING the pandemic<br>◯ Yes, BOTH before and during the pandemic<br>◯ No<br>◯ Prefer not to answer
In the last 12 months, were you ever unable to afford to eat balanced meals?<br><br>Durante los últimos 12 meses había alguna vez que no se puede permitir comidas balanceadas?<br>1. Sí, ANTES de la pandemia<br>2. Sí, DURANTE la pandemia<br>3. Sí antes de Y durante la pandemia<br>4. No<br>5. Prefiero no responder	◯ Yes, BEFORE the pandemic<br>◯ Yes, DURING the pandemic<br>◯ Yes, BOTH before and during the pandemic<br>◯ No<br>◯ Prefer not to answer
During the last 12 months, have you or other adults in your household cut the size of your meals or skip meals because there wasn't enough money for food?<br><br>Durante los últimos 12 meses necesita usted o otros adultos en su casa come menos durante comidas o falta comidas porque no hay suficiente dinero para comida?<br>1. Sí, ANTES de la pandemia<br>2. Sí, DURANTE la pandemia<br>3. Sí antes de Y durante la pandemia<br>4. No<br>5. Prefiero no responder	◯ Yes, BEFORE the pandemic<br>◯ Yes, DURING the pandemic<br>◯ Yes, BOTH before and during the pandemic<br>◯ No<br>◯ Prefer not to answer
If yes, how often did you or other adults in your household cut the size of your meals or skip meals because there wasn't enough money for food? (check all that apply)<br><br>De ser así con que frecuencia come menos durante comidas o falta comidas? (Marque todos que apliquen)<br>1. Casi todos los meses antes de la pandemia<br>2. Unos meses antes de la pandemia<br>3. Un par de veces antes de la pandemia<br>4. Casi todos los meses durante la pandemia<br>5. Unos meses durante la pandemia<br>6. Un par de veces durante la pandemia<br>7. No sé<br>8. Prefiero no responder	☐ Almost every month before the pandemic<br>☐ Some months before the pandemic<br>☐ Once or twice before the pandemic<br>☐ Almost every month during the pandemic<br>☐ Some months during the pandemic<br>☐ Once or twice during the pandemic<br>☐ Not sure<br>☐ Prefer not to answer

In the last 12 months, did you ever eat less than you felt you should because there wasn't enough money to buy food?<br><br>Durante los últimos 12 meses comía menos porque no había suficiente dinero para comprar comida?<br>1. Sí, ANTES de la pandemia<br>2. Sí, DURANTE la pandemia<br>3. Sí antes de Y durante la pandemia<br>4. No<br>5. Prefiero no responder	◯ Yes, BEFORE the pandemic<br>◯ Yes, DURING the pandemic<br>◯ Yes, BOTH before and during the pandemic<br>◯ No<br>◯ Prefer not to answer
In the last 12 months, were you ever hungry but didn't eat because you couldn't afford enough food?<br><br>Durante los últimos 12 meses, tenía hambre pero no comer porque no podía comprar comida?<br>1. Sí, ANTES de la pandemia<br>2. Sí, DURANTE la pandemia<br>3. Sí antes de Y durante la pandemia<br>4. No<br>5. Prefiero no responder	◯ Yes, BEFORE the pandemic<br>◯ Yes, DURING the pandemic<br>◯ Yes, BOTH before and during the pandemic<br>◯ No<br>◯ Prefer not to answer

Because of the disruption that the Covid 19 Pandemic has caused to daily life, people may be feeling increased stress levels and need for mental healthcare services. This portion of the questionnaire aims to understand how this has affected our BMC patients.

Por causa de la interrupción que la pandemia de Covid 19 ha causado a la vida diara, muchas personas sienten más estresados y la necesidad para los servicios de salud mental. Esta sección del cuestionario tiene objectivo de entender cómo este ha afectado nuestros pacientes.

How would you rate the level of stress in your life in general?<br><br>En general, ¿cómo evaluaría el nivel de estrés en su vida?<br>1. Bajo<br>2. Moderado<br>3. Alto	◯ Low ◯ Moderate ◯ High
How would you rate the level of stress in your life since the beginning of the Covid 19 Pandemic in March 2020?<br><br>¿Cómo evaluaría el nivel de estrés en su vida desde el principio de la pandemia de Covid 19 en Marzo 2020?<br>1. Bajo<br>2. Moderado<br>3. Alto	◯ Low ◯ Moderate ◯ High

Since the beginning of the Covid 19 Pandemic in March 2020 how often have you felt that you were NOT able to control the important things in your life?<br><br>Desde el principio de la pandemia de Covid 19 en Marzo 2020, con qué frecuencia siente como no puede controllar las cosas importantes en su vida?<br>1. Nunca<br>2. Casi nunca<br>3. A veces<br>4. Casi todo el tiempo<br>5. Todo el tiempo<br>6. Prefiero no responder	◯ Never<br>◯ Almost never<br>◯ Sometimes<br>◯ Almost all of the time<br>◯ All of the time<br>◯ Prefer not to answer
Compared to your stress level prior to the beginning of the Covid 19 Pandemic in March 2020 how do you rate your stress level?<br><br>Comparado con su nivel de estrés antes del principio de la pandemia de Covid 19 en Marzo 2020, cómo evaluaría su nivel de estrés?<br>1. Más bajo<br>2. Igual<br>3. Más alto	◯ Lower ◯ About the same<br>◯ Higher
How would you rate your level of stress regarding your child's education while they are doing remote learning?<br><br>¿Cómo evaluaría su nivel de estrés sobre la educación de su hijo cuando hacer el aprendizaje remoto?<br>1. Sin estrés<br>2. Estrés leve<br>3. Estrés moderado<br>4. Estrés severo<br>5. Prefiero no responder	◯ No stress<br>◯ Mild stress<br>◯ Moderate stress<br>◯ Severe stress<br>◯ Prefer not to answer
Since the beginning of the Covid 19 Pandemic in March 2020 have you experienced any of these major stressful events? (Check all that apply)<br><br>¿Ha experimentado alguno de estes eventos estresantes desde el principio de la pandemia de Covid 19? (Marque todos que apliquen)<br>1. Muerte de un miembro de la familia<br>2. Divorcio/ruptura<br>3. Perdió un empleo<br>4. Enfermedad severa<br>5. Enfermedad severa de un miembro de la familia<br>6. Muda/ perdió el hogar<br>7. Desalojo<br>8. Ninguno de los arriba<br>9. Prefiero no responder	☐ Death of a family member<br>☐ Divorce/ breakup<br>☐ Lost job<br>☐ Severe illness<br>☐ Severe illness of a family member<br>☐ Moved or lost where I was staying<br>☐ Eviction<br>☐ None of the above<br>☐ Prefer not to answer

Did you or your child seek mental healthcare services due to the stress of the Covid 19 Pandemic that you had not received prior?

¿Busca usted o su hijo/a servicios de salud mental debido al estrés de la pandemia de Covid 19 que no ha recibido antes?
1. Sí- yo
2. Sí- mi hijo/a
3. Sí mi hijo/a y yo
4. No
5. Prefiero no responder

- ◯ Yes- me
- ◯ Yes- my child
- ◯ Yes- both me and my child
- ◯ No
- ◯ Prefer not to answer

We would like to gain an understanding of our participants feelings regarding a Coronavirus (Covid 19) vaccine. Please answer the following questions based on your opinions for when a vaccine becomes available. If you have received the vaccine, please still respond to the questions regarding your attitudes towards it.

Queremos entender los sentimientos de nuestros participantes sobre la vacuna de Covid 19, Por favor conteste las preguntas sobre sus opiniones cuando hay una vacuna disponible.

When a Covid 19 vaccine becomes available how likely are you to get the vaccine yourself?

Cuando una vacuna de Covid 19 está disponible, cuál es la probabilidad que se vacuna?
1. Muy probable
2. Probable
3. Neutral
4. Improbable
5. Muy improbable
6. Prefiero no responder

- ◯ Highly likely
- ◯ Very likely
- ◯ Neutral
- ◯ Unlikely
- ◯ Highly unlikely
- ◯ Prefer not to answer

What are the reasons you would get the Covid 19 vaccine? (Check all that apply)

¿Cuáles son las razones que se vacunaría de Covid 19? (Marque todos que apliquen)
1. Mantenerme saludable
2. Volver a trabajo sin peligro
3. Sentirse más seguro en lugares públicos
4. Mantenerse sanos a mis amigos y familia
5. Otro

- ☐ To stay healthy
- ☐ To safely return to work
- ☐ To feel safer in public places
- ☐ To keep my friends and family healthy
- ☐ Other

If other, please specify why you would get the Covid 19 vaccine

Si es otro, por favor especifique porque se vacunaría de Covid 19

____

What are the reasons you would not get the Covid 19 vaccine? (Check all that apply)<br><br>¿Cuáles son las razones que no se vacunaría de Covid 19? (Marque todos que apliquen)<br>1. Ya tuve Covid 19<br>2. No pienso que necesito vacunarme<br>3. Estoy preocupada sobre la seguridad y los efectos secundarios<br>4. No creo en una vacuna de Covid 19<br>5. Otro	☐ I already had Covid 19<br>☐ I don't think I need to get the vaccine<br>☐ I am concerned about the safety and side effects<br>☐ I don't believe in a vaccine for Covid 19<br>☐ Other
If other, please specify why you would not get the Covid 19 vaccine<br><br>Si es otro, por favor especifique porque no se vacunaría de Covid 19	______________________________
When a Covid 19 Vaccine becomes available how likely are you to have your child get the vaccine?<br><br>Cuando una vacuna de Covid 19 está disponible, cuál es la probabilidad que se vacuna su hijo/a?<br>1. Muy probable<br>2. Probable<br>3. Neutral<br>4. Improbable<br>5. Muy improbable<br>6. No se aplica a mi<br>7. Prefiero no responder	◯ Highly likely<br>◯ Very likely<br>◯ Neutral<br>◯ Unlikely<br>◯ Highly unlikely<br>◯ Does not apply to me<br>◯ Prefer not to answer
What are the reasons you would get the Covid 19 vaccine for your child(ren)? (Check all that apply)<br><br>¿Cuáles son las razones que se vacunaría su hijo/a de Covid 19? (Marque todos que apliquen)<br>1. Mantenerse saludable<br>2. Volver a escuela sin peligro<br>3. Sentirse más seguro en lugares públicos<br>4. Mantenerse sanos a mis amigos y familia<br>5. Otro	☐ To keep them healthy<br>☐ To safely return to school<br>☐ To feel safer in public places<br>☐ To keep friends and family healthy<br>☐ Other
If other, please specify why you would get the Covid 19 vaccine for your child(ren)<br><br>Si es otro, por favor especifique porque se vacunaría su hijo/a de Covid 19	______________________________

What are the reasons you would not get the Covid 19 vaccine for your child? (Check all that apply)<br><br>¿Cuáles son las razones que no se vacunaría su hijo/a de Covid 19? (Marque todos que apliquen)<br>1. Ya tuvo Covid 19<br>2. Niños no necesitan vacunarse porque no se ponen muy enfermos de Covid 19<br>3. Quiero ver cómo afectar a los demas antes de mi hijo/a se vacuna<br>4. Estoy preocupada sobre la seguridad y los efectos secundarios<br>5. Otro	☐ My child(ren) already had Covid 19<br>☐ Children don't need a vaccine because they don't get very sick from Covid 19<br>☐ I want to see how the vaccine affects others before giving it to my child<br>☐ I am concerned about the safety and side effects<br>☐ Other
If other, please specify why you would not get a Covid 19 vaccine for your child<br><br>Si es otro, por favor especifique porque no se vacunaría su hijo/a de Covid 19	__________________________________
Who completed this form?<br><br>¿Quién completó este formulario?<br>1. Madre<br>2. Niño/a adulto/a	◯ Mother<br>◯ Adult Child
Interview Date<br><br>Fecha	__________________________________

NIDA Clinical Trials Network

Fagerstrom Test for Nicotine Dependence (FND)

Segment: _ _

Visit Number: _ _

Date of Assessment: (mm/dd/yyyy) _ _/_ _/_ _ _ _

Do you currently smoke cigarettes?

☐No ☐Yes

If "yes," read each question below. For each question, enter the answer choice which best describes your response.

1. **How soon after you wake up do you smoke your first cigarette?**

   ☐Within 5 minutes ☐31 to 60 minutes

   ☐6 to 30 minutes ☐After 60 minutes

2. **Do you find it difficult to refrain from smoking in places where it is forbidden** (e.g., in church, at the library, in the cinema)**?**

   ☐No ☐Yes

3. **Which cigarette would you hate most to give up?**

   ☐The first one in the morning ☐Any other

4. **How many cigarettes per day do you smoke?**

   ☐10 or less ☐21 to 30

   ☐11 to 20 ☐31 or more

5. **Do you smoke more frequently during the first hours after waking than during the rest of the day?**

   ☐No ☐Yes

6. **Do you smoke when you are so ill that you are in bed most of the day?**

   ☐No ☐Yes

Comments:

Heatherton TF, Kozlowski LT Frecker RC (1991). The Fagerström Test for Nicotine Dependence: A revision of the Fagerström Tolerance Questionnaire. British Journal of Addiction 86:1119-27.

NIDA Clinical Trials Network

Fagerstrom Test for Nicotine Dependence (FND)

Instructions

Clinic personnel will follow standard scoring to calculate score based on responses.

Your score was: (your level of dependence on nicotine is): _ _

Heatherton TF, Kozlowski LT Frecker RC (1991). The Fagerström Test for Nicotine Dependence: A revision of the Fagerström Tolerance Questionnaire. British Journal of Addiction 86:1119-27.

Follow-UP

Family ID	______________________
Visit ID	______________________<br>(IN__)
Date of last interview	______________________
Interview Date	______________________
Interviewer's Name	______________________<br>(First and last name)
Location of Interview	______________________
Child's home zipcode<br><br>¿Qúe es su código poste?	______________________

SCREENING: FOR INTERVIEWS
Eligibility

Are you this child's legal guardian?<br><br>¿Tiene usted custodia legal de...?	◯ Yes<br>◯ No<br>(IF NO STOP)
Are you this child's biological mother?<br><br>¿Usted es la madre biológica de..., verdad?	◯ Yes<br>◯ No
Mother's Name Matches Query	◯ Yes<br>◯ No
Child's Name Matches Query	◯ Yes<br>◯ No

IF NO STOP

Section I. Family Pedigree

Can I ask you a few questions about your child's biological father's medical history?<br><br>¿Puedo preguntar sobre el historial médico del padre?	◯ Yes<br>◯ No

Father's Birth Month<br><br>¿Cuál es su fecha de nacimiento?	______________________<br>(Month)
Father's Birth Year<br><br>¿Cuál es su fecha de nacimiento?	______________________<br>(Year)
Father's Medical History<br><br>Usted sabe si el padre de ...tiene algunas enfermedades como<br><br>Alergias alimentarias<br>Eccema<br>Asma<br>Alergias estacionales<br>Alergias a medicinas<br>Otros<br>Reflujo de ácido	☐ Food Allergy<br>☐ Eczema<br>☐ Asthma<br>☐ Hay Fever<br>☐ Drug Allergy<br>☐ Other Allergies<br>☐ EE<br>☐ GERD
Do you have any other children with her/his father? (Full sibling)<br><br>¿Tiene ud. otros hijos con el padre de (index kid)?	◯ Yes<br>◯ No
Full Sibling 1. Gender	☐ Male<br>☐ Female
Full Sibling 1 Birth Month<br><br>¿Cuál es la fecha de nacimiento de el/ella?	______________________<br>(Month)
Full Sibling 1 Birth Year<br><br>¿Cuál es la fecha de nacimiento de el/ella?	______________________<br>(Year)
Full Sibling 1 Medical History<br><br>Tiene algunas enfermedades como<br><br>Alergias alimentarias<br>Eccema<br>Asma<br>Alergias estacionales<br>Alergias a medicinas<br>Otros<br>Reflujo de ácido	☐ Food Allergy<br>☐ Eczema<br>☐ Asthma<br>☐ Hay Fever<br>☐ Drug Allergy<br>☐ Other Allergies<br>☐ EE<br>☐ GERD
Full Sibling 2 Gender	◯ Male<br>◯ Female
Full Sibling 2 Birth Month	______________________<br>(Month)
Full Sibling 2 Birth Year	______________________<br>(year)

Full Sibling 2 Medical History	☐ Food Allergy<br>☐ Eczema<br>☐ Asthma<br>☐ Hay Fever<br>☐ Drug Allergy<br>☐ Other Allergies<br>☐ EE<br>☐ GERD
Full Sibling 3 Gender	◯ Male<br>◯ Female
Full Sibling 3 Birth Month	__________________________________<br>(Month)
Full Sibling 3 Birth Year	__________________________________<br>(Year)
Full Sibling 3 Medical History	☐ Food Allergy<br>☐ Eczema<br>☐ Asthma<br>☐ Hay Fever<br>☐ Drug Allergy<br>☐ Other Allergies<br>☐ EE<br>☐ GERD
Full Sibling 4 Gender	◯ Male<br>◯ Female
Full Sibling 4 Birth Month	__________________________________<br>(Month)
Full Sibling 4 Birth Year	__________________________________<br>(Year)
Full Sibling 4 Medical History	☐ Food Allergy<br>☐ Eczema<br>☐ Asthma<br>☐ Hay Fever<br>☐ Drug Allergy<br>☐ Other Allergies<br>☐ EE<br>☐ GERD
Full Sibling 5 Gender	◯ Male<br>◯ Female
Full Sibling 5 Birth Month	__________________________________<br>(Month)

Full Sibling 5 Birth Year

____
(Year)

Full Sibling 5 Medical History

- ☐ Food Allergy
- ☐ Eczema
- ☐ Asthma
- ☐ Hay Fever
- ☐ Drug Allergy
- ☐ Other Allergies
- ☐ EE
- ☐ GERD

1. Since the last interview, has your child had any of the following illnesses?

¿en el ultimo año, tenía...algunas enfermedades como?

	Yes	No	Unsure
Common Cold / Gripe	○	○	○
Gastric/intestinal infection / Infección intestinal	○	○	○
Conjunctivitis/ Pink eye / Conjunctivitis	○	○	○
Strep Throat / Infeccion de garganta (faringitis estreptocócica)	○	○	○
RSV/Bronchiolitis / Bronquilitis	○	○	○
If yes, hospitalized	○	○	○
Bronchitis / Bronquitis	○	○	○
Ear Infection / Infección de oreja	○	○	○
Pneumonia / Pulmonía	○	○	○
Skin Infection / Infección de piel	○	○	○
Urinary Tract Infection / Infección urinaria	○	○	○
Parasite Infection / Infección de parasito	○	○	○
Bone Infection (osteomyelitis) / Infección de hueso	○	○	○
Meningitis	○	○	○
Bacteremia/Sepsis (Blood Infection) / Infección de sangre	○	○	○
Sinus Infection / Infección de sino	○	○	○

Has your child been diagnosed with any other illnesses within the last year? / ¿En el año pasado, ... ha sido diagnosticado con algunas otras enfermedades? ○ ○ ○

Other illness specify ________________________________

Other illness specify ________________________________

If yes, how many times?
Cold

¿Cuantas veces tenía? ________________________________

If yes, how many times?
Gastric/Intestinal Infection ________________________________

If yes, how many times?
Conjunctivitis ________________________________

If yes, how many times?
Strep Throat ________________________________

If yes, how many times?
Ear Infection ________________________________

If yes, how many times?
Pneumonia ________________________________

If yes, how many times?
Skin Infection ________________________________

If yes, how many times?
Urinary Tract Infection ________________________________

2. Antibiotics are medicines that your doctor prescribes for illnesses caused by infections. Examples of some names of commonly prescribed antibiotics are amoxicillin and penicillin. Since the last visit did your child take any antibiotics by oral or IV. Not topical antibiotics?

¿En el último año, tomó antibióticos? Oral o intravenosa

○ Yes
○ No
○ Unsure

If yes, how many times was your child prescribed an antibiotic medicine since the last visit?

¿Cuántas veces fue recetado un antibiótico?

________________________________ (times)

3a. Is the child YOUNGER than 5 years old?

○ Yes
○ No

3b. Currently, did anyone other than your child's parent help in caring for your child for even part of the day? (nanny, daycare, preschool, relative)<br><br>Durante el día, hay alguna diferente de los padres que cuida de ... como	◯ Yes<br>◯ No<br>◯ Not sure
Childcare/preschool<br>Days per week<br><br>Una guardería<br>Cuantas días por semana	______________________<br>(# of days per week)
Childcare/preschool<br># of other children<br><br>Una guardería<br>Número de otros niños	______________________
Childcare/preschool<br>Don't Know	◯ Don't know
Home Based Child Care (not in own home)<br># of days per week<br><br>Otra pariente / niñera en otra casa<br>Cuantos días por semana	______________________<br>(# of days per week)
Home Based Child Care (not in own home)<br># of other children<br><br>Otra pariente / niñera en otra casa<br>Número de otros niños	______________________<br>(# of other children)
Home Based Child Care (not in own home)	◯ Don't Know
In home care (in own home, nanny)<br># of days per week<br><br>Una niñera u otro pariente en su casa<br>Cuantos días por semana	______________________<br>(# of days per week)
In Home Care (in own home, nanny)<br># of other children<br><br>Una niñera u otro pariente en su casa<br>Número de otros niños	______________________<br>(# of other children)
In Home Care (in own home, nanny)	◯ Don't Know
4a. Are you, the mother, currently breastfeeding this child?<br><br>¿Ud. Está dar el pecho este hijo ahora?	◯ Yes<br>◯ No

4b. If you are currently breastfeeding do you (the mother) take medications for gastrointestinal upset?<br><br>¿Ud. Toma medicinas para dolor del estómago?	◯ No<br>◯ Yes<br>◯ Unsure
If YES, which one of the following medications did you take?	◯ Antacids (Mylanta, Rolaids, TUMS, Pepto-Bismol)<br>◯ H2 Blockers (Pepcid AC, Zantac)<br>◯ Proton Pump inhibitors (Aciphex, Prilosec, Preveacid, Nexium)<br>◯ Prokinetic agents (Urecholine, Regland, Erythromycin)<br>◯ Unsure<br>◯ Other
If Others, specify:	__________________________________<br>(Other GI medications taken during breast feeding)

5. In a typical week during the period of breast feeding, how often did you (THE MOTHER) eat the following foods?

	None	< 1 days	1-2 days	3-5 days	6-7 days	Unsure
Cow's milk/Dairy Products/Cheese (Leche, queso, productos lactos)	◯	◯	◯	◯	◯	◯
Egg (Huevos)	◯	◯	◯	◯	◯	◯
Peanut (including peanut butter) (Cacahuete/ maní (Incluyendo crema/mantequilla de maní)	◯	◯	◯	◯	◯	◯
Tree Nuts (ie almond, cashew, filbert/hazel, walnut, brazil, macadamia, pecan, pine, pistachio) / (Nueces de árbol)	◯	◯	◯	◯	◯	◯
Fish (ie salmon, tuna, catfish, cod, flounder, halibut, trout, bass) / Pescado	◯	◯	◯	◯	◯	◯
Shellfish (shrimp, crab, lobster, clam, oyster, mussels) / Mariscos	◯	◯	◯	◯	◯	◯
Wheat (ie pasta, bread, cereal) / Productos de trigo	◯	◯	◯	◯	◯	◯
Soy/Tofu / Soja/tofu	◯	◯	◯	◯	◯	◯
Seeds (ie sesame, sunflower, pumpkin) / Semilla (sésamo)	◯	◯	◯	◯	◯	◯
Green vegetables / Verduras verdes	◯	◯	◯	◯	◯	◯

Orange veggies (carrots, squash, etc) / Verduras naranjas	○	○	○	○	○	○
Fruits / Frutas	○	○	○	○	○	○
Meats / Carne	○	○	○	○	○	○
Beans / Frijoles	○	○	○	○	○	○
Rice / Arroz	○	○	○	○	○	○

6. At present, does your child take any nutritional supplements or vitamins?

¿Toma ... algunas vitaminas?

○ Yes
○ No

If YES, on average how many days per week does your child take a nutritional supplement or vitamin?

¿Cuántas días toma... vitaminas en una semana?

	None	1-2 days	3-4 days	5-6 days	Everyday
7. Multivitamin/polyvisol	○	○	○	○	○
8. Trivisol	○	○	○	○	○
9. Calcium Supplement	○	○	○	○	○
10. Pediasure/Ensure	○	○	○	○	○
11. Other	○	○	○	○	○

Other specify: ______________________________

7a. Does the multivitamin contain extra iron?

¿Contiene hierro adicional?

○ Yes
○ No
○ Unsure

7b. Does the multivitamin contain extra calcium?

¿Contiene calcio adicional?

○ Yes
○ No
○ Unsure

12a. At present, how often does your child eat the following foods per week?

¿Ahora, me diré una lista de comidas u Ud. Me dirá cuántas veces por semana... las come? Cuantos días por semana come...

	None	< 1 day	1-2 days	3-5 days	6-7 days	Unsure
Cow's milk/Dairy Products/Cheese / Leche, queso, productos lactos	○	○	○	○	○	○
Eggs / Huevos	○	○	○	○	○	○

Peanut (Including peanut butter) / Cacahuete/ maní (Incluyendo crema/mantequilla de maní)	○	○	○	○	○	○
Tree Nuts (ie almond, cashew, filbert/hazel, walnut, brazil, macadamia, pecan, pine, pistachio) / Nueces de árbol	○	○	○	○	○	○
Fish (ie salmon, tuna, catfish, cod, flounder, halibut, trout, bass) / Pescado	○	○	○	○	○	○
Shellfish (ie shrimp, crab, lobster, clam, oyster, mussels) / Mariscos	○	○	○	○	○	○
Wheat (ie pasta, bread, cereal) / Productos de trigo	○	○	○	○	○	○
Soy/Tofu / Soja/tofu	○	○	○	○	○	○
Seeds (ie sesame, sunflower, pumpkin) / Semilla (sésamo)	○	○	○	○	○	○
Green vegetables / Verduras verdes	○	○	○	○	○	○
Orange veggies (carrots, squash, etc) / Verduras naranjas	○	○	○	○	○	○
Fruits / Frutas	○	○	○	○	○	○
Fruit Juice (without calcium) / jugo de fruta (sin calcio)	○	○	○	○	○	○
Calcium-fortified Juice / jugo de fruta (con calcio)	○	○	○	○	○	○
Meats / Carne	○	○	○	○	○	○
Beans / Frijoles	○	○	○	○	○	○
Rice / Arroz	○	○	○	○	○	○

12b. At present, how often does your child eat breakfast per week?

¿Cuántas días por semana come el desayuno?

- ○ None
- ○ < 1 day
- ○ 1-2 days
- ○ 3-5 days
- ○ 6-7 days
- ○ Unsure

---

13. What is your child's current eczema status?
¿Ha tenido eccema?

- ○ Current
- ○ Outgrown since last visit
- ○ Never had it
- ○ Don't know

14. Do you currently use cream, lotion, or ointment containing steroids on your child's skin for eczema? (for example: hydrocortisone cream or triamcinolone cream)<br><br>¿Ud. Usa una crema que tiene esteroides (como hidrocortisona) en el piel de...?	◯ Yes<br>◯ No<br>◯ Unsure
15. Does your child have hay fever or seasonal allergies?<br><br>¿Tiene alergias estacionales?	◯ Yes, he/she has it now<br>◯ No<br>◯ Unsure
15b. Which season does your child have seasonal allergies? (select all that apply)<br><br>Primavera<br>Verano<br>Otoño<br>Invierno<br>Todo el año	☐ Spring<br>☐ Summer<br>☐ Autumn<br>☐ Winter<br>☐ Year round
16. Does your child have pet allergies?<br><br>¿Tiene algunas alergias a animales o mascotas?	◯ Yes<br>◯ No<br>◯ Don't Know
If YES, what type of pet allergy? (select all that apply)	☐ Cat<br>☐ Dog
If OTHER, specify:	________________________<br>(name of other type of pet that child is allergic to)
If OTHER, specify:	________________________<br>(name of other type of pet that child is allergic to)

17. Has your child been diagnosed by a doctor with any of the follow environmental allergies?

¿Ha diagnosticado... con una alergia de...?

	Current	Outgrown since last visit	Never	Don't know
Polleen(tree, grass, ragweed) / polen	◯	◯	◯	◯
Dustmite / Polvo	◯	◯	◯	◯
Cockroach / Cucaracha	◯	◯	◯	◯
Mold / Moho	◯	◯	◯	◯
Other 1	◯	◯	◯	◯

Other 2	◯	◯	◯	◯
Other 3	◯	◯	◯	◯

Other allergy 1 specify ______________________

Other allergy 2 specify ______________________

Other allergy 3 specify ______________________

18. Has your child ever used anti-allergy medication? (ie Benadryl, Zyrtec, Claritin, Atarax, Dimetapp)

¿En el último año, ha usado medicina anti alergia?

◯ Yes
◯ No
◯ Don't Know

19. Has your child ever used medications for gastrointestinal upset?

¿En el último año, ha usado medicina por el dolor de estómago?

◯ Yes
◯ No
◯ Don't Know

if YES, which of the following medications did he/she take?

¿Qué tipo?

◯ Antacids (Mylants, Rolaids, TUMS, Pepto-Bismol)
◯ H2 Blockers
◯ Proton Pump inhibitors (Aciphex, Prilosec, Preveacid, Nexium)
◯ Prokinetic agents (Urecholine, Reglin, Erythromycin)
◯ Unsure
◯ Other

If Others, specify: ______________________

20. Does your child have any drug allergies?

¿Tiene alergias a algunas medicinas o drogas?

◯ Yes
◯ No
◯ Don't Know

If yes, specify the drug (use "," to separate): ______________________

If yes, specify the drug (use "," to separate): ______________________

21. Is your child allergic to insect stings?

¿Tiene alergias a algunos insectos?
¿Ha sido picado por una abeja o auispa?

◯ Yes
◯ No
◯ Don't know/Child has never been stung

22. Has your child ever had E.E. (Eosinophilic esophagitis)?

¿Tiene ... esofagitis eosinofilica?

◯ No
◯ Yes, only when she/he was a baby, but outgrew by age
◯ Yes, he/she has it now
◯ Don't know

If outgrown, at what age did your child outgrow?<br>Year(s)	______________________<br>(Years)
If outgrown, at what age did your child outgrow?<br>Months	______________________<br>(Months)
If yes, was your child's EE diagnosed by a doctor?<br><br>¿Fue diagnosticado por un doctor?	◯ Yes<br>◯ No<br>◯ Don't know
How old was your child when first diagnosed by a doctor?<br>Year(s)<br>¿Cuándo fue diagnosticado?<br>Años	______________________<br>(Years / Años)
How old was your child when first diagnosed by a doctor?<br>Months<br><br>¿Cuándo fue diagnosticado?<br>Meses	______________________<br>(months / Meses)
23. Has your child ever had GERD (Gastroesophageal Reflux Disease)?<br><br>¿Ha tenido reflujo de ácida?	◯ No<br>◯ Yes, only when she/he was a baby, but outgrew<br>◯ Yes, he/she has it now<br>◯ Don't know
If outgrown, at what age?<br>Year(s)	______________________<br>(years)
If outgrown, at what age?<br>Months	______________________<br>(Months)
If yes, was your child's GERD diagnosed by a doctor?<br><br>¿Fue diagnosticado por un doctor?	◯ Yes<br>◯ No<br>◯ Don't know
How old was your child when first diagnosed by a doctor?<br>Year(s)<br><br>¿Cuándo fue diagnosticado?<br>Años	______________________<br>(years)
How old was your child when first diagnosed by a doctor?<br>Months<br><br>¿Cuándo fue diagnosticado?<br>Meses	______________________<br>(months / Meses)

24. What is your child's food allergy status (meaning any food)?<br><br>¿Ha tenido... alergias a algunas comidas? ¿Ahora tiene?	◯ Current<br>◯ Outgrown since last visit<br>◯ Food was never introduced due to positive skin test or RAST<br>◯ Never

If NEVER skip to PEDIATRIC SLEEP QUESTIONNAIRE

24b. Allergy to Dairy products / Cheese / Milk?<br><br>Leche, queso, productos lactos	◯ Current<br>◯ Outgrown since last visit<br>◯ Food was never introduced due to positive skin test or RAST<br>◯ Never
Allergy to Egg?<br><br>Huevos	◯ Current<br>◯ Outgrown since last visit<br>◯ Food was never introduced due to positive skin test or RAST<br>◯ Never
Allergy to Peanuts?<br><br>Cacahuete/ maní (Incluyendo crema/mantequilla de maní)	◯ Current<br>◯ Outgrown since last visit<br>◯ Food was never introduced due to positive skin test or RAST<br>◯ Never
Allergy to Tree Nuts<br><br>Nueces de árbol	◯ Current<br>◯ Outgrown since last visit<br>◯ Food was never introduced due to positive skin test or RAST<br>◯ Never
Allergy to Fish?<br><br>Pescado	◯ Current<br>◯ Outgrown since last visit<br>◯ Food was never introduced due to positive skin test or RAST<br>◯ Never
Allergy to Shellfish?<br><br>Mariscos	◯ Current<br>◯ Outgrown since last visit<br>◯ Food was never introduced due to positive skin test or RAST<br>◯ Never
Allergy to Wheat?<br><br>Productos de trigo	◯ Current<br>◯ Outgrown since last visit<br>◯ Food was never introduced due to positive skin test or RAST<br>◯ Never
Allergy to Soy/Tofu?<br><br>Soja/tofu	◯ Current<br>◯ Outgrown since last visit<br>◯ Food was never introduced due to positive skin test or RAST<br>◯ Never

Allergy to Seeds?<br><br>Semillas	◯ Current<br>◯ Outgrown since last visit<br>◯ Food was never introduced due to positive skin test or RAST<br>◯ Never
Specify Other Food Allergy #1:	__________________________________<br>(name of other food #1 child is allergic to)
Other Food Allergy #1	◯ Current<br>◯ Outgrown since last visit<br>◯ Food was never introduced due to positive skin test or RAST<br>◯ Never
Specify Other Food Allergy #2:	__________________________________<br>(name of other food #2 child is allergic to)
Other Food Allergy #2	◯ Current<br>◯ Outgrown since last visit<br>◯ Food was never introduced due to positive skin test or RAST<br>◯ Never
Specify Other Food Allergy #3:	__________________________________<br>(name of other food #3 child is allergic to)
Other Food Allergy #3	◯ Current<br>◯ Outgrown since last visit<br>◯ Food was never introduced due to positive skin test or RAST<br>◯ Never
Specify Other Food Allergy #4:	__________________________________<br>(name of other food #4 child is allergic to)
Other Food Allergy #4	◯ Current<br>◯ Outgrown since last visit<br>◯ Food was never introduced due to positive skin test or RAST<br>◯ Never
Specify Other Food Allergy #5:	__________________________________<br>(name of other food #5 child is allergic to)
Other Food Allergy #5	◯ Current<br>◯ Outgrown since last visit<br>◯ Food was never introduced due to positive skin test or RAST<br>◯ Never

Specify Other Food Allergy #6: ________________________________
(name of other food #6 child is allergic to)

Other Food Allergy #6
- ◯ Current
- ◯ Outgrown since last visit
- ◯ Food was never introduced due to positive skin test or RAST
- ◯ Never

25a. Has your child been breast fed since the last visit?

¿Sido alimentado con leche materna...en el último año?
- ◯ Yes
- ◯ No

Skip to question 26 if child has not been breast fed since the last visit

25b. Since the last visit, has your child ever experienced allergic symptoms to any food that was passed exclusively through breast milk?

¿En el año pasado ha experimentado... sin toma alérgica a los alimentos pasados a través de la leche materna?
- ◯ Yes
- ◯ No
- ◯ Don't know

If yes, to which foods?
- ☐ Dairy products/Cheese/Milk
- ☐ Egg
- ☐ Peanuts
- ☐ Tree Nuts
- ☐ Fish
- ☐ Shellfish
- ☐ Wheat
- ☐ Soy/Tofu
- ☐ Seeds
- ☐ Other

If other, list other foods:
Other food #1 ________________________________

If other, list other foods:
Other food #2 ________________________________

If other, list other foods:
Other food #3 ________________________________

If other, list other foods:
Other food #4 ________________________________

If other, list other foods:
Other food #5 ________________________________

If other, list other foods:
Other food #6 ________________________________

26. Has your child experienced any of the following symptoms from ingestion since the last visit?

¿Ha experimentado su hijo alguno de los siguientes síntomas por ingestión en el último año?

26a. Any mouth symptoms<br><br>Síntomas de boca	◯ Yes<br>◯ No<br>◯ Don't know

26a. Specific symptoms of food allergy (through ingestion):
a. MOUTH (Boca)
Cow's Milk/Dairy Products/Cheese

	Check box if yes
Lips Itching/Tingling / Picazón en los labios	☐
Lips Swelling / Labios hinchados	☐
Tongue Itching/Tingling / Picazón la lengua	☐
Tongue Swelling / Lengua hinchada	☐

26 a. Specific symptoms of food allergy (through ingestion):
a. MOUTH
Egg

	Check box if yes
Lips Itching/Tingling	☐
Lips Swelling	☐
Tongue Itching/Tingling	☐
Tongue Swelling	☐

26a. Specific symptoms of food allergy (through ingestion):
a. MOUTH
Peanut

	Check box if yes
Lips Itching/Tinging	☐
Lips Swelling	☐
Tongue Itching/Tingling	☐
Tongue Swelling	☐

26a. Specific symptoms of food allergy (through ingestion):
a. MOUTH
Tree Nuts

	Check box if yes
Lips Itching/Tingling	☐
Lips Swelling	☐
Tongue Itching/Tingling	☐
Tongue Swelling	☐

26a. Specific symptoms of food allergy (through ingestion):
a. MOUTH
Fish

	Check box if yes
Lips Itching/Tingling	☐
Lips Swelling	☐
Tongue Itching/Tingling	☐
Tongue Swelling	☐

26a. Specific symptoms of food allergy (through ingestion):
a. MOUTH
Shellfish

	Check box if yes
Lips Itching/Tingling	☐
Lips Swelling	☐
Tongue Itching/Tingling	☐
Tongue Swelling	☐

26. Specific symptoms of food allergy (through ingestion):
a. MOUTH
Wheat

	Check box if yes
Lips Itching/Tingling	☐
Lips Swelling	☐
Tongue Itching/Tingling	☐
Tongue Swelling	☐

26. Specific symptoms of food allergy (through ingestion):
a. MOUTH
Soy/Tofu

	Check box if yes
Lips Itching/Tingling	☐
Lips Swelling	☐

Tongue Itching/Tingling	☐
Tongue Swelling	☐

26. Specific symptoms of food allergy (through ingestion):
a. MOUTH
Seeds

	Check box if yes
Lips Itching/Tingling	☐
Lips Swelling	☐
Tongue Itching/Tingling	☐
Tongue Swelling	☐

26a. Name of Other Food Allergy #1 ________________________________

26. Specific symptoms of food allergy (through ingestion):
a. MOUTH
Other Food Allergy #1

	Check box if yes
Lips Itching/Tingling	☐
Lips Swelling	☐
Tongue Itching/Tingling	☐
Tongue Swelling	☐

26a. Name of Other Food Allergy #2 ________________________________

26. Specific symptoms of food allergy (through ingestion):
a. MOUTH
Other Food Allergy #2

	Check box if yes
Lips Itching/Tingling	☐
Lips Swelling	☐
Tongue Itching/Tingling	☐
Tongue Swelling	☐

26a. Name of Other Food Allergy #3 ________________________________

26. Specific symptoms of food allergy (through ingestion):
a. MOUTH
Other Food Allergy #3

	Check box if yes
Lips Itching/Tingling	○
Lips Swelling	○
Tongue Itching/Tingling	○
Tongue Swelling	○

26a. Name of Other Food Allergy #4 ________________________

26. Specific symptoms of food allergy (through ingestion):
a. MOUTH
Other Food Allergy #4

	Check box if yes
Lips Itching/Tingling	☐
Lips Swelling	☐
Tongue Itching/Tingling	☐
Tongue Swelling	☐

26a. Name of Other Food Allergy #5 ________________________

26. Specific symptoms of food allergy (through ingestion):
a. MOUTH
Other Food Allergy #5

	Check box if yes
Lips Itching/Tingling	☐
Lips Swelling	☐
Tongue Itching/Tingling	☐
Toungue Swelling	☐

26a. Name of Other Food Allergy #6 ________________________

26. Specific symptoms of food allergy (through ingestion):
a. MOUTH
Other Food Allergy #6

	Check box if yes
Lips Itching/Tingling	○

Lips Swelling	◯
Tongue Itching/Tingling	◯
Tongue Swelling	◯

26 b/c. Eye or nose symptoms

Síntomas de ojos/ nariz

◯ Yes
◯ No
◯ Don't know

26. Specific symptoms of food allergy (through ingestion):
b/c. EYE/NOSE
Cow's Milk/Dairy Products/Cheese

	Check box if yes
Red/Watery/Itchy Eye / Ojo rojo/pica	☐
Swollen Eye / Ojo hinchado	☐
Stuffy/Runny Nose / Congestión nasal	☐
Sneezing / Estornudo	☐
Itchy Nose / Picazón en la nariz	☐

26. Specific symptoms of food allergy (through ingestion):
b/c. EYE/NOSE
Egg

	Check box if yes
Red/Watery/Itchy Eye	☐
Swollen Eye	☐
Stuffy/Runny Nose	☐
Sneezing	☐
Itchy Nose	☐

26. Specific symptoms of food allergy (through ingestion):
b/c. EYE/NOSE
Peanut

	Check box if yes
Red/Watery/Itchy Eye	☐
Swollen Eye	☐
Stuffy/Runny Nose	☐
Sneezing	☐
Itchy Nose	☐

26. Specific symptoms of food allergy (through ingestion):
b/c. EYE/NOSE
Tree Nuts

	Check box if yes
Red/Watery/Itchy Eye	☐
Swollen Eye	☐
Stuffy/Runny Nose	☐
Sneezing	☐
Itchy Nose	☐

26. Specific symptoms of food allergy (through ingestion):
b/c. EYE/NOSE
Fish

	Check box if yes
Red/Watery/Itchy Eye	☐
Swollen Eye	☐
Stuffy/Runny Nose	☐
Sneezing	☐
Itchy Nose	☐

26. Specific symptoms of food allergy (through ingestion):
b/c. EYE/NOSE
Shellfish

	Check box if yes
Red/Watery/Itchy Eye	☐
Swollen Eye	☐
Stuffy/Runny Nose	☐
Sneezing	☐
Itchy Nose	☐

26. Specific symptoms of food allergy (through ingestion):
b/c. EYE/NOSE
Wheat

	Check box if yes
Red/Watery/Itchy Eye	☐
Swollen Eye	☐
Stuffy/Runny Nose	☐
Sneezing	☐
Itchy Nose	☐

26. Specific symptoms of food allergy (through ingestion):
b/c. EYE/NOSE
Soy/Tofu

	Check box if yes
Red/Watery/Itchy Eye	☐
Swollen Eye	☐
Stuffy/Runny Nose	☐
Sneezing	☐
Itchy Nose	☐

26. Specific symptoms of food allergy (through ingestion):
b/c. EYE/NOSE
Seeds

	Check box if yes
Red/Watery/Itchy Eye	☐
Swollen Eye	☐
Stuffy/Runny Nose	☐
Sneezing	☐
Itchy Nose	☐

26b/c. Name of Other Food Allergy #1 ______________________________

26. Specific symptoms of food allergy (through ingestion):
b/c. EYE/NOSE
Other Food Allergy #1

	Check box if yes
Red/Watery/Itchy Eye	☐
Swollen Eye	☐
Stuffy/Runny Nose	☐
Sneezing	☐
Itchy Nose	☐

26b/c. Name of Other Food Allergy #2 ______________________________

26. Specific symptoms of food allergy (through ingestion):
b/c. EYE/NOSE
Other Food Allergy #2

	Check box if yes
Red/Watery/Itchy Eye	☐

Swollen Eye	☐
Stuffy/Runny Nose	☐
Sneezing	☐
Itchy Nose	☐

26b/c. Name of Other Food Allergy #3 __________________________________

26. Specific symptoms of food allergy (through ingestion):
b/c. EYE/NOSE
Other Food Allergy #3

	Check box if yes
Red/Watery/Itchy Eye	◯
Swollen Eye	◯
Stuffy/Runny Nose	◯
Sneezing	◯
Itchy Nose	◯

26b/c. Name of Other Food Allergy #4 __________________________________

26. Specific symptoms of food allergy (through ingestion):
b/c. EYE/NOSE
Other Food Allergy #4

	Check box if yes
Red/Watery/Itchy Eye	☐
Swollen Eye	☐
Stuffy/Runny Nose	☐
Sneezing	☐
Itchy Nose	☐

26b/c. Name of Other Food Allergy #5 __________________________________

26. Specific symptoms of food allergy (through ingestion):
b/c. EYE/NOSE
Other Food Allergy #5

	Check box if yes
Red/Watery/Itchy Eye	◯
Swollen Eye	◯
Stuffy/Runny Nose	◯

Sneezing ◯
Itchy Nose ◯

26b/c. Name of Other Food Allergy #6 ____________________

26. Specific symptoms of food allergy (through ingestion):
b/c. EYE/NOSE
Other Food Allergy #6

Check box if yes

Red/Watery/Itchy Eye ☐
Swollen Eye ☐
Stuffy/Runny Nose ☐
Sneezing ☐
Itchy Nose ☐

26d. Throat symptoms

Síntomas de la garganta

◯ Yes
◯ No
◯ Don't know

26. Specific symptoms of food allergy (through ingestion):
d. THROAT
Cow's Milk/Dairy Products/Cheese

Check box if yes

Itching and/or tightness in the throat / Picazón u opresión en la garganta ☐

Hoarseness/change of voice / Voz ronco ☐

Choking/Difficulty Swallowing / Dificultad para deglutir ☐

Throat Clearing / Limpiado de la garganta ☐

26. Specific symptoms of food allergy (through ingestion):
d. THROAT
Egg

Check box if yes

Itching and/or tightness in the throat ◯

Hoarseness/change of voice ◯

Choking/Difficulty Swallowing ◯

Throat Clearing ○

26. Specific symptoms of food allergy (through ingestion):
d. THROAT
Peanut

	Check box if yes
Itching and/or tightness in the throat	☐
Hoarseness/change of voice	☐
Choking/Difficulty swallowing	☐
Throat Clearing	☐

26. Specific symptoms of food allergy (through ingestion):
d. THROAT
Tree Nuts

	Check box if yes
Itching and/or tightness in the throat	☐
Hoarseness/change of voice	☐
Choking/Difficulty Swallowing	☐
Throat Clearing	☐

26. Specific symptoms of food allergy (through ingestion):
d. THROAT
Fish

	Check box if yes
Itching and/or tightness in the throat	☐
Hoarseness/change of voice	☐
Choking/Difficulty Swallowing	☐
Throat Clearing	☐

26. Specific symptoms of food allergy (through ingestion):
d. THROAT
Shellfish

	Check box if yes
Itching and/or tightness in the throat	☐
Hoarseness/change of voice	☐
Choking/Difficulty Swallowing	☐
Throat Clearing	☐

26. Specific symptoms of food allergy (through ingestion):
d. THROAT
Wheat

	Check box if yes
Itching and/or tightness in the throat	☐
Hoarseness/change of voice	☐
Choking/Difficulty Swallowing	☐
Throat Clearing	☐

26. Specific symptoms of food allergy (through ingestion):
d. THROAT
Soy/Tofu

	Check box if yes
Itching and/or tightness in the throat	☐
Hoarseness/change of voice	☐
Choking/Difficulty Swallowing	☐
Throat Clearing	☐

26. Specific symptoms of food allergy (through ingestion):
d. THROAT
Seeds

	Check box if yes
Itching and/or tightness in the throat	☐
Hoarseness/change of voice	☐
Choking/Difficulty Swallowing	☐
Throat Clearing	☐

26d. Name of Other Food Allergy #1 ________________________________

26. Specific symptoms of food allergy (through ingestion):
d. THROAT
Other Food Allergy #1

	Check box if yes
Itching and/or tightness in the throat	☐
Hoarseness/change of voice	☐
Choking/Difficulty Swallowing	☐
Throat Clearing	☐

26d. Name of Other Food Allergy #2 ________________________________

26. Specific symptoms of food allergy (through ingestion):
d. THROAT
Other Food Allergy #2

	Check box if yes
Itching and/or tightness in the throat	◯
Hoarseness/change of voice	◯
Choking/Difficulty Swallowing	◯
Throat Clearing	◯

26d. Name of Other Food Allergy #3 ______________________

26. Specific symptoms of food allergy (through ingestion):
d. THROAT
Other Food Allergy #3

	Check box if yes
Itching and/or tightness in the throat	◯
Hoarseness/change of voice	◯
Choking/Difficulty Swallowing	◯
Throat Clearing	◯

26d. Name of Other Food Allergy #4 ______________________

26. Specific symptoms of food allergy (through ingestion):
d. THROAT
Other Food Allergy #4

	Check box if yes
Itching and/or tightness in the throat	☐
Hoarseness/change of voice	☐
Choking/Difficulty Swallowing	☐
Throat Clearing	☐

26d. Name of Other Food Allergy #5 ______________________

26. Specific symptoms of food allergy (through ingestion):
d. THROAT
Other Food Allergy #5

	Check box if yes
Itching and/or tightness in the throat	☐
Hoarseness/change of voice	☐
Choking/Difficulty Swallowing	☐
Throat Clearing	☐

26d. Name of Other Food Allergy #6 ________________________________

26. Specific symptoms of food allergy (through ingestion):
d. THROAT
Other Food Allergy #6

	Check box if yes
Itching and/or tightness in the throat	☐
Hoarseness/change of voice	☐
Choking/Difficulty Swallowing	☐
Throat Clearing	☐

26e. Skin symptoms

Síntomas de piel

◯ Yes
◯ No
◯ Don't know

26. Specific symptoms of food allergy (through ingestion):
e. SKIN
Cow's Milk/Dairy Products/Cheese

	Check box if yes
Itching / Pica	☐
Hives / Urticaria	☐
Swelling of the face and/or extremeties / Hinchazón de la cara o extremidades	☐
Redness of the skin / Piel rojo	☐

26. Specific symptoms of food allergy (through ingestion):
e. SKIN
Egg

	Check box if yes
Itching	☐
Hives	☐
Swelling of the face and/or extremeties	☐
Redness of the skin	☐

26. Specific symptoms of food allergy (through ingestion):
e. SKIN
Peanut

	Check box if yes
Itching	☐
Hives	☐
Swelling of the face and/or extremeties	☐
Redness of the skin	☐

26. Specific symptoms of food allergy (through ingestion):
e. SKIN
Treenut

	Check box if yes
Itching	☐
Hives	☐
Swelling of the face and/or extremeties	☐
Redness of the skin	☐

26. Specific symptoms of food allergy (through ingestion):
e. SKIN
Fish

	Check box if yes
Itching	☐
Hives	☐
Swelling of the face and/or extremeties	☐
Redness of the skin	☐

26. Specific symptoms of food allergy (through ingestion):
e. SKIN
Shellfish

	Check box if yes
Itching	☐
Hives	☐
Swelling of the face and/or extremeties	☐
Redness of the skin	☐

26. Specific symptoms of food allergy (through ingestion):
e. SKIN
Wheat

	Check box if yes
Itching	☐
Hives	☐
Swelling of the face and/or extremeties	☐
Redness of the skin	☐

26. Specific symptoms of food allergy (through ingestion):
e. SKIN
Soy/Tofu

	Check box if yes
Itching	☐
Hives	☐
Swelling of the face and/or extremeties	☐
Redness of the skin	☐

26. Specific symptoms of food allergy (through ingestion):
e. SKIN
Seeds

	Check box if yes
Itching	☐
Hives	☐
Swelling of the face and/or extremeties	☐
Redness of the skin	☐

26e. Name of Other Food Allergy #1 ______________________

26. Specific symptoms of food allergy (through ingestion):
e. SKIN
Other Food Allergy #1

	Check box if yes
Itching	☐
Hives	☐
Swelling of the face and/or extremeties	☐
Redness of the skin	☐

26e. Name of Other Food Allergy #2 ________________________

26. Specific symptoms of food allergy (through ingestion):
e. SKIN
Other Food Allergy #2

	Check box if yes
Itching	☐
Hives	☐
Swelling of the face and/or extremeties	☐
Redness of the skin	☐

26e. Name of Other Food Allergy #3 ________________________

26. Specific symptoms of food allergy (through ingestion):
e. SKIN
Other Food Allergy #3

	Check box if yes
Itching	☐
Hives	☐
Swelling of the face and/or extremities	☐
Redness of the skin	☐

26e. Name of Other Food Allergy #4 ________________________

26. Specific symptoms of food allergy (through ingestion):
e. SKIN
Other Food Allergy #4

	Check box if yes
Itching	☐
Hives	☐
Swelling of the face and/or extremities	☐
Redness of the skin	☐

26e. Name of Other Food Allergy #5 ________________________

26. Specific symptoms of food allergy (through ingestion):
e. SKIN
Other Food Allergy #5

	Check box if yes
Itching	☐
Hives	☐
Swelling of the face and/or extremities	☐
Redness of the skin	☐

26e. Name of Other Food Allergy #6 ________________________

26. Specific symptoms of food allergy (through ingestion):
e. SKIN
Other Food Allergy #6

	Check box if yes
Itching	☐
Hives	☐
Swelling of the face and/or extremities	☐
Redness of the skin	☐

26f. Lung Symptoms

Síntomas de Pulmón

◯ Yes
◯ No
◯ Don't know

26. Specific symptoms of food allergy (through ingestion):
f. LUNG
Cow's Milk/Dairy Products/Cheese

	Check box if yes
Shortness of breath / Falta de aliento	☐
Repetitive coughing / Tos repetitiva	☐
Wheezing / Aliento ruidoso	☐
Chest Tightness/Opresión en el pecho	☐

26. Specific symptoms of food allergy (through ingestion):
f. LUNG
Egg

	Check box if yes
Shortness of breath	☐
Repetitive coughing	☐
Wheezing	☐
Chest Tightness	☐

26. Specific symptoms of food allergy (through ingestion):
f. LUNG
Peanut

	Check box if yes
Shortness of breath	☐
Repetitive coughing	☐
Wheezing	☐
Chest Tightness	☐

26. Specific symptoms of food allergy (through ingestion):
f. LUNG
Treenut

	Check box if yes
Shortness of Breath	☐
Repetitive Coughing	☐
Wheezing	☐
Chest Tightness	☐

26. Specific symptoms of food allergy (through ingestion):
f. LUNG
Fish

	Check box if yes
Shortness of breath	☐
Repetitive coughing	☐
Wheezing	☐
Chest Tightness	☐

26. Specific symptoms of food allergy (through ingestion):
f. LUNG
Shellfish

	Check box if yes
Shortness of breath	☐
Repetitive coughing	☐
Wheezing	☐
Chest Tightness	☐

26. Specific symptoms of food allergy (through ingestion):
f. LUNG
Wheat

	Check box if yes
Shortness of breath	☐
Repetitive coughing	☐
Wheezing	☐
Chest Tightness	☐

26. Specific symptoms of food allergy (through ingestion):
f. LUNG
Soy/Tofu

	Check box if yes
Shortness of breath	☐
Repetitive coughing	☐
Wheezing	☐
Chest Tightness	☐

26. Specific symptoms of food allergy (through ingestion):
f. LUNG
Seeds

	Check box if yes
Shortness of breath	☐
Repetitive coughing	☐

Wheezing ☐
Chest Tightness ☐

26f. Name of Other Food Allergy #1 ______________________

26. Specific symptoms of food allergy (through ingestion):
f. LUNG
Other Food Allergy #1

	Check box if yes
Shortness of breath	☐
Repetitive coughing	☐
Wheezing	☐
Chest Tightness	☐

26f. Name of Other Food Allergy #2 ______________________

26. Specific symptoms of food allergy (through ingestion):
f. LUNG
Other Food Allergy #2

	Check box if yes
Shortness of breath	☐
Repetitive coughing	☐
Wheezing	☐
Chest Tighness	☐

26f. Name of Other Food Allergy #3 ______________________

26. Specific symptoms of food allergy (through ingestion):
f. LUNG
Other Food Allergy #3

	Check box if yes
Shortness of breath	☐
Repetitive coughing	☐
Wheezing	☐
Chest Tightness	☐

26f. Name of Other Food Allergy #4 ______________________

26. Specific symptoms of food allergy (through ingestion):
f. LUNG
Other Food Allergy #4

	Check box if yes
Shortness of breath	☐
Repetitive coughing	☐
Wheezing	☐
Chest Tightness	☐

26f. Name of Other Food Allergy #5 ______________________________

26. Specific symptoms of food allergy (through ingestion):
f. LUNG
Other Food Allergy #5

	Check box if yes
Shortness of breath	☐
Repetitive coughing	☐
Wheezing	☐
Chest Tightness	☐

26f. Name of Other Food Allergy #6 ______________________________

34. Specific symptoms of food allergy (through ingestion):
f. LUNG
Other Food Allergy #6

	Check box if yes
Shortness of breath	☐
Repetitive coughing	☐
Wheezing	☐
Chest Tightness	☐

26g. Gut symptoms

Síntomas de tripa / intestino

◯ Yes
◯ No
◯ Don't know

26. Specific symptoms of food allergy (through ingestion):
g. GUT
Cow's Milk/Dairy Products/Cheese

Check box if yes

Stomach cramps/pain / Dolor de estómago	☐
Nausea / Náusea	☐
Vomiting / Vómito	☐
Diarrhea / Diarrea	☐
Bloating (swelling, gassy feeling) / Estómago hinchado	☐

26. Specific symptoms of food allergy (through ingestion):
g. GUT
Egg

	Check box if yes
Stomach cramps/pain	☐
Nausea	☐
Vomiting	☐
Diarrhea	☐
Bloating (swelling, gassy feeling)	☐

26. Specific symptoms of food allergy (through ingestion):
g. GUT
Peanut

	Check box if yes
Stomach cramps/pain	☐
Nausea	☐
Vomiting	☐
Diarrhea	☐
Bloating (swelling, gassy feeling)	☐

26. Specific symptoms of food allergy (through ingestion):
g. GUT
Tree Nuts

	Check box if yes
Stomach cramps/pain	☐
Nausea	☐
Vomiting	☐
Diarrhea	☐
Bloating (swelling, gassy feeling)	☐

26. Specific symptoms of food allergy (through ingestion):
g. GUT
Fish

	Check box if yes
Stomach cramps/pain	☐
Nausea	☐
Vomiting	☐
Diarrhea	☐
Bloating (swelling, gassy feeling)	☐

26. Specific symptoms of food allergy (through ingestion):
g. GUT
Shellfish

	Check box if yes
Stomach cramps/pain	☐
Nausea	☐
Vomiting	☐
Diarrhea	☐
Bloating (swelling, gassy feeling)	☐

26. Specific symptoms of food allergy (through ingestion):
g. GUT
Wheat

	Check box if yes
Stomach cramps/pain	☐
Nausea	☐
Vomiting	☐
Diarrhea	☐
Blaoting (swelling, gassy feeling)	☐

26. Specific symptoms of food allergy (through ingestion):
g. GUT
Soy/Tofu

	Check box if yes
Stomach cramps/pain	☐
Nausea	☐
Vomiting	☐
Diarrhea	☐
Bloating (swelling, gassy feeling)	☐

26. Specific symptoms of food allergy (through ingestion):
g. GUT
Seeds

	Check box if yes
Stomach cramps/pain	☐
Nausea	☐
Vomiting	☐
Diarrhea	☐
Bloating (swelling, gassy feeling)	☐

26g. Name of Other Food Allergy #1 __________________________________

26. Specific symptoms of food allergy (through ingestion):
g. GUT
Other Food Allergy #1

	Check box if yes
Stomach cramps/pain	☐
Nausea	☐
Vomiting	☐
Diarrhea	☐
Bloating (swelling, gassy feeling)	☐

26g. Name of Other Food Allergy #2 __________________________________

26. Specific symptoms of food allergy (through ingestion):
g. GUT
Other Food Allergy #2

	Check box if yes
Stomach cramps/pain	☐
Nausea	☐
Vomiting	☐
Diarrhea	☐
Bloating (swelling, gassy feeling)	☐

26g. Name of Other Food Allergy #3 __________________________________

26. Specific symptoms of food allergy (through ingestion):
g. GUT
Other Food Allergy #3

	Check box if yes
Stomach cramps/pain	☐
Nausea	☐
Vomiting	☐
Diarrhea	☐
Bloating (swelling, gassy feeling)	☐

26g. Name of Other Food Allergy #4 __________________________________

26. Specific symptoms of food allergy (through ingestion):
g. GUT
Other Food Allergy #4

	Check box if yes
Stomach cramps/pain	☐
Nausea	☐
Vomiting	☐
Diarrhea	☐
Bloating (swelling, gassy feeling)	☐

26g. Name of Other Food Allergy #5 __________________________________

26. Specific symptoms of food allergy (through ingestion):
g. GUT
Other Food Allergy #5

	Check box if yes
Stomach cramps/pain	☐
Nausea	☐
Vomiting	☐
Diarrhea	☐
Bloating (swelling, gassy feeling)	☐

26g. Name of Other Food Allergy #6 __________________________________

26. Specific symptoms of food allergy (through ingestion):
g. GUT
Other Food Allergy #6

	Check box if yes
Stomach cramps/pain	☐
Nausea	☐
Vomiting	☐
Diarrhea	☐
Bloating (swelling, gassy feeling)	☐

26h. Cardiovascular symptoms

Síntomas de cardiovascular

◯ Yes
◯ No
◯ Don't know

26. Specific symptoms of food allergy (through ingestion):
h. CARDIOVACULAR
Cow's Milk/Dairy Products/Cheese

	Check box if yes
Pale or turn blue / Piel pálida o azul	☐
Dizzy/Light-headed / Marceo	☐
Passing out/Fainting / Desmogo	☐

26. Specific symptoms of food allergy (through ingestion):
h. CARDIOVACULAR
Egg

	Check box if yes
Pale or turn blue	☐
Dizzy/Light-headed	☐
Passing out/Fainting	☐

26. Specific symptoms of food allergy (through ingestion):
h. CARDIOVACULAR
Peanut

	Check box if yes
Pale or turn blue	☐
Dizzy/Light-headed	☐
Passing out/Fainting	☐

26. Specific symptoms of food allergy (through ingestion):
h. CARDIOVACULAR
Treenut

	Check box if yes
Pale or turn blue	☐
Dizzy/Light-headed	☐
Passing out/Fainting	☐

26. Specific symptoms of food allergy (through ingestion):
h. CARDIOVACULAR
Fish

	Check box if yes
Pale or turn blue	☐
Dizzy/Light-headed	☐
Passing out/Fainting	☐

26. Specific symptoms of food allergy (through ingestion):
h. CARDIOVACULAR
Shellfish

	Check box if yes
Pale or turn blue	☐
Dizzy/Light-headed	☐
Passing out/Fainting	☐

26. Specific symptoms of food allergy (through ingestion):
h. CARDIOVACULAR
Wheat

	Check box if yes
Pale or turn blue	☐
Dizzy/Light-headed	☐
Passing out/Fainting	☐

26. Specific symptoms of food allergy (through ingestion):
h. CARDIOVACULAR
Soy/Tofu

	Check box if yes
Pale or turn blue	☐
Dizzy/Light-headed	☐
Passing out/Fainting	☐

26. Specific symptoms of food allergy (through ingestion):
h. CARDIOVACULAR
Seeds

	Check box if yes
Pale or turn blue	☐
Dizzy/Light-headed	☐
Passing out/Fainting	☐

26h. Name of Other Food Allergy #1 ________________________________

26. Specific symptoms of food allergy (through ingestion):
h. CARDIOVACULAR
Other Food Allergy #1

	Check box if yes
Pale or turn blue	☐
Dizzy/Light-headed	☐
Passing out/Fainting	☐

26h. Name of Other Food Allergy #2 ________________________________

26. Specific symptoms of food allergy (through ingestion):
h. CARDIOVACULAR
Other Food Allergy #2

	Check box if yes
Pale or turn blue	☐
Dizzy/Light-headed	☐
Passing out/Fainting	☐

26h. Name of Other Food Allergy #3 ________________________________

26. Specific symptoms of food allergy (through ingestion):
h. CARDIOVACULAR
Other Food Allergy #3

	Check box if yes
Pale or turn blue	☐
Dizzy/Light-headed	☐
Passing out/Fainting	☐

26h. Name of Other Food Allergy #4 ________________________________

26. Specific symptoms of food allergy (through ingestion):
h. CARDIOVASCULAR
Other Food Allergy #4

	Check box if yes
Pale or turn blue	☐
Dizzy/Light-headed	☐
Passing out/Fainting	☐

26h. Name of Other Food Allergy #5 __________________________________

26. Specific symptoms of food allergy (through ingestion):
h. CARDIOVASCULAR
Other Food Allergy #5

	Check box if yes
Pale or turn blue	☐
Dizzy/Light-headed	☐
Passing out/Fainting	☐

26h. Name of Other Food Allergy #6 __________________________________

26. Specific symptoms of food allergy (through ingestion):
h. CARDIOVASCULAR
Other Food Allergy #6

	Check box if yes
Pale or turn blue	☐
Dizzy/Light-headed	☐
Passing out/Fainting	☐

27. Since the last visit, has your child ever experienced a severe allergic reaction that affect the throat, lungs, and/or cardiovascular system?

¿En el último año, ha experimentado una reacción alérgica grave que afecto la garganta, los pulmones o corazón?

IF NO SKIP TO QUESTION 28

- ◯ Yes
- ◯ No
- ◯ Don't know

If yes, to what foods? (select all that apply

	Yes, Doctor diagnosed	No, not doctor diagnosed
Cow's Mild/Dairy Products/Cheese	○	○
Egg	○	○
Peanut	○	○
Tree Nuts	○	○
Fish	○	○
Shellfish	○	○
Wheat	○	○
Soy/Tofu	○	○
Seeds	○	○
Other 1	○	○
Other 2	○	○
Other 3	○	○
Other 4	○	○
Other 5	○	○
Other 6	○	○

Number of episodes since last visit
Cow's Milk/Dairy Products/Cheese ________________________

Number of episodes since last visit
Eggs ________________________

Number of episodes since last visit
Peanut ________________________

Number of episodes since last visit
Tree Nuts ________________________

Number of episodes since last visit
Fish ________________________

Number of episodes since last visit
Shellfish ________________________

Number of episodes since last visit
Wheat ________________________

Number of episodes since last visit
Soy/tofu ________________________

Number of episodes since last visit
Seeds ________________________

IF other, specify:
Other 1 ________________________

Number of episodes since last visit
Other 1 ________________________

IF other, specify:
Other 2 __________________________________

Number of episodes since last visit
Other 2 __________________________________

IF other, specify:
Other 3 __________________________________

Number of episodes since last visit
Other 3 __________________________________

IF other, specify:
Other 4 __________________________________

Number of episodes since last visit
Other 4 __________________________________

IF other, specify:
Other 5 __________________________________

Number of episodes since last visit
Other 5 __________________________________

IF other, specify:
Other 6 __________________________________

Number of episodes since last visit
Other 6 __________________________________

28. For food that you child had an allergic reaction to since the last visit, how long does it usually take form eating the food to the onset of the allergic symptoms.?

¿Después de comer la comida, cuánto tiempo toma para los síntomas a aparecer?

28. How long does it usually take from eating the food to the onset of the allergic symptoms for COW'S MILK/DAIRY PRODUCTS/CHEESE? (Time until onset in DAYS) __________________________________ (number of days)

¿Después de comer la comida, cuánto tiempo toma para los síntomas a aparecer?

28. How long does it usually take from eating the food to the onset of the allergic symptoms for COW'S MILK/DAIRY PRODUCTS/CHEESE? (Time until onset in HOURS) __________________________________ (number of hours)

¿Después de comer la comida, cuánto tiempo toma para los síntomas a aparecer?

28. How long does it usually take from eating the food to the onset of the allergic symptoms for COW'S MILK/DAIRY PRODUCTS/CHEESE? (Time until onset in MINUTES)<br><br>¿Después de comer la comida, cuánto tiempo toma para los síntomas a aparecer?	______________________ (number of minutes)
28. How long does it usually take from eating the food to the onset of the allergic symptoms for EGGS? (Time until onset in DAYS)	______________________ (number of days)
28. How long does it usually take from eating the food to the onset of the allergic symptoms for EGGS? (Time until onset in HOURS)	______________________ (number of hours)
28. How long does it usually take from eating the food to the onset of the allergic symptoms for EGGS? (Time until onset in MINUTES)	______________________ (number of minutes)
28. How long does it usually take from eating the food to the onset of the allergic symptoms for PEANUTS? (Time until onset in DAYS)	______________________ (number of days)
28. How long does it usually take from eating the food to the onset of the allergic symptoms for PEANUTS? (Time until onset in HOURS)	______________________ (number of hours)
28. How long does it usually take from eating the food to the onset of the allergic symptoms for PEANUTS? (Time until onset in MINUTES)	______________________ (number of minutes)
28. How long does it usually take from eating the food to the onset of the allergic symptoms for TREE NUTS? (Time until onset in DAYS)	______________________ (number of days)
28. How long does it usually take from eating the food to the onset of the allergic symptoms for TREE NUTS? (Time until onset in HOURS)	______________________ (number of hours)
28. How long does it usually take from eating the food to the onset of the allergic symptoms for TREE NUTS? (Time until onset in MINUTES)	______________________ (number of minutes)
28. How long does it usually take from eating the food to the onset of the allergic symptoms for FISH? (Time until onset in DAYS)	______________________ (number of days)
28. How long does it usually take from eating the food to the onset of the allergic symptoms for FISH? (Time until onset in HOURS)	______________________ (number of hours)
28. How long does it usually take from eating the food to the onset of the allergic symptoms for FISH? (Time until onset in MINUTES)	______________________ (number of minutes)

28. How long does it usually take from eating the food to the onset of the allergic symptoms for SHELLFISH? (Time until onset in DAYS)	__________ (number of days)
28. How long does it usually take from eating the food to the onset of the allergic symptoms for SHELLFISH? (Time until onset in HOURS)	__________ (number of hours)
28. How long does it usually take from eating the food to the onset of the allergic symptoms for SHELLFISH? (Time until onset in MINUTES)	__________ (number of minutes)
28. How long does it usually take from eating the food to the onset of the allergic symptoms for WHEAT? (Time until onset in DAYS)	__________ (number of days)
28. How long does it usually take from eating the food to the onset of the allergic symptoms for WHEAT? (Time until onset in HOURS)	__________ (number of hours)
28. How long does it usually take from eating the food to the onset of the allergic symptoms for WHEAT? (Time until onset in MINUTES)	__________ (number of minutes)
28. How long does it usually take from eating the food to the onset of the allergic symptoms for SOY/TOFU? (Time until onset in DAYS)	__________ (number of days)
28. How long does it usually take from eating the food to the onset of the allergic symptoms for SOY/TOFU? (Time until onset in HOURS)	__________ (number of hours)
28. How long does it usually take from eating the food to the onset of the allergic symptoms for SOY/TOFU? (Time until onset in MINUTES)	__________ (number of minutes)
28. How long does it usually take from eating the food to the onset of the allergic symptoms for SEEDS? (Time until onset in DAYS)	__________ (number of days)
28. How long does it usually take from eating the food to the onset of the allergic symptoms for SEEDS? (Time until onset in HOURS)	__________ (number of hours)
28. How long does it usually take from eating the food to the onset of the allergic symptoms for SEEDS? (Time until onset in MINUTES)	__________ (number of minutes)
28. Name of Other Food Allergy #1	__________
28. How long does it usually take from eating the food to the onset of the allergic symptoms for OTHER FOOD ALLERGY #1? (Time until onset in DAYS)	__________ (number of days)

28. How long does it usually take from eating the food to the onset of the allergic symptoms for OTHER FOOD ALLERGY #1? (Time until onset in HOURS)	__________________ (number of hours)
28. How long does it usually take from eating the food to the onset of the allergic symptoms for OTHER FOOD ALLERGY #1? (Time until onset in MINUTES)	__________________ (number of minutes)
28. Name of Other Food Allergy #2	__________________
28. How long does it usually take from eating the food to the onset of the allergic symptoms for OTHER FOOD ALLERGY #2? (Time until onset in DAYS)	__________________ (number of days)
28. How long does it usually take from eating the food to the onset of the allergic symptoms for OTHER FOOD ALLERGY #2? (Time until onset in HOURS)	__________________ (number of hours)
28. How long does it usually take from eating the food to the onset of the allergic symptoms for OTHER FOOD ALLERGY #2? (Time until onset in MINUTES)	__________________ (number of minutes)
28. Name of Other Food Allergy #3	__________________
28. How long does it usually take from eating the food to the onset of the allergic symptoms for OTHER FOOD ALLERGY #3? (Time until onset in DAYS)	__________________ (number of days)
28. How long does it usually take from eating the food to the onset of the allergic symptoms for OTHER FOOD ALLERGY #3? (Time until onset in HOURS)	__________________ (number of hours)
28. How long does it usually take from eating the food to the onset of the allergic symptoms for OTHER FOOD ALLERGY #3? (Time until onset in MINUTES)	__________________ (number of minutes)
28. Name of Other Food Allergy #4	__________________
28. How long does it usually take from eating the food to the onset of the allergic symptoms for OTHER FOOD ALLERGY #4? (Time until onset in DAYS)	__________________ (number of days)
28. How long does it usually take from eating the food to the onset of the allergic symptoms for OTHER FOOD ALLERGY #4? (Time until onset in HOURS)	__________________ (number of hours)

28. How long does it usually take from eating the food to the onset of the allergic symptoms for OTHER FOOD ALLERGY #4? (Time until onset in MINUTES)	______________ (number of minutes)
28. Name of Other Food Allergy #5	______________
28. How long does it usually take from eating the food to the onset of the allergic symptoms for OTHER FOOD ALLERGY #5? (Time until onset in DAYS)	______________ (number of days)
28. How long does it usually take from eating the food to the onset of the allergic symptoms for OTHER FOOD ALLERGY #5? (Time until onset in HOURS)	______________ (number of hours)
28. How long does it usually take from eating the food to the onset of the allergic symptoms for OTHER FOOD ALLERGY #5? (Time until onset in MINUTES)	______________ (number of minutes)
28. Name of Other Food Allergy #6	______________
28. How long does it usually take from eating the food to the onset of the allergic symptoms for OTHER FOOD ALLERGY #6? (Time until onset in DAYS)	______________ (number of days)
28. How long does it usually take from eating the food to the onset of the allergic symptoms for OTHER FOOD ALLERGY #6? (Time until onset in HOURS)	______________ (number of hours)
28. How long does it usually take from eating the food to the onset of the allergic symptoms for OTHER FOOD ALLERGY #6? (Time until onset in MINUTES)	______________ (number of minutes)

29. Since the last visit, has your child ever had an allergic reaction that improved completely and then came back?

¿En el último año, ha tenido ... una reacción alérgica que mejoró completamente y luego regreso?

29. Since the last visit, has your child ever had an allergic reaction that improved completely and then came back?	○ Yes<br>○ No
29. If yes, timing to onset of recurrent symptoms: Cow's Milk/Dairy Products/Cheese Days	______________ (Days)

29. If yes, timing to onset of recurrent symptoms:
Cow's Milk/Dairy Products/Cheese
Hours
_______________ (Hours)

29. If yes, timing to onset of recurrent symptoms:
Cow's Milk/Dairy Products/Cheese
Minutes
_______________ (Minutes)

29. If yes, timing to onset of recurrent symptoms:
Eggs
Days
_______________ (Days)

29. If yes, timing to onset of recurrent symptoms:
Eggs
Hours
_______________ (hours)

29. If yes, timing to onset of recurrent symptoms:
Eggs
Minutes
_______________ (Minutes)

29. If yes, timing to onset of recurrent symptoms:
Peanut
Days
_______________ (days)

29. If yes, timing to onset of recurrent symptoms:
Peanut
Hours
_______________ (Hours)

29. If yes, timing to onset of recurrent symptoms:
Peanut
Minutes
_______________ (Minutes)

29. If yes, timing to onset of recurrent symptoms:
Tree nuts
Days
_______________ (days)

29. If yes, timing to onset of recurrent symptoms:
Tree nuts
Hours
_______________ (Hours)

29. If yes, timing to onset of recurrent symptoms:
Tree nuts
Minutes
_______________ (Minutes)

29. If yes, timing to onset of recurrent symptoms:
Fish
Days
_______________ (Days)

29. If yes, timing to onset of recurrent symptoms:
Fish
Hours
_______________ (Hours)

29. If yes, timing to onset of recurrent symptoms:
Fish
Minutes
_______________ (Minutes)

29. If yes, timing to onset of recurrent symptoms:
Shellfish
Days

_______________ (Days)

29. If yes, timing to onset of recurrent symptoms:
Shellfish
Hours

_______________ (Hours)

29. If yes, timing to onset of recurrent symptoms:
Shellfish
Minutes

_______________ (Minutes)

29. If yes, timing to onset of recurrent symptoms:
Wheat
Days

_______________ (Days)

29. If yes, timing to onset of recurrent symptoms:
Wheat
Hours

_______________ (Hours)

29. If yes, timing to onset of recurrent symptoms:
Wheat
Minutes

_______________ (Minutes)

29. If yes, timing to onset of recurrent symptoms:
Soy/Tofu
Days

_______________ (Days)

29. If yes, timing to onset of recurrent symptoms:
Soy/Tofu
Hours

_______________ (Hours)

29. If yes, timing to onset of recurrent symptoms:
Soy/Tofu
Minutes

_______________ (Minutes)

29. If yes, timing to onset of recurrent symptoms:
Seeds
Days

_______________ (Days)

29. If yes, timing to onset of recurrent symptoms:
Seeds
Hours

_______________ (Hours)

29. If yes, timing to onset of recurrent symptoms:
Seeds
Minutes

_______________ (Min)

29. If yes, timing to onset of recurrent symptoms:
Other 1
Days

_______________ (Days)

29. If yes, timing to onset of recurrent symptoms:
Other 1
Hours

_______________ (Hours)

29. If yes, timing to onset of recurrent symptoms:
Other 1
Minutes
_______________ (Minutes)

29. If yes, timing to onset of recurrent symptoms:
Other 2
Days
_______________ (Days)

29. If yes, timing to onset of recurrent symptoms:
Other 2
Hours
_______________ (Hours)

29. If yes, timing to onset of recurrent symptoms:
Other 2
Minutes
_______________ (Minutes)

29. If yes, timing to onset of recurrent symptoms:
Other 3
Days
_______________ (Days)

29. If yes, timing to onset of recurrent symptoms:
Other 3
Hours
_______________ (Hours)

29. If yes, timing to onset of recurrent symptoms:
Other 3
Minutes
_______________ (Minutes)

29. If yes, timing to onset of recurrent symptoms:
Other 4
Days
_______________ (Days)

29. If yes, timing to onset of recurrent symptoms:
Other 4
Hours
_______________ (Hours)

29. If yes, timing to onset of recurrent symptoms:
Other 4
Minutes
_______________ (Minutes)

29. If yes, timing to onset of recurrent symptoms:
Other 5
Days
_______________ (Days)

29. If yes, timing to onset of recurrent symptoms:
Other 5
Hours
_______________ (Hours)

29. If yes, timing to onset of recurrent symptoms:
Other 5
Minutes
_______________ (Minutes)

29. If yes, timing to onset of recurrent symptoms:
Other 6
Days
_______________ (Days)

29. If yes, timing to onset of recurrent symptoms:
Other 6
Hours

____
(Hours)

29. If yes, timing to onset of recurrent symptoms:
Other 6
Minutes

____
(Minutes)

Pediatric Sleep Questionnaire

A47. Does the time at which your child goes to bed change a lot from day to day?

¿La hora en que... duerme cambia mucho cada día o normalmente duerme a la misma hora cada día?

○ Yes
○ No
○ Don't know

A48. Does the time at which your child gets up from bed change a lot from day to day?

¿La hora en que... levante cambia mucho cada día o se levante a la misma hora cada día?

○ Yes
○ No
○ Don't know

A49. What time does your child go to bed (fall asleep) during the week?

¿A que hora duerme... normalmente durante la semana?

____
(24hr)

A50. What time does your child go to bed (fall asleep) on the weekend or vacation?

¿A que hora duerme... normalmente durante la fin de semana?

____
(24hr)

A51. What time does your child usually get out of bed (wake up) on weekday mornings?

¿A que hora se levanta ... normalmente la semana?

____
(24 hr)

A52. What time does your child usually get out of bed (wake up) on weekend or vacation mornings?

¿A que hora se levanta... normalmente durante la fin de semana?

____
(24 hr)

A.53 How many hours of sleep does your child usually get on (weekday) school nights?
Hours

____
(Hours)

A.53 How many hours of sleep does your child usually get on (weekday) school nights?
Minutes

____
(Minutes)

A.54 How many hours of sleep does your child usually get on (weekend) non-school nights?
Hours

____
(Hours)

A.54 How many hours of sleep does your child usually get on (weekend) non-school nights?
Minutes

____
(Minutes)

If the child is < 2 years old skip to question B10

B7. Does your child wake up with headaches in the morning?

¿Normalmente, tiene...dolor de cabeza en la mañana?

◯ Yes
◯ No
◯ Don't know

B8. Does your child get a headache at least once a month, on average?

¿Tiene dolor de cabeza al menos una vez por mes?

◯ Yes
◯ No
◯ Don't know

B10. Does your child still have tonsils and/or adenoids?

¿Tiene... sus amígdalas o adenoides o han sido removidos? (por una cirugía)

◯ Yes
◯ No
◯ Don't know

If not, when were they removed?
years

____
(years)

If not, when were they removed?
Months

____
(Months)

B11. Has your child ever had a condition causing difficulty with breathing?

¿Ha tenido... un problema que causa dificultad para respirar?

◯ Yes
◯ No
◯ Don't know

If so, please describe

____

30. On an average day, how many hours and minutes does your child watch Tv?

◯ Don't know

30. On an average day, how many hours and minutes does your child watch TV?
Hours

¿Cuántas horas por día mira televisión?
Horas

____
(Hours/ horas)

30. On an average day, how many hours and minutes does your child watch TV?

¿Cuántas horas por día mira televisión?

____
(Minutes)

31. On an average day, how many hours and minutes does your child spend playing video games or sitting in front of the computer? Include both time spent on the computer at home and at school.	◯ Don't know
31. On an average day, how many hours and minutes does your child spend playing video games or sitting in front of the computer? Include both time spent on the computer at home and at school.<br>Hours<br><br>¿Cuántas horas por día está... en la computadora?	________________<br>(Hours)
31. On an average day, how many hours and minutes does your child spend playing video games or sitting in front of the computer? Include both time spent on the computer at home and at school.<br>Minutes<br><br>¿Cuántas horas por día está... en la computadora?	________________<br>(Minutes)
32. If your child goes to school, in an average week when your child is in school, how many days does your child go to physical education (PE) classes?	◯ Don't know<br>◯ Doesn't attend school
32. If your child goes to school, in an average week when your child is in school, how many days does your child go to physical education (PE) classes?<br>Days<br><br>¿En la escuela, va... a clase de gimnasia?<br>¿Cuántas días por semana?	________________<br>(Days)
33. Since the last visit, did your child play on any sports teams or participate in other organized physical activities? Some examples would include dance classes, YMCA swim classes, weekend park district, church or school basketball teams, or other teams or activities run by schools or local community centers.<br><br>¿En el último año, participó... en algunos deportes?	◯ Yes<br>◯ No<br>◯ Don't know
34. On an average day, how many hours and minutes does your child spend in active play? Some examples of active play include running around, playing catch, basketball and bicycling. (PROMPT: If respondent indicates that answer would vary based on the time of year, ask them to average out their child's activity. For example, if the answer in the summer would be 8 hours a day, and in the winter 4 hours, the answer should be hours. You may need to help them with the math.<br>Hours<br><br>¿Cuántas horas por día está corriendo, jugando, haciendo muy activo(a)?	________________<br>(Hours)

34. On an average day, how many hours and minutes does your child spend in active play? Some examples of active play include running around, playing catch, basketball and bicycling. (PROMPT: If respondent indicates that answer would vary based on the time of year, ask them to average out their child's activity. For example, if the answer in the summer would be 8 hours a day, and in the winter 4 hours, the answer should be hours. You may need to help them with the math.<br>Minutes<br><br>¿Cuántas horas por día está corriendo, jugando, haciendo muy activo(a)?	________________________<br>(Minutes)
34. On an average day, how many hours and minutes does your child spend in active play? Some examples of active play include running around, playing catch, basketball and bicycling. (PROMPT: If respondent indicates that answer would vary based on the time of year, ask them to average out their child's activity. For example, if the answer in the summer would be 8 hours a day, and in the winter 4 hours, the answer should be hours. You may need to help them with the math.<br>Hours	◯ Don't know<br>(Hours)
35. About how physically active is you child compared to other children his/her age? Would you say about the same, a lot less, a little less, a little more, a lot more active?<br><br>¿Ud. Cree que, comparado a otros niños de la misma edad, que... es más activo, o menos activos que otros niños? ¿Mucho más (o menos) o poco más (menos)?	◯ 1. A lot less active<br>◯ 2. A little less active<br>◯ 3. Same<br>◯ 4. A little more active<br>◯ 5. A lot more active
36. Do you live close enough to your child's school that he/she could walk or bike to school?<br><br>¿Ud. Vive cerca de la escuela para que puede caminar o montar la bicicleta a escuela?	◯ Yes<br>◯ No<br>◯ Don't know<br>◯ Not applicable
37. How many days a week does your child bike to school?	◯ Don't know
37. How many days a week does your child bike to school?<br>Days<br><br>¿Cuántas días por semana monta su bicicleta para ir a la escuela?	________________________<br>(days)
38. How many days a week does your child walk to school?	◯ Don't Know

38. How many days a week does your child walk to school?
Days

¿Cuántas días por semana camina para ir a la escuela?

____
(Days)

Section II. Family History

39. What is your present marital status?

¿Mamá Ud. esta...?
Casada
Viuda
Divorciada
Separada
Soltera

- ◯ Married
- ◯ Widowed
- ◯ Divorced
- ◯ Separated
- ◯ Single

40. What is the highest grade of school you have completed to date?

¿Qué grado de escuela Ud. terminó?

- ◯ No school
- ◯ Elementary school
- ◯ Some secondary school (9th grade and above)
- ◯ High school graduate or GED
- ◯ Some college
- ◯ College degree
- ◯ Graduate school degree
- ◯ Post Graduate (PhD/MD/Other)

41. Are you currently working for pay?

¿Ud. Está trabajando?

- ◯ Yes
- ◯ No
- ◯ Retired

42. What is your occupation/job title?

¿Cuál es su ocupación?

____

41. What field does your occupation fall under?

- ◯ Not Applicable
- ◯ Management/Business/Administration
- ◯ Financial/Computer/Mathematical
- ◯ Architecture and Engineering
- ◯ Life, Physical, and Social Science
- ◯ Legal Occupations
- ◯ Education, Training, and Library
- ◯ Sales, Arts, Design, Entertainment, and Media
- ◯ Athletics (Sports, Dancing, etc)
- ◯ Healthcare
- ◯ Food Preparation and Serving
- ◯ Building and Grounds Cleaning and Maintenance
- ◯ Personal Care and Service
- ◯ Farming, Fishing, and Forestry
- ◯ Construction Trades
- ◯ Extraction Workers
- ◯ Installation, Maintenance, and Repair Workers
- ◯ Production Occupations
- ◯ Transportation and Material Moving
- ◯ Military Specific
- ◯ Other
- ◯ Don't know

42. Will you answer some questions about your child's biological father?<br><br>¿Puedo preguntar sobre el padre?	◯ Yes<br>◯ No
43. What is the highest grade of school he has completed to date?<br><br>¿Y por el padre que grado de escuela terminó el?	◯ Elementary school<br>◯ Some secondary school (9th grade and above)<br>◯ High school graduate or GED<br>◯ Some college<br>◯ College degree<br>◯ Graduate school degree<br>◯ Post Graduate (PhD/MD/Other)<br>◯ Don't know
44. Is he currently working for pay?<br><br>¿Y él está trabajando?	◯ Yes<br>◯ No<br>◯ Retired<br>◯ Don't know
What is his occupation/ Job title<br>Don't know	◯ Don't know
45. What is his occupation/job title?<br><br>¿Cuál es su ocupación?	__________________________________
46. What field does his occupation fall under?	◯ Not Applicable<br>◯ Management/Business/Administration<br>◯ Financial/Computer/Mathematical<br>◯ Architecture and Engineering<br>◯ Life, Physical, and Social Science<br>◯ Legal Occupations<br>◯ Education, Training, and Library<br>◯ Sales, Arts, Design, Entertainment, and Media<br>◯ Athletics (Sports, Dancing, etc)<br>◯ Healthcare<br>◯ Food Preparation and Serving<br>◯ Building and Grounds Cleaning and Maintenance<br>◯ Personal Care and Service<br>◯ Farming, Fishing, and Forestry<br>◯ Construction Trades<br>◯ Extraction Workers<br>◯ Installation, Maintenance, and Repair Workers<br>◯ Production Occupations<br>◯ Transportation and Material Moving<br>◯ Military Specific<br>◯ Other<br>◯ Don't know

Section III. Home Environment

47. What was your total household income last year, before taxes? (INCLUDES PUBLIC ASSISTANCE)

¿Cuál fue su ingreso familiar el año pasado antes de impuestos?

- ◯ < $5,000
- ◯ $5,000-9,999
- ◯ $10,000-14,999
- ◯ $15,000-19,999
- ◯ $20,000-24,999
- ◯ $25,000-29,999
- ◯ $30,000-34,999
- ◯ $35,000-39,999
- ◯ $40,000-49,999
- ◯ $50,000-59,999
- ◯ $60,000-79,999
- ◯ $80,000-99,999
- ◯ > $100,000
- ◯ Don't know

48. Here are some questions about your current home:

a) How long have you lived in your current home? (TIME IN YEARS)

¿Cuántos años ha vivido Ud. en su Casa?

____

a) How long have you lived in your current home? (TIME IN MONTHS)

____

b) What type of housing is your home?

¿Qué tipo de casa?
aparatamento

- ◯ Single family
- ◯ Duplex
- ◯ Row House
- ◯ Condo/Apartment
- ◯ Trailer Home
- ◯ Shelter

c) # of bedrooms

¿Cuántas habitaciones tiene en la casa?

____

d) # of bathrooms

¿Cuántos baños?

____

e) # of people who permanently live in your home

¿Cuántas personas viven allí?

____

f) What type of fuel do you use for heating your home?

¿Qué usa Ud. para calentar la casa?
Aceite
Electricidad
Gas

- ◯ Oil
- ◯ Electricity
- ◯ Gas

If Others, specify:

____
(other type of fuel used for heating the home)

g) What type of stove do you use for cooking?<br><br>¿Y para cocinar?<br>Gas<br>Electricidad	◯ Gas<br>◯ Electric
If Others, specify:	__________________________________<br>(other type of fuel used for cooking)
h) Do you have any wall to wall carpet in your home?<br><br>¿Hay alfombra de pared a pared en alguna parte de la casa?	◯ Yes<br>◯ No
If yes, specify location:<br><br>Sala<br>Sala de estar<br>Comendar<br>Cocina<br>Habitaciones<br>Sótano<br>Baño	☐ Living room<br>☐ Family room<br>☐ Dining room<br>☐ Kitchen<br>☐ Bedroom (master) parents<br>☐ Bedroom index child<br>☐ Bedroom Sib#1<br>☐ Bedroom Sib#2<br>☐ Basement<br>☐ Bathroom
i) Approximately how old is the building/apartment/home you live in?<br><br>¿Cuántos años tiene desde su casa ha sido consumado?	◯ 10 years or less<br>◯ 11-25 years<br>◯ 26-50 years<br>◯ 51-75 years<br>◯ Greater than 75 years old<br>◯ Don't know
49. Do you (mother of the child) currently smoke cigarettes, cigars, or pipes?<br><br>¿Ud. fuma? (¿Ha fumado?)	◯ Yes<br>◯ No
If yes, what do/did you smoke?<br><br>Cigarrillos<br>Cigarros<br>Pipa	◯ Cigarettes<br>◯ Cigars<br>◯ Pipes
If yes to Q 49,<br>Do you smoke inside the home?<br><br>¿Fuma en la casa?	◯ Yes<br>◯ No
How many (cigarettes, cigars, pipes) do you smoke PER DAY (Regardless of indoor or outdoor)<br><br>¿Cuántos cigarrillos fuma por día?<br>En la casa o a fuera	__________________________________
50a. Can I ask you about your child's biological father's smoking status?	◯ Yes<br>◯ No

50b. Does your child's father currently smoke cigarettes, cigars, or pipes?<br><br>¿Y el padre de ... fuma?	◯ Yes<br>◯ No<br>◯ Don't Know
If yes, what does/did he smoke?<br><br>Cigarrillos<br>Cigarros<br>Pipa	◯ Cigarettes<br>◯ Cigars<br>◯ Pipes
If yes to Q 50b,<br>Does he smoke inside the home?<br><br>¿Fuma él en la casa?	◯ Yes<br>◯ No
How many (cigarettes, cigars, pipes) does he smoke PER DAY (Regardless of indoor or outdoor)?<br><br>¿Cuántos cigarrillos fuma por día?	______________________<br>(per day)
51. Do other people who currently live in your home cigarettes, cigars or pipes (not including the mother and father of the child)?<br><br>¿Hay otras personas en la casa que fuman?	◯ Yes<br>◯ No
How many people?<br><br>¿Cuántas personas?	______________________<br>(# of people)
How many of them smoke inside the home?<br><br>¿Cuántas personas fuman en la casa?	______________________
52. Total numbers of cigarettes smoked inside your home per day (NOT INCLUDING AMOUNT SMOKED by yourself and the father of your child)?<br><br>¿Cuántos cigarrillos fuman por día en la casa?	______________________
53. Do you currently have any pets in your home?<br><br>¿Tiene Ud. mascotas o animales en la casa?	◯ Yes<br>◯ No

If yes, specify type of pet and how many of each type:

	Yes	No
Cat / Gato	◯	◯
Dog / Pero	◯	◯
Reptiles / Reptil	◯	◯
Rabbit / Conejo	◯	◯

Fish / Pez	◯	◯
Guinea Pig	◯	◯
Birds / Pájaro	◯	◯
Others	◯	◯

How many cats? ______________________

How many dogs? ______________________

How many reptiles? ______________________

How many rabbits? ______________________

How many fish? ______________________

How many guinea pigs? ______________________

How many birds? ______________________

If others, specify:
Type other 1 ______________________

How many others?
First other ______________________

If others, specify:
Type other 2 ______________________

How many others?
Second other ______________________

If others, specify:
Type other 3 ______________________

How many others?
Third other ______________________

54. Does the house you live in have any cockroaches?

¿Hay cucarachas en la casa?

◯ Yes
◯ No
◯ Unsure

55. Does the house you live in have any mice/rats?

¿Hay ratones en las casa?

◯ Yes
◯ No

56. Does the house you live in have any visible mold, mildew, water damage, leakage or seepage?

¿Hay moho o daños por agua en la casa?

◯ Yes
◯ No

56. Do you currently live in a farming environment?

¿Ud. no vive en una granja, verdad?

◯ Yes
◯ No

Modified Checklist for Autism in Toddlers, Revised, with Follow-Up

(M-CHAT-R/F)™

Diana L. Robins, Ph.D.
Deborah Fein, Ph.D.
Marianne Barton, Ph.D.

Acknowledgement: We thank the M-CHAT Study Group in Spain for developing the flow chart format used in this document.

For more information, please see www.mchatscreen.com
or contact Diana Robins at mchatscreen2009@gmail.com

Note. This version contains minor corrections. August 10, 2018.

© 2009 Diana Robins, Deborah Fein, & Marianne Barton

Permissions for Use of the M-CHAT-R/F^{TM}

The Modified Checklist for Autism in Toddlers, Revised with Follow-Up (M-CHAT-R/F; Robins, Fein, & Barton, 2009) is a 2-stage parent-report screening tool to assess risk for Autism Spectrum Disorder (ASD). The M-CHAT-R/F is available for free download for clinical, research, and educational purposes. Download of the M-CHAT-R/F and related material is authorized from www.mchatscreen.com.

The M-CHAT-R/F is a copyrighted instrument, and use of the M-CHAT-R/F must follow these guidelines:

(1) Reprints/reproductions of the M-CHAT-R must include the copyright at the bottom (© 2009 Robins, Fein, & Barton). No modifications can be made to items, instructions, or item order without permission from the authors.
(2) The M-CHAT-R must be used in its entirety. Evidence indicates that any subsets of items do not demonstrate adequate psychometric properties.
(3) Parties interested in reproducing the M-CHAT-R/F in print (e.g., a book or journal article) or electronically for use by others (e.g., as part of digital medical record or other software packages) must contact Diana Robins to request permission (mchatscreen2009@gmail.com).
(4) If you are part of a medical practice, and you want to incorporate the first stage M-CHAT-R questions into your own practice's electronic medical record (EMR), you are welcome to do so. However, if you ever want to distribute your EMR page outside of your practice, please contact Diana Robins to request a licensing agreement.

Instructions for Use

The M-CHAT-R can be administered and scored as part of a well-child care visit, and also can be used by specialists or other professionals to assess risk for ASD. The primary goal of the M-CHAT-R is to maximize sensitivity, meaning to detect as many cases of ASD as possible. Therefore, there is a high false positive rate, meaning that not all children who score at risk will be diagnosed with ASD. To address this, we have developed the Follow-Up questions (M-CHAT-R/F). Users should be aware that even with the Follow-Up, a significant number of the children who screen positive on the M-CHAT-R will not be diagnosed with ASD; however, these children are at high risk for other developmental disorders or delays, and therefore, evaluation is warranted for any child who screens positive. The M-CHAT-R can be scored in less than two minutes. Scoring instructions can be downloaded from http://www.mchatscreen.com. Associated documents will be available for download as well.

Scoring Algorithm

For all items except 2, 5, and 12, the response "NO" indicates ASD risk; for items 2, 5, and 12, "YES" indicates ASD risk. The following algorithm maximizes psychometric properties of the M-CHAT-R:

LOW-RISK: **Total Score is 0-2**; if child is younger than 24 months, screen again after second birthday. No further action required unless surveillance indicates risk for ASD.

MEDIUM-RISK: **Total Score is 3-7**; Administer the Follow-Up (second stage of M-CHAT-R/F) to get additional information about at-risk responses. If M-CHAT-R/F score remains at 2 or higher, the child has screened positive. Action required: refer child for diagnostic evaluation and eligibility evaluation for early intervention. If score on Follow-Up is 0-1, child has screened negative. No further action required unless surveillance indicates risk for ASD. Child should be rescreened at future well-child visits.

HIGH-RISK: **Total Score is 8-20**; It is acceptable to bypass the Follow-Up and refer immediately for diagnostic evaluation and eligibility evaluation for early intervention.

© 2009 Diana Robins, Deborah Fein, & Marianne Barton

M-CHAT-R[TM]

Please answer these questions about your child. Keep in mind how your child usually behaves. If you have seen your child do the behavior a few times, but he or she does not usually do it, then please answer **no**. Please circle **yes** or **no** for every question. Thank you very much.

1. If you point at something across the room, does your child look at it? (**FOR EXAMPLE**, if you point at a toy or an animal, does your child look at the toy or animal?)	Yes	No
2. Have you ever wondered if your child might be deaf?	Yes	No
3. Does your child play pretend or make-believe? (**FOR EXAMPLE**, pretend to drink from an empty cup, pretend to talk on a phone, or pretend to feed a doll or stuffed animal?)	Yes	No
4. Does your child like climbing on things? (**FOR EXAMPLE**, furniture, playground equipment, or stairs)	Yes	No
5. Does your child make unusual finger movements near his or her eyes? (**FOR EXAMPLE**, does your child wiggle his or her fingers close to his or her eyes?)	Yes	No
6. Does your child point with one finger to ask for something or to get help? (**FOR EXAMPLE**, pointing to a snack or toy that is out of reach)	Yes	No
7. Does your child point with one finger to show you something interesting? (**FOR EXAMPLE**, pointing to an airplane in the sky or a big truck in the road)	Yes	No
8. Is your child interested in other children? (**FOR EXAMPLE**, does your child watch other children, smile at them, or go to them?)	Yes	No
9. Does your child show you things by bringing them to you or holding them up for you to see – not to get help, but just to share? (**FOR EXAMPLE**, showing you a flower, a stuffed animal, or a toy truck)	Yes	No
10. Does your child respond when you call his or her name? (**FOR EXAMPLE**, does he or she look up, talk or babble, or stop what he or she is doing when you call his or her name?)	Yes	No
11. When you smile at your child, does he or she smile back at you?	Yes	No
12. Does your child get upset by everyday noises? (**FOR EXAMPLE**, does your child scream or cry to noise such as a vacuum cleaner or loud music?)	Yes	No
13. Does your child walk?	Yes	No
14. Does your child look you in the eye when you are talking to him or her, playing with him or her, or dressing him or her?	Yes	No
15. Does your child try to copy what you do? (**FOR EXAMPLE**, wave bye-bye, clap, or make a funny noise when you do)	Yes	No
16. If you turn your head to look at something, does your child look around to see what you are looking at?	Yes	No
17. Does your child try to get you to watch him or her? (**FOR EXAMPLE**, does your child look at you for praise, or say "look" or "watch me"?)	Yes	No
18. Does your child understand when you tell him or her to do something? (**FOR EXAMPLE**, if you don't point, can your child understand "put the book on the chair" or "bring me the blanket"?)	Yes	No
19. If something new happens, does your child look at your face to see how you feel about it? (**FOR EXAMPLE**, if he or she hears a strange or funny noise, or sees a new toy, will he or she look at your face?)	Yes	No
20. Does your child like movement activities? (**FOR EXAMPLE**, being swung or bounced on your knee)	Yes	No

© 2009 Diana Robins, Deborah Fein, & Marianne Barton

M-CHAT-R Follow-Up (M-CHAT-R/F)™

Permissions for Use

The Modified Checklist for Autism in Toddlers, Revised, with Follow-Up (M-CHAT-R/F; Robins, Fein, & Barton, 2009) is designed to accompany the M-CHAT-R. The M-CHAT-R/F may be downloaded from www.mchatscreen.com.

The M-CHAT-R/F is a copyrighted instrument, and use of this instrument is limited by the authors and copyright holders. The M-CHAT-R and M-CHAT-R/F may be used for clinical, research, and educational purposes. Although we are making the tool available free of charge for these uses, this is copyrighted material and it is not open source. Anyone interested in using the M-CHAT-R/F in any commercial or electronic products must contact Diana L. Robins at mchatscreen2009@gmail.com to request permission.

Instructions for Use

The M-CHAT-R/F is designed to be used with the M-CHAT-R; the M-CHAT-R is valid for screening toddlers between 16 and 30 months of age, to assess risk for autism spectrum disorder (ASD). Users should be aware that even with the Follow-Up, a significant number of the children who fail the M-CHAT-R will not be diagnosed with ASD; however, these children are at risk for other developmental disorders or delays, and therefore, follow-up is warranted for any child who screens positive.

Once a parent has completed the M-CHAT-R, score the instrument according to the instructions. If the child screens positive, select the Follow-Up items based on which items the child failed on the M-CHAT-R; only those items that were originally failed need to be administered for a complete interview.

Each page of the interview corresponds to one item from the M-CHAT-R. Follow the flowchart format, asking questions until a PASS or FAIL is scored. Please note that parents may report "maybe" in response to questions during the interview. When a parent reports "maybe," ask whether most often the answer is "yes" or "no" and continue the interview according to that response. In places where there is room to report an "other" response, the interviewer must use his/her judgment to determine whether it is a passing response or not.

Score the responses to each item on the M-CHAT-R/F Scoring Sheet (which contains the same items as the M-CHAT-R, but Yes/No has been replaced by Pass/Fail). The interview is considered to be a screen positive if the child fails any two items on the Follow-Up. If a child screens positive on the M-CHAT-R/F, it is strongly recommended that the child is referred for early intervention and diagnostic testing as soon as possible. Please note that if the healthcare provider or parent has concerns about ASDs, children should be referred for evaluation regardless of the score on the M-CHAT-R or M-CHAT-R/F.

© 2009 Diana Robins, Deborah Fein, & Marianne Barton

M-CHAT-R Follow-Up™ Scoring Sheet

Please note: Yes/No has been replaced with Pass/Fail

1.	If you point at something across the room, does your child look at it? (**FOR EXAMPLE**, if you point at a toy or an animal, does your child look at the toy or animal?)	Pass	Fail
2.	Have you ever wondered if your child might be deaf?	Pass	Fail
3.	Does your child play pretend or make-believe? (**FOR EXAMPLE**, pretend to drink from an empty cup, pretend to talk on a phone, or pretend to feed a doll or stuffed animal)	Pass	Fail
4.	Does your child like climbing on things? (**FOR EXAMPLE**, furniture, playground equipment, or stairs)	Pass	Fail
5.	Does your child make <u>unusual</u> finger movements near his or her eyes? (**FOR EXAMPLE**, does your child wiggle his or her fingers close to his or her eyes?)	Pass	Fail
6.	Does your child point with one finger to ask for something or to get help? (**FOR EXAMPLE**, pointing to a snack or toy that is out of reach)	Pass	Fail
7.	Does your child point with one finger to show you something interesting? (**FOR EXAMPLE**, pointing to an airplane in the sky or a big truck in the road)	Pass	Fail
8.	Is your child interested in other children? (**FOR EXAMPLE**, does your child watch other children, smile at them, or go to them?)	Pass	Fail
9.	Does your child show you things by bringing them to you or holding them up for you to see – not to get help, but just to share? (**FOR EXAMPLE**, showing you a flower, a stuffed animal, or a toy truck)	Pass	Fail
10.	Does your child respond when you call his or her name? (**FOR EXAMPLE**, does he or she look up, talk or babble, or stop what he or she is doing when you call his or her name?)	Pass	Fail
11.	When you smile at your child, does he or she smile back at you?	Pass	Fail
12.	Does your child get upset by everyday noises? (**FOR EXAMPLE**, a vacuum cleaner or loud music)	Pass	Fail
13.	Does your child walk?	Pass	Fail
14.	Does your child look you in the eye when you are talking to him or her, playing with him or her, or dressing him or her?	Pass	Fail
15.	Does your child try to copy what you do? (**FOR EXAMPLE**, wave bye-bye, clap, or make a funny noise when you do)	Pass	Fail
16.	If you turn your head to look at something, does your child look around to see what you are looking at?	Pass	Fail
17.	Does your child try to get you to watch him or her? (**FOR EXAMPLE**, does your child look at you for praise, or say "look" or "watch me")	Pass	Fail
18.	Does your child understand when you tell him or her to do something? (**FOR EXAMPLE**, if you don't point, can your child understand "put the book on the chair" or "bring me the blanket")	Pass	Fail
19.	If something new happens, does your child look at your face to see how you feel about it? (**FOR EXAMPLE**, if he or she hears a strange or funny noise, or sees a new toy, will he or she look at your face?)	Pass	Fail
20.	Does your child like movement activities? (**FOR EXAMPLE**, being swung or bounced on your knee)	Pass	Fail

Total Score: _______

© 2009 Diana Robins, Deborah Fein, & Marianne Barton

1. If you point at something across the room, does _____________ look at it?

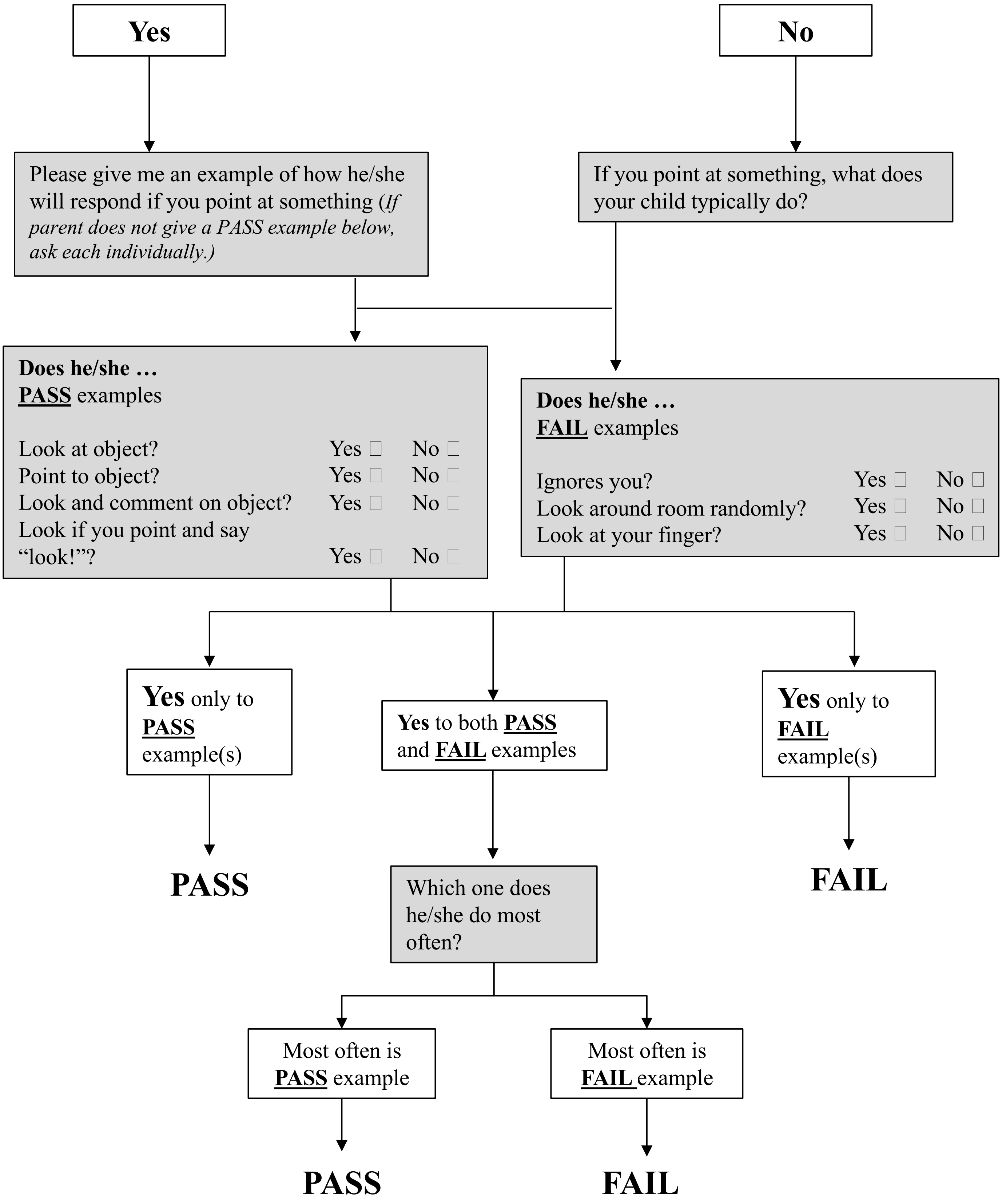

© 2009 Diana Robins, Deborah Fein, & Marianne Barton

2. You reported that you have wondered if you child is deaf. What led you to wonder that?

Does he/she…

often ignore sounds? Yes ☐ No ☐
often ignore people? Yes ☐ No ☐

No to both → **PASS**

Yes to either → **FAIL**

ALSO ASK FOR ALL CHILDREN:

Has your child's hearing been tested?

Yes | **No**

What were the results of the hearing test? *(choose one):*

☐ Hearing in normal range
☐ Hearing below normal
☐ Results inconclusive or not definitive

© 2009 Diana Robins, Deborah Fein, & Marianne Barton

3. Does __________ play pretend or make-believe?

Yes

Please give me an example of his/her pretend play. (*If parent does not give a PASS example below, ask each individually.)*

No

Does he/she usually…

Pretend to drink from a **toy** cup?	Yes ☐	No ☐
Pretend to eat from a **toy** spoon or fork?	Yes ☐	No ☐
Pretend to talk on the telephone?	Yes ☐	No ☐
Pretend to feed a doll or stuffed animal with real or imaginary food?	Yes ☐	No ☐
Push a car as if it is going along a pretend road?	Yes ☐	No ☐
Pretend to be a robot, an airplane, a ballerina, or any other favorite character?	Yes ☐	No ☐
Put a toy pot on a pretend stove?	Yes ☐	No ☐
Stir imaginary food?	Yes ☐	No ☐
Put an action figure or doll into a car or truck as if it is the driver or passenger?	Yes ☐	No ☐
Pretend to vacuum the rug, sweep the floor, or the mow lawn?	Yes ☐	No ☐
Other (describe)	Yes ☐	No ☐

____

____

Yes to any → **PASS**

No to all → **FAIL**

© 2009 Diana Robins, Deborah Fein, & Marianne Barton

4. Does ________ like climbing on things?

Yes

Please give me an example of something he/she enjoys climbing on. (*If parent does not give a PASS example below, ask each individually.)*

No

Does he/she enjoy climbing on…

Stairs?	Yes ☐	No ☐
Chairs?	Yes ☐	No ☐
Furniture?	Yes ☐	No ☐
Playground equipment?	Yes ☐	No ☐

Yes to any of the above → **PASS**

No to all → **FAIL**

© 2009 Diana Robins, Deborah Fein, & Marianne Barton

5. Does ________ make unusual finger movements near his/her eyes?

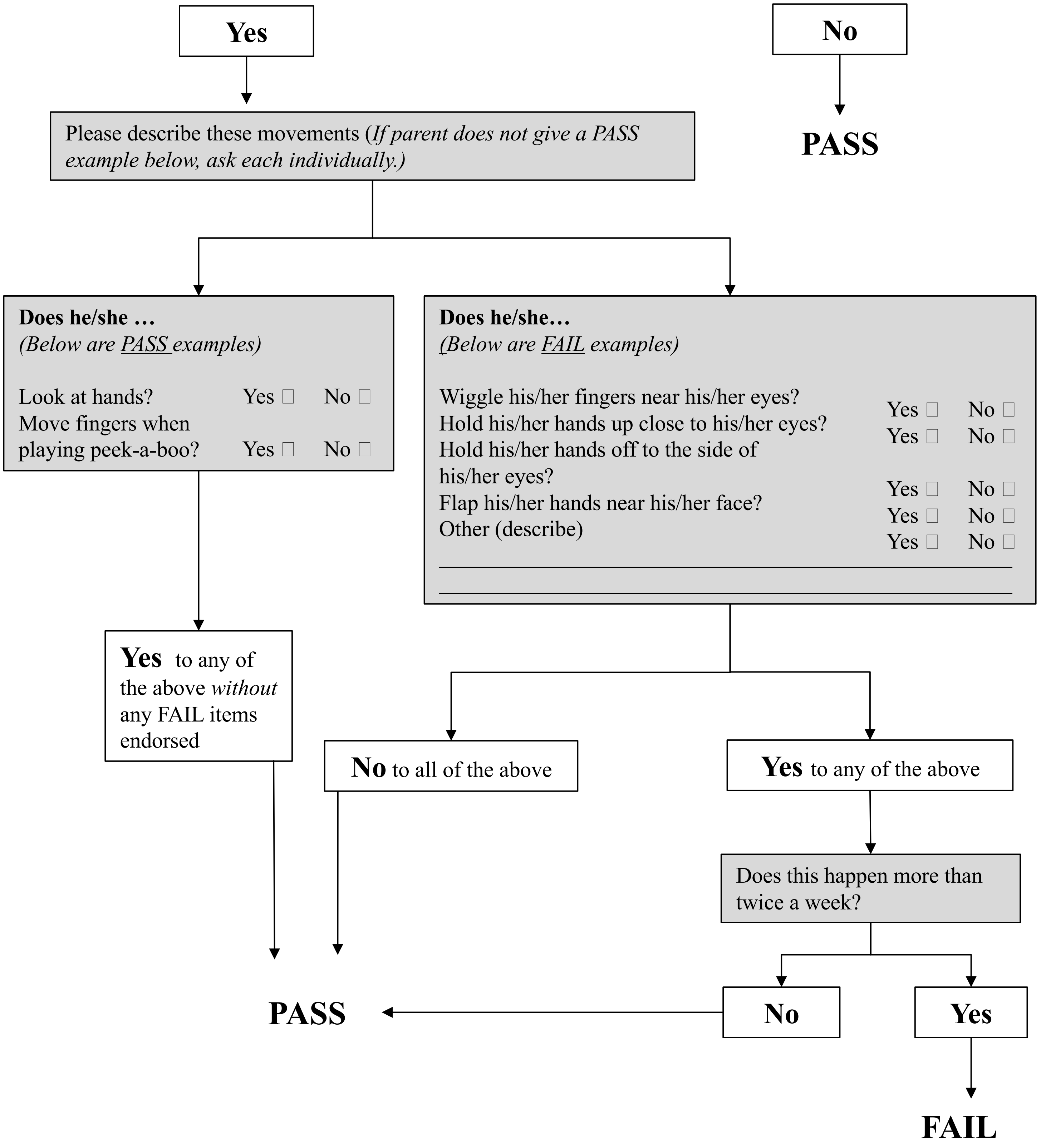

© 2009 Diana Robins, Deborah Fein, & Marianne Barton

6. Does ___________ point with one finger to ask for something or to get help?

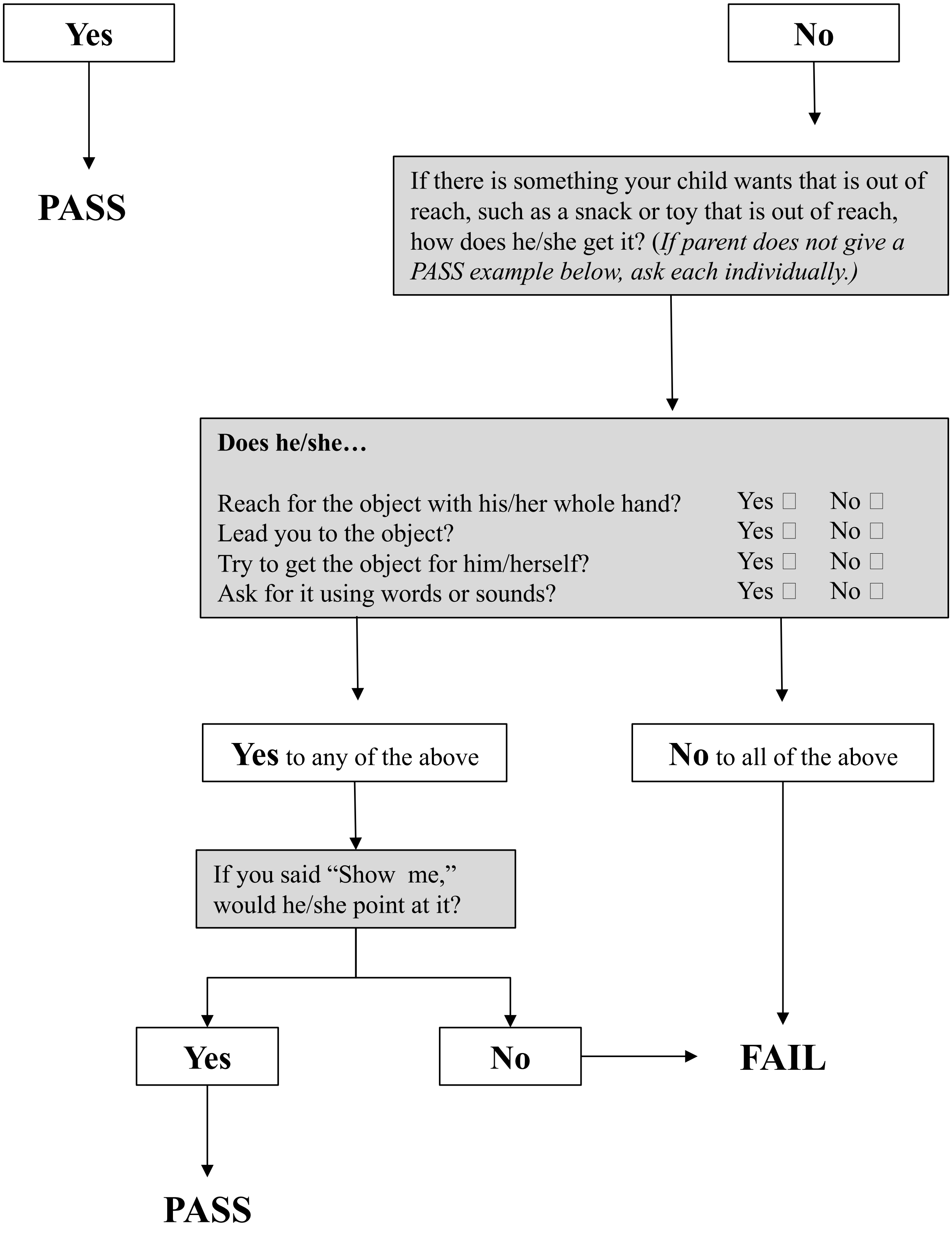

© 2009 Diana Robins, Deborah Fein, & Marianne Barton

7. * **If the interviewer just asked #6, begin here: We just talked about pointing to *ask* for something, ASK ALL → Does _______________ point with one finger just to show you something interesting?**

Yes

Please give me an example something he/she might point at to show you. (*If parent does not give a PASS example below, ask each individually.)*

No

Does your child sometimes want you to see something interesting such as….

An airplane in the sky?	Yes ☐	No ☐
A truck on the road?	Yes ☐	No ☐
A bug on the ground?	Yes ☐	No ☐
An animal in the yard?	Yes ☐	No ☐

Yes to any

How does your child draw your attention to it? Would he/she point with one finger?

Yes

Is this to show their interest, not to get help?

No → **FAIL**

Yes OR both to show interest and to get help

PASS

No

FAIL

No to all of the above

FAIL

© 2009 Diana Robins, Deborah Fein, & Marianne Barton

8. Is ____________ interested in other children?

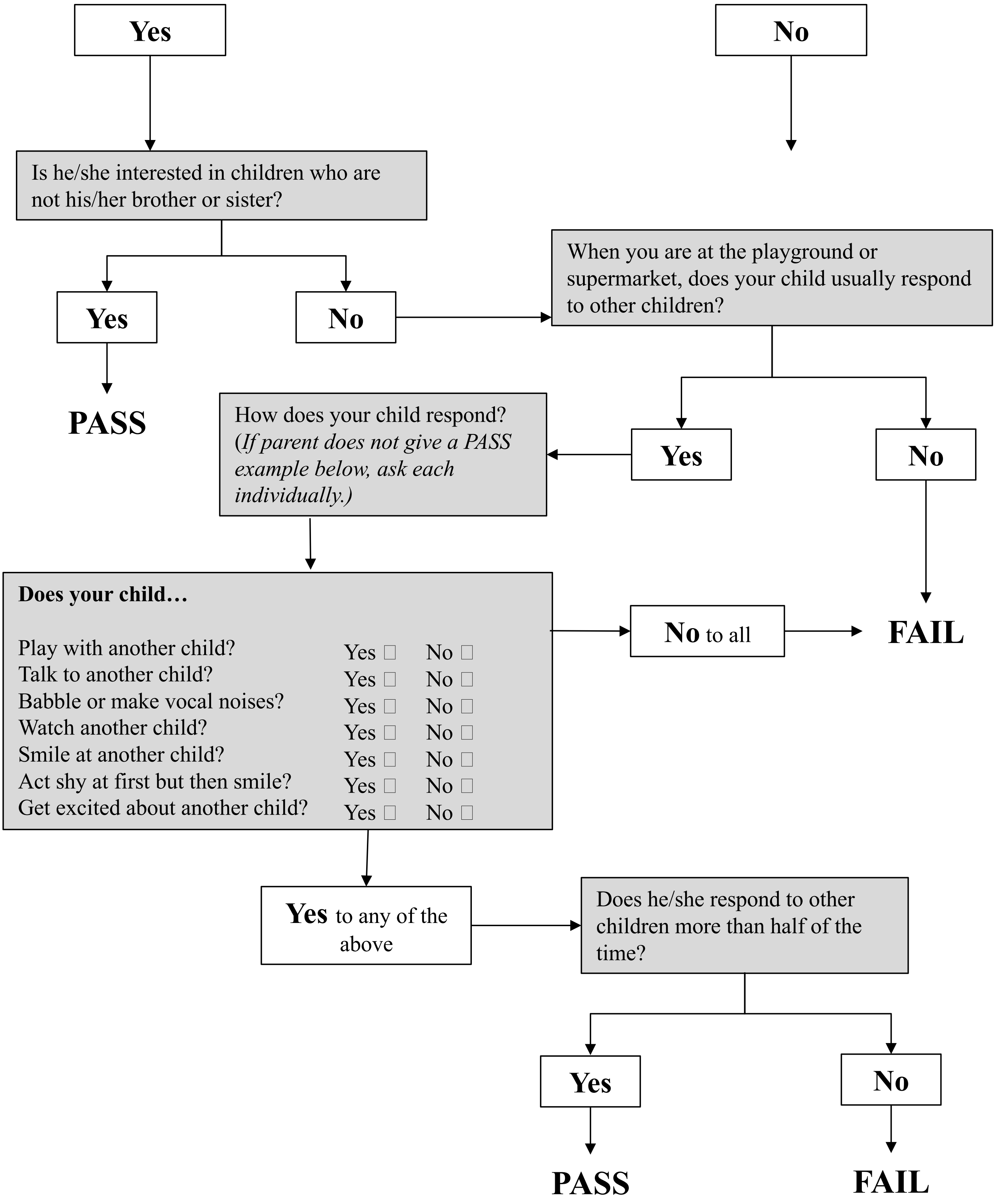

© 2009 Diana Robins, Deborah Fein, & Marianne Barton

9. Does ________ show you things by bringing them to you or holding them up for you to see? Not just to get help, but to share?

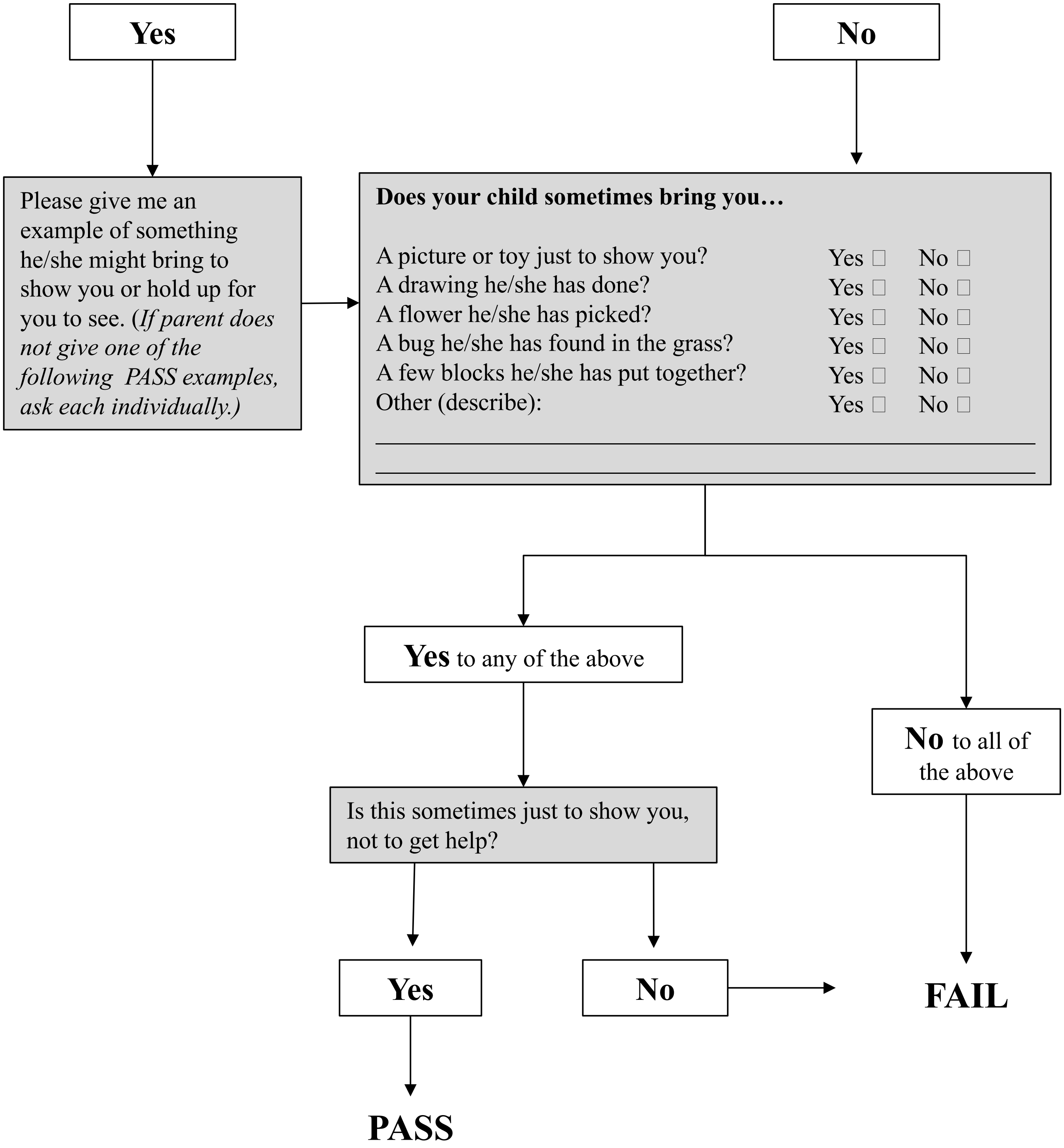

© 2009 Diana Robins, Deborah Fein, & Marianne Barton

10. Does ____________ respond when you call his/her name?

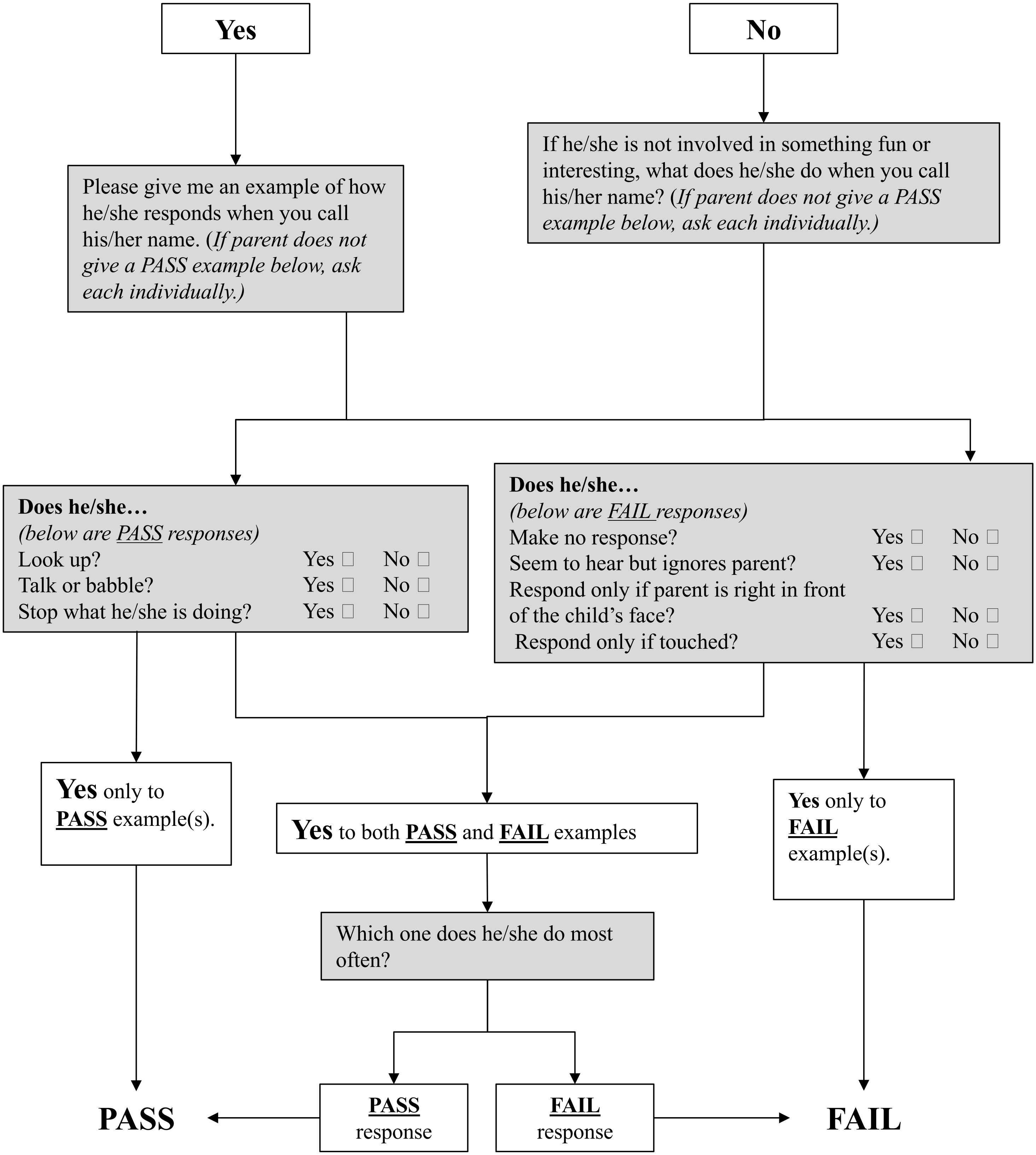

© 2009 Diana Robins, Deborah Fein, & Marianne Barton

11. When you smile at ____________, does he/she smile back at you?

Yes → **PASS**

No → What makes ____________ smile? (*If parent does not give a PASS example below, ask each individually.)*

Does your child…
(Below are PASS examples)

Smile when you smile?	Yes ☐	No ☐
Smile when you enter the room?	Yes ☐	No ☐
Smile when you return from being away?	Yes ☐	No ☐

Does he/she …
(Below are FAIL examples)

Always smile?	Yes ☐	No ☐
Smile at a favorite toy or activity?	Yes ☐	No ☐
Smile randomly or at nothing in particular?	Yes ☐	No ☐

Yes only to **PASS** example(s) → **PASS**

Yes to both **PASS** and **FAIL** examples → Which one does he/she do most often?

PASS response → **PASS**

FAIL response → **FAIL**

Yes only to **FAIL** example(s) → **FAIL**

© 2009 Diana Robins, Deborah Fein, & Marianne Barton

12. Does __________ get upset by everyday noises?

Yes

Does your child have a negative reaction to the sound of…

A washing machine?	Yes ☐	No ☐
Babies crying?	Yes ☐	No ☐
Vacuum cleaner?	Yes ☐	No ☐
Hairdryer?	Yes ☐	No ☐
Traffic?	Yes ☐	No ☐
Babies squealing or screeching?	Yes ☐	No ☐
Loud music?	Yes ☐	No ☐
Telephone/ doorbell ringing?	Yes ☐	No ☐
Noisy places such as a supermarket or restaurant?	Yes ☐	No ☐
Other (describe):	Yes ☐	No ☐

____

____

Yes to one or none → **PASS**

No → **PASS**

Yes to two or more

How does your child react to those noises? (*If parent does not give a PASS example below, ask each individually.)*

Does your child…
(Below are PASS responses)

Calmly cover his/her ears?	Yes ☐	No ☐
Tell you that he/she does not like the noise?	Yes ☐	No ☐

Does your child…
(Below are FAIL responses)

Scream?	Yes ☐	No ☐
Cry?	Yes ☐	No ☐
Cover his/her ears while upset?	Yes ☐	No ☐

Yes only to **PASS** example(s) → **PASS**

Yes to both **PASS** and **FAIL** examples

Which one does he/she do most often?

PASS response → **PASS**

FAIL response → **FAIL**

Yes only to **FAIL** example(s) → **FAIL**

© 2009 Diana Robins, Deborah Fein, & Marianne Barton

13. Does __________ walk?

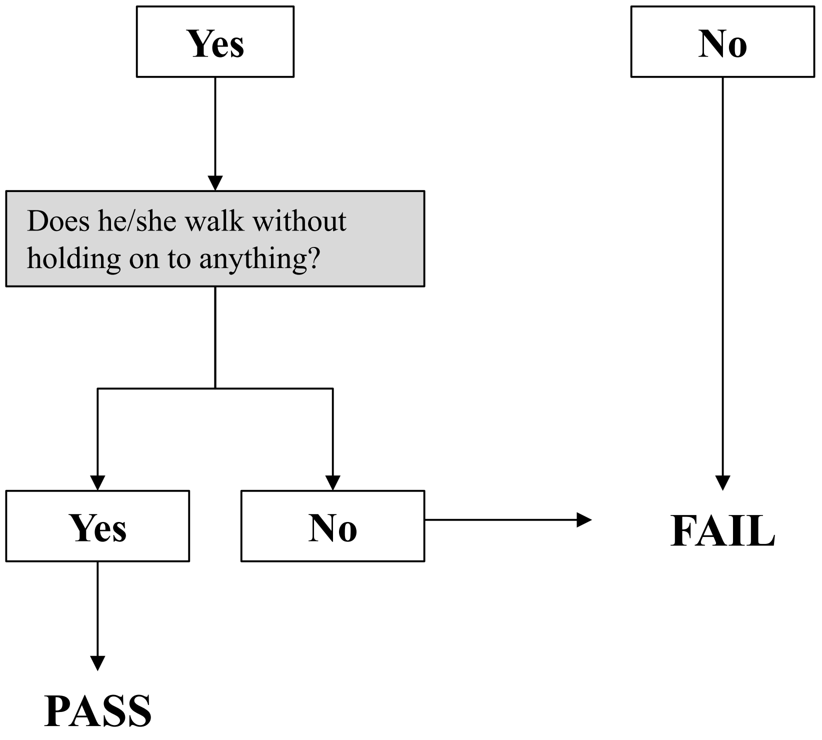

© 2009 Diana Robins, Deborah Fein, & Marianne Barton

14. Does _________ look you in the eye when you are talking to him/her, playing with him/her, or changing him/her?

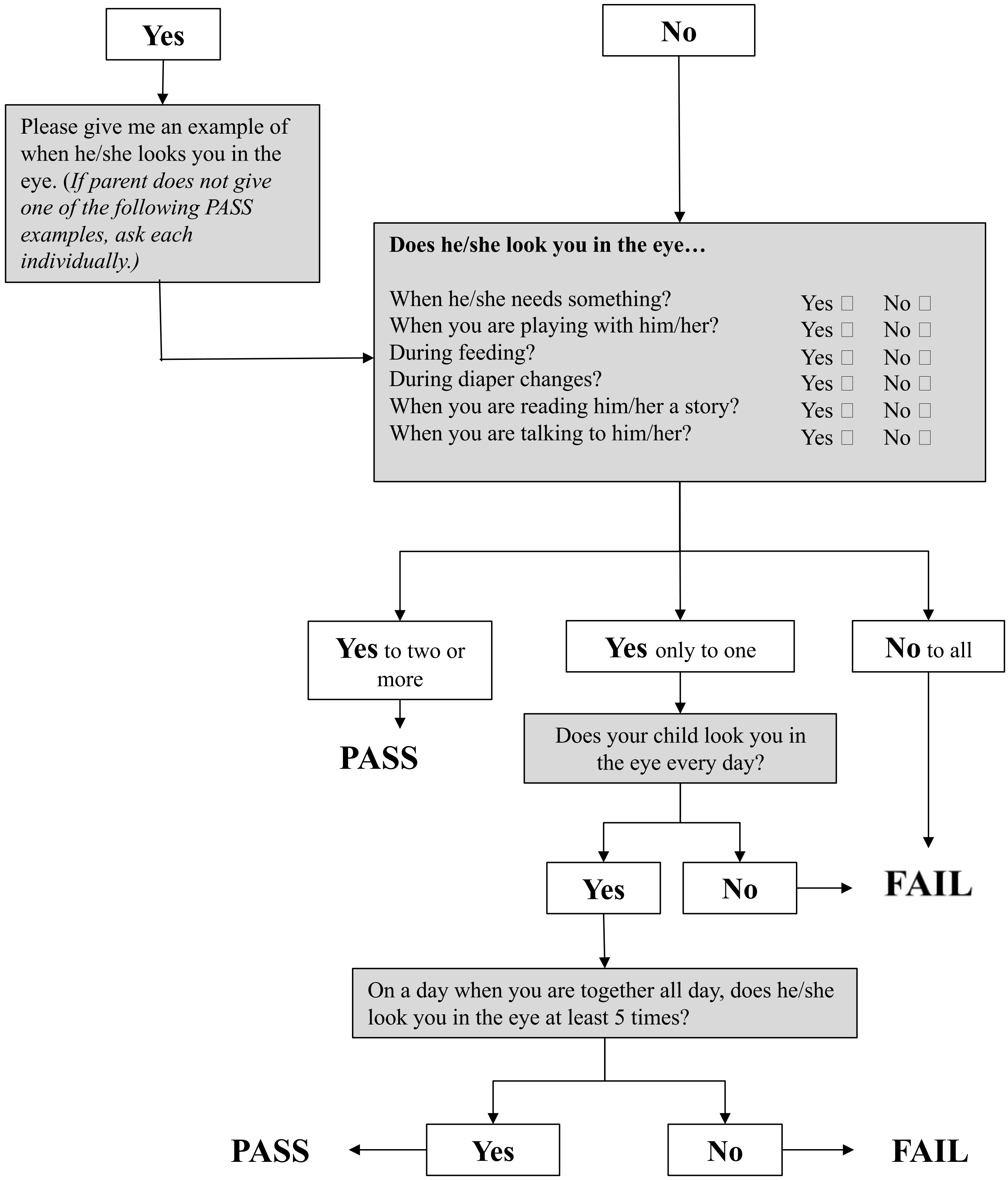

© 2009 Diana Robins, Deborah Fein, & Marianne Barton

15. Does __________ try to copy what you do?

Yes

Please give me an example of something he/she would try to copy. (*If parent does not give one of the following pass examples, ask each individually.)*

No

Does your child try to copy you if you…

Stick out your tongue?	Yes ☐	No ☐
Make a funny sound?	Yes ☐	No ☐
Wave good bye?	Yes ☐	No ☐
Clap your hands?	Yes ☐	No ☐
Put your fingers to your lips to signal "Shhh"?	Yes ☐	No ☐
Blow a kiss?	Yes ☐	No ☐
Other (describe):	Yes ☐	No ☐

____

____

Yes to two or more → **PASS**

Yes to one or none → **FAIL**

© 2009 Diana Robins, Deborah Fein, & Marianne Barton

16. If you turn your head to look at something, does ________ look around to see what you are looking at?

Yes → **PASS**

No →

What does he/she do when you turn to look at something? (*If parent does not give a PASS example below, ask each individually.)*

Does your child…
(*Below are PASS responses)*

Look toward the thing you are looking at?	Yes □	No □
Point toward the thing you are looking at?	Yes □	No □
Look around to see what you are looking at?	Yes □	No □

Does you child…
(Below are FAIL responses)

Ignore you?	Yes □	No □
Look at your face?	Yes □	No □

Yes only to **PASS** example(s) → **PASS**

Yes to both **PASS** and **FAIL** examples → Which one does he/she do most often?

- **PASS** response → **PASS**
- **FAIL** response → **FAIL**

Yes only to **FAIL** example(s) → **FAIL**

© 2009 Diana Robins, Deborah Fein, & Marianne Barton

17. Does __________ try to get you to watch him/her?

Yes

Please give me an example of how he/she would try to get you to watch him/her. (*If parent does not give a PASS example below, ask each individually.)*

No

Does he/she…

Say "Look!" or "Watch me!"?	Yes ☐	No ☐
Babble or make a noise to get you to watch what he/ she is doing?	Yes ☐	No ☐
Look at you to get praise or comment?	Yes ☐	No ☐
Keep looking to see if you are looking?	Yes ☐	No ☐
Other (describe):	Yes ☐	No ☐

____

____

Yes to any → **PASS**

Yes to none → **FAIL**

© 2009 Diana Robins, Deborah Fein, & Marianne Barton

18. Does _____________ understand when you tell him/her to do something?

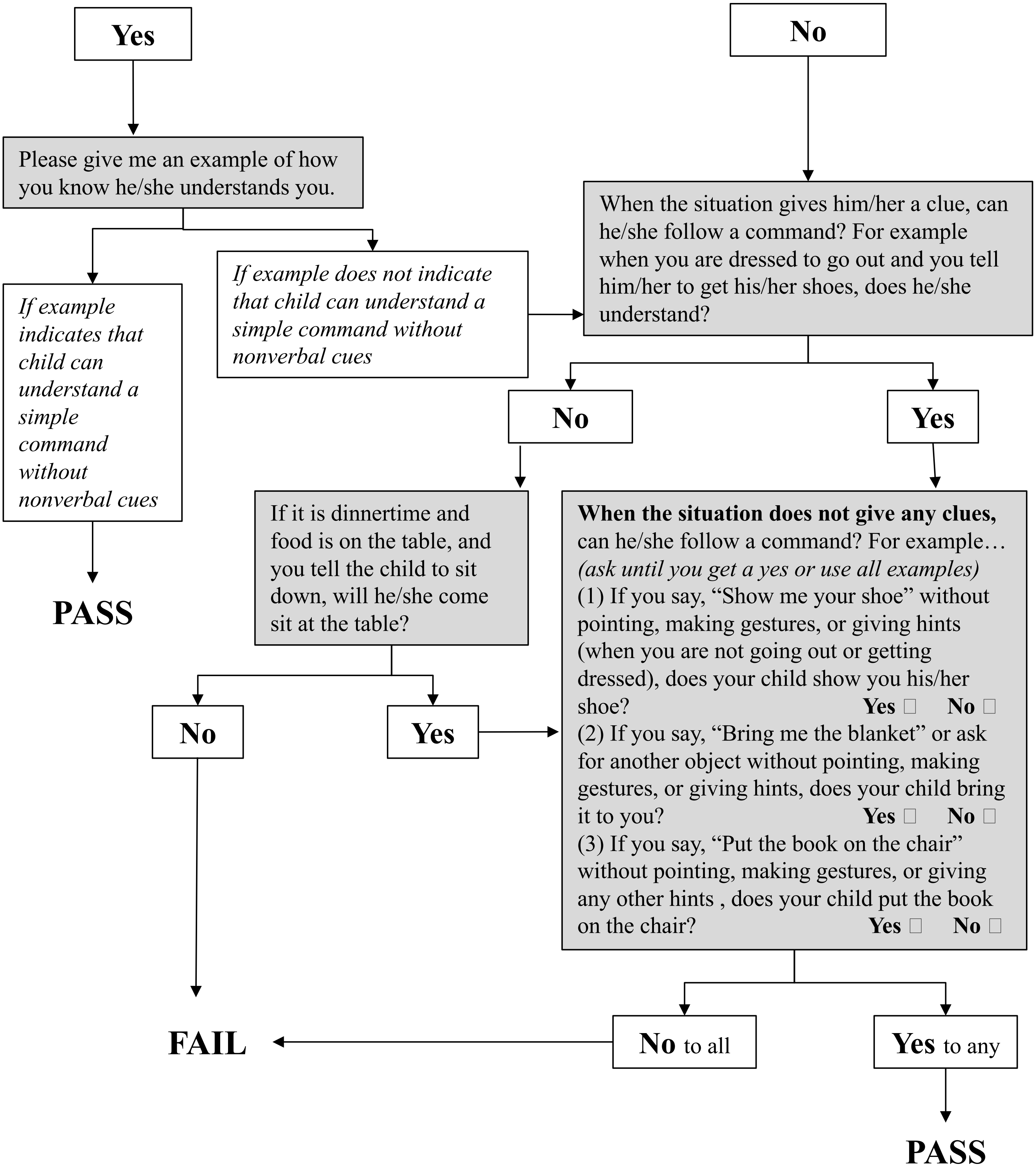

© 2009 Diana Robins, Deborah Fein, & Marianne Barton

19. If something new happens, does _________ look at your face to see how you feel about it?

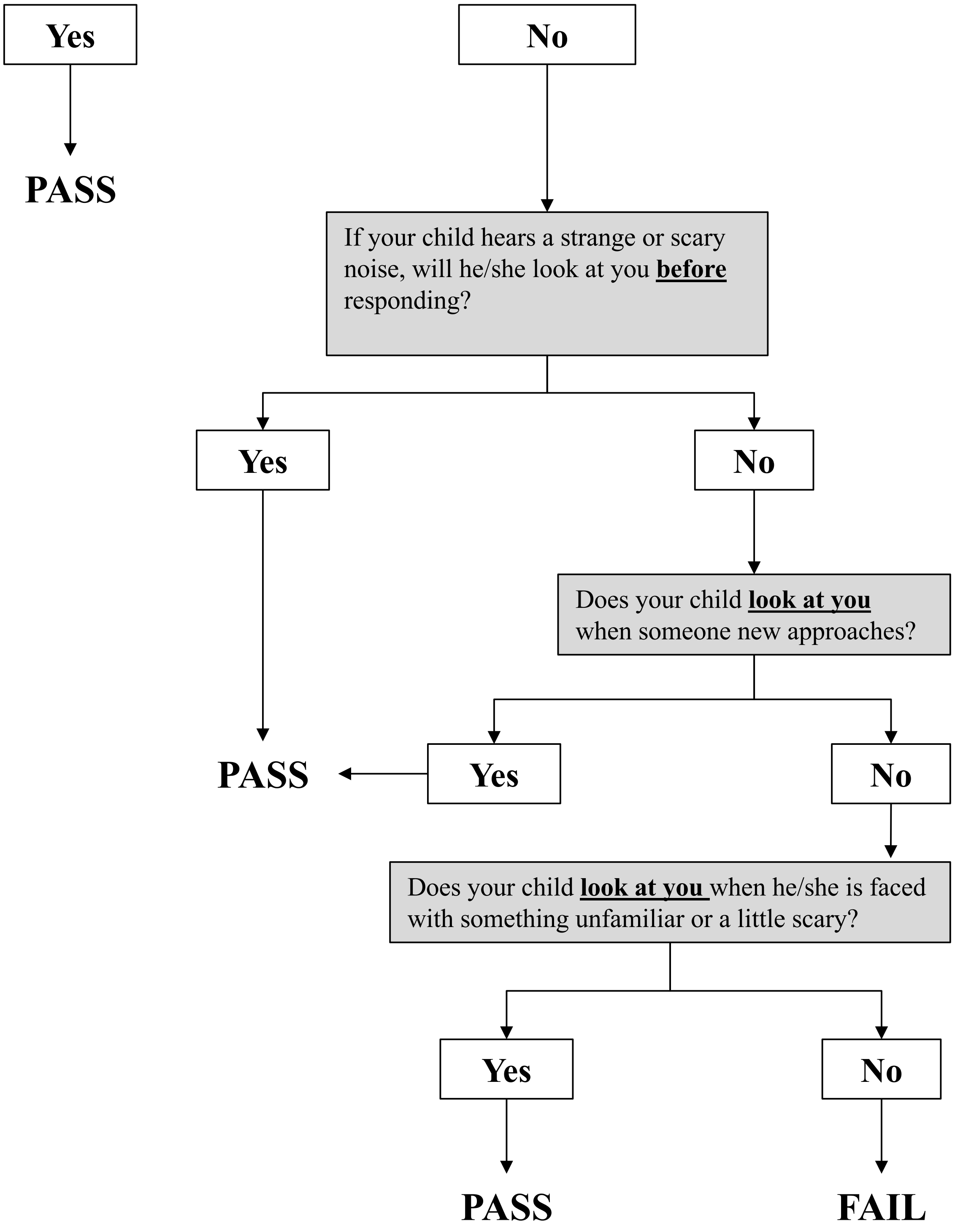

© 2009 Diana Robins, Deborah Fein, & Marianne Barton

20. Does ___________ like movement activities?

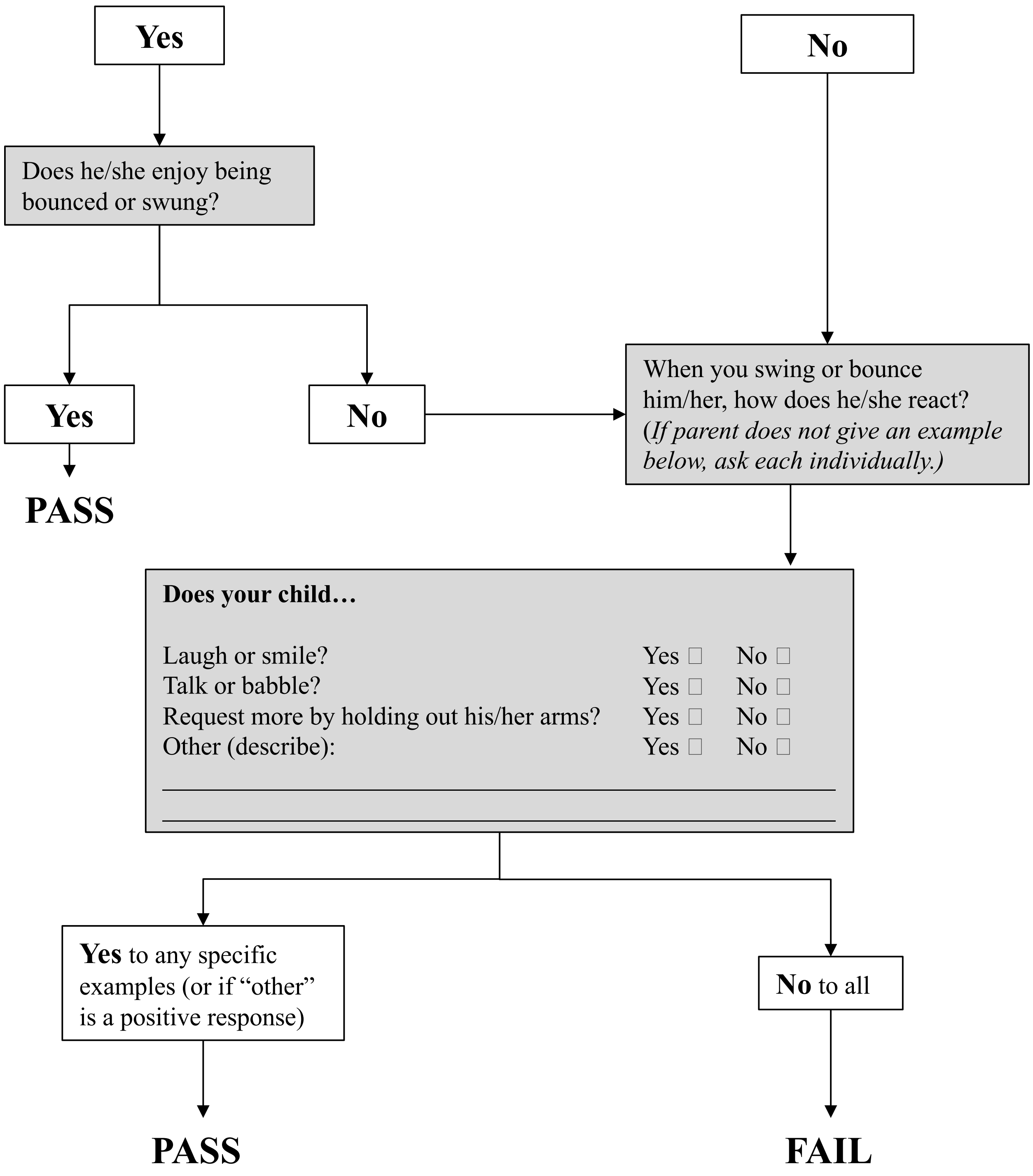

© 2009 Diana Robins, Deborah Fein, & Marianne Barton

Medical Record Abstraction

Study ID

____

Quality Assurance: Interview

Interviewer's Name

____
(First and last name)

Date of Interview

____

Result of Interview

◯ Complete
◯ Incomplete

If complete, specify

____

Reviewed by

____
(First and last name)

Date of Review

____

Result of review

◯ Complete
◯ Incomplete

Recommendations

____

Quality Assurance: Abstract

Abstractor Name

____
(First and last name)

Abstraction Date

____

Reviewed by

____
(First and last name)

Date of Review

____

Result of review

◯ Complete
◯ Incomplete

Recommendations

____

1. Prenatal Care	◯ No Prenatal Care<br>◯ Prenatal Care Outside BMC<br>◯ Prenatal Care at BMC<br>◯ Prenatal Care Both Outside and at BMC
Starts at week at BMC	__________________________
# of Documented Visits at BMC	__________________________
Rec. avail from week at BMC	__________________________
Starts at Week Outside BMC	__________________________
# Documented Visits Outside BMC	__________________________
Rec avail from week Outside BMC	__________________________
Starts at Week at BMC	__________________________
# Documented Visits BMC	__________________________
Rec avail from wk BMC	__________________________
Starts at week Outside BMC	__________________________
# Documented Visits Outside BMC	__________________________
Rec Avail from Wk Outside BMC	__________________________
2. Date of Last Menstrual Period	◯ Certain<br>◯ Uncertain<br>◯ Unknown
Please Specify LMP Date	__________________________
3a. Gravidity	__________________________
3b. Parity	__________________________
3c. TAB	__________________________

3d. SAB

____

4a. Gestational Age By LMP

____
(Decimal weeks by LMP)

4a. EDC by LMP

____

4b. Gestational Age by Ultrasound

____
(Decimal Weeks by Ultrasound)

4b. EDC by Ultrasound

____

4c. Gestational Age by Dubowitz at birth

____
(Decimal weeks by Dubowitz at birth)

4c. Dubowitz Total score

____

4d. Gestational Age by New Ballard Score

____
(Decimal weeks by New Ballard Score)

4d. New Ballard Score: Total score

____

5a. Apgars at 1 Minute

____

5b. Apgars at 5 Min

____

5c. Apgars at 10 Min

____

6. Birth Weight

____

6a. Type of Delivery

○ Vaginal
○ C/S

6b. Length of Ruptured of Membranes

○ Known
○ Unknown

Hours

____
(hours)

Minutes

____
(minutes)

6c. Clinical Presentation Before Delivery	◯ Uterine contraction as first sign of labor<br>◯ Rupture of Membrane without uterine contraction as first sign of labor: Water broke noted by mom<br>◯ Rupture of Membrane without uterine contraction as first sign of labor: Fern test positive<br>◯ Rupture of Membrane without uterine contraction as first sign of labor: Both by mom and fern test<br>◯ Both uterine contraction and rupture of membrane as first sign of labor<br>◯ Medical Induction(no Contraction, no ROM, to end pregnancy due to medical reasons)
Medical Induction	◯ Artificial Initiation of Labor<br>◯ No artificial Initiation of Labor
Artificial Initiation of Labor	☐ Postdate (>40 Weeks)<br>☐ Maternal Complications (eg, PIH)<br>☐ Fetal Distress/IUGR<br>☐ Other
Artificial Initiation of labor if Other	______________________
C/S: No artificial initiation of labor	☐ Elective C/S<br>☐ Repeat C/S<br>☐ Postdate (>40 weeks)<br>☐ Breech Presentation<br>☐ Pelvic-fetal Disproportion<br>☐ Maternal Complications (eg, PIH)<br>☐ Fetal Distress / IUGR<br>☐ Other
CS: No Artificial Initiation of Labor if other	______________________

8. Complications of the Index Pregnancy

8a. Preeclampsia	◯ No<br>◯ Mild<br>◯ Severe
8b. Eclampsia	◯ Yes<br>◯ No
8c. Chronic Hypertension	◯ Yes<br>◯ No
8d. Gestational Hypertension	◯ Yes<br>◯ No
8e. Placental Abruption	◯ Yes<br>◯ No
8f. Placenta Previa	◯ Yes<br>◯ No
8f. Incompetent Cervix	◯ Yes<br>◯ No

If yes, Suture placed	◯ Yes<br>◯ No
At week	________________________
8h. Diabetes	◯ No<br>◯ GDM<br>◯ DM
8i. Genital Tract Infections	◯ Yes<br>◯ No
8j. Urinary Tract Infections	◯ Yes<br>◯ No
8k. HEELP Syndrome	◯ Yes<br>◯ No
8l. Oligohydramnios	◯ Yes<br>◯ No
If yes, the lowest amniotic fluid level(< =5cm)	________________________
8m. Polyhydramnios	◯ Yes<br>◯ No
If yes, the highest amniotic fluid level (>=25)	________________________
8n. Meconium in Amniotic Fluid	◯ Yes<br>◯ No
8o. Any documented preterm non Braxton-Hicks contractions	◯ Yes<br>◯ No
8p. First Documented preterm contractions at	________________________<br>(weeks)
8q. Number of Documented Preterm Contraction Episodes	________________________
8r. Bed Rest	◯ Yes<br>◯ No
8r. Tocolysis	☐ None<br>☐ Magnesium Sulfate<br>☐ Beta2-adrenergic agents<br>☐ other
8r. Betamethazone	◯ None<br>◯ 1 Dose<br>◯ 2 or more doses

8r. IV Fluids	◯ Yes<br>◯ No
8r. Other	◯ Yes<br>◯ No
Specify Other	__________________________
8s. Fetal Fibronectin 1	__________________________
Fetal Fibronectin 1 Results	◯ Positive<br>◯ Negative<br>◯ Unknown
Fetal Fibronectin 2	__________________________
Fetal Fibronectin 2 Results	◯ Positive<br>◯ Negative<br>◯ Unknown
8t. Vaginal Bleeding	◯ Yes<br>◯ No
During first trimester	◯ Yes<br>◯ No
During 2nd trimester	◯ Yes<br>◯ No
During 3rd trimester	◯ Yes<br>◯ No
Preceding onset of labor	◯ Yes<br>◯ No
8u. Signs of Chorioamnionitis	◯ Yes<br>◯ No
Maternal Temperature >38c	◯ Yes<br>◯ No
Uterine tenderness	◯ Yes<br>◯ No
Foul smelling vaginal discharge or amniotic fluid	◯ Yes<br>◯ No
Maternal tarchycardia	◯ Yes<br>◯ No
Fetal tachycardia	◯ Yes<br>◯ No

Maternal white blood cell count >15,000

◯ Yes
◯ No

8v. Were any intrapartum antibiotics administered

◯ Yes
◯ No

If yes, specify intrapartum antibiotic administration: Check all that apply
If none administered check "None"

	None	One dose < 4 hours prior to delivery	One does >=4 hours prior to delivery	2 or more doses	Given, but time unknown
Ampicillin	◯	◯	◯	◯	◯
Clindamycin	◯	◯	◯	◯	◯
Gentamicin	◯	◯	◯	◯	◯
Penicillin	◯	◯	◯	◯	◯
Other Antibiotic 1	◯	◯	◯	◯	◯
Other Antibiotic 2	◯	◯	◯	◯	◯

If other, specify antibiotic 1

____

If other, specify antibiotic 2

____

9. Amniocentesis

◯ Yes
◯ No

If yes, what was the result

◯ Normal
◯ Abnormal
◯ Unknown

If Abnormal, specify

____

10. Number of Prenatal Ultrasounds

____

Please specify any abnormal results

____

U/S gestational week at which the FIRST U/S was performed

____
(2 decimal places)

LMP gestational week at which the FIRST U/S was performed

____
(2 Decimal places)

11. Placenta Sent for Pathology

◯ Yes
◯ No

12. Does the mother smoke or drink or take drug	◯ Yes<br>◯ No
Pre pregnancy Smoking	◯ Yes<br>◯ No
Type of cigarette	☐ Cigarette<br>☐ E-Cigarette
Number of cigs per day	________________________
Pre pregnancy Alcohol	◯ Yes<br>◯ No
Number drinks/week	________________________
Pre pregnancy Drug Type 1	________________________
#use/wk	________________________
Pre pregnancy Drug Type 2	________________________
#use/wk	________________________
Pre pregnancy Drug Type 3	________________________
#use/wk	________________________
Pre pregnancy Drug Type 4	________________________
#use/wk	________________________
1st Trimester Smoking	◯ Yes<br>◯ No
Type of cigarette	☐ Cigarette<br>☐ E-Cigarette
Number of sigs per day	________________________
1st Trimester Alcohol	◯ Yes<br>◯ No

Number drinks/week	______________________
1st Trimester Drug type 1	______________________
#use/wk	______________________
1st Trimester Drug type 2	______________________
#use/wk	______________________
1st Trimester Drug type 3	______________________
#use/wk	______________________
1st Trimester Drug type 4	______________________
#use/wk	______________________
2nd trimester Smoking	◯ Yes<br>◯ No
Type of cigarette	☐ Cigarette<br>☐ E-Cigarette
#cigs/day	______________________
2nd Trimester Alcohol	◯ Yes<br>◯ No
#drinks/week	______________________
2nd trimester drug type 1	______________________
#use/wk 1	______________________
2nd trimester drug type 2	______________________
#use/wk 2	______________________

2nd trimester drug type 3	__________________________
#use/wk 3	__________________________
2nd trimester drug type 4	__________________________
#use/wk 4	__________________________
3rd trimester smoking	◯ Yes<br>◯ No
Type of cigarette	☐ Cigarette<br>☐ E-Cigarette
#cigs/days	__________________________
3rd trimester alcohol	◯ Yes<br>◯ No
#drinks/wk	__________________________
3rd trimester Drug Type 1	__________________________
#use/wk	__________________________
3rd trimester Drug Type 2	__________________________
#use/wk	__________________________
3rd trimester Drug Type 3	__________________________
#use/wk	__________________________
3rd trimester Drug Type 4	__________________________
#use/wk	__________________________
15. Mother Received anesthesia	◯ Yes<br>◯ No

17. CBC	◯ Yes<br>◯ No
18. Mother Transfused	◯ Yes<br>◯ No
If yes, Date Transfused:	______________________
Transfusion Time: 24 Hour Clock	______________________
Transfusion, number of units	______________________
19. Amniotic Fluid Culture	◯ Yes<br>◯ No
If Yes, specify pathogen	______________________
20. Urine Culture	◯ Yes<br>◯ No
21a. Urinary Tract Infection during 1st and 2nd trimester (< 27 weeks gestation)	◯ 1. Neither Reported or indicated by labs<br>◯ 2. Pt Report Only<br>◯ 3. (+) urine Culture only or chart mentioned in problem list<br>◯ 4. Both 2 and 3<br>◯ 5. (+) urine culture but < 50,000 colonies<br>◯ 6. Unable to determine
21b. Urinary Tract Infection during 3rd trimester (>= 27 weeks gestation)	◯ 1. Neither Reported or indicated by labs<br>◯ 2. Pt Report Only<br>◯ 3. (+) urine Culture only or chart mentioned in problem list<br>◯ 4. Both 2 and 3<br>◯ 5. (+) urine culture but < 50,000 colonies<br>◯ 6. Unable to determine

Any significant past medical history: USE MEDICAL RECORD INFO ONLY

Asthma	◯ Yes<br>◯ No
If Yes	◯ During Pregnancy<br>◯ Before Pregnancy<br>◯ Both
Hyperthyroidism	◯ Yes<br>◯ No
If Yes	◯ During Pregnancy<br>◯ Before Pregnancy<br>◯ Both

Hypothyroidism	◯ Yes<br>◯ No
If Yes:	◯ During Pregnancy<br>◯ Before Pregnancy<br>◯ Both
Endometriosis	◯ Yes<br>◯ No
If yes:	◯ During Pregnancy<br>◯ Before Pregnancy<br>◯ Both
Uterine Myoma	◯ Yes<br>◯ No
If yes	◯ During Pregnancy<br>◯ Before Pregnancy<br>◯ Both
Uterine Malformation	◯ Yes<br>◯ No
If yes:	◯ During Pregnancy<br>◯ Before Pregnancy<br>◯ Both
Pelvic Inflammatory Disease	◯ Yes<br>◯ No
If yes	◯ During Pregnancy<br>◯ Before Pregnancy<br>◯ Both
Abnormal PAP Smear	◯ Yes<br>◯ No
If yes	◯ During Pregnancy<br>◯ Before Pregnancy<br>◯ Both
Polycystic Ovaries	◯ Yes<br>◯ No
If yes	◯ During Pregnancy<br>◯ Before Pregnancy<br>◯ Both
Abdominal Operation	◯ Yes<br>◯ No
If yes	◯ During Pregnancy<br>◯ Before Pregnancy<br>◯ Both

Anemia	◯ Yes<br>◯ No
If yes	◯ During Pregnancy<br>◯ Before Pregnancy<br>◯ Both
Malignant Tumor	◯ Yes<br>◯ No
If yes	◯ During Pregnancy<br>◯ Before Pregnancy<br>◯ Both
Tuberculosis	◯ Yes<br>◯ No
If yes	◯ During Pregnancy<br>◯ Before Pregnancy<br>◯ Both
Cardiovascular Disease	◯ Yes<br>◯ No
If yes	◯ During Pregnancy<br>◯ Before Pregnancy<br>◯ Both
High Blood Pressure	◯ Yes<br>◯ No
If Yes	◯ During Pregnancy<br>◯ Before Pregnancy<br>◯ Both
Auto-immune Disease	◯ Yes<br>◯ No
If Yes	◯ During Pregnancy<br>◯ Before Pregnancy<br>◯ Both
Drug Allergy	◯ Yes<br>◯ No
If yes	◯ During Pregnancy<br>◯ Before Pregnancy<br>◯ Both
Seizure Disorder	◯ Yes<br>◯ No
If yes	◯ During Pregnancy<br>◯ Before Pregnancy<br>◯ Both

Gestational Diabetes	◯ Yes<br>◯ No
If yes	◯ During Pregnancy<br>◯ Before Pregnancy<br>◯ Both
Diabetes Mellitus	◯ Yes<br>◯ No
If yes	◯ During Pregnancy<br>◯ Before Pregnancy<br>◯ Both
Infertility (Unable to get pregnant after one year of unprotected intercourse)	◯ Yes<br>◯ No
Age Diagnosed	__________________________________
Any Treatment:(check all that apply)	☐ Medications<br>☐ Intrauterine Insemination(IUI)<br>☐ In-vitro-Fertilization<br>☐ Others
If Other	__________________________________
Mother is allergic to food or environmental allergens	◯ Yes<br>◯ No
Cow's Milk	◯ Yes<br>◯ No
Egg	◯ Yes<br>◯ No
Peanut	◯ Yes<br>◯ No
Walnut	◯ Yes<br>◯ No
sesame	◯ Yes<br>◯ No
Shellfish	◯ Yes<br>◯ No
Fish	◯ Yes<br>◯ No
Soy	◯ Yes<br>◯ No

Wheat	◯ Yes<br>◯ No
Cat	◯ Yes<br>◯ No
Dog	◯ Yes<br>◯ No
Cockroach	◯ Yes<br>◯ No
Dust Mites	◯ Yes<br>◯ No
Molds	◯ Yes<br>◯ No
Others	◯ Yes<br>◯ No
If others, specify	______________________
Eczema	◯ Yes<br>◯ No
Seasonal Allergy (or Hay Fever)	◯ Yes<br>◯ No
If Others, specify	______________________

BABY INFORMATION

Date of Delivery	______________________<br>(Month and Year only)
Time of delivery	______________________
Baby Gender	◯ Male<br>◯ Female
Length	______________________<br>(cm)
Head Circumference	______________________<br>(cm)
Birth Defect Present	◯ Yes<br>◯ No

Was birth defect diagnosed	◯ Prenatally<br>◯ Perinatally<br>◯ Unknown
Type of Birth Defect	☐ Anecephalus<br>☐ Cleft lib/palate<br>☐ Club foot<br>☐ congenital hip dislocation<br>☐ Diaphragmatic hernia<br>☐ Down Syndrome<br>☐ Gastroschisis<br>☐ Hydrocephalus<br>☐ Hypospadius<br>☐ Microcephalus<br>☐ Omphalocele<br>☐ Other Cardiac<br>☐ Other chromosomal<br>☐ Other CNS<br>☐ Other GI<br>☐ Other musculoskeletal<br>☐ Other urogenital<br>☐ Other(specify)<br>☐ Patent ductus ateriosus<br>☐ Polydactyly<br>☐ Rectal atresia/stenosis<br>☐ Renal agenesis<br>☐ Spina bifida<br>☐ Syndactyly<br>☐ Unknown
If Other, specify	______________________________

BMC Health System

THRIVE SCREENER

Addressing Social Determinants of Health

Place Patient Sticker Here

Please fill this out and give to the medical assistant when you are called into the exam room. Your answers will help your care team take better care of your health and connect you with resources. Thank you!

Please check "✓" your answers:

I am a ☐ Patient ☐ Parent / Caregiver

What is your living situation today?
- ❍ I have a steady place to live
- ❍ I have a place to live today, but I am worried about losing it in the future
- ❍ I do not have a steady place to live (I am temporarily staying with others, in a hotel, in a shelter, living outside on the street, on a beach, in a car, abandoned building, bus or train station, or in a park)

Question	Answer
**Within the past 12 months, the food you bought just didn't last and you didn't have money to get more.**	❍ Often true<br>❍ Sometimes true<br>❍ Never true
**Within the past 12 months, you worried whether your food would run out before you got money to buy more.**	❍ Often true<br>❍ Sometimes true<br>❍ Never true
**Is this an emergency, do you need food for tonight?**	❍ Yes ❍ No
**Do you have trouble paying for medicines?**	❍ Yes ❍ No
**Do you have trouble getting transportation to medical appointments?**	❍ Yes ❍ No
**Do you have trouble paying your heating or electricity bill?**	❍ Yes ❍ No
**Do you have trouble taking care of a child, family member or friend?**	❍ Yes ❍ No
**Do you have trouble with day-to-day activities such as bathing, preparing meals, shopping, managing finances, etc.?**	❍ Yes ❍ No
**Are you currently unemployed and looking for a job?**	❍ Yes ❍ No
**Are you interested in more education?**	❍ Yes ❍ No

Please check "✓" the resources you want help with:

Housing / Shelter	Food	Paying for Medicine	Transport	Utilities	Childcare	Care for elder or disabled	Daily Support	Job search / training	Education
❍	❍	❍	❍	❍	❍	❍	❍	❍	❍

❍ I do not want to answer these questions

© 2017 Boston Medical Center

Pandemic Health and Resources Questionnaire

Hello,

Thanks for participating in Children's Health Study! This survey is about you and your family's experience with healthcare and other services during the months from March 2020 through August 2020 during the Coronavirus Pandemic (COVID19).

Please answer the questions based on your experiences from March 2020-August 2020 (6) months throughout the quarantine period.

The questionnaire should take approximately 15-20 minutes to fill out. The Visit ID and interviewer fields are already filled out, so you just need to answer the remaining questions.

Once you have answered all the questions please press submit.

Please contact colleen.pearson@bmc.org with any questions.

Thank you!

1. Visit ID __________________________________

2. Interview Date __________________________________

Fecha

3. Interviewer Name __________________________________
(First and last name)

If you need help with resources because of hardships you or your family may be experiencing during COVID-19, please visit: Si usted o su familia necesita ayuda con dificultades durante la pandemia por favor hace click aqui: https://www.bmc.org/here-for-you/resources/community-resources

For the following questions please consider the events from March 2020 through August 2020 (6 month period) when choosing your answers. When a question asks about "your child" please answer about your child who is participating in this study.

Para estas preguntas, piensa en los eventos desde marzo 2020 hasta agosto 2020 cuando elige sus respuestas. Cuando una preguna dice "su hijo/a" es sobre so hijo/a quien participa en este estudio.

4. Including yourself, how many people do you live with?

Please answer based on those living with you from March 2020 until August 2020 (6 months). The people did not have to live with you the entire 6 months.

Con cuantas personas vive usted? (incluyendo usted misma)

Por favor contesta con las personas que viven con usted durante marzo 2020-agosto 2020. No tienen que vivir con usted por todo de las 6 meses.

____

5. Has anyone you lived with from March 2020 through August 2020 (6 months) been TESTED for the Coronavirus (Covid 19)? (had a nasal swab to find out if they have the Coronavirus (Covid 19)

Alguien con que usted vive ha tomado la prueba de Covid 19? (recibe un hisopo nasal para determinar si tiene el Coronavirus)
1. Sí
2. No
3. Prefiero no responder
4. No se

○ Yes
○ No
○ Prefer not to answer
○ Don't know

5a. If yes, who was tested for the Coronavirus (Covid 19)?

De ser asi, quien?
1. adulto(s)
2. Nino(s)
3. yo misma(o)
4. prefiero no responder

☐ Adult(s)
☐ Children
☐ Myself
☐ Prefer not to answer

5b. Please check off the age group(s) of anyone in your home who was tested for the Coronavirus (Covid 19).

Check all that apply

Por favor marque el grupo/ los grupos de edad de las personas probadas para Covid 19 en su casa

Marque todas las que apliquen

☐ Less than 1 year old
☐ 1-4 years old
☐ 5-9 years old
☐ 10-14 years old
☐ 15-24 years old
☐ 25-34 years old
☐ 35-44 years old
☐ 45-54 years old
☐ 55-64 years old
☐ 65-74 years old
☐ 75-84 years old
☐ 85 years and over

6. Did anyone living in your household from March 2020 through August 2020 test POSITIVE for the Coronavirus (Covid 19)?

Alguien quien vive en su casa dar positivo por Covid 19?
1. Sí
2. No
3. Prefiero no responder
4. No se

○ Yes
○ No
○ Prefer not to answer
○ Don't know

6a. If yes, please check off the age group(s) of who from your home tested POSITIVE for the Coronavirus (Covid 19).<br><br>Check all that apply<br><br>De ser asi, quien?<br><br>Marque todas las que apliquen	☐ Less than 1 year old<br>☐ 1-4 years old<br>☐ 5-9 years old<br>☐ 10-14 years old<br>☐ 15-24 years old<br>☐ 25-34 years old<br>☐ 35-44 years old<br>☐ 45-54 years old<br>☐ 55-64 years old<br>☐ 65-74 years old<br>☐ 75-84 years old<br>☐ 85 years or older
7. Has anyone you lived with from March 2020-August 2020 been pregnant during the Coronavirus (Covid 19) Pandemic?<br><br>Alguien quien vive en su casa desde marzo 2020 hasta agosto 2020 tiene un embarazo durante la pandemia de Covid 19?<br>1. Sí<br>2. No<br>3. Prefiero no responder<br>4. No se	◯ Yes<br>◯ No<br>◯ Prefer not to answer<br>◯ Don't know
8. Have you or your child had any healthcare visits by video or phone from the period of March 2020 through August 2020 because of the Coronavirus (Covid 19) Pandemic?<br><br>Usted o su hijo/a tiene alguna cita médica por una llamada o videollamada desde marzo 2020 hasta agosto 2020 a causa de la pandemia?<br>1. Sí- yo<br>2. Sí- mi hijo/a<br>3. Sí mi hijo/a y yo<br>4. No<br>5. Prefiero no responder	◯ Yes-me<br>◯ Yes- my child<br>◯ Yes- both me and my child<br>◯ No<br>◯ Prefer not to answer
9. Did you or your child miss any health care visits during the period from March 2020 through August 2020 because of the Coronavirus (Covid19) Pandemic?<br><br>Falta usted o su hijo/a alguna cita medica desde marzo 2020 hasta agosto 2020 a causa de la pandemia de Covid 19?<br>1. Sí- yo<br>2. Sí- mi hijo/a<br>3. Sí mi hijo/a y yo<br>4. No<br>5. Prefiero no responder	◯ Yes- me<br>◯ Yes- my child<br>◯ Yes- both me and my child<br>◯ No<br>◯ Prefer not to answer

9a. If you or your child missed any healthcare visits from March through August 2020, please check what type(s) of visits were missed (Check all that apply)

Si usted o su hijo/a falta una cita medica desde marzo 2020 hasta agosto 2020 por favor marque que tipo(s) (Marque todas que apliquen)
1. atención primaria para adultos
2. atención primaria pediátrica
3. pediatría del desarrollo
4. la neurología
5. la psiquiatría o la psicología
6. la endocrinología
7. la oftalmóloga
8. gastrointestinal
9. la hematología
10. la cardiología
11. tocoginecología
12. la dermatología
13. la odontología
14. otro

☐ Adult Primary Care
☐ Pediatric Primary Care
☐ Developmental/Behavioral Pediatrics
☐ Neurology
☐ Psychiatry/Psychology
☐ Endocrinology
☐ Eye appointment
☐ GI
☐ Hematology
☐ Cardiology
☐ OB/GYN
☐ Dermatology
☐ Dental Clinic
☐ Other

If "Other", please specify

Si es "otro" que tipo?

____

9b. How many health care visits did YOU miss?

Cuantas citas médicas falta USTED?

____

9c. How many health care visits did YOUR CHILD miss?

Cuantas citas médicas falta SU HIJO/A?

____

9d. Did any of the following reasons contribute to you or your child missing any health care appointments from March 2020 through August 2020? (Check all that apply)

Por que usted o su hijo/a falta una cita? (Marque todos que apliquen)
1. La clínica estaba cerrada por la pandemia de Covid 19
2. La clínica estaba abierta con citas limitadas por la pandemia de Covid 19
3. Padre o nino/a estaba preocupado para ir a la clínica por la pandemia de Covid 19
4. Mi niño/a no tenía seguro medico/ su seguro médico cambiaba
5. Alguien en el hogar estaba enferma
6. Alguien en el hogar estaba en contacto con alguien que estaba enferma
7. Ninguno de los arriba
8. Prefiero no responder

☐ Health care provider's location was closed due to the Coronavirus pandemic (Covid19)
☐ Health care provider's location was open but had limited appointments due to the Coronavirus pandemic (Covid19)
☐ Parent, adult caregiver, or child was concerned about going to the health care provider's location due to the Coronavirus pandemic (Covid19)
☐ My child no longer had health insurance or had a change in health insurance
☐ Someone in the household was ill
☐ Someone in the household had been in contact with someone who was ill
☐ None of the above
☐ Prefer not to answer

10. Did any of the following events happen in your household as a result of the Coronavirus pandemic (Covid19)? (Check all that apply)

Occure alguno de estos eventos a causa de la pandemia de Covid 19? (Marque todos que apliquen)

1. La esuela o la guardería de mi niño/a estaba cerrado por 2 semanas o más
2. Niño/a estaba seperado de padre o cuidador por 2 semanas o mas
3. Un adulto en el hogar perdió su empleo o no trabajo
4. Un adulto trabajaba fuera de casa
5. Alguien en el hogar hospitalizó a causa del coronavirus (covid 19)
6. Alguien en el hogar murrió del coronavirus (Covid 19)
7. Ninguno de los arriba
8. Prefiero no responder

- ☐ Child's school, daycare, or other child care arrangement was closed or unavailable for 2 weeks or longer
- ☐ Child was separated from a parent or adult caregiver for 2 weeks or longer
- ☐ At least one adult in the household lost a job or was unable to work
- ☐ At least one adult in the household worked outside the home
- ☐ A household member was hospitalized due to the Coronavirus (Covid19)
- ☐ A household member died from the Coronavirus (Covid19)
- ☐ None of the above
- ☐ Prefer not to answer

---

11. Has this child's school building, daycare, or other child care arrangement been closed at any time as a result of the Coronavirus pandemic (Covid19)?

Estaba cerrada la escuela o guarderia de este niño/a a causa de la pandemia del coronavirus (covid 19)?
1. Sí
2. No
3. No se aplica
4. Prefiero no responder

- ◯ Yes
- ◯ No
- ◯ N/A (Does not apply to me)
- ◯ Prefer not to answer

11a. If yes, for how long was the child's school or childcare arrangement been closed? (in months)
For example, 3 months

De ser asi, por cuanto tiempo? (en meses)

____

11b. If less than one month, for how long was the child's school or childcare arrangement been closed? (in weeks)

For example, 2 weeks.

Si menos de un mes, por cuanto tiempo? (en semanas)

____

This section of the questionnaire is about resources and your access to food before and during the Coronavirus pandemic (Covid19) from March 2020-August 2020. We are looking to better understand the hardships that were created by the Coronavirus pandemic (Covid19). Please consider the past 12 months when answering these questions and answer BEFORE the Coronavirus (Covid19) period March-August 2020 and the 6 months before the Coronavirus (Covid19) pandemic appeared or roughly last September 2019 through February 2020.

BEFORE CORONAVIRUS PANDEMIC (COVID19) USE SEPTEMBER 2019-FEBRUARY 2020
DURING CORONAVIRUS PANDEMIC (COVID19) USE TIME PERIOD MARCH 2020-AUGUST 2020

Esta sección del cuestionario es sobre sus recursos y su acceso a la comida antes de y durante la pandemia del Coronavirus desde marzo 2020 hasta agosto 2020. Queremos entender los dificultades creado por la pandemia del Coronavirus (Covid 19). Por favor, piensa en los últimos 12 meses cuando elige sus respuestas.

Antes de la pandemia del Coronavirus (Covid 19)- desde septiembre 2019 hasta febrero 2020

Durante la pandemia del Coronavirus (Covid 19)- desde marzo 2020 hasta agosto 2020

---

12. In the last 12 months, the food that was purchased for my household just didn't last, and we didn't have money to get more. (Please check all that apply)

Durante los últimos 12 meses, la comida que compramos en mi hogar no duró y no tenemos dinero para comprar mas. (Marque todos que apliquen)
1. Frecuentemente verdad antes de la pandemia
2. A veces verdad antes de la pandemia
3. Frecuentemente verdad durante la pandemia
4. A veces verdad durante la pandemia
5. Nunca es verdad
6. Prefiero no responder

☐ Often true before the pandemic
☐ Sometimes true before the pandemic
☐ Often true during the pandemic
☐ Sometimes true during the pandemic
☐ Never true
☐ Prefer not to answer

---

13. In my household during the last 12 months, we could not afford to eat balanced meals. (Please check all that apply)

En mi hogar, durante los últimos 12 meses, no podemos comprar comidas balanceadas. (Marque todos que apliquen)
1. Frecuentemente verdad antes de la pandemia
2. A veces verdad antes de la pandemia
3. Frecuentemente verdad durante la pandemia
4. A veces verdad durante la pandemia
5. Nunca es verdad
6. Prefiero no responder

☐ Often true before the pandemic
☐ Sometimes true before the pandemic
☐ Often true during the pandemic
☐ Sometimes true during the pandemic
☐ Never true
☐ Prefer not to answer

---

14. During the last 12 months, have you or other adults in your household cut the size of your meals or skip meals because there wasn't enough money for food? (Please check all that apply)

Durante los últimos 12 meses necesita usted o otros adultos en su casa come menos durante comidas o falta comidas porque no hay suficiente dinero para comida? (Marque todos que apliquen)
1. Sí, antes de la pandemia
2. Sí, durante la pandemia
3. No
4. Prefiero no responder

☐ Yes, before the pandemic
☐ Yes, during the pandemic
☐ No
☐ Prefer not to answer

14a. If yes, how often did you or other adults in your household cut the size of your meals or skip meals because there wasn't enough money for food? (Please check all that apply)<br><br>De ser así con que frecuencia come menos durante comidas o falta comidas? (Marque todos que apliquen)<br>1. Casi todos los meses antes de la cuarentena<br>2. Unos meses antes de la cuarentena<br>3. Un par de veces antes de la cuarentena<br>4. Casi todos los meses durante la cuarentena<br>5. Unos meses durante la cuarentena<br>6. Un par de veces durante la cuarentena<br>7. No se<br>8. Prefiero no responder	☐ Almost every month before quarantine<br>☐ Some months before quarantine<br>☐ Once or twice before quarantine<br>☐ Almost every month during quarantine<br>☐ Some months during quarantine<br>☐ Once or twice during quarantine<br>☐ Not sure<br>☐ Prefer not to answer
15. In the last 12 months, did you ever eat less than you felt you should because there wasn't enough money to buy food? (Please check all that apply.)<br><br>Durante los últimos 12 meses comía menos porque no había suficiente dinero para comprar comida? (Marque todos que apliquen)<br>1. Sí, antes de la pandemia<br>2. Sí, durante la pandemia<br>3. No<br>4. Prefiero no responder	☐ Yes, before the pandemic<br>☐ Yes, during the pandemic<br>☐ No<br>☐ Prefer not to answer
16. In the last 12 months, were you ever hungry but didn't eat because you couldn't afford enough food? (Please check all that apply)<br><br>Durante los últimos 12 meses, tenía hambre pero no comer porque no podía comprar comida? (Marque todos que apliquen)<br>1. Sí, antes de la pandemia<br>2. Sí, durante la pandemia<br>3. No<br>4. Prefiero no responder	☐ Yes, before the pandemic<br>☐ Yes, during the pandemic<br>☐ No<br>☐ Prefer not to answer
17. Who completed this form?<br><br>Quien completo el formulario?<br>1. La madre<br>2. Niño/a adulto/a	○ Mother<br>○ Adult child
Email address	______________________________

BMC Health System
THRIVE SCREENER
Addressing Social Determinants of Health

Place Patient Sticker Here

Please fill this out and give to the medical assistant when you are called into the exam room. Your answers will help your care team take better care of your health and connect you with resources. Thank you!

Please check "✓" your answers: I am a ☐ Patient ☐ Parent / Caregiver

**What is your living situation today?**	❍ I have a steady place to live<br>❍ I have a place to live today, but I am worried about losing it in the future<br>❍ I do not have a steady place to live (I am temporarily staying with others, in a hotel, in a shelter, living outside on the street, on a beach, in a car, abandoned building, bus or train station, or in a park)<br>→ ❍ Emergency: I do not have a safe place to stay tonight.
**Within the past 12 months, the food you bought just didn't last and you didn't have money to get more.**	❍ Often true<br>❍ Sometimes true<br>❍ Never true
**Within the past 12 months, you worried whether your food would run out before you got money to buy more.**	❍ Often true<br>❍ Sometimes true<br>❍ Never true
**Is this an emergency, do you need food for tonight?**	❍ Yes ❍ No
**Do you have trouble paying for medicines?**	❍ Yes ❍ No
**Do you have trouble getting transportation to medical appointments?**	❍ Yes ❍ No
**Do you have trouble paying your heating or electricity bill?**	❍ Yes ❍ No
**If yes, are you at risk of having your utilities shut off in the next week?**	❍ Yes ❍ No
**Do you have trouble taking care of a child, family member or friend?**	❍ Yes ❍ No
**Do you have trouble with day-to-day activities such as bathing, preparing meals, shopping, managing finances, etc.?**	❍ Yes ❍ No
**Are you currently unemployed and looking for a job?**	❍ Yes ❍ No
**Are you interested in more education?**	❍ Yes ❍ No

Please check "✓" the resources you want help with:

Housing / Shelter	Food	Paying for Medicine	Transport	Utilities	Childcare	Care for elder or disabled	Daily Support	Job search / training	Education
❍	❍	❍	❍	❍	❍	❍	❍	❍	❍

❍ I do not want to answer these questions

© 2017 Boston Medical Center

Preterm Questionnaire

Study ID

____

Interview Date

____

Location of Interview

____

Interviewer's Name

____
(First and last name)

Protocol #98-38
"Molecular Epidemiologic Study of Low Birth Weight"

I attest that I have fully and appropriately informed this subject of the nature of the above research study and have offered to answer any questions that she may have. This subject has agreed to participate in the study and signed the written informed consent form.

____

1. Who was in the room during the interview?

- ☐ Alone
- ☐ Friends
- ☐ Father of baby
- ☐ Interpreter

Interviewers: Please read the following statement to the subject before you begin interview. I would like to remind you that you may skip any question you do not wish to answer. The following questions are about your general health before and during this pregnancy.

Me gustaría recordarle que puede omitir cualquier pregunta que no desee responder. Las siguientes preguntas son sobre su salud general antes y durante este embarazo

I. General Health Status

Estatus General de Salud

2. Your prepregnancy height

Su altura antes del embarazo
Feet (pie)

____
(Feet)

2. Your prepregnancy height

Su altura antes del embarazo
inches (pulgada)

____
(inches)

2. Your prepregnancy height

Su altura antes del embarazo
cm (Centímetro)

______________________ (cm)

3. Your prepregnant weight

Su peso antes del embarazo
pounds (libras)

______________________ (pounds)

3. Your Prepregnancy weight

Su peso antes del embarazo
kilograms (kilogramo)

______________________ (kilograms)

4. Your total weight gain during pregnancy

Aumento de peso durante su embarazo
Pounds (pies)

______________________ (lbs)

4. Your total weight gain during pregnancy

Aumento de peso durante se embarazo
Kilograms

______________________ (kilograms)

5. Can I ask you about your child's biological father's height, weight and age?

¿Puedo preguntarle sobre la estatura, peso, e edad del padre biológico del bebe?

◯ Yes ◯ No

5a. Your baby's father's height?

Altura del padre del bebe
Feet(Pie)

______________________ (Feet)

5a. Baby's father's height

Altura del padre del bebe
Inches (Pulgada)

______________________ (Inches)

5a. Baby's father's height

Altura del padre del bebe
cm

______________________ (cm)

5b. Baby's father's current weight

Peso del padre del bebe
pounds

______________________ (pounds)

5b. Baby's father's current weight

Peso del padre del bebe
kilograms

______________________ (kilograms)

5c. What is your baby's father's age?

Edad del padre del bebe

____
(years)

II. Information About This Index Pregnancy

6. Did you have a vaginal delivery or C-section of this baby?

¿Tuvo usted un parto vaginal o cesariano?

○ Vaginal ○ C-section

When you came to the hopital, what was your first sign that you were in labor?

¿Cuándo vino al hospital cual que la primera señal del parto?
Contracciones uterinas, Se le Rompió la bolsa, ambos, nunca

○ Uterine CTX
○ ROM without CTX
○ Both CTX and ROM
○ None of the above

If you answered "none of the above," was your labor INDUCED by your doctor or midwife?

¿Su labor de parto fue inducia por un médico durante este embarazo?

○ Yes
○ No

7. Did you get prenatal care from a doctor or midwife during this pregnancy?

¿Tuvo algún cuidado prenatal por parte del médico durante este embarazo?

○ Yes
○ No

8. Where did you get your prenatal care?

¿Dónde tuvo su cuidado prenatal?

☐ BMC-Women's Center
☐ BMC-Doctor's Office Building
☐ BMC-Adolescent Center
☐ Other

If you got your prenatal care somewhere other than BMC, where was that?

____

9. How many weeks pregnant were you when you found out you were pregnant?

¿Con cuantas semanas de gestación descubrió que estaba embarazada?

____
(4-40)

10. How many weeks pregnant were you when you went for your first prenatal visit?

¿Con cuantas semanas tuvo su primera visita prenatal?

____
(0-40)

11. How many prenatal appointments did you miss?

¿Cuántas visitas prenatales faltaste?

____
(appointments number 0-20)

11a. How many prenatal appointments did you have?<br><br>¿Cuántas visitas prenatales tuvo?<br>Menos de cinco, cinco a diez, más de diez.	◯ less than 5 visits<br>◯ 5-10 visits<br>◯ more than 10 visits
11b. How many prenatal ultrasounds did you have?<br><br>¿Cuántos ultrasonidos prenatales tuvo?	______________________________
12. Did you have any flu during this pregnancy?<br><br>¿Tuvo alguna gripe durante este embarazo?	◯ Yes<br>◯ No
a. Did you have the flu in your first trimester?<br><br>En el primer trimestre	◯ Yes<br>◯ No
b. Did you have the flu in your second trimester?<br><br>En el Segundo trimestre	◯ Yes<br>◯ No
c. Did you have the flu in your third trimester?<br><br>En el tercer trimestre	◯ Yes<br>◯ No
13. Did you have any fever during your pregnancy?<br><br>¿Tuvo alguna fiebre durante este embarazo?	◯ Yes<br>◯ No
a. Did you have a fever in your first trimester?<br><br>En el primer trimestre	◯ Yes<br>◯ No
b. Did you have a fever in your second trimester?<br><br>En el Segundo trimestre	◯ Yes<br>◯ No
c. Did you have a fever in your third trimester?<br><br>En el tercer trimestre	◯ Yes<br>◯ No
14. During this pregnancy, did you have any swelling, water retention, or edema?<br><br>¿Durante este embarazo tuvo alguna hinchazón, retención de agua, o edema?	◯ Yes<br>◯ No
a. Did your ankles swell?<br><br>¿Se le hincharon los tobillos/los pies?	◯ Yes<br>◯ No
If your ankles swelled, what week did that start<br><br>¿Cuándo?	______________________________<br>(only if ankles swelled)

b. Did your legs swell?<br><br>¿Se le hincharon las piernas?	◯ Yes<br>◯ No
If your legs swelled, what week of your pregnancy did that begin?<br><br>¿Cuándo?	______________<br>(week)
c. Did your hands swell?<br><br>¿Se le hincharon las manos?	◯ Yes<br>◯ No
If your hands swelled, what week of your pregnancy did that begin<br><br>¿Cuándo?	______________<br>(week)
d. Did your face swell?<br><br>¿Se le hinchó la cara?	◯ Yes<br>◯ No
If your face swelled, at what week in your pregnancy did this begin?<br><br>¿Cuándo?	______________<br>(week)
15. Do you or have you ever had any history of asthma?<br><br>¿Tiene o tuvo alguna historia de asma?<br>No, Solo cuando era Niño/a, Sí	◯ No<br>◯ Only when I was a child, but outgrew it now<br>◯ Yes, I have it now
Did you experience any asthma attacks during your pregnancy?<br><br>¿Tuvo algún ataque de asma durante el embarazo?	◯ Yes<br>◯ No

	Yes	No
First Trimester	◯	◯
Second trimester	◯	◯
Third Trimester	◯	◯

Number of times mother experienced asthma attacks in 1st Trimester of Pregnancy<br><br>Cuantas veces en el primer trimestre	______________<br>(1st trimester)
Number of times mother experienced asthma attacks in 2ndTrimester of Pregnancy<br><br>Cuantas veces en el Segundo trimestre	______________<br>(2nd trimester)
Number of times mother experienced asthma attacks in 3rd Trimester of Pregnancy<br><br>Cuantas veces en el tercer trimestre	______________<br>(3rd trimester)

16. Do you or have you ever had Eczema?

¿Alguna vez tuvo o tiene eczema?
No, solo cuando era Niño/a, Sí

◯ No
◯ Yes, I have it now
◯ Only when I was a child, but outgrew now

17. Do you or have you ever had hay fever or seasonal allergies?

¿Tiene o tuvo alguna fiebre o alergia estacional?
No, solo cuando era Niño/a, Sí

◯ No
◯ Only when I was a child, but outgrew now
◯ Yes, I have it now

18. Do you have any drug allergies?

¿Tiene alergias a algún medicamento?

◯ Yes
◯ No

What is the name of the drug(s)

¿Cuál es el nombre del medicamento?

____

19. Do you or have you ever had food or environmental allergies?

¿Tuvo o tiene alguna alergia alimental o ambiental?
No, Solo cuando era niño/a, Sí

◯ No
◯ Only when I was a child, but out grown now
◯ Yes, I have them now

If you every had an allergy, are you allergic to

	yes	no
Cow's milk, cheese, diary products (Leche de vaca)	◯	◯
Egg (huevos)	◯	◯
Peanut (Maní)	◯	◯
Walnut (Nuez)	◯	◯
Sesame (sesamo)	◯	◯
Shellfish (mariscos)	◯	◯
Fish (pescado)	◯	◯
Soy (soja)	◯	◯
Wheat (Trigo)	◯	◯
Cat (Gatos)	◯	◯
Dog (perros)	◯	◯
Cockroach (cucarachas)	◯	◯
Dust mites (Ácaro)	◯	◯
Mold (Moho)	◯	◯
Other (Otros)	◯	◯

If other allergies, specify allergy name 1

____

If other allergies, specify allergy name 2

____

If other allergies, specify allergy name 3 __________________________________

If other allergies, specify allergy name 4 __________________________________

If other allergies, specify allergy name 5 __________________________________

If other allergies, specify allergy name 6 __________________________________

III. Allergy Related Conditions in Baby's Father

20. Can I ask you some questions about allergies in your baby's father?

¿Puedo hacerle preguntas sobre alergias del padre del bebe?

- ◯ Yes
- ◯ No

21. Does he or has he ever had eczema?

¿Tiene o tuvo alguna vez eczema?
No, Solo cuando era niño/a, Sí, o no sé

- ◯ No
- ◯ Only when he was a child, but has outgrown it
- ◯ Yes, he has it now
- ◯ Don't know

22. Does he or has he ever had any history of asthma?

¿Tiene o tuvo alguna historia de asma?
No, Solo cuando era niño/a, Sí, no sabe, o no sé

- ◯ No
- ◯ Only when he was a child, but has outgrown now
- ◯ Yes, he has it now
- ◯ Don't know

23. Does he or has he ever had hay fever or seasonal allergies?

¿Tiene o tuvo alguna alergia estacional?
No, Solo cuando era niño/a, Sí, no sabe, o no sé

- ◯ No
- ◯ Only when he was a child, but outgrew it
- ◯ Yes, he has them now
- ◯ Don't know

24. Does he or has he ever had any drug allergies?

¿Tiene o tuvo alguna alergia a algún medicamento?

- ◯ Yes
- ◯ No
- ◯ Don't know

If he has a drug allergy, what is the name(s) of the drugs?

¿Cuál es el nombre del medicamento?

____
(Names of drugs)

25. Does he or has he ever had and food or environmental allergies?

¿Tuvo o tiene alguna alergia alimental o ambiental?
No, Solo cuando era niño/a, Sí, no sabe, o no sé

- ◯ No
- ◯ Only when he was a child, but he outgrew it
- ◯ Yes, he has it now
- ◯ Don't know

Yes No

Cow's milk, cheese, dairy products (Leche de vaca y derivados)	◯	◯
Egg (Huevos)	◯	◯
Peanut (Maní)	◯	◯
Walnut (Nuez)	◯	◯
Sesame (Sesamo)	◯	◯
Shellfish (Mariscos)	◯	◯
Fish (Pescado)	◯	◯
Soy (Soja)	◯	◯
Wheat (Trigo)	◯	◯
Cat (Gato)	◯	◯
Dog (Perro)	◯	◯
Coackroaches (Cucarocha)	◯	◯
Dust MItes (Ácaro)	◯	◯
Molds (Moho)	◯	◯
Other (Otros)	◯	◯

Name of product 1 baby's father is allergic to __________________________________

Name of product 2 baby's father is allergic to __________________________________

Name of product 3 baby's father is allergic to __________________________________

Name of product 4 baby's father is allergic to __________________________________

Name of product 5 baby's father is allergic to __________________________________

Name of product 6 baby's father is allergic to __________________________________

26. During this pregnancy, did you have any vaginal bleeding?

¿Durante este embarazo, tuvo algún sangramiento vaginal?

◯ Yes
◯ No

	Yes	No
During the first trimester (Primer Trimestre)	◯	◯
During the second trimester (Segundo Trimestre)	◯	◯

During the third trimester (Tercer Trimestre)	◯	◯
Preceding labor and delivery (Antes de entra en labor y parto)	◯	◯

---

27. Did you have any vaginal or genital tract or urinary tract infections during pregnancy (including yeast infections)?

¿Tuvo alguna infección vaginal, genital, o urinaria durante el embarazo?

◯ Yes
◯ No

In which trimester did your 1st infection occur?

¿En qué trimestre ocurrió su primera infección?

◯ 1 ◯ 2 ◯ 3

First infection type

Clamidia, Gonorrea, Sífilis, Trichonmonas, Estreptococo, Vaginosis, Hongos, Herpes, Papiloma, Algún Otra genital, Desconocida genital, infección urinaria.

◯ Chlamydia
◯ Gonorrhea
◯ Syphilis
◯ Trichomonas
◯ GBS
◯ BV
◯ Yeast
◯ Herpes
◯ HPV
◯ Other GT
◯ Unknown GTI
◯ Urinary Tract

Name of 1st Other Genital Tract Infection

____

What treatment did you take for your infection(S)?
1st Infection

¿Qué tratamiento ha tenido para su infección?

Ninguno, Píldoras, Inyección, Crema, Otra

◯ None
◯ Pill
◯ Shot
◯ Cream
◯ Other

How much of the treatment did you take?
1st infection

¿Cuánto del tratamiento has tenido?
Nada, Algo, Todo

◯ None
◯ Some
◯ All

Specify Other treatment
1st Infection

____

In which trimester did your 2nd infection occur

¿En qué trimestre ocurrió su segundo infección?

◯ 1 ◯ 2 ◯ 3

Second infection type<br><br>Clamidia, Gonorrea, Sífilis, Trichonmonas, Estreptococo, Vaginosis, Hongos, Herpes, Papiloma, Algún Otra genital, Desconocida genital, infección urinaria.	◯ Chlamydia<br>◯ Gonorrhea<br>◯ Syphilis<br>◯ Trichomonas<br>◯ GBS<br>◯ BV<br>◯ Yeast<br>◯ Herpes<br>◯ HPV<br>◯ Other GT<br>◯ Unknown GTI<br>◯ Urinary Tract
Name of 2nd Other Genital Tract Infection	__________________________________
What treatment did you take for your infection(S)?<br>2nd Infection<br><br>¿Qué tratamiento ha tenido para su infección?<br>Ninguno, Píldoras, Inyección, Crema, Otra	◯ None<br>◯ Pill<br>◯ Shot<br>◯ Cream<br>◯ Other
How much of the treatment did you take?<br>2nd infection<br><br>¿Cuánto del tratamiento has tenido?<br>Nada, Algo, Todo	◯ None<br>◯ Some<br>◯ All
Specify Other treatment<br>2nd Infection	__________________________________
In which trimester did your 3rd infection occur<br><br>¿En qué trimestre ocurrió su tercer infección?	◯ 1 ◯ 2 ◯ 3
Third infection type<br><br>Clamidia, Gonorrea, Sífilis, Trichonmonas, Estreptococo, Vaginosis, Hongos, Herpes, Papiloma, Algún Otra genital, Desconocida genital, infección urinaria.	◯ Chlamydia<br>◯ Gonorrhea<br>◯ Syphilis<br>◯ Trichomonas<br>◯ GBS<br>◯ BV<br>◯ Yeast<br>◯ Herpes<br>◯ HPV<br>◯ Other GT<br>◯ Unknown GTI<br>◯ Urinary Tract
Name of 3rd Other Genital Tract Infection	__________________________________
What treatment did you take for your infection(S)?<br>3rd Infection<br><br>¿Qué tratamiento ha tenido para su infección?<br>Ninguno, Píldoras, Inyección, Crema, Otra	◯ None<br>◯ Pill<br>◯ Shot<br>◯ Cream<br>◯ Other

How much of the treatment did you take?<br>3rd infection<br><br>¿Cuánto del tratamiento has tenido?<br>Nada, Algo, Todo	◯ None<br>◯ Some<br>◯ All
Specify Other treatment<br>3rd Infection	__________________________________
In which trimester did your 4th infection occur<br><br>¿En qué trimestre ocurrió su cuarto infección?	◯ 1 ◯ 2 ◯ 3
Fourth infection type<br><br>Clamidia, Gonorrea, Sífilis, Trichonmonas, Estreptococo, Vaginosis, Hongos, Herpes, Papiloma, Algún Otra genital, Desconocida genital, infección urinaria.	◯ Chlamydia<br>◯ Gonorrhea<br>◯ Syphilis<br>◯ Trichomonas<br>◯ GBS<br>◯ BV<br>◯ Yeast<br>◯ Herpes<br>◯ HPV<br>◯ Other GT<br>◯ Unknown GTI<br>◯ Urinary Tract
Name of 4th Other Genital Tract Infection	__________________________________
What treatment did you take for your infection(S)?<br>4th Infection<br><br>¿Qué tratamiento ha tenido para su infección?<br>Ninguno, Píldoras, Inyección, Crema, Otra	◯ None<br>◯ Pill<br>◯ Shot<br>◯ Cream<br>◯ Other
How much of the treatment did you take?<br>4th infection<br><br>¿Cuánto del tratamiento has tenido?<br>Nada, Algo, Todo	◯ None<br>◯ Some<br>◯ All
Specify Other treatment<br>4th Infection	__________________________________
28. Thinking back just before you became pregnant, did you want to become pregnant at that time?<br><br>Pensando en el pasado, antes de embarazarse, quería embarazo	◯ Yes<br>◯ No
28a. IF NO, did you want to become pregnant in the future?<br><br>¿Quiere embarazarse en el futuro?	◯ Yes<br>◯ No

29. How would you characterize the amount of stress in your life in general?<br><br>¿Cómo calificaría la cantidad de estrés en su vida en general antes de su embarazo?<br>No estresante, Más o menos, Muy Estresante	◯ not stressful<br>◯ average<br>◯ very stressful
30. How would you characterize the amount of stress in your life during this pregnancy?<br><br>¿Cómo calificaría la cantidad de estrés en su vida durante este embarazo?<br>No estresante, Más o menos, Muy Estresante	◯ not stressful<br>◯ average<br>◯ very stressful
31. In the last month, how often have you felt that you were unable to control the important things in your life?<br><br>¿En el último mes con qué frecuencia sintió que no podía contrala las cosas importantes de su vida?<br>Nunca, Casi Nunca, A veces, Frecuentemente, Muy frecuentemente	◯ never<br>◯ almost never<br>◯ sometimes<br>◯ fairly often<br>◯ very often
32. In the last month, how often have you felt confident about your ability to handle your personal problems?<br><br>¿En el último mes qué tan frecuentemente se sintió segura de manejar sus problemas personales?<br>Nunca, Casi Nunca, A veces, Frecuentemente, Muy frecuentemente	◯ never<br>◯ almost never<br>◯ sometimes<br>◯ fairly often<br>◯ very often
33. In the last month, how often have you felt things were going your way?<br><br>¿En el último mes, con qué frecuencia sintió que las cosas iban a tu parecer?<br>Nunca, Casi Nunca, A veces, Frecuentemente, Muy frecuentemente	◯ never<br>◯ almost never<br>◯ sometimes<br>◯ fairly often<br>◯ very often
34. In the last month, how often have you felt difficulties were piling up so high that you could not overcome them?<br><br>En el último mes, con qué frecuencia sintió que las dificultades se amontonaban que no podía superarlas?<br>Nunca, Casi Nunca, A veces, Frecuentemente, Muy frecuentemente	◯ Never<br>◯ Almost Never<br>◯ Sometimes<br>◯ Fairly Often<br>◯ Very Often
35. Did you experience any major stressful events, such as loss of family members, divorce, lost job, severe illness/injury of family member etc.?'<br>Mark for each time period.<br><br>¿Experimentó algún evento de gran estrés como la pérdida de un miembro de la familia durante éste embarazo?	◯ Yes<br>◯ No

a. Prepregnancy (within 1 year of conception)<br><br>¿Experimentó algo como eso dentro del año previo a su embarazo?	◯ Yes<br>◯ No
Prepregnancy stress specify	__________________________________
b. First trimester<br><br>Primer	◯ Yes<br>◯ No
first trimester- specify	__________________________________
c. Second trimester<br><br>Segundo	◯ Yes<br>◯ No
Second trimester stress specify	__________________________________
d. Third Trimester<br><br>Tercer	◯ Yes<br>◯ No
Third trimester- specify	__________________________________
36. Did you witness any violence in your pregnmancy?<br><br>¿Fue testigo de algún acto de violencia durante su embarazo?	◯ Yes<br>◯ No
IF YES, specify	__________________________________
36a. If yes to violence, where did the violence occur?<br><br>Donde fue la violencia<br><br>Dentro de su casa, Fuera de su case, Ambos	◯ Inside your home<br>◯ Outside your home<br>◯ Both
37. How would you describe the amount of involvement there was during your pregnancy from the father of your baby?<br><br>¿Cómo describiría el monto de participación que hubo por parte del padre de bebe?<br>No, Un Poco, Bastante, Muy participativo	◯ Not involved ◯ A little involved<br>◯ Mostly involved ◯ Very involved
38. How would you rate the amount of social support you received from the father of your baby?<br><br>¿Cómo describiría el monto de apoyo social que recibió por parte del padre de bebe?<br>No, Un Poco, Bastante, Muy participativo	◯ None ◯ A little ◯ A good amount<br>◯ An excellent amount

39. How would you rate the amount of social support you received during your pregnancy from other family member and your friends?<br><br>¿Cómo describiría el monto de apoyo social que recibió por parte de miembros de la familia y amigos?<br><br>No, Un Poco, Bastante, Muy participativo	◯ None ◯ A little ◯ A good amount<br>◯ An excellent amount

IV. Reproductive History

Ahora me gustaría preguntar sobre su historia reproductiva

40. How old were you when you had your first period?<br><br>¿Qué edad tenía cuando tuvo su primer periodo?	______________________
a. Does your period come each month?<br><br>¿Su periodo viene cada mes?	◯ Yes ◯ No
b. Does your menstrual period come around the same time each month (+/- 7 days from your last period?)<br><br>¿Su period viene alredor de la misma fecha cada mes?	◯ Yes<br>◯ No
c. What is your average cycle length in days, that is, how many days are there from one period to the next?<br><br>¿Cuál es el promedio de su ciclo menstrual-cuantos días entre el final de su periodo y el comienzo del próximo?	______________________
d. How long does each period's bleeding last in days?<br><br>¿Cuánto dura el sangrado durante su periodo?	______________________
41. Do you have pelvic or abdominal pain during your menstrual period?<br><br>¿Tiene dolor pélvico o abdominal durante su periodo?<br>No, Ocasionalmente, Casi siempre	◯ No<br>◯ Occasionally<br>◯ Almost all of the time
42a. If you answered "occasionally" or "almost all of the time," how do you rate your menstrual pain?<br><br>¿Cómo calificaría el dolor?<br>Bajo, Moderado, Severo	◯ Mild<br>◯ Moderate<br>◯ Severe (could not go to work or school)

42. Prior to this pregnancy, what kind of birth control were you using? (check all that apply)

¿Antes de este embarazo, qué tipo de metedos anticonceptivos usaba?
Ninguno, Abstinencia durante días fértiles, Pastillas, Capuchón cervical, Condones, Cremas, Inyecciones de hormonas, IUD, Parche, Retirada, Otra

☐ None ☐ Abstinence during fertile day (i.e. natural family planning) ☐ Birth Control Pills
☐ Cervical Cap ☐ Condoms ☐ Spermicide Creams ☐ Hormone Shots ☐ IUD ☐ Birth Control Patch
☐ Withdrawal ☐ Other

If you chose "other" please specify the name.

____

---

43. How many times have you been pregnant? (include miscarriages, abortions, or stillbirths)

¿Cuántas veces ha estado embarazado? (incluyendo abortos, abortos espontáneos, muertes fetales, y este embarazo

○ 0 ○ 1 ○ 2 ○ 3 ○ 4 ○ 5 ○ 6 ○ 7 ○ 8 ○ 9 ○ 10 ○ 11 ○ 12
○ 13

---

a. On what date did your first pregnancy end?

¿En qué fecha acabó el embarazo?

____

---

b. A full term pregnancy generally lasts 40 weeks, how many weeks did your first pregnancy last?

¿Cuántas semanas duró el embarazo?

____

---

c. How did the pregnancy end?

¿Cómo acabó el embarazo?
Nacimiento vivo, Muerte fetal, Aborto espontaneo, Aborto, Embarazo Ectopico, Moles

○ Live birth ○ Still birth ○ Miscarriage ○ Abortion ○ Ectopic pregnancy ○ Moles

---

d. Did you have any of the following pregnancy complications in your first pregnancy? (check all that apply)

¿Complicaciones en el embarazo?
Ninguna, Moderado Preeclampsia, Severa Preeclampsia, eclampsia, desprendimiento prematuro de la placenta, placenta previa, Incompetencia cervical, Diabetes gestacional, Infección intrauterina, otras.

☐ None ☐ Mild Preeclampsia ☐ Severe Preeclampsia ☐ Eclampsia ☐ Abruptio Placentae
☐ Placenta Previa ☐ Incompetent Cervix ☐ Gestational Diabetes ☐ Intrauterine Infection
☐ Others

If others, specify

____

e. If this was a live birth, was your first baby a boy or girl?

¿Sexo del bebe?

◯ Male ◯ Female

f. Did you deliver your first baby vaginally or by a C-Section?

¿Tipo de parto?

◯ Vaginal ◯ C-Section

g. What was the birthweight of your first child in pounds ?

¿Peso del bebe?
Libras

____
(pounds)

How many ounces?

Onzas

____
(ounces)

Did your first child have any birth defects? (specify)

Defectos de nacimiento

____

a. On what date did your second pregnancy end?

¿En qué fecha acabó el embarazo? _______________________________

b. How many weeks did your second pregnancy last?

¿Cuántas semanas duró el embarazo? _______________________________

c. How did your second pregnancy end?

¿Cómo acabó el embarazo?
Nacimiento vivo, Muerte fetal, Aborto espontaneo, Aborto, Embarazo Ectopico, Moles

◯ Live birth ◯ Still birth ◯ Miscarriage ◯ Abortion ◯ Ectopic pregnancy ◯ Moles

d. Did you have any complications in your second pregnancy?

¿Complicaciones en el embarazo?
Ninguna, Moderado Preeclampsia, Severa Preeclampsia, eclampsia, desprendimiento prematuro de la placenta, placenta previa, Incompetencia cervical, Diabetes gestacional, Infección intrauterina, otras.

☐ None ☐ Mild Preeclampsia ☐ Severe Preclampsia ☐ Eclampsia ☐ Abruptio Placentae
☐ Placenta Previa ☐ Incompetent Cervix ☐ Gestational Diabetes ☐ Intrauterine Infection
☐ Others

If "others," please specify

____

e. (If live birth) What was the sex of your second baby?

¿Sexo del bebe?

◯ Male ◯ Female

f. Did you deliver your second baby vaginally or by C-section?

¿Tipo de parto?

◯ Vaginal ◯ C-section

g. How much did your second baby weigh in pounds?

¿Peso del bebe?

____
(pounds)

How many ounces did your second baby weigh?

____
(ounces)

h. Did your second baby have any birth defects? If yes, specify

Defectos de nacimiento

____

a. On what date did your third pregnancy end?

¿En qué fecha acabó el embarazo?

____

b. A full term pregnancy usually lasts about 40 weeks, how many weeks did your third pregnancy last?

¿Cuántas semanas duró el embarazo?

____

c. How did your third pregnancy end?

¿Cómo acabó el embarazo?
Nacimiento vivo, Muerte fetal, Aborto espontaneo, Aborto, Embarazo Ectopico, Moles

◯ Live birth ◯ Still birth ◯ Miscarriage ◯ Abortion ◯ Ectopic pregnancy ◯ Moles

d. Did your third pregnancy have any of the following complications?

¿Complicaciones en el embarazo?
Ninguna, Moderado Preeclampsia, Severa Preeclampsia, eclampsia, desprendimiento prematuro de la placenta, placenta previa, Incompetencia cervical, Diabetes gestacional, Infección intrauterina, otras.

☐ None ☐ Mild Preeclampsia ☐ Severe Preeclampsia ☐ Eclampsia ☐ Abruptio Placentae
☐ Placenta Previa ☐ Incompetent Cervix ☐ Gestational Diabetes ☐ Intrauterine Infection
☐ Others

If Others, specify

____

e. (If live birth) What was the sex of your third baby?

¿Sexo del bebe?

◯ Male ◯ Female

f. Did you deliver your third baby vaginally or by C-section?

¿Tipo de parto?

◯ Vaginal ◯ C-section

g. How much did your third baby weigh in pounds?

¿Peso del bebe?

____
(pounds)

How much did your third baby weigh in ounces?

____
(ounces)

h. Did your 3rd baby have any Birth defects, specify

Defectos de nacimiento

____

a. On what date did your fourth pregnancy end?

¿En qué fecha acabó el embarazo?

____

b. A full-term pregnancy usually lasts about 40 weeks, how many weeks did your fourth pregnancy last?

¿Cuántas semanas duró el embarazo?

____

c. How did your fourth pregnancy end?

¿Cómo acabó el embarazo?
Nacimiento vivo, Muerte fetal, Aborto espontaneo, Aborto, Embarazo Ectopico, Moles

○ Live birth ○ Still birth ○ Miscarriage ○ Abortion ○ Ectopic pregnancy ○ Moles

d. Did your fourth pregnancy have any of the following complications?

¿Complicaciones en el embarazo?
Ninguna, Moderado Preeclampsia, Severa Preeclampsia, eclampsia, desprendimiento prematuro de la placenta, placenta previa, Incompetencia cervical, Diabetes gestacional, Infección intrauterina, otras.

☐ None ☐ Mild Preeclampsia ☐ Severe Preeclampsia ☐ Eclampsia ☐ Abruptio Placentae
☐ Placenta Previa ☐ Incompetent Cervix ☐ Gestational Diabetes ☐ Intrauterine Infection
☐ Others

If others, specify:

____

e. (If live birth) What was the sex of your fourth baby?

¿Sexo del bebe?

○ Male ○ Female

f. Did you deliver your fourth baby vaginally or by C-section?

¿Tipo de parto?

○ Vaginal ○ C-section

g. How much did your fourth baby weigh in pounds?

¿Peso del bebe?

____
(pounds)

How much did your fourth baby weigh in ounces?

____

h. Did your fourth baby have any birth defects? If yes, specify

Defectos de nacimiento

____

On what date did your fifth pregnancy end?

____

A full-term pregnancy usually lasts about 40 weeks, how many weeks did your fifth pregnancy last?

____

How did your fifth pregnancy end?

◯ Live birth ◯ Still birth ◯ Miscarriage ◯ Abortion ◯ Ectopic pregnancy ◯ Moles

Did your fifth pregnancy have any of the following complications?

☐ None ☐ Mild Preeclampsia ☐ Severe Preeclampsia ☐ Eclampsia ☐ Abruptio Placentae
☐ Placenta Previa ☐ Incompetent Cervix ☐ Gestational Diabetes ☐ Intrauterine Infection
☐ Others

If others, specify:

____

(If live birth) What was the sex of your fifth baby?

◯ Male ◯ Female

Did you deliver your fifth baby vaginally or by C-section?

◯ Vaginal ◯ C-section

How much did your fifth baby weigh in pounds?

____
(pounds)

How much did your fifth baby weigh in ounces?

____
(ounces)

Did your fifth child have any birth defects? If yes, specify

____

On what date did your 6th pregnancy end?

____

How many weeks did your 6th pregnancy last?

____

How did the 6th pregnancy end?	◯ Live Birth<br>◯ Still Birth<br>◯ Miscarriage<br>◯ Abortion<br>◯ Ectopic Pregnancy<br>◯ Moles
Complications during 6th pregnancy	☐ None<br>☐ Mild Preeclampsia<br>☐ Severe Preeclampsia<br>☐ Eclampsia<br>☐ Abruptio Placentae<br>☐ Placenta Previa<br>☐ Incompetent Cervix<br>☐ Gestational Diabetes<br>☐ Intrauterine Infection<br>☐ Others
If others, specify	______________________
(If live Birth) What was the sex of your 6th baby?	◯ Male<br>◯ Female
Did you deliver your 6th baby vaginally or by C-Section?	◯ Vaginal<br>◯ C-section
How much did your 6th baby weigh? pounds	______________________<br>(pounds)
How much did your 6th baby weigh? Ounces	______________________<br>(ounces)
Did your 6th baby have any birth defects? If yes, specify:	______________________
On what date did your 7th pregnancy end?	______________________
How many weeks did your 7th pregnancy last?	______________________
How did the 7th pregnancy end?	◯ Live Birth<br>◯ Still Birth<br>◯ Miscarriage<br>◯ Abortion<br>◯ Ectopic Pregnancy<br>◯ Moles

Complications during 7th pregnancy	☐ None<br>☐ Mild Preeclampsia<br>☐ Severe Preeclampsia<br>☐ Eclampsia<br>☐ Abruptio Placentae<br>☐ Placenta Previa<br>☐ Incompetent Cervix<br>☐ Gestational Diabetes<br>☐ Intrauterine Infection<br>☐ Others
If others, specify	______________________
(If live Birth) What was the sex of your 7th baby?	◯ Male<br>◯ Female
Did you deliver your 7th baby vaginally or by C-Section?	◯ Vaginal<br>◯ C-section
How much did your 7th baby weigh? pounds	______________________<br>(pounds)
How much did your 7th baby weigh? Ounces	______________________<br>(ounces)
Did your 7th baby have any birth defects? If yes, specify:	______________________
On what date did your 8th pregnancy end?	______________________
How many weeks did your 8th pregnancy last?	______________________
How did the 8th pregnancy end?	◯ Live Birth<br>◯ Still Birth<br>◯ Miscarriage<br>◯ Abortion<br>◯ Ectopic Pregnancy<br>◯ Moles
Complications during 8th pregnancy	☐ None<br>☐ Mild Preeclampsia<br>☐ Severe Preeclampsia<br>☐ Eclampsia<br>☐ Abruptio Placentae<br>☐ Placenta Previa<br>☐ Incompetent Cervix<br>☐ Gestational Diabetes<br>☐ Intrauterine Infection<br>☐ Others
If others, specify	______________________

(If live Birth) What was the sex of your 8th baby?	◯ Male<br>◯ Female
Did you deliver your 8th baby vaginally or by C-Section?	◯ Vaginal<br>◯ C-section
How much did your 8th baby weigh?<br>pounds	____________________<br>(pounds)
How much did your 8th baby weigh?<br>Ounces	____________________<br>(ounces)
Did your 8th baby have any birth defects? If yes, specify:	____________________
On what date did your 9th pregnancy end?	____________________
How many weeks did your 9th pregnancy last?	____________________
How did the 9th pregnancy end?	◯ Live Birth<br>◯ Still Birth<br>◯ Miscarriage<br>◯ Abortion<br>◯ Ectopic Pregnancy<br>◯ Moles
Complications during 9th pregnancy	☐ None<br>☐ Mild Preeclampsia<br>☐ Severe Preeclampsia<br>☐ Eclampsia<br>☐ Abruptio Placentae<br>☐ Placenta Previa<br>☐ Incompetent Cervix<br>☐ Gestational Diabetes<br>☐ Intrauterine Infection<br>☐ Others
If others, specify	____________________
(If live Birth) What was the sex of your 9th baby?	◯ Male<br>◯ Female
Did you deliver your 9th baby vaginally or by C-Section?	◯ Vaginal<br>◯ C-section
How much did your 9th baby weigh?<br>pounds	____________________<br>(pounds)
How much did your 9th baby weigh?<br>Ounces	____________________<br>(ounces)

Did your 9th baby have any birth defects? If yes, specify:	______________________________
On what date did your 10th pregnancy end?	______________________________
How many weeks did your 10th pregnancy last?	______________________________
How did the 10th pregnancy end?	◯ Live Birth<br>◯ Still Birth<br>◯ Miscarriage<br>◯ Abortion<br>◯ Ectopic Pregnancy<br>◯ Moles
Complications during 10th pregnancy	☐ None<br>☐ Mild Preeclampsia<br>☐ Severe Preeclampsia<br>☐ Eclampsia<br>☐ Abruptio Placentae<br>☐ Placenta Previa<br>☐ Incompetent Cervix<br>☐ Gestational Diabetes<br>☐ Intrauterine Infection<br>☐ Others
If others, specify	______________________________
(If live Birth) What was the sex of your 10th baby?	◯ Male<br>◯ Female
Did you deliver your 10th baby vaginally or by C-Section?	◯ Vaginal<br>◯ C-section
How much did your 10th baby weigh? pounds	______________________________<br>(pounds)
How much did your 10th baby weigh? Ounces	______________________________<br>(ounces)
Did your 10th baby have any birth defects? If yes, specify:	______________________________
On what date did your 11th pregnancy end?	______________________________
How many weeks did your 11th pregnancy last?	______________________________

How did the 11th pregnancy end?	◯ Live Birth<br>◯ Still Birth<br>◯ Miscarriage<br>◯ Abortion<br>◯ Ectopic Pregnancy<br>◯ Moles
Complications during 11th pregnancy	☐ None<br>☐ Mild Preeclampsia<br>☐ Severe Preeclampsia<br>☐ Eclampsia<br>☐ Abruptio Placentae<br>☐ Placenta Previa<br>☐ Incompetent Cervix<br>☐ Gestational Diabetes<br>☐ Intrauterine Infection<br>☐ Others
If others, specify	__________________________
(If live Birth) What was the sex of your 11th baby?	◯ Male<br>◯ Female
Did you deliver your 11th baby vaginally or by C-Section?	◯ Vaginal<br>◯ C-section
How much did your 11th baby weigh? pounds	__________________________<br>(pounds)
How much did your 11th baby weigh? Ounces	__________________________<br>(ounces)
Did your 11th baby have any birth defects? If yes, specify:	__________________________
On what date did your 12th pregnancy end?	__________________________
How many weeks did your 12th pregnancy last?	__________________________
How did the 12th pregnancy end?	◯ Live Birth<br>◯ Still Birth<br>◯ Miscarriage<br>◯ Abortion<br>◯ Ectopic Pregnancy<br>◯ Moles

Complications during 12th pregnancy	☐ None<br>☐ Mild Preeclampsia<br>☐ Severe Preeclampsia<br>☐ Eclampsia<br>☐ Abruptio Placentae<br>☐ Placenta Previa<br>☐ Incompetent Cervix<br>☐ Gestational Diabetes<br>☐ Intrauterine Infection<br>☐ Others
If others, specify	__________________________________
(If live Birth) What was the sex of your 12th baby?	◯ Male<br>◯ Female
Did you deliver your 12th baby vaginally or by C-Section?	◯ Vaginal<br>◯ C-section
How much did your 12th baby weigh? pounds	__________________________________<br>(pounds)
How much did your 12th baby weigh? Ounces	__________________________________<br>(ounces)
Did your 12th baby have any birth defects? If yes, specify:	__________________________________

Did you have sexual intercourse during this pregnancy in the 1st trimester (0-12 weeks pregnant)

¿Tuvo relaciones sexuales durante este embarazo? ¿Primer Trimestre?

◯ Yes ◯ No

If yes, did you used condoms?

¿Usó condón?

◯ Yes ◯ No

Approximately how many times per month did you have sexual intercourse in your first trimester?

¿Con qué frecuencia tuvo relaciones sexuales por mes?

____

Did you have sexual intercourse in your second trimester? (weeks 13-27)

¿Tuvo relaciones sexuales durante este embarazo? ¿Segundo trimestre?

◯ Yes ◯ No

If yes, did you use condoms?

¿Usó condón?

◯ Yes ◯ No

Approximately how many times per month did you have sexual intercourse in your second trimester?

¿Con qué frecuencia tuvo relaciones sexuales por mes?

____

Did you have sexual intercourse in your third trimester? (weeks 28 and over)

¿Tuvo relaciones sexuales durante este embarazo? ¿Tercer trimestre?

◯ Yes ◯ No

If yes, did you use condoms?

¿Usó condón?

◯ Yes ◯ No

Approximately how many times per month did you have sexual intercourse in your third trimester?

¿Con qué frecuencia tuvo relaciones sexuales por mes?

____

Did you have more than one sexual partner during this pregnancy?

¿Tuvo más de una pareja sexual durante este embarazo?

◯ Yes ◯ No

Gravidity (total # of pregnancies including index case)

____
(FIELD DATA ENTRY ONLY)

Parity (#of live births NOT INCLUDING index case)

____

Number of priot preterm births (< 37 weeks)

____

Number of prior LBW births (< 2500g)

____

Number of prior stillbirths

____

Number of spontaneous abortions

____

Number of induced abortions

____

V. Daily Physical Activity Before and During the Index Pregnancy

47a. Were you working 3 months prior to your pregnancy?<br><br>Fue trabajando en los 3 meses del pre embarazo	◯ Yes<br>◯ No
47b. Industry	__________________________________<br>(3 mo. prior)
47c. Job Title<br><br>Título del trabajo	__________________________________<br>(3 mo. prior)
47d. Duties	__________________________________<br>(3 mo. prior)
47e. Work schedule<br><br>Su horario de trabajo<br>Diario<br>Tarde<br>Noches	◯ Not working<br>◯ Regular day shift<br>◯ Regular evening shift<br>◯ Regular night shift<br>◯ Irregular shift
47f. How many hours did you work each week?<br><br>¿Cuántas horas trabajaba a la semana?	__________________________________<br>(hours/wk)
47g. How long did it take you to get to work? (one way in minutes?<br><br>¿Cuánto tiempo necesitaba para llegar a su trabajo?	__________________________________<br>(minutes)
47h. How physically demanding is your job?<br><br>¿Qué tan físicamente difícil es su trabajo?	◯ Slight<br>◯ Moderate<br>◯ Very Much

47i. How much job-related mental stress did you experience?<br><br>¿Qué cantidad de estrés mental relacionado a su trabajo experimento?	◯ Slight<br>◯ Moderate<br>◯ Very Much
Were you working in your 1st trimester?	◯ Yes<br>◯ No
Industry	______________________<br>(1st trimester)
Job Title	______________________<br>(1st trimester)
Duties	______________________<br>(1st trimester)
Work schedule	◯ Not working<br>◯ Regular day shift<br>◯ Regular evening shift<br>◯ Regular night shift<br>◯ Irregular shift
How many hours did you work each week?	______________________<br>(hours/wk)
How long did it take you to get to work? (one way in minutes	______________________<br>(minutes)
How physically demanding is your job?	◯ Slight<br>◯ Moderate<br>◯ Very Much
How much job-related mental stress did you experience?	◯ Slight<br>◯ Moderate<br>◯ Very Much
Were you working in the 2nd trimester?	◯ Yes<br>◯ No
Industry	______________________<br>(2nd trimester)
Job Title	______________________<br>(2nd trimester)
Duties	______________________<br>(2nd trimester)

Work schedule	◯ Not working<br>◯ Regular day shift<br>◯ Regular evening shift<br>◯ Regular night shift<br>◯ Irregular shift
How many hours did you work each week?	______________________<br>(hours/wk)
How long did it take you to get to work? (one way in minutes	______________________<br>(minutes)
How physically demanding is your job?	◯ Slight<br>◯ Moderate<br>◯ Very Much
How much job-related mental stress did you experience?	◯ Slight<br>◯ Moderate<br>◯ Very Much
Were you working in the 3rd trimester	◯ Yes<br>◯ No
Industry	______________________<br>(3rd trimester)
Job Title	______________________<br>(3rd trimester)
Duties	______________________<br>(3rd trimester)
Work schedule	◯ Not working<br>◯ Regular day shift<br>◯ Regular evening shift<br>◯ Regular night shift<br>◯ Irregular shift
How many hours did you work each week?	______________________<br>(hours/wk)
How long did it take you to get to work? (one way in minutes	______________________<br>(minutes)
How physically demanding is your job?	◯ Slight<br>◯ Moderate<br>◯ Very Much

How much job-related mental stress did you experience?

◯ Slight
◯ Moderate
◯ Very Much

VI. Home Environment

Ambiente del Hogar

48. Did you Live outside the U.S. during this (index) pregnancy

¿Vivió fuera de los Estados Unidos durante este embarazo?

◯ Yes
◯ No

If Yes, what country(s) did you live in?

¿En qué país vivió?

____

Country Code

____

For how long did you live outside the U.S.?

¿Por cuánto tiempo vivió fuera de los Estado Unidos?

____
(weeks)

(Calculate, do not ask) Most of pregnancy was:

◯ Inside U.S.
◯ Outside U.S.

49. a. If Lived Most of Pregnancy in the U.S.
What is the zip code of the place you lived the longest?

¿Cuál es el código postal del lugar donde vivió durante la mayoría del embarazo?

____

b. If lived most of pregnancy in U.S.
If Don't know zip code: What town was it?

¿o el nombre de la ciudad?

____

50. How Long have you lived in your current home?

¿Cuánto ha vivido en su casa actual?

____
(years)

How long have you lived in your current home?

____
(months)

How long have you lived in your current home?

____
(day)

51. Did you live in a shelter for any part of this pregnancy?

¿Vivió en algún refugio durante cualquier parte de este embarazo?

◯ Yes
◯ No

How long?

¿Cuánto tiempo?

____
(months)

How long?

¿Cuánto tiempo?

____
(week)

How long?

¿Cuánto tiempo?

____
(day)

52. All questions below refer to the home mother lived the longest

# of bedrooms

¿Cuántas habitaciones tiene el hogar?

____
(longest home during preg)

# of bathrooms

¿Cuántos baños?

____
(longest home during preg)

# of people who permanently live in your home

¿Cuántas personas residen permanente amenté en su casa?

____
(longest home during preg)

What type of fuel do you use for heating your home?

¿Qué usa Ud. para calentar la casa?
Aceite
Electricidad
Gas

◯ Oil
◯ Electricity
◯ Gas
◯ Other
(longest home during preg)

Specify other type of fuel

____
(longest home during preg)

What type of stove do you use for cooking?

¿Y para cocinar?
Gas
Electricidad

◯ Gas
◯ Electric
◯ Other

Specify other stove

____
(longest home during preg)

Do you have any wall to wall carpet?

¿Hay alfombra de pared a pared en alguna parte de la casa?

◯ Yes
◯ No

Specify Location<br><br>Sala<br>Sala de estar<br>Comendar<br>Cocina<br>Habitaciones<br>Sótano<br>Baño	☐ Living Room<br>☐ Family Room<br>☐ Dinning Room<br>☐ Kitchen<br>☐ Bedroom<br>☐ Basement<br>☐ Bathroom<br>☐ Hallways<br>☐ Other<br>(longest home during preg)
If other, specify	___________________________________<br>(longest home during preg)
Do/did you have any pets at the place you lived the longest?<br><br>¿Tiene Ud. mascotas o animales en la casa?	◯ Yes<br>◯ No

	Yes	No
Cat / Gato	◯	◯
Dog / Pero	◯	◯
Fish / Pez	◯	◯
Bird / Pájaro	◯	◯
Reptile / Reptiles	◯	◯
Rabbit / Conejo	◯	◯
Guinea Pig / Conejillo de indias	◯	◯
Other / otro	◯	◯

If other pet, specify:	___________________________________
Does the place you lived in the longest have any cockroaches?<br><br>¿Ha visto cucarachas en la casa?	◯ Yes<br>◯ No
Does the house you lived in the longest have any mice/rats?<br><br>¿Ha visto ratones o ratas en las casa?	◯ Yes<br>◯ No
Does the house you lived in the longest have any visible mold, mildew, water damage, leakage, or seepage?<br><br>¿Hay moho o daños por agua en la casa?	◯ Yes<br>◯ No
Was the place you lived in the longest a farming environment?<br><br>¿Ud. no vive en una granja, verdad?	◯ Yes<br>◯ No

VII. CIGARETTE SMOKING

Uso del Tabaco/ cigarrillos

53. Have you ever smoked cigarettes, cigars, or pipe tobacco, or used chewing tobacco or snuff? (Even once)<br><br>¿Ha fumado cigarrillos, cigarros, pipas, o usado tabaco masticable alguna vez en su vida?	◯ Yes<br>◯ No
54. Have you ever smoked or used tobacco regularly for at least a month?<br><br>¿Ha fumado regularmente por lo menos un mes?	◯ Yes<br>◯ No
55. How old were you when you began to smoke or use tobacco regularly?<br><br>¿Cuál era su edad cuando comienzo a usar tabaco regularmente?	______________________
56. Altogether, over your entire life, how long have you smoked or used tobacco regularly?<br><br>¿Durante toda su vida, cuánto tiempo ha fumando regularmente?	______________________<br>(years)
56. Altogether, over your entire life, how long have you smoked or used tobacco regularly?<br><br>¿Durante toda su vida, cuánto tiempo ha fumando regularmente?	______________________<br>(months)
57a. When you used tobacco regularly, did you use cigarettes?<br><br>¿Cuándo usó el tabaco regularmente, usó cigarillos?	◯ Yes<br>◯ No
Did you use cigarettes or E-Cigarettes?<br>Check all that apply:	☐ Cigarette<br>☐ E-Cigarette
57a. If yes: When you smoked cigarettes, on average how many cigarettes would you smoke per day?	______________________<br>(cigarettes)
57b. When you used tobacco regularly, did you use cigars?<br><br>¿Cuándo usó el tabaco regularmente, usó cigarros?	◯ Yes<br>◯ No
If yes; When you smoked cigars, on average how many cigars would you smoke per day?	______________________<br>(cigars)

57c. When you used tobacco regularly, did you use pipes?<br><br>¿Cuándo usó el tabaco regularmente, usó pipas?	◯ Yes<br>◯ No
If yes: When you smoked pipes, on average how many pipes would you smoke per day?	__________________________________<br>(pipes)
57d. When you used tobacco regularly, did you use chewing tobacco?<br><br>¿Cuándo usó el tabaco regularmente, usó usado tabaco masticable?	◯ Yes<br>◯ No
If yes: When you chewed tobacco, on average how much would you use per day?	__________________________________<br>(chaws)
57e. When you used tobacco regularly, did you use snuff?<br><br>¿Cuándo usó el tabaco regularmente, usó usado en polvo?	◯ Yes<br>◯ No
If yes: When you used snuff, on average how much would you use per day?	__________________________________<br>(dips)
58. I would now like to ask you some questions about your (smoking/tobacco use) during the time in your like when you were using tobacco the most heavily. How old were you at the that time? (IF OVER A PERIOD OF TIME RECORD AGE AT WHICH BEGAN USING HEAVILY)<br><br>Me gustaría hacerle unas preguntas sobre el tiempo en el que hizo uso del tabaco con más frecuencia. ¿Qué edad tenía en este periodo?	__________________________________<br>(years)
59a. During the time when you were(smoking/using tobacco) most heavily, on average, how many cigarettes would you have per day?<br><br>¿Durante el tiempo en el que usó el tabaco más pesadamente en promedio cuántos cigarrillos al día?	__________________________________<br>(cigarettes)
59b. During the time when you were(smoking/using tobacco) most heavily, on average, how many cigars would you have per day?<br><br>¿Durante el tiempo en el que usó el tabaco más pesadamente en promedio cuántos cigarros al día?	__________________________________<br>(cigars)
59c. During the time when you were(smoking/using tobacco) most heavily, on average, how many pipes would you have per day?<br><br>¿Durante el tiempo en el que usó el tabaco más pesadamente en promedio cuántos pipas al día?	__________________________________<br>(pipes)

59d. During the time when you were(smoking/using tobacco) most heavily, on average, how many dips would you have per day?

¿Durante el tiempo en el que usó el tabaco más pesadamente en promedio cuántos Tabaco masticable al día?

____
(dips)

59e. During the time when you were(smoking/using tobacco) most heavily, on average, how many chaws would you have per day?

¿Durante el tiempo en el que usó el tabaco más pesadamente en promedio cuántos en polvo al día?

____
(chaws)

60. During this time when you (smoked/used tobacco) most heavily, how soon after you awoke did you (smoke/use tobacco)

Igualmente durante este tiempo. ¿Con cuánto tiempo después de se despertaba hacia uso del tabaco?

____
(hours)

60b. During this time when you (smoked/used tobacco) most heavily, how soon after you awoke did you (smoke/use tobacco)

Igualmente durante este tiempo. ¿Con cuánto tiempo después de se despertaba hacia uso del tabaco?

____
(minutes)

61. During this when you (smoked/used tobacco) most heavily, how would you check to make sure that you had (cigarettes/cigars/tobacco) around to (smoke/use)?

¿Durante este tiempo con que frecuencia checaba que hubieren alredor para usar?
Frecuentemente
A veces
Raramente
Nunca

- ◯ Often
- ◯ Sometimes
- ◯ Rarely
- ◯ Never

62. During this time when you (smoked/used tobacco) most heavily,, if you didn't (smoke/use tobacco) for a period of time, how strong would your cravings get for another (cigarette/cigar/pip/dip/chaw)?

¿Durante este tiempo si no fumaba por un periodo de tiempo que tan grande era su necesidad de obtener otro?
Muy Fuerte
Fuerte
Moderada
Muy Baja

- ◯ Very Strong
- ◯ Strong
- ◯ Moderate
- ◯ Hardly any

63. During this time when you (smoked/used tobacco) most heavily, how difficult was it for you to not (smoke/use) it in places where is was forbidden? Would you say...<br><br>¿Durante este tiempo qué tan difícil era para usted no fumar en lugares donde era prohibido?<br>Muy difícil<br>Difícil<br>Poco difícil<br>Nada difícil	◯ Very difficult<br>◯ Somewhat difficult<br>◯ A little difficult<br>◯ Not difficult at all
64. During this time when you (smoked/used tobacco) most heavily, would you (smoke/use tobacco) when you were so ill that you were in bed most of the day?<br><br>¿Durante este tiempo fumaria cuando estuviese muy enferma?	◯ Yes<br>◯ No
65. During this time when you (smoked/used tobacco) most heavily, would you (smoke/use tobacco) more during the morning than during the rest of the day?<br><br>¿Durante este tiempo fumaria más durante la mañana que durante el resto del día?	◯ Yes<br>◯ No
66. During this time when you (smoked/used tobacco) most heavily, which (cigarette/cigar/pipe/dip/chaw) of the day would be the most satisfying? Was it the first?<br><br>¿Durante este tiempo que cigarrillo era el más satisfactorio? ¿Era el primero?	◯ First<br>◯ Other<br>◯ Not sure
67. IF SMOKED IN HEAVIEST USE PERIOD: During that time when you smoked most heavily, how often did you inhale? would you say:<br><br>¿Con que frecuencia inhalaba?	◯ Always<br>◯ Sometimes<br>◯ Never
67a. IF ALWAYS OR SOMETIMES: How often did you inhale deep into your lungs: would you say:<br><br>¿Con que frecuencia inhalaba profundamente has sus pulmones?	◯ Always<br>◯ Sometimes<br>◯ Never
68. Have you ever seriously attempted to stop (smoking/using tobacco)?<br><br>¿Ha intentado parar de fumar seriamente?	◯ Yes<br>◯ No
69. How many times in your like have you seriously tried to stop (smoking/using tobacco)?<br><br>¿Cuántas veces?	____________________<br>(times)
70. How depressed did you get when you tried to quit (smoking/using tobacco)?<br><br>¿Qué tan deprimida se sentía cuando intentaba para?	◯ Very<br>◯ Somewhat<br>◯ A little<br>◯ Hardly at all

72. How nervous, jittery, or irritable did you get when you tried to quit (smoking/using tobacco)?<br><br>¿Qué tan nerviosa, irritable, o ansiosa se ponía cuando intentaba parar?	◯ Very<br>◯ Somewhat<br>◯ A little<br>◯ Hardly at all
71. Have you ever gone to a professional to help you stop (smoking/using tobacco)?<br><br>¿Ha buscado ayuda profesional para ayudarla a parar?	◯ Yes<br>◯ No
IF YES: Whom did you see? Check all that apply<br><br>¿Quién?<br>Doctor<br>Profesional de la salud mental<br>Clínica<br>Hipnotista	☐ Regular doctor<br>☐ Mental health professional<br>☐ Stop smoking clinic/workshop<br>☐ Hypnotist<br>☐ Other
If other, specify	____________________________
73. Have you ever used nicotine gum or patches to help you stop (smoking/using tobacco)?<br><br>¿Ha usado alguna vez parches o chicle de nicotina para ayudarla a parar?	◯ Yes<br>◯ No
74. a. In the first six months before you found out you were pregnant, did you (smoke/use tobacco)?<br><br>En los seis meses antes de embarazarse, ¿hizo uso del tabaco?	◯ Yes<br>◯ No
If yes, on average, how many cigarettes did you use per day?	____________________________<br>(cigarettes)
What type of cigarette?	☐ Cigarette<br>☐ E-Cigarette
If yes, on average, how many cigars did you use per day?	____________________________<br>(cigars)
If yes, on average, how many pipes did you use per day?	____________________________<br>(pipes)
If yes, on average, how many dips did you use per day?	____________________________<br>(dips)
If yes, on average, how many chaws did you use per day?	____________________________<br>(chaws)

74b. In the first three months of your pregnancy, did you (smoke/use tobacco)?

En los primeros tres meses del embarazo, ¿hizo uso del tabaco?

◯ Yes
◯ No

If yes, On average, how many cigarettes did you have?

____
(cigarettes)

What type of cigarette?

☐ Cigarette
☐ E-Cigarette

If yes, On average, how many cigars did you have?

____
(cigars)

If yes, On average, how many pipes did you have?

____
(pipes)

If yes, On average, how many dips did you have?

____
(dips)

If yes, On average, how many chaws did you have?

____
(chaws)

74c. In the middle three months of your pregnancy, did you (smoke/use tobacco)?

En los mediados tres meses ¿hizo uso del tabaco?

◯ Yes
◯ No

If yes, On average, how many cigarettes did you have per day?

____
(cigarettes)

What type of cigarette?

☐ Cigarette
☐ E-Cigarette

If yes, On average, how many cigars did you have per day?

____
(cigars)

If yes, On average, how many pipes did you have per day?

____
(pipes)

If yes, On average, how many dips did you have per day?

____
(dips)

If yes, On average, how many chaws did you have per day?

____
(chaws)

74d. In the last three months of your pregnancy, did you (smoke/use tobacco)?<br><br>En los últimos tres meses ¿hizo uso del tabaco?	◯ Yes<br>◯ No
If yes, On average, how many cigarettes did you have you have per day?	____________________<br>(cigarettes)
What type of cigarette?	☐ Cigarette<br>☐ E-Cigarette
If yes, On average, how many cigars did you have you have per day?	____________________<br>(cigars)
If yes, On average, how many pipes did you have you have per day?	____________________<br>(pipes)
If yes, On average, how many dips did you have you have per day?	____________________<br>(dips)
If yes, On average, how many chaws did you have you have per day?	____________________<br>(chaws)
75. How many people who live in you home smoke cigarettes (NOT counting yourself)?<br><br>¿Cuántas otras personas que viven en su casa fuman?	____________________
76. How many of them smoke inside the home?<br><br>¿Cuántas fuman dentro de su casa?	____________________
77. Total number of cigarettes smoking inside your home per day (not including amount subject smoked)<br><br>¿Qué es el número total de cigarrillos fumados dentro de su casa por día?	____________________<br>(cigs/day)

VIII. Alcohol and Drug Use

Alcohol y uso de drogas

I'd like to ask you some questions about alcohol and drinking.

Me gustaría hacerle algunas preguntas sobre el alcohol y drogas

78. In the six months before you found out you were pregnant, how often did you drink?<br><br>En los 6 meses antes de embarazarse, ¿con que regularidad bebía?<br><br>Nunca<br>Ocasionalmente<br>Regularmente	◯ Never<br>◯ Occasionally(special occasions/holidays)<br>◯ Regularly
How many drinks did you have in a typical week?<br><br>¿Cuántas bebidas tomaba a lo largo de una semana típica?	________________________
What type drinks were they?<br>Beers or wine coolers<br><br>Cervezas	________________________<br>(beers or wine coolers)
What type of drinks were they?<br>Glasses of wine<br><br>Tazas de vino	________________________<br>(glasses of wine)
what type of drinks were they?<br>Shots of liquor<br><br>Shots de licor	________________________<br>(shots of liquor)
What type of drinks were they?<br>Mixed drinks<br><br>Mezcladas	________________________<br>(Mixed drinks)
IF MIXED DRINKS: How much alcohol was in each drink?<br><br>Cuánto alcohol había en cada bebida	________________________
79. In the first three months of your pregnancy, how often did you drink?<br><br>En los primeros tres meses, ¿con que regularidad bebía?	◯ Never<br>◯ Occasionally(special occasions/holidays)<br>◯ Regularly
How many drinks did you have in a typical week?	________________________<br>(drinks)
What type drinks were they?<br>Beers or wine coolers	________________________<br>(beers or wine coolers)
What type of drinks were they?<br>Glasses of wine	________________________<br>(glasses of wine)

what type of drinks were they?<br>Shots of liquor	______________________<br>(shots of liquor)
What type of drinks were they?<br>Mixed drinks	______________________<br>(Mixed drinks)
IF MIXED DRINKS: How much alcohol was in each drink?	______________________
80. In the middle three months of your pregnancy, how often did you drink?<br><br>En los promedios tres meses, ¿con que regularidad bebía?	◯ Never<br>◯ Occasionally(special occasions/holidays)<br>◯ Regularly
How many drinks did you have in a typical week?	______________________<br>(drinks)
What type drinks were they?<br>Beers or wine coolers	______________________<br>(beers or wine coolers)
What type of drinks were they?<br>Glasses of wine	______________________<br>(glasses of wine)
what type of drinks were they?<br>Shots of liquor	______________________<br>(shots of liquor)
What type of drinks were they?<br>Mixed drinks	______________________<br>(Mixed drinks)
IF MIXED DRINKS: How much alcohol was in each drink?	______________________
81. In the last three months of your pregnancy, how often did you drink?<br><br>En los últimos tres meses, ¿con que regularidad bebía?	◯ Never<br>◯ Occasionally(special occasions/holidays)<br>◯ Regularly
How many drinks did you have in a typical week?	______________________<br>(drinks)
What type drinks were they?<br>Beers or wine coolers	______________________<br>(beers or wine coolers)
What type of drinks were they?<br>Glasses of wine	______________________<br>(glasses of wine)

what type of drinks were they?
Shots of liquor

____
(shots of liquor)

What type of drinks were they?
Mixed drinks

____
(Mixed drinks)

IF MIXED DRINKS: How much alcohol was in each drink?

____

82. Now I'd like to ask you some questions about drug use. Have you ever used..(read each one)

Ahora me gustaría hacerle algunas preguntas sobre el uso de drogas. ¿Ha usado alguna vez ...?

	Yes	No
Marijuana	◯	◯
Heroin / Heroiona	◯	◯
If yes, have you ever been on methadone treatment? / ¿Ha usado el tratamiento de metadona?	◯	◯
Cocaine / Cocaino	◯	◯
Crack	◯	◯
Speed/Amphetamines / Amphetamina	◯	◯
Paint/Glue Inhalant	◯	◯
PCP	◯	◯
Barbituates	◯	◯
Benzo's/Valium	◯	◯
Ecstasy	◯	◯
LSD/Hallucinogens / Halucinogenas	◯	◯
Oxycodone	◯	◯
Others	◯	◯

If others, specify drug

____

83. If any drug WITHIN 6 months pre-pregnancy and during CURRENT (index) pregnancy, fill out the chart below:
CODE: 1=occasional; 2=Regular; IF regular, write in amount X/week

¿Ha usado en los 6 meses antes de embarazarse?
Veces por semana

Marijuana use 6 months pre-pregnancy	◯ Occasional<br>◯ Regular<br>(6 mo pre-pregnancy)
Number of times used	______________________________<br>(x/wk)
Marijuana use 1st trimester	◯ Occasional<br>◯ Regular<br>(1st trimester)
Times used	______________________________<br>(x/wk)
Marijuana use 2nd trimester	◯ Occasional<br>◯ Regular<br>(2nd trimester)
Times used	______________________________<br>(x/wk)
Marijuana use 3rd trimester	◯ Occasional<br>◯ Regular<br>(3rd trimester)
Times used	______________________________<br>(x/wk)
Heroin use 6 months pre-pregnancy	◯ Occasional<br>◯ Regular<br>(6 mo pre-pregnancy)
Times used per week	______________________________<br>(x/wk)
Heroin use 1st trimester	◯ Occasional<br>◯ Regular<br>(1st trimester)
Times used per week	______________________________<br>(x/wk)

Heroin use 2nd trimester	◯ Occasional<br>◯ Regular<br>(2nd trimester)
Times used per week	__________________________<br>(x/wk)
Heroin use 3rd trimester	◯ Occasional<br>◯ Regular<br>(3rd trimester)
Times used per week	__________________________<br>(x/wk)
Methadone use 6 months pre-pregnancy	◯ Occasional<br>◯ Regular<br>(6 mo pre-pregnancy)
Methadone times per week	__________________________<br>(x/wk)
Methadone use 1st trimester	◯ Occasional<br>◯ Regular<br>(1st Trimester)
Methadone times per week	__________________________<br>(x/wk)
Methadone use 2nd trimester	◯ Occasional<br>◯ Regular<br>(2nd trimester)
Methadone times per week	__________________________<br>(x/wk)
Methadone use 3rd trimester	◯ Occasional<br>◯ Regular<br>(3rd trimester)
Methadone times per week	__________________________<br>(x/wk)
Cocaine use 6 months pre-pregnancy	◯ Occasional<br>◯ Regular<br>(6 mo pre-pregnancy)
Cocaine times used per week	__________________________<br>(x/wk)

Cocaine use 1st trimester	◯ Occasional<br>◯ Regular<br>(1st trimester)
Cocaine times used per week	__________________________<br>(x/wk)
Cocaine use 2nd trimester	◯ Occasional<br>◯ Regular<br>(2nd trimester)
Cocaine times used per week	__________________________<br>(x/wk)
Cocaine use 3rd trimester	◯ Occasional<br>◯ Regular<br>(3rd trimester)
Cocaine times used per week	__________________________<br>(x/wk)
Crack use 6 months pre-pregnancy	◯ Occasional<br>◯ Regular<br>(6 mo pre-pregnancy)
Crack Times used per week	__________________________<br>(x/wk)
Crack use 1st trimester	◯ Occasional<br>◯ Regular<br>(1st trimester)
Crack Times used per week	__________________________<br>(x/wk)
Crack use 2nd trimester	◯ Occasional<br>◯ Regular<br>(2nd trimester)
Crack Times used per week	__________________________<br>(x/wk)
Crack use 3rd trimester	◯ Occasional<br>◯ Regular<br>(3rd trimester)
Crack Times used per week	__________________________<br>(x/wk)

Speed/Amphetamine use 6 months pre-pregnancy	◯ Occasional<br>◯ Regular<br>(6 mo pre-pregnancy)
Speed/Amphetamine use per week	________________<br>(x/wk)
Speed/Amphetamine use 1st trimester	◯ Occasional<br>◯ Regular<br>(1st trimester)
Speed/Amphetamine use per week	________________<br>(x/wk)
Speed/Amphetamine use 2nd trimester	◯ Occasional<br>◯ Regular<br>(2nd trimester)
Speed/Amphetamine use per week	________________<br>(x/wk)
Speed/Amphetamine use 3rd trimester	◯ Occasional<br>◯ Regular<br>(3rd trimester)
Speed/Amphetamine use per week	________________<br>(x/wk)
Paint/Glue use 6 months pre-pregnancy	◯ Occasionally<br>◯ Regular<br>(6 mo pre-pregnancy)
Paint/Glue use per week	________________<br>(x/wk)
Paint/Glue use 1st trimester	◯ Occasionally<br>◯ Regular<br>(1st trimester)
Paint/Glue use per week	________________<br>(x/wk)
Paint/Glue use 2nd trimester	◯ Occasionally<br>◯ Regular<br>(2nd trimester)
Paint/Glue use per week	________________<br>(x/wk)

Paint/Glue use 3rd trimester	◯ Occasionally<br>◯ Regular<br>(3rd trimester)
Paint/Glue use per week	________________<br>(x/wk)
PCP use 6 months pre-pregnancy	◯ Occasionally<br>◯ Regular<br>(6 mo pre-pregnancy)
PCP use per week	________________<br>(X/week)
PCP use 1st trimester	◯ Occasionally<br>◯ Regular<br>(1st trimester)
PCP use per week	________________<br>(X/week)
PCP use 2nd trimester	◯ Occasionally<br>◯ Regular<br>(2nd trimester)
PCP use per week	________________<br>(X/week)
PCP use 3rd trimester	◯ Occasionally<br>◯ Regular<br>(3rd trimester)
PCP use per week	________________<br>(X/week)
Barbituates use 6 months pre-pregnancy	◯ Occasional<br>◯ Regular<br>(6 mo pre-pregnancy)
Barbituates use per week	________________<br>(X/wk)
Barbituates use 1st trimester	◯ Occasional<br>◯ Regular<br>(1st trimester)
Barituates use per week	________________<br>(X/wk)

Barbituates use 2nd trimester	◯ Occasional<br>◯ Regular<br>(2nd trimester)
Barituates use per week	______________________<br>(X/wk)
Barbituates use 3rd trimester	◯ Occasional<br>◯ Regular<br>(3rd trimester)
Barituates use per week	______________________<br>(X/wk)
Benzo's/Valium use 6 months pre-pregnancy	◯ Occasional<br>◯ Regular<br>(6 mo pre-pregnancy)
Benzo's/Valium use per week	______________________<br>(X/wk)
Benzo's/Valium use 1st trimester	◯ Occasional<br>◯ Regular<br>(1st trimester)
Benzo's/Valium use per week	______________________<br>(X/wk)
Benzo's/Valium use 2nd trimester	◯ Occasional<br>◯ Regular<br>(2nd trimester)
Benzo's/Valium use per week	______________________<br>(X/wk)
Benzo's/Valium use 3rd trimester	◯ Occasional<br>◯ Regular<br>(3rd trimester)
Benzo's/Valium use per week	______________________<br>(X/wk)
Ecstasy use 6 months pre-pregnancy	◯ Occasional<br>◯ Regular<br>(6 mo pre-pregnancy)
Ecstasy use per week	______________________<br>(x/wk)

Ecstasy use 1st trimester	◯ Occasional<br>◯ Regular<br>(1st trimester)
Ecstasy use per week	____________________<br>(x/wk)
Ecstasy use 2nd trimester	◯ Occasional<br>◯ Regular<br>(2nd trimester)
Ecstasy use per week	____________________<br>(x/wk)
Ecstasy use 3rd trimester	◯ Occasional<br>◯ Regular<br>(3rd trimester)
Ecstasy use per week	____________________<br>(x/wk)
LSD/Hallucinogen use during 6 months pre-pregnancy	◯ Occasional<br>◯ Regular<br>(6 mo pre-pregnancy)
LSD/Hallucinogen use per week	____________________<br>(X/wk)
LSD/Hallucinogen use during 1st trimester	◯ Occasional<br>◯ Regular<br>(1st trimester)
LSD/Hallucinogen use per week	____________________<br>(X/wk)
LSD/Hallucinogen use during 2nd trimester	◯ Occasional<br>◯ Regular<br>(2nd trimester)
LSD/Hallucinogen use per week	____________________<br>(X/wk)
LSD/Hallucinogen use during 3rd trimester	◯ Occasional<br>◯ Regular<br>(3rd trimester)
LSD/Hallucinogen use per week	____________________<br>(X/wk)

Other drug used during 6 months pre-pregnancy	◯ Occasional<br>◯ Regular<br>(6 mo pre-pregnancy)
Other drug use per week	______________________<br>(X/wk)
Other drug used during 1st trimester	◯ Occasional<br>◯ Regular<br>(1st trimester)
Other drug use per week	______________________<br>(X/wk)
Other drug used during 2nd trimester	◯ Occasional<br>◯ Regular<br>(2nd trimester)
Other drug use per week	______________________<br>(X/wk)
Other drug used during 3rd trimester	◯ Occasional<br>◯ Regular<br>(3rd trimester)
Other drug use per week	______________________<br>(X/wk)
If other, please specify drug:	______________________

IX: General Information

Información General

84. How much did you weigh when you were born? Pounds<br><br>¿Cuál fue su peso al nacer?	______________________<br>(pounds)
How much did you weigh when you were born? Ounces<br><br>¿Cuál fue su peso al nacer?	______________________<br>(Ounces)
How much did you weigh when you were born? Grams<br><br>¿Cuál fue su peso al nacer?	______________________<br>(grams)

85. Were you born Prematurely?<br><br>¿Nació prematuramente?	◯ Yes<br>◯ No
85a. If yes, at what gestation?<br><br>¿En qué semana?	______________________<br>(weeks)
86. What is the highest grade of school you have completed?<br><br>¿Cuál es su mayor nivel de escolaridad?	◯ No school/Elementary School<br>◯ Some secondary school (9th grade and above)<br>◯ High school graduate or GED<br>◯ Some College<br>◯ College Degree and above
87. Which one of these groups best describes your racial background?<br><br>¿Qué grupo describe su grupo racial?<br>Negro<br>Blanco<br>Hispánico<br>Asiático<br>Haitiano<br>Cabo Verdiano<br>Orto	◯ Black/African American<br>◯ White<br>◯ Hispanic<br>◯ Asian<br>◯ Haitian<br>◯ Cape Verdian<br>◯ Pacific Islander<br>◯ Other
88. Where were you born?<br><br>¿Dónde nació?	◯ U.S.<br>◯ Foreign country
If born in other country, specify:	______________________<br>(Place of Birth)
Mother Country Code	______________________<br>(Determine when able)
88a. IF FOREIGN BORN: How long have you lived in the U.S.<br><br>¿Cuánto tiempo ha vivido en los Estados Unidos?	______________________<br>(years)
88a. IF FOREIGN BORN: How long have you lived in the U.S.<br><br>¿Cuánto tiempo ha vivido en los Estados Unidos?	______________________<br>(Months)
IF FOREIGN BORN: How long have you lived in the U.S.	______________________<br>(days)
89. Where was your Mother Born?<br><br>¿Dónde nació su madre?	◯ U.S.<br>◯ Foreign Country (specify)

Mother's mother Other country

____
(Mother's mother)

Mother's mother country code

____

90. Where was your Father born?

¿Dónde nació su padre?

- ◯ U.S.
- ◯ Foreign Country (Specify)

Mother's father other country

____

Mother's father Country Code

____

91. What is your native language?

¿Cuál es su idioma nativo?

- ◯ English
- ◯ Spanish
- ◯ Haitian Creole
- ◯ French
- ◯ Portuguese
- ◯ Other

If not English: How would you rate your ability to speak English?

- ◯ Very Well
- ◯ Well
- ◯ Not Very Well
- ◯ Not at all

92. Will you answer some questions about your child's biological father?

¿Va a contestar algunas preguntas sobre el padre biológico de su hijo?

- ◯ Yes
- ◯ No

93. What is the highest grade of school the baby's father has completed?

¿Cuál es el mayor nivel de escolaridad que ha completada el padre del bebe?

- ◯ No school/Elementary School
- ◯ Some Secondary School (9th grade and above)
- ◯ High School Graduate or GED
- ◯ Some College
- ◯ College Degree and Above

94. Which on of these groups best describes the racial background of the baby's father?

¿Cuál de estos grupos describe mejor el grupo racial de padre?
Negro
Blanco
Hispánico
Asiático
Haitiano
Cabo Verdiano
Orto

- ◯ Black/African American
- ◯ White
- ◯ Hispanic
- ◯ Asian
- ◯ Haitian
- ◯ Cape Verdian
- ◯ Pacific Island
- ◯ Other

95. Where was the baby's father born?

¿Dónde nació el padre de bebe?

- ◯ U.S.
- ◯ Foreign Country (specify)

Baby's father other country	__________________________________
Baby's Father Country Code	__________________________________
96. What is your present marital status?<br><br>¿Cuál es su estado marital?<br>Casa<br>Viuda<br>Divorciada<br>Separada<br>Soltera	◯ Married<br>◯ Widowed<br>◯ Divorced<br>◯ Separated<br>◯ Single
97. What was your total household income last year, before taxes? (Includes public assistance)<br><br>¿Cuál fue su ganancia financiero el último año antes de los impuestos?	◯ < $5,000<br>◯ $5000-9,999<br>◯ $10,000-14,999<br>◯ $15,000-19,999<br>◯ $20,000-24,999<br>◯ $25,000-29,999<br>◯ $30,000-34,999<br>◯ $35,000-39,999<br>◯ $40,000-49,999<br>◯ $50,000-59,000<br>◯ $60,000 and over<br>◯ Don't Know
Please ask if mother does not know annual income only: What is your weekly income?<br><br>¿O salario semanal?	__________________________________

98. Are you getting any public assistance?

¿Tiene algún tipo de asistencia publica?

Are you getting: WIC	◯ Yes<br>◯ No
Are you getting: Food Stamps	◯ Yes<br>◯ No
Are you getting: AFDC	◯ Yes<br>◯ No
Are you getting: Housing Assistance	◯ Yes<br>◯ No
Are you getting: Fuel Assistance	◯ Yes<br>◯ No
Are you getting: any other public assistance	◯ Yes<br>◯ No
if other specify	__________________________________

99. Did you take prenatal vitamins prescribed by your doctor?<br><br>¿Tomó vitaminas prenatales prescritas por su doctor?	◯ Yes<br>◯ No
a. Pre-pregnancy	◯ No<br>◯ < 1x/wk<br>◯ 1-2x/wk<br>◯ 3-5x/wk<br>◯ Almost Daily<br>(pre-pregnancy)
b. 1st Trimester	◯ No<br>◯ < 1x/wk<br>◯ 1-2x/wk<br>◯ 3-5x/wk<br>◯ Almost Daily<br>(1st trimester)
c. 2nd Trimester	◯ No<br>◯ < 1x/wk<br>◯ 1-2x/wk<br>◯ 3-5x/wk<br>◯ Almost Daily<br>(2nd trimester)
d. 3rd Trimester	◯ No<br>◯ < 1x/wk<br>◯ 1-2x/wk<br>◯ 3-5x/wk<br>◯ Almost Daily<br>(3rd trimester)
100. Did you take iron?<br><br>¿Tomó hierro?	◯ Yes<br>◯ No
a. Pre-pregnancy	◯ No<br>◯ < 1x/wk<br>◯ 1-2x/wk<br>◯ 3-5x/wk<br>◯ Almost Daily<br>(Pre-pregnancy)
b. 1st trimester	◯ No<br>◯ < 1x/wk<br>◯ 1-2x/wk<br>◯ 3-5x/wk<br>◯ Almost Daily<br>(1st trimester)
c. 2nd trimester	◯ No<br>◯ < 1x/wk<br>◯ 1-2x/wk<br>◯ 3-5x/wk<br>◯ Almost Daily<br>(2nd trimester)

d. 3rd trimester	◯ No<br>◯ < 1x/wk<br>◯ 1-2x/wk<br>◯ 3-5x/wk<br>◯ Almost Daily<br>(3rd trimester)
101. Did you take any over the counter vitamins?<br><br>¿Tomó otras vitaminas sin prescripción?	◯ Yes<br>◯ No
a. Pre-pregnancy	◯ No<br>◯ < 1x/wk<br>◯ 1-2x/wk<br>◯ 3-5x/wk<br>◯ Almost Daily<br>(pre-pregnancy)
b. 1st trimester	◯ No<br>◯ < 1x/wk<br>◯ 1-2x/wk<br>◯ 3-5x/wk<br>◯ Almost Daily<br>(1st trimester)
c. 2nd trimester	◯ No<br>◯ < 1x/wk<br>◯ 1-2x/wk<br>◯ 3-5x/wk<br>◯ Almost Daily<br>(2nd trimester)
d. 3rd trimester	◯ No<br>◯ < 1x/wk<br>◯ 1-2x/wk<br>◯ 3-5x/wk<br>◯ Almost Daily<br>(3rd trimester)
Name of over the counter vitamin	______________________________
102. Did you take any herbal supplements?<br><br>¿Tomó algún suplemento herbal?	◯ Yes<br>◯ No
a. Pre-pregnancy	◯ No<br>◯ < 1x/wk<br>◯ 1-2x/wk<br>◯ 3-5x/wk<br>◯ Almost daily<br>(pre-pregnancy)
b. 1st trimester	◯ No<br>◯ < 1x/wk<br>◯ 1-2x/wk<br>◯ 3-5x/wk<br>◯ Almost daily<br>(1st trimester)

c. 2nd trimester	◯ No<br>◯ < 1x/wk<br>◯ 1-2x/wk<br>◯ 3-5x/wk<br>◯ Almost daily<br>(2nd trimester)
d. 3rd trimester	◯ No<br>◯ < 1x/wk<br>◯ 1-2x/wk<br>◯ 3-5x/wk<br>◯ Almost daily<br>(3rd trimester)
Name of herbal supplement	________________________
Name of herbal supplement	________________________
Name of herbal supplement	________________________

103. During this pregnancy, on average, how often do you eat or drink following foods per week?

¿Durante este embarazo en promedio con qué frecuencia comió los siguientes alimentos?

During this pregnancy, on average, how often do you eat or drink following foods per week?<br>a. Green Vegetables<br><br>Vegetales verdes	◯ None<br>◯ < 1 days<br>◯ 1-2 days<br>◯ 3-5 days<br>◯ 6-7 days<br>◯ don't know
During this pregnancy, on average, how often do you eat or drink following foods per week?<br>b. Orange Vegetables(carrots, squash, etc)<br><br>Vegetales naranjas	◯ None<br>◯ < 1 days<br>◯ 1-2 days<br>◯ 3-5 days<br>◯ 6-7 days<br>◯ don't know
During this pregnancy, on average, how often do you eat or drink following foods per week?<br>c. Fruits<br><br>Frutas	◯ None<br>◯ < 1 days<br>◯ 1-2 days<br>◯ 3-5 days<br>◯ 6-7 days<br>◯ don't know
During this pregnancy, on average, how often do you eat or drink following foods per week?<br>d. Meat<br><br>Carnes	◯ None<br>◯ < 1 days<br>◯ 1-2 days<br>◯ 3-5 days<br>◯ 6-7 days<br>◯ don't know

During this pregnancy, on average, how often do you eat or drink following foods per week?<br>e. Shellfish<br><br>Mariscos	◯ None<br>◯ < 1 days<br>◯ 1-2 days<br>◯ 3-5 days<br>◯ 6-7 days<br>◯ don't know
During this pregnancy, on average, how often do you eat or drink following foods per week?<br>f. Fish<br><br>Pescado	◯ None<br>◯ < 1 days<br>◯ 1-2 days<br>◯ 3-5 days<br>◯ 6-7 days<br>◯ don't know
During this pregnancy, on average, how often do you eat or drink following foods per week?<br>g. Eggs<br><br>Huevos	◯ None<br>◯ < 1 days<br>◯ 1-2 days<br>◯ 3-5 days<br>◯ 6-7 days<br>◯ don't know
During this pregnancy, on average, how often do you eat or drink following foods per week?<br>h. Cow's Milk/Dairy Products/Cheese<br><br>Productos lácteos (Leche/queso)	◯ None<br>◯ < 1 days<br>◯ 1-2 days<br>◯ 3-5 days<br>◯ 6-7 days<br>◯ don't know
During this pregnancy, on average, how often do you eat or drink following foods per week?<br>i. beans<br><br>Frijoles	◯ None<br>◯ < 1 days<br>◯ 1-2 days<br>◯ 3-5 days<br>◯ 6-7 days<br>◯ don't know
During this pregnancy, on average, how often do you eat or drink following foods per week?<br>j. Rice<br><br>Arroz	◯ None<br>◯ < 1 days<br>◯ 1-2 days<br>◯ 3-5 days<br>◯ 6-7 days<br>◯ don't know
During this pregnancy, on average, how often do you eat or drink following foods per week?<br>k. Wheat(pasta, bread, cereal)<br><br>Trigo (pan/pasta)	◯ None<br>◯ < 1 days<br>◯ 1-2 days<br>◯ 3-5 days<br>◯ 6-7 days<br>◯ don't know
During this pregnancy, on average, how often do you eat or drink following foods per week?<br>l. Soy/Tofu<br><br>Soya/Tofu	◯ None<br>◯ < 1 days<br>◯ 1-2 days<br>◯ 3-5 days<br>◯ 6-7 days<br>◯ don't know

During this pregnancy, on average, how often do you eat or drink following foods per week?<br>m. Seeds(Sesame, Sunflower, Pumpkin)<br><br>Semillas	◯ None<br>◯ < 1 days<br>◯ 1-2 days<br>◯ 3-5 days<br>◯ 6-7 days<br>◯ don't know
During this pregnancy, on average, how often do you eat or drink following foods per week?<br>n. Calcium Fortified Juice<br><br>Jugo fortificado con calcio	◯ None<br>◯ < 1 days<br>◯ 1-2 days<br>◯ 3-5 days<br>◯ 6-7 days<br>◯ don't know
During this pregnancy, on average, how often do you eat or drink following foods per week?<br>o. Peanut<br><br>Maní	◯ None<br>◯ < 1 days<br>◯ 1-2 days<br>◯ 3-5 days<br>◯ 6-7 days<br>◯ don't know
During this pregnancy, on average, how often do you eat or drink following foods per week?<br>p. Tree nuts<br><br>Nuezes	◯ None<br>◯ < 1 days<br>◯ 1-2 days<br>◯ 3-5 days<br>◯ 6-7 days<br>◯ don't know
104. Did you drink coffee before or during the index pregnancy?<br><br>¿Tomó café antes o durante el embarazo?	◯ Yes<br>◯ No
If yes, was it regular or decaffeinated?<br><br>¿Era regular o descafeinado?<br>¿Ambos?	◯ Regular<br>◯ Decaffeinated<br>◯ Both
Number of regular cups per week: Pre-pregnancy<br><br>¿Cuántas tazas bebía a la semana?<br>Pre-emarazo	______________________
Number of regular cups per week: 1st Trimester<br><br>¿Cuántas tazas bebía a la semana?<br>Primer Trimestre	______________________
Number of regular cups per week: 2nd Trimester<br><br>¿Cuántas tazas bebía a la semana?<br>Segundo Trimestre	______________________
Number of regular cups per week: 3rd Trimester<br><br>¿Cuántas tazas bebía a la semana?<br>Tercer Trimestre	______________________

Number of decaf cups per week: Pre-pregnancy<br><br>¿Cuántas tazas bebía a la semana?<br>Pre-emarazo	__________________________________
Number of decaf cups per week: 1st trimester<br><br>¿Cuántas tazas bebía a la semana?<br>Primer Trimestre	__________________________________
Number of decaf cups per week: 2nd trimester<br><br>¿Cuántas tazas bebía a la semana?<br>Segundo Trimestre	__________________________________
Number of decaf cups per week: 3rd trimester<br><br>¿Cuántas tazas bebía a la semana?<br>Tercer Trimestre	__________________________________
105. Did you drink tea before or during the index pregnancy?<br><br>¿Bebió té antes o durante el embarazo?	◯ Yes<br>◯ No
If yes, was it regular tea or herbal tea?	◯ Regular<br>◯ Herbal<br>◯ Both
Number of regular cups per week: Pre-pregnancy<br><br>¿Cuántas tazas bebía a la semana?<br>Pre-emarazo	__________________________________<br>(cups/wk)
Number of regular cups per week: 1st trimester<br><br>¿Cuántas tazas bebía a la semana?<br>Primer Trimestre	__________________________________<br>(cups/wk)
Number of regular cups per week: 2nd trimester<br><br>¿Cuántas tazas bebía a la semana?<br>Segundo Trimestre	__________________________________<br>(cups/wk)
Number of regular cups per week: 3rd trimester<br><br>¿Cuántas tazas bebía a la semana?<br>Tercer Trimestre	__________________________________<br>(cups/wk)
Number of herbal cups per week: Pre-pregnancy<br><br>¿Cuántas tazas bebía a la semana?<br>Pre-emarazo	__________________________________<br>(cups/wk)
Number of herbal cups per week: 1st trimester<br><br>¿Cuántas tazas bebía a la semana?<br>Primer Trimestre	__________________________________<br>(cups/wk)

Number of herbal cups per week: 2nd trimester<br><br>¿Cuántas tazas bebía a la semana?<br>Segundo Trimestre	______________________<br>(cups/wk)
Number of herbal cups per week: 3rd trimester<br><br>¿Cuántas tazas bebía a la semana?<br>Tercer Trimestre	______________________<br>(cups/wk)
106. Did you drink soft drinks during the index pregnancy?<br><br>¿Bebió sodas o refrescos antes o durante el embarazo?	◯ Yes<br>◯ No
If yes, what kinds?<br><br>¿Cafeinado o Descafeinado? ¿Ambos?	◯ Caffeinated<br>◯ Decaf<br>◯ Both
Number of cups of soda per week: Pre-pregnancy<br><br>¿Cuántas tazas bebía a la semana?<br>Pre-emarazo	______________________<br>(cups/wk)
Number of cups of soda per week: 1st trimester<br><br>¿Cuántas tazas bebía a la semana?<br>Primer Trimestre	______________________<br>(cups/wk)
Number of cups of soda per week: 2nd trimester<br><br>¿Cuántas tazas bebía a la semana?<br>Segundo Trimestre	______________________<br>(cups/wk)
Number of cups of soda per week: 3rd trimester<br><br>¿Cuántas tazas bebía a la semana?<br>Tercer Trimestre	______________________<br>(cups/wk)
107. How do you plan to feed your baby?<br><br>¿Cómo planea alimentar al bebé?<br>Pecho solamente<br>Formula solamente<br>Ambos pecho y formula<br>No lo sé aún	◯ Breast Feed Only<br>◯ Formula Feed Only<br>◯ Both Breast Feed and Formula Feed<br>◯ Don't Know Yet

XI. Medical History

108. What medicines did you take during your pregnancy excluding vitamins?

¿Qué medicamentos tomó durante su embarazo excluyendo vitaminas?

a. Medication name 1 ______________________________

Used in first trimester
- ◯ Yes
- ◯ No
- ◯ Unsure

Used in second trimester
- ◯ Yes
- ◯ No
- ◯ Unsure

Used in third trimester
- ◯ Yes
- ◯ No
- ◯ Unsure

b. Medication name 2 ______________________________

Used in first trimester
- ◯ Yes
- ◯ No
- ◯ Unsure

Used in second trimester
- ◯ Yes
- ◯ No
- ◯ Unsure

Used in third trimester
- ◯ Yes
- ◯ No
- ◯ Unsure

c. Medication name 3 ______________________________

Used in first trimester
- ◯ Yes
- ◯ No
- ◯ Unsure

Used in second trimester
- ◯ Yes
- ◯ No
- ◯ Unsure

Used in third trimester
- ◯ Yes
- ◯ No
- ◯ Unsure

d. Medication name 4 ______________________________

Used in first trimester	◯ Yes<br>◯ No<br>◯ Unsure
Used in second trimester	◯ Yes<br>◯ No<br>◯ Unsure
Used in third trimester	◯ Yes<br>◯ No<br>◯ Unsure
e. Medication name 5	__________________________________
Used in first trimester	◯ Yes<br>◯ No<br>◯ Unsure
Used in second trimester	◯ Yes<br>◯ No<br>◯ Unsure
Used in third trimester	◯ Yes<br>◯ No<br>◯ Unsure

BMC Children's Health Study

Self-reported pubertal development questionnaire for ages 6 and older

Introduction for research staff

This questionnaire is used to assess pubertal development for boys and girls aged 6 years and older. Although both child and mother can answer the questions, please let child complete the questionnaire as much as possible.

This questionnaire includes three types of questions:

1. For multiple choice questions, please circle one answer.
2. For fill in blank questions, please fill in appropriate number.
3. For figures, please request participants to (1) "choose the drawing closest to your stage of development and mark 1" ; (2) "choose the drawing next closest to your stage of development and mark 2." Written descriptions explain the drawings.

   You may skip any question(s) you are uncomfortable answering. If you are unsure of an answer, just answer as best you can.
   You can still participate in this study even if you choose not to answer the questions about puberty.

BMC Children’s Health Study

Self-reported pubertal development questionnaire for ages 6 and older

Introduction for participants

You are being asked to complete a questionnaire that asks you about puberty- that is the changes that happen to how our bodies look as we grow up and mature, Some of the questions we will ask, you read and circle your answer and others you answer from looking at a picture to pick the drawing that looks most like your body or second most like your body. Your mother can help you answer the questions, but we ask that you try to answer the questions yourself if you can. After you answer the questions, you can put the form in an envelope and return to us so no one can see how you answered the questions.

Study ID#____________
Date________________

BMC Children's Health Study

Self-reported pubertal development questionnaire for Boys ages 6 and older (circle one answer)

1. How much underarm hair do you have now?
   a. None at all b. There is a little soft hair c. The hair is dark, thick and curly d. The hair covers the whole armpit
2. How much hair do you have on your face now?
   a. None at all b. There is a little soft hair c. The hair is thick d. The hair covers as much of the face as an adult
3. Do you shave?
   a. Yes b. No
   If yes, how old were you when you first shaved? _____years___months old.
   If no, skip to question 5.
4. How often do you shave face?
   a. None at all b. One time per week c. Twice per week d. 3-5 times per week e. Almost everyday
5. How much hair do you have on your abdomen/ lower belly?
   a. None at all b. A little c. A lot
6. How much hair do you have on your legs?
   a. None at all b. A little c. A lot
7. Do the clothes you wore last year still fit?
   a. No b. Yes

USE THE DRAWINGS TO ANSWER THESE QUESTIONS

8. **In figure 1**, the drawings show the different amounts of male public hair. A boy passes through each of the five stages in the drawings.

8a. Which drawing is closest to your stage of hair development?
Drawing A Drawing B Drawing C Drawing D Drawing E

8b. Which drawing is next closest to your stage of hair development?
Drawing A Drawing B Drawing C Drawing D Drawing E

9. **In figure 2**, the drawings show the different stage of development of the Testes, scrotum, and penis. A boy passes through each of the five stages shown by these drawings.

9a. Which drawing is closest to your stage of development?
Drawing A Drawing B Drawing C Drawing D Drawing E

9b. Which drawing is next closest to your stage of development?
Drawing A Drawing B Drawing C Drawing D Drawing E

10. Who completed the form?
    a. Child b. Mother c. Both child and mother

Study ID#____________
Date_________________

Figure 1. The development of pubic hair for **questions 8a and 8b**

The drawings on this page show different amounts of male pubic hair. A boy passes through each of the five stages shown by these drawings. Please look at each drawing and read the sentences under the drawing. Then choose the drawing closest to your stage of your hair development, circle it on question 8a. Then choose the drawing that is next closest to your stage of hair development and circle it on question 8b.

1. Drawing A	2. Drawing B	3. Drawing C	4. Drawing D	5. Drawing E
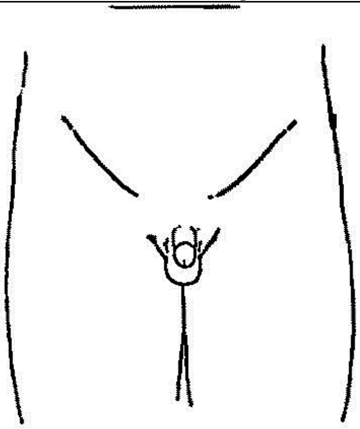	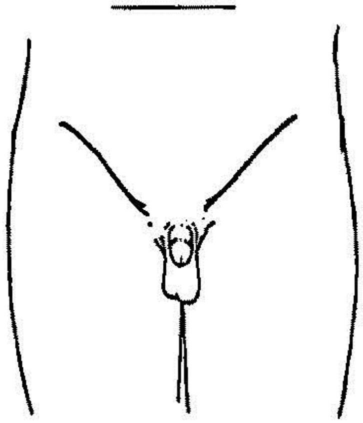	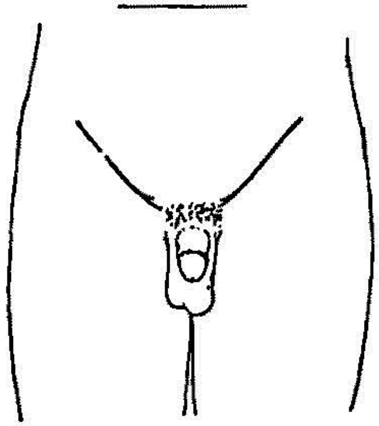	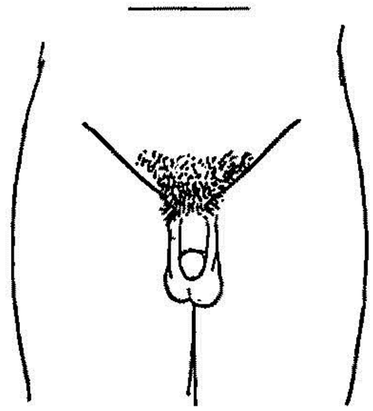	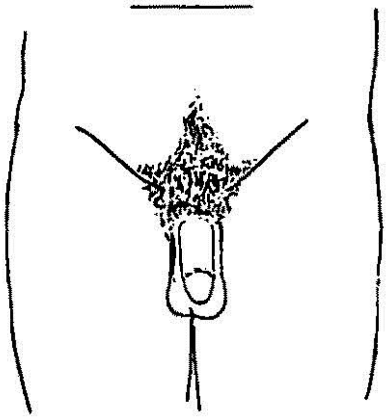
There is no pubic hair at all	There is a little soft, long, lightly colored hair, most of the hair is at the base of the penis. This hair may be straight or a little curly.	The hair is darker in this stage. It is coarser and more curled. It has spread out and thinly covers a somewhat larger area.	The hair is now as dark, curly, and coarse as that of an adult male. However, the area that the hair covers is not as large as that of an adult male. The hair has not spread out to the thighs.	The hair has spread out to the thighs. The hair is now like that of an adult male. It covers the same area as that of an adult male.

Study ID#____________
Date_________________

Figure 2. The development of the Testes, Scrotum and Penis for **questions 9a and 9b**

The drawings on this page show different stages of development of the Testes, Scrotum, and Penis. A boy passes through each of the five stages shown by these drawings. Please look at the drawings and read the sentences under the drawing. Then choose the drawing closest to your stage of development, circle it on question 9a. Then choose the drawing that is next closest to your stage of development and circle it on question 9b.

1. Drawing A	2. Drawing B	3. Drawing C	4. Drawing D	5. Drawing E
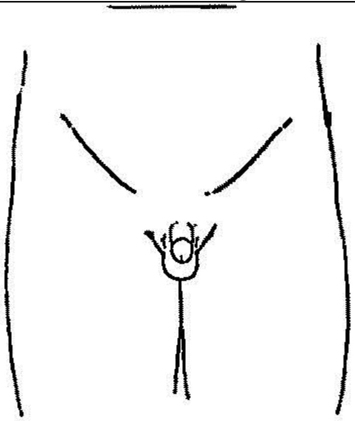	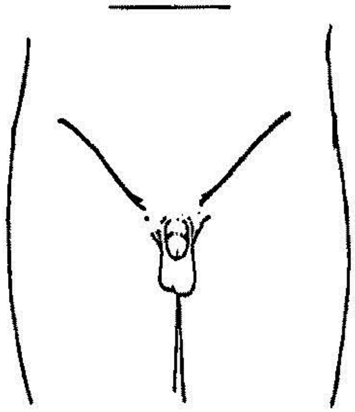	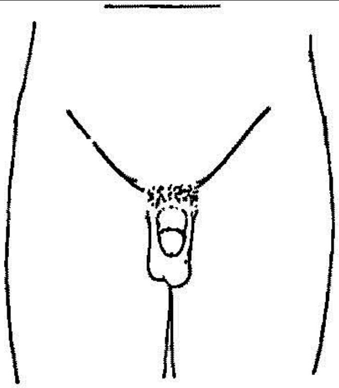	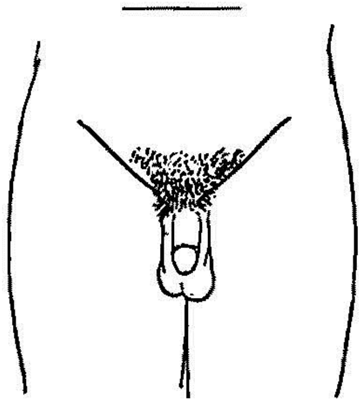	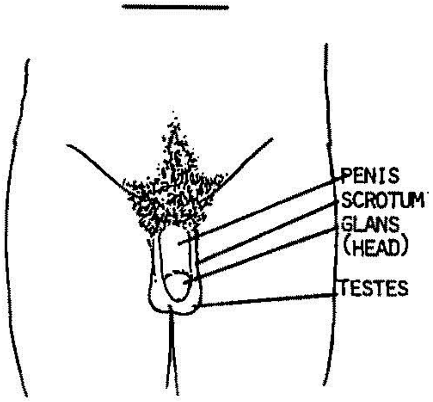
The testes, scrotum, and penis are about the same size and shape as they were when you are a child.	The testes and scrotum have gotten a little larger, the skin of the scrotum has changed, the scrotum, the sack holding the testes, has lowered a bit. The penis has gotten only a little larger.	The penis has grown mainly in length, the testes and scrotum have grown and dropped lower than in stage 2.	The penis has grown even larger, it is wider. The glans (the head of the penis) is bigger. The scrotum is darker than before. It is bigger because the testes have gotten bigger.	The penis, scrotum, and testes are the size and shape of that of an adult male.

Study ID#____________
Date________________

BMC Children's Health Study

Self-reported pubertal development questionnaire for Girls ages 6 and older (circle one answer)

1. Do you have your period?
   a. Yes b. No
   If yes, how old were you when you had your first period? _____years___months old,
   If no, skip to question 3.

2. Do you have pelvic or stomach/belly pain during your menstrual period?
   a. No b. Occasionally c. Almost all the time

2a. If you answered "Occasionally" or "Almost all the time", do you rate your menstrual pain as:
   a. Mild b. Moderate c. Severe (could not go to school or work)

3. How much underarm hair do you have now?
   a. None at all b. There is a little soft hair c. The hair is dark and thick d. The hair covers the whole armpit

4. How much hair do you have on your abdomen/lower belly?
   a. None at all b. A little c. A lot

5. How much hair do you have on your legs?
   a. None at all b. A little c. A lot

6. Do the clothes you wore last year still fit?
   a. No b. Yes

USE THE DRAWINGS TO ANSWER THESE QUESTIONS

7. **In figure 1**, the drawings show the different stage of breast development. A female passes through each of the five stages shown by these set of drawings.

7a. Which set of drawings is closest to your stage of breast development?
   Drawing A Drawing B Drawing C Drawing D Drawing E

7b. Which set of drawings is next closest to your stage of breast development?
   Drawing A Drawing B Drawing C Drawing D Drawing E

8. **In figure 2**, the drawings show the different amounts of public hair. A girl passes through each of the five stages shown by these drawings.

8a. Which drawing is closest to your stage of hair development?
   Drawing A Drawing B Drawing C Drawing D Drawing E

8b. Which drawing is next closest to your stage of hair development?
   Drawing A Drawing B Drawing C Drawing D Drawing E

9. Who completed the form?
   a. Child b. Mother c. Both child and mother

Study ID#____________

Date________________

Figure 1. The development of breast for **questions 7a and 7b**

The drawings on this page show different stage of development of the breasts. A female passes through each of the five stages shown by these set of drawings. Please look at each set of drawing and read the sentences under the drawing. Then choose the set of drawings closest to your stage of breast development, circle it on question 7a. Then choose the drawing that is next closest to your stage of breast development and circle it on question 7b.

1. Drawing A	2. Drawing B	3. Drawing C	4. Drawing D	5. Drawing E
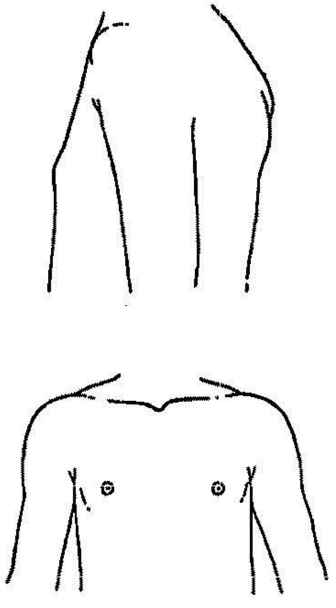	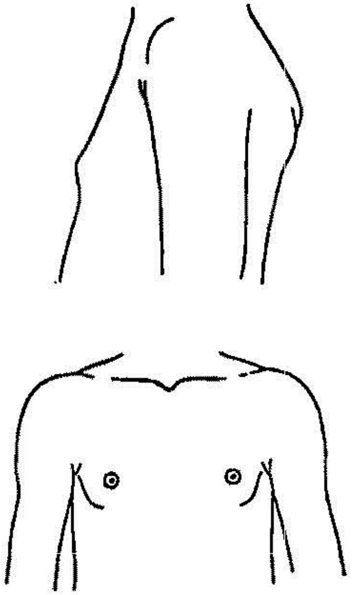	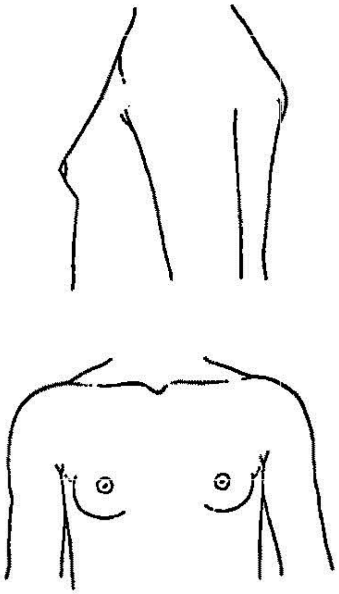	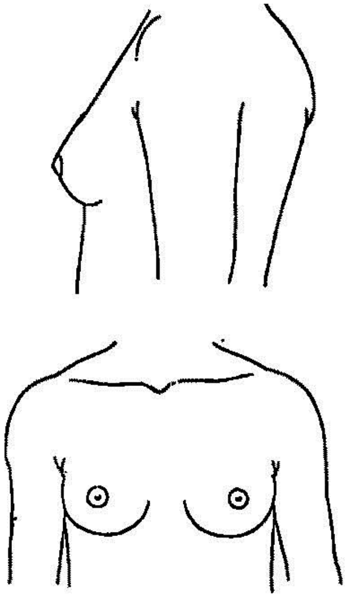	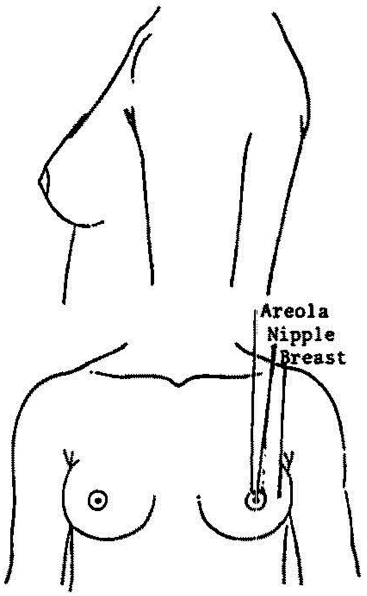
The nipple is raised a little in this stage. The rest of the breast is still flat.	This is the breast bud stage. In this stage, the nipple is raised more than in stage 1. The breast is a small mound. The areola is larger than in stage 1.	The areola and the breast are both larger than in stage2. The areola does not stick out away from the breast.	The areola and the nipple make up a mound that sticks up above the shape of the breast. (Note: this stage may not happen at all for some girls. Some girls develop from stage 3 to stage 5, with no stage 4).	This is the mature adult stage. The breasts are fully developed, only the nipple sticks out in this stage. The areola has moved back to the general shape of the breast.

Figure 2. The development of pubic hair for **questions 8a and 8b**

The drawings on this page show different amounts of female pubic hair. A girl passes through each of the five stages shown by these drawings. Please look at each drawing and read the sentences under the drawing. Then choose the drawing closest to your stage of your hair development, circle it on question 8a. Then choose the drawing that is next closest to your stage of hair development and circle it on question 8b.

1. Drawing A	2. Drawing B	3. Drawing C	4. Drawing D	5. Drawing E
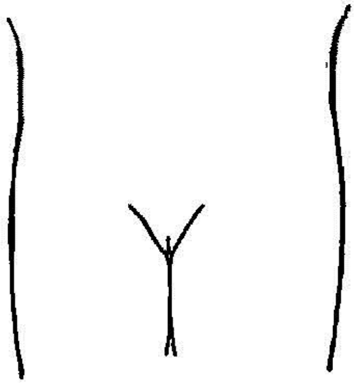	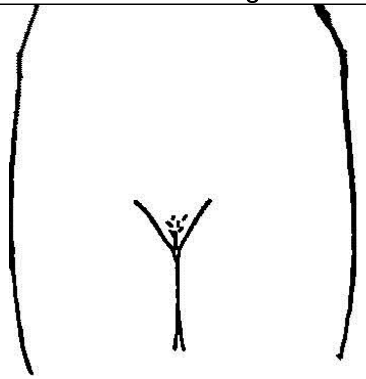	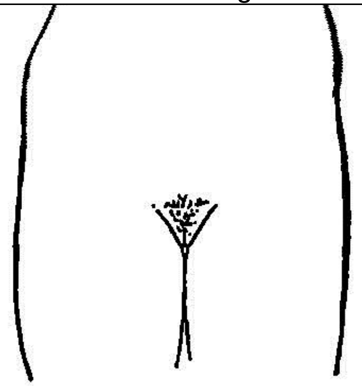	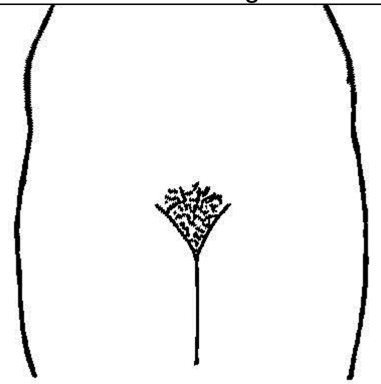	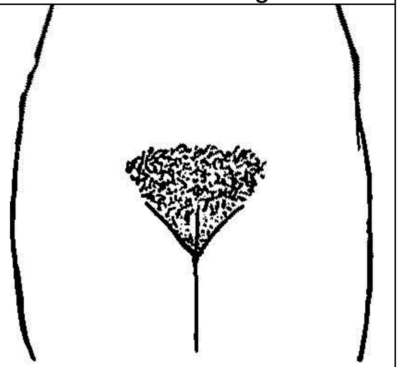
There is no pubic hair at all	There is a little soft, long, lightly colored hair. This hair may be straight or a little curly.	The hair is darker in this stage. It is coarser and more curled. It has spread out and thinly covers a larger area.	The hair is now as dark, curly, and coarse as that of an adult female. However, the area that the hair covers is not as large as that of an adult female. The hair has not spread out to the thighs.	The hair is now like that of an adult female. It also covers the same area as that of an adult female. The hair usually forms a triangle (∇) pattern as it spreads out to the thighs.

CURRENT

Social Communication Questionnaire (SCQ)

AutoScore™ Form

Michael Rutter, M.D., F.R.S., Anthony Bailey, M.D., Sibel Kazak Berument, Ph.D., Catherine Lord, Ph.D., and Andrew Pickles, Ph.D.

wps
Test with Confidence

Name of Subject ______

Date of Birth ______

Date of Interview ______

Chronological Age ______ ______F ______M Gender

Name of Respondent ______

Relation to Subject ______

Clinician Name ______

School/Clinic ______

Directions

Thank you for taking the time to complete this questionnaire. Please answer each question by circling *yes* or *no*. A few questions ask about several related types of behavior; please circle *yes* if *any* of these behaviors were present during the past 3 months. Although you may be uncertain about whether some behaviors were present or not, please answer *yes* or *no* to every question on the basis of what you think.

1. Is she/he now able to talk using short phrases or sentences? If *no*, skip to question 8. .... yes no
2. Do you have a to and fro "conversation" with her/him that involves taking turns or building on what you have said?........ yes no
3. Does she/he ever use odd phrases or say the same thing over and over in almost exactly the same way (either phrases that she/he hears other people use or ones that she/he makes up)?........ yes no
4. Does she/he ever use socially inappropriate questions or statements? For example, does she/he ever regularly ask personal questions or make personal comments at awkward times? .... yes no
5. Does she/he ever get her/his pronouns mixed up (e.g., saying *you* or *she/he* for *I*)? .... yes no
6. Does she/he ever use words that she/he seems to have invented or made up her/himself; put things in odd, indirect ways; or use metaphorical ways of saying things (e.g., saying *hot rain* for *steam*)?........ yes no
7. Does she/he ever say the same thing over and over in exactly the same way or insist that you say the same thing over and over again?........ yes no
8. Does she/he ever have things that she/he seems to have to do in a very particular way or order or rituals that she/he insists that you go through?........ yes no
9. Does her/his facial expression usually seem appropriate to the particular situation, as far as you can tell?........ yes no
10. Does she/he ever use your hand like a tool or as if it were part of her/his own body (e.g., pointing with your finger or putting your hand on a doorknob to get you to open the door)?........ yes no
11. Does she/he ever have any interests that preoccupy her/him and might seem odd to other people (e.g., traffic lights, drainpipes, or timetables)?........ yes no
12. Does she/he ever seem to be more interested in parts of a toy or an object (e.g., spinning the wheels of a car), rather than in using the object as it was intended? .... yes no
13. Does she/he ever have any special interests that are *unusual* in their intensity but otherwise appropriate for her/his age and peer group (e.g., trains or dinosaurs)? .... yes no
14. Does she/he ever seem to be *unusually* interested in the sight, feel, sound, taste, or smell of things or people? .... yes no
15. Does she/he ever have any mannerisms or odd ways of moving her/his hands or fingers, such as flapping or moving her/his fingers in front of her/his eyes? .... yes no
16. Does she/he ever have any complicated movements of her/his whole body, such as spinning or repeatedly bouncing up and down? .... yes no
17. Does she/he ever injure her/himself deliberately, such as by biting her/his arm or banging her/his head? .... yes no

Additional copies of this form (W-381A) may be purchased from WPS. Please contact us at **800.648.8857** or **www.wpspublish.com**.
Copyright © 2003 by Western Psychological Services. Not to be reproduced, adapted, and/or translated in whole or in part without prior written permission of WPS (rights@wpspublish.com). All rights reserved. Printed in USA.
4 5 6 7 8 9

W-381A

18. Does she/he ever have any objects (*other* than a soft toy or comfort blanket) that she/he *has* to carry around? .... yes no

19. Does she/he have any particular friends or a best friend? .... yes no

20. Does she/he ever talk with you just to be friendly (rather than to get something)? .... yes no

21. Does she/he ever *spontaneously* copy you (or other people) or what you are doing (such as vacuuming, gardening, or mending things)? .... yes no

22. Does she/he ever spontaneously point at things around her/him just to show you things (not because she/he wants them)? .... yes no

23. Does she/he ever use gestures, other than pointing or pulling your hand, to let you know what she/he wants? .... yes no

24. Does she/he nod her/his head to indicate *yes*? .... yes no

25. Does she/he shake her/his head to indicate *no*? .... yes no

26. Does she/he usually look at you directly in the face when doing things with you or talking with you? .... yes no

27. Does she/he smile back if someone smiles at her/him? .... yes no

28. Does she/he ever show you things that interest her/him to engage your attention? .... yes no

29. Does she/he ever offer to share things other than food with you? .... yes no

30. Does she/he ever seem to want you to join in her/his enjoyment of something? .... yes no

31. Does she/he ever try to comfort you if you are sad or hurt? .... yes no

32. If she/he wants something or wants help, does she/he look at you and use gestures with sounds or words to get your attention? .... yes no

33. Does she/he show a normal range of facial expressions? .... yes no

34. Does she/he ever spontaneously join in and try to copy the actions in social games, such as *The Mulberry Bush* or *London Bridge Is Falling Down*? .... yes no

35. Does she/he play any pretend or make-believe games? .... yes no

36. Does she/he seem interested in other children of approximately the same age whom she/he does not know? .... yes no

37. Does she/he respond positively when another child approaches her/him? .... yes no

38. If you come into a room and start talking to her/him without calling her/his name, does she/he usually look up and pay attention to you? .... yes no

39. Does she/he ever play imaginative games with another child in such a way that you can tell that each child understands what the other is pretending? .... yes no

40. Does she/he play cooperatively in games that need some form of joining in with a group of other children, such as hide-and-seek or ball games? .... yes no

wps
Test with Confidence

John N. Constantino, MD

Assessment ID ____________

SRS-2 AutoScore™ Form Preschool

○ MALE ○ FEMALE

INSTRUCTIONS

For each question, please darken the circle that best describes this child's behavior **over the past 6 months.**

Child's name ____________ Child's age ____ years ____ months

Rater's name ____________ Date of rating ____________

Relationship to rated individual ☐ Mother ☐ Father ☐ Other custodial adult ☐ Teacher ☐ Other specialist

School or clinic ____________

PLEASE PRESS HARD WHEN MARKING YOUR RESPONSES.

1 = NOT TRUE 2 = SOMETIMES TRUE 3 = OFTEN TRUE 4 = ALMOST ALWAYS TRUE

1. Seems much more fidgety in social situations than when alone. ① ② ③ ④
2. Expressions on his or her face don't match what he or she is saying. ① ② ③ ④
3. Seems self-confident when interacting with others. ① ② ③ ④
4. When under stress, child seems to go on "autopilot" (for example, shows rigid or inflexible patterns of behavior that seem odd). ① ② ③ ④
5. Doesn't recognize when others are trying to take advantage of him or her. ① ② ③ ④
6. Would rather be alone than with others. ① ② ③ ④
7. Is aware of what others are thinking or feeling. ① ② ③ ④
8. Behaves in ways that seem strange or bizarre. ① ② ③ ④
9. Clings to adults, seems too dependent on them. ① ② ③ ④
10. Unable to pick up on any of the meaning of conversations of older children or adults. ① ② ③ ④
11. Has good self-confidence. ① ② ③ ④
12. Is able to communicate his or her feelings to others in words or gestures. ① ② ③ ④
13. Is slow or awkward in turn-taking interactions with peers. ① ② ③ ④
14. Is not well coordinated in physical activities. ① ② ③ ④
15. Is able to understand the meaning of other people's tone of voice and facial expressions. ① ② ③ ④
16. Avoids eye contact or has unusual eye contact. ① ② ③ ④
17. Recognizes when something is unfair. ① ② ③ ④
18. When on the playground or in a group with other young children, child does not attempt to interact with other children. ① ② ③ ④
19. Gets frustrated trying to get ideas across in conversations. ① ② ③ ④
20. Has a strange way of playing with a toy. ① ② ③ ④
21. Is able to imitate others' actions. ① ② ③ ④
22. Plays appropriately with children his or her age. ① ② ③ ④
23. Does not join group activities unless told to do so. ① ② ③ ④
24. Has more difficulty than other children with changes in his or her routine. ① ② ③ ④
25. Doesn't seem to mind being out of step with or "not on the same wavelength" as others. ① ② ③ ④
26. Offers comfort to others when they are sad. ① ② ③ ④
27. Avoids starting social interactions with peers or adults. ① ② ③ ④
28. Thinks or talks about the same thing over and over. ① ② ③ ④
29. Is regarded by other children as odd or weird. ① ② ③ ④
30. Becomes upset in a situation with lots of things going on. ① ② ③ ④
31. Can't get his or her mind off something once he or she starts thinking about it. ① ② ③ ④
32. Wants to be changed when diaper or underwear is soiled or wet. ① ② ③ ④

Continue on back page

Additional copies of this form (W-608B) may be purchased from WPS. Please contact us at **800.648.8857** or **www.wpspublish.com**.
Copyright © 2012 by Western Psychological Services. Not to be reproduced, adapted, and/or translated in whole or in part without prior written permission of WPS (rights@wpspublish.com). All rights reserved. Printed in USA. 2 3 4 5 6 7 8 9

PLEASE PRESS HARD WHEN MARKING YOUR RESPONSES.

1 = NOT TRUE   2 = SOMETIMES TRUE   3 = OFTEN TRUE   4 = ALMOST ALWAYS TRUE

33. Is socially awkward, even when he or she is trying to be polite. .... ① ② ③ ④
34. Avoids people who want to be emotionally close to him or her. .... ① ② ③ ④
35. Has trouble keeping up with the flow of normal interaction with other children. .... ① ② ③ ④
36. Has difficulty relating to adults. .... ① ② ③ ④
37. Has difficulty relating to peers. .... ① ② ③ ④
38. Responds appropriately to mood changes in others (for example, when a friend's or playmate's mood changes from happy to sad). .... ① ② ③ ④
39. Has a restricted (or unusually narrow) range of interests. .... ① ② ③ ④
40. Is imaginative, good at pretending (without losing touch with reality). .... ① ② ③ ④
41. Wanders aimlessly from one activity to another. .... ① ② ③ ④
42. Seems overly sensitive to sounds, textures, or smells. .... ① ② ③ ④
43. Separates easily from caregivers. .... ① ② ③ ④
44. Doesn't understand how events are related to one another the way other children his or her age do. .... ① ② ③ ④
45. Focuses his or her attention to where others are looking or listening. .... ① ② ③ ④
46. Has overly serious facial expressions. .... ① ② ③ ④
47. Is too silly or laughs inappropriately. .... ① ② ③ ④
48. Has a sense of humor, understands jokes. .... ① ② ③ ④
49. Does extremely well at a few tasks, but does not do as well at most other tasks. .... ① ② ③ ④
50. Has repetitive, odd behaviors such as hand flapping or rocking. .... ① ② ③ ④
51. Responds to clear, direct questions in ways that don't seem to make any sense. .... ① ② ③ ④
52. Knows when he or she is talking too loud or making too much noise. .... ① ② ③ ④
53. Talks to people with an unusual tone of voice (for example, talks like a robot or like he or she is giving a lecture). .... ① ② ③ ④
54. Seems to react to people as if they are objects. .... ① ② ③ ④
55. Knows when he or she is too close to someone or is invading someone's space. .... ① ② ③ ④
56. Walks in between two people who are talking. .... ① ② ③ ④
57. Other children do not like to play with him or her. .... ① ② ③ ④
58. Concentrates too much on parts of things rather than seeing the whole picture. For example, spins the wheels of a toy car but doesn't play with it as a car, or plays with doll's hair but not with the whole doll. .... ① ② ③ ④
59. Is overly suspicious. .... ① ② ③ ④
60. Is emotionally distant, doesn't show his or her feelings. .... ① ② ③ ④
61. Is inflexible, has a hard time changing his or her mind. .... ① ② ③ ④
62. Gives unusual or illogical reasons for doing things. .... ① ② ③ ④
63. Touches others in an unusual way (for example, he or she may touch someone just to make contact and then walk away without saying anything). .... ① ② ③ ④
64. Is too tense in social settings. .... ① ② ③ ④
65. Stares or gazes off into space. .... ① ② ③ ④

wps
Test with Confidence

John N. Constantino, MD

Assessment ID ____________

SRS-2 AutoScore™ Form School-Age ◯MALE ◯FEMALE

INSTRUCTIONS

For each question, please darken the circle that best describes this child's behavior **over the past 6 months.**

Child's name ____________ Child's age in years ____________

Rater's name ____________ Date of rating ____________

Relationship to rated individual ☐ Mother ☐ Father ☐ Other custodial adult ☐ Teacher ☐ Other specialist

Grade ____________ School or clinic ____________

PLEASE PRESS HARD WHEN MARKING YOUR RESPONSES.

1 = NOT TRUE 2 = SOMETIMES TRUE 3 = OFTEN TRUE 4 = ALMOST ALWAYS TRUE

1. Seems much more fidgety in social situations than when alone. ① ② ③ ④
2. Expressions on his or her face don't match what he or she is saying. ① ② ③ ④
3. Seems self-confident when interacting with others. ① ② ③ ④
4. When under stress, he or she shows rigid or inflexible patterns of behavior that seem odd. ① ② ③ ④
5. Doesn't recognize when others are trying to take advantage of him or her. ① ② ③ ④
6. Would rather be alone than with others. ① ② ③ ④
7. Is aware of what others are thinking or feeling. ① ② ③ ④
8. Behaves in ways that seem strange or bizarre. ① ② ③ ④
9. Clings to adults, seems too dependent on them. ① ② ③ ④
10. Takes things too literally and doesn't get the real meaning of a conversation. ① ② ③ ④
11. Has good self-confidence. ① ② ③ ④
12. Is able to communicate his or her feelings to others. ① ② ③ ④
13. Is awkward in turn-taking interactions with peers (for example, doesn't seem to understand the give-and-take of conversations). ① ② ③ ④
14. Is not well coordinated. ① ② ③ ④
15. Is able to understand the meaning of other people's tone of voice and facial expressions. ① ② ③ ④
16. Avoids eye contact or has unusual eye contact. ① ② ③ ④
17. Recognizes when something is unfair. ① ② ③ ④
18. Has difficulty making friends, even when trying his or her best. ① ② ③ ④
19. Gets frustrated trying to get ideas across in conversations. ① ② ③ ④
20. Shows unusual sensory interests (for example, mouthing or spinning objects) or strange ways of playing with toys. ① ② ③ ④
21. Is able to imitate others' actions. ① ② ③ ④
22. Plays appropriately with children his or her age. ① ② ③ ④
23. Does not join group activities unless told to do so. ① ② ③ ④
24. Has more difficulty than other children with changes in his or her routine. ① ② ③ ④
25. Doesn't seem to mind being out of step with or "not on the same wavelength" as others. ① ② ③ ④
26. Offers comfort to others when they are sad. ① ② ③ ④
27. Avoids starting social interactions with peers or adults. ① ② ③ ④
28. Thinks or talks about the same thing over and over. ① ② ③ ④
29. Is regarded by other children as odd or weird. ① ② ③ ④
30. Becomes upset in a situation with lots of things going on. ① ② ③ ④
31. Can't get his or her mind off something once he or she starts thinking about it. ① ② ③ ④
32. Has good personal hygiene. ① ② ③ ④

Continue on back page

Additional copies of this form (W-608A) may be purchased from WPS. Please contact us at **800.648.8857** or **www.wpspublish.com**.
Copyright © 2012 by Western Psychological Services. Not to be reproduced, adapted, and/or translated in whole or in part without prior written permission of WPS (rights@wpspublish.com). All rights reserved. Printed in USA. 2 3 4 5 6 7 8 9

PLEASE PRESS HARD WHEN MARKING YOUR RESPONSES.

1 = NOT TRUE   2 = SOMETIMES TRUE   3 = OFTEN TRUE   4 = ALMOST ALWAYS TRUE

33. Is socially awkward, even when he or she is trying to be polite. ① ② ③ ④
34. Avoids people who want to be emotionally close to him or her. ① ② ③ ④
35. Has trouble keeping up with the flow of a normal conversation. ① ② ③ ④
36. Has difficulty relating to adults. ① ② ③ ④
37. Has difficulty relating to peers. ① ② ③ ④
38. Responds appropriately to mood changes in others (for example, when a friend's or playmate's mood changes from happy to sad). ① ② ③ ④
39. Has an unusually narrow range of interests. ① ② ③ ④
40. Is imaginative, good at pretending (without losing touch with reality). ① ② ③ ④
41. Wanders aimlessly from one activity to another. ① ② ③ ④
42. Seems overly sensitive to sounds, textures, or smells. ① ② ③ ④
43. Separates easily from caregivers. ① ② ③ ④
44. Doesn't understand how events relate to one another (cause and effect) the way other children his or her age do. ① ② ③ ④
45. Focuses his or her attention to where others are looking or listening. ① ② ③ ④
46. Has overly serious facial expressions. ① ② ③ ④
47. Is too silly or laughs inappropriately. ① ② ③ ④
48. Has a sense of humor, understands jokes. ① ② ③ ④
49. Does extremely well at a few tasks, but does not do as well at most other tasks. ① ② ③ ④
50. Has repetitive, odd behaviors such as hand flapping or rocking. ① ② ③ ④
51. Has difficulty answering questions directly and ends up talking around the subject. ① ② ③ ④
52. Knows when he or she is talking too loud or making too much noise. ① ② ③ ④
53. Talks to people with an unusual tone of voice (for example, talks like a robot or like he or she is giving a lecture). ① ② ③ ④
54. Seems to react to people as if they are objects. ① ② ③ ④
55. Knows when he or she is too close to someone or is invading someone's space. ① ② ③ ④
56. Walks in between two people who are talking. ① ② ③ ④
57. Gets teased a lot. ① ② ③ ④
58. Concentrates too much on parts of things rather than seeing the whole picture. For example, if asked to describe what happened in a story, he or she may talk only about the kind of clothes the characters were wearing. ① ② ③ ④
59. Is overly suspicious. ① ② ③ ④
60. Is emotionally distant, doesn't show his or her feelings. ① ② ③ ④
61. Is inflexible, has a hard time changing his or her mind. ① ② ③ ④
62. Gives unusual or illogical reasons for doing things. ① ② ③ ④
63. Touches others in an unusual way (for example, he or she may touch someone just to make contact and then walk away without saying anything). ① ② ③ ④
64. Is too tense in social settings. ① ② ③ ④
65. Stares or gazes off into space. ① ② ③ ④

Stress Assessments

Stress is a necessary part of our lives and can have both beneficial and negative effects. The stress response is primarily determined by our perception of an event, transition, or problem. Finding a balance in our lives and managing our stress can be a challenge. An important first step is recognizing the degree to which we are affected by the stress in our lives and then move toward strategies to make it better.

The following are series of self-assessment scales to help us determine the degree and type of stress we are experiencing and how well our stress coping skills are working.

Disclaimer: Test scores on the following self-assessments do not reflect any particular diagnosis or course of treatment. They are meant as a tool to help assess your level of stress. Should you have any further concerns about your current well-being, you may contact NYSUT Social Services and talk confidentially to one of our social service specialists.

Symptoms of Stress

How frequently do you find yourself experiencing such problems as headaches, problems going to sleep or staying asleep, unexplained muscle pain, jaw pain, uncontrolled anger, and frustration? Using the table below, assess the frequency that you experience these common symptoms of stress.

	Frequency of symptoms						
**Symptoms**	Almost all day, every day	Once or twice daily	Every night or day	2-3 times per week	Once a week	Once a month	Never
Headaches							
Tense muscles, sore neck and back							
Fatigue							
Anxiety, worry, phobias							
Difficulty falling asleep							
Irritability							
Insomnia							
Bouts of anger/hostility							
Boredom, depression							
Eating too much or too little							
Diarrhea, cramps, gas, constipation							
Restlessness, itching, tics							

The more often you experience these symptoms of stress, the more likely stress is having a negative impact on your life. You may be so used to feeling a certain way that you assume this is normal. Look back over the Symptoms of Stress Table. Are there symptoms of stress that you would like to eliminate or change?

Perceived Stress Scale

A more precise measure of personal stress can be determined by using a variety of instruments that have been designed to help measure individual stress levels. The first of these is called the **Perceived Stress Scale.**

The Perceived Stress Scale (PSS) is a classic stress assessment instrument. This tool, while originally developed in 1983, remains a popular choice for helping us understand how different situations affect our feelings and our perceived stress. The questions in this scale ask about your feelings and thoughts during the last month. In each case, you will be asked to indicate how often you felt or thought a certain way. Although some of the questions are similar, there are differences between them and you should treat each one as a separate question. The best approach is to answer fairly quickly. That is, don't try to count up the number of times you felt a particular way; rather indicate the alternative that seems like a reasonable estimate.

For each question choose from the following alternatives:

0 – never 1 - almost never 2 – sometimes 3 - fairly often 4 - very often

____ 1. In the last month, how often have you been upset because of something that happened unexpectedly?

_____ 2. In the last month, how often have you felt that you were unable to control the important things in your life?

_____ 3. In the last month, how often have you felt nervous and stressed?

_____ 4. In the last month, how often have you felt confident about your ability to handle your personal problems?

_____ 5. In the last month, how often have you felt that things were going your way?

_____ 6. In the last month, how often have you found that you could not cope with all the things that you had to do?

_____ 7. In the last month, how often have you been able to control irritations in your life?

_____ 8. In the last month, how often have you felt that you were on top of things?

_____ 9. In the last month, how often have you been angered because of things that happened that were outside of your control?

_____ 10. In the last month, how often have you felt difficulties were piling up so high that you could not overcome them?

<u>Figuring your PSS score:</u>

You can determine your PSS score by following these directions:

First, reverse your scores for questions 4, 5, 7, & 8. On these 4 questions, change the scores like this: 0 = 4, 1 = 3, 2 = 2, 3 = 1, 4 = 0.

Now add up your scores for each item to get a total. **My total score is ______.**

Individual scores on the PSS can range from 0 to 40 with higher scores indicating higher perceived stress.

Scores ranging from 0-13 would be considered low stress.

Scores ranging from 14-26 would be considered moderate stress.

Scores ranging from 27-40 would be considered high perceived stress.

The Perceived Stress Scale is interesting and important because your perception of what is happening in your life is most important. Consider the idea that 2 individuals could have the exact same events and experiences in their lives for the past month. Depending on their perception, total score could put one of those individuals in the low stress category and the total score could put the second person in the high stress category.

The Ardell Wellness Stress Test

Don Ardell developed a stress assessment that is unique in its holistic approach to stress. In chapter one, you learned about the importance of incorporating all dimensions of health in your understanding of stress. The Ardell Wellness Stress Test incorporates physical, mental, emotional, spiritual, and social aspects of health for a balanced assessment.

Rate your satisfaction with each of the following items by using this scale:

+ 3 = Ecstatic | **-1 = Mildly disappointed**

+ 2 = Very happy | **- 2 = Very disappointed**

+ 1 = Mildly happy | **- 3 = Completely dismayed**

0 = Indifferent

_____ 1. Choice of career

_____ 2. Present job/ business/ school

_____ 3. Marital status

_____ 4. Primary relationships

_____ 5. Capacity to have fun

_____ 6. Amount of fun experienced in last month

_____ 7. Financial prospects

_____ 8. Current income level

_____ 9. Spirituality

_____ 10. Level of self-esteem

_____ 11. Prospects for having impact on those who know you and possibly others

_____ 12. Sex life

_____ 13. Body, how it looks and performs

_____ 14. Home life

_____ 15. Life skills and knowledge of issues and facts unrelated to your job or profession

_____ 16. Learned stress management capacities

_____ 17. Nutritional knowledge, attitudes, and choices

_____ 18. Ability to recover from disappointment, hurts, setbacks, and tragedies

_____ 19. Confidence that you currently are, or will in the future be, reasonably close to your highest potential.

_____ 20. Achievement of a rounded or balanced quality in your life

_____ 21. Sense that life for you is on an upward curve, getting better and fuller all the time

_____ 22. Level of participation in issues and concerns beyond your immediate interests

_____ 23. Choice whether to parent or not and with the consequences or results of that choice

_____ 24. Role in some kind of network of friends, relatives, and/or others about whom you care deeply and who reciprocate that commitment to you.

_____ 25. Emotional acceptance of the inescapable reality of aging

Total ______

Ardell Wellness Stress Test Interpretation

+ 51 to + 75 You are a self-actualized person, nearly immune from the ravages of stress. There are few, if any, challenges likely to untrack you from a sense of near total well-being.

+ 25 to + 50 You have mastered the wellness approach to life and have the capacity to deal creatively and efficiently with events and circumstances.

+ 1 to + 24 You are a wellness-oriented person, with an ability to prosper as a whole person, but you should give a bit more attention to optimal health concepts and skill building.

0 to - 24 You are a candidate for additional training in how to deal with stress. A sudden increase in potentially negative events and circumstances could cause a severe emotional setback.

- 25 to - 50 You are a candidate for counseling. You are either too pessimistic or have severe problems in dealing with stress.

- 51 to - 75 You are a candidate for major psychological care with virtually no capacity for coping with life's problems.

(Adapted from High Level Wellness: An Alternative to Doc, Drugs and Disease by Don Ardell)

Look back at the items in the Ardell Wellness Stress Test. Identify which items related more to physical health, to mental health, to emotional health, to spiritual health, and to social health. Do you see any patterns develop? For instance, are more areas of disappointment related to physical health than to social health? Remember, for holistic health we are seeking a balance in all dimensions of health.

Source: http://faculty.weber.edu/molpin/healthclasses/1110/bookchapters/selfassessmentchapter.htm

If you find yourself needing to talk to someone about how you are being affected by stress, you can call NYSUT Social Services, Monday through Friday, 9:00am to 5:00pm at 1-800-342-9810, ext. 6206, and speak to one of our social service specialists.

Stress Coping Resources Inventory:
A Self-Assessment

Instructions: People differ remarkably in their responses to potentially stressful events. For instance, about one in ten hostages comes out of captivity a mentally healthier person that when entering, while the others my face extreme emotional difficulty. What are the factors associated with coping success? The questions below relate to factors most closely associated with the capacity to cope successfully with stress. Circle the letter which lists the option that you choose. Answer each question as honestly as possible.

1. How frequently do you moderately exercise?
    a. Daily or more often
    b. Once or twice a week
    c. Once or twice a month
    d. Seldom

2. How often do you get a full, restful night of sleep?
    a. Most every night
    b. Four to five times a each week
    c. Two to three times each week
    d. Seldom

3. To what extent is your energy sufficient for our work and daily activities?
    a. to a very great extent
    b. to some extent
    c. to little extent
    d. to very little extent

4. How closely does your weight approach the ideal level?
    a. My weight is at the ideal level
    b. My weight is close to the idea level
    c. My weight is not close to the ideal level
    d. I am dangerously overweight (underweight)

5. To what extent do you eat a nutritious diet?
    a. to a very great extent
    b. to some extent
    c. to little extent
    d. to very little extent

6. Which of the following best describes your use of tobacco?
    a. In no period of my life have I had the habit of smoking or chewing tobacco.
    b. Early in my life for a short period I smoked or chewed tobacco
    c. I stopped smoking or chewing tobacco over the past two years
    d. I currently smoke or chew tobacco

7. Which of the following best describes your use of alcohol?
    a. I do not abuse alcohol, and never have. (Abuse is defined as drinking more than two drinks within a short period such as an evening.)
    b. Very occasionally I abuse alcohol.
    c. I have a history of abusing alcohol, but am not presently abusing it.
    d. I am presently abusing alcohol.

8. To what extent do you believe that you have a history of coping well with highly stressful situations?
    a. to a very great extent
    b. to a great extent
    c. to a little extent
    d. to a very little extent

9. How confident are you of being able to control your emotions in stressful situations?
    a. I never let my emotions run away me.
    b. I seldom let my emotions run away with me.
    c. I sometimes let my emotions run away with me.
    d. I often let my emotions run away with me.

10. When things are not going well, how likely are you to view the situation as being temporary rather than permanent?
    a. very likely
    b. likely
    c. unlikely
    d. very unlikely

11. When something bad happens to you, how likely are you to exaggerate its importance?
    a. very unlikely
    b. unlikely
    c. likely
    d. very likely

12. When stressed by a complex situation, how likely are you to focus your attention on those aspects of the situation that you can manage?
    a. very likely
    b. likely
    c. unlikely
    d. very unlikely

13. When highly stressed, how capable are you of changing your thinking to calm down?
    a. very capable
    b. capable
    c. incapable
    d. very incapable

14. When confronted with a stressful situation, how likely are you to wait passively for events to develop rather than to take charge?

a. very unlikely
b. unlikely
c. likely
d. very likely

15. Which of the following courses of action are you most likely to take when you have become thoroughly frustrated?

a. identify an alternate goal and pursue it
b. pursue a relaxing activity
c. withdraw and fell sorry for yourself
d. vent your aggression on someone weaker than you

16. If you had worn an article of clothing one day and then found it to be flawed, how likely would you be to return it and ask for a refund?

a. very likely
b. likely
c. unlikely
d. very unlikely

17. When an unexpected, negative event happens to you, how likely are you to actively seek information about the event and how to cope with it?

a. very likely
b. likely
c. unlikely
d. very unlikely

18. How much decision-making power so you have in your family?

a. more power than any other member of my family
b. as much power as any other member of my family
c. less power than most members of my family
d. less power than any other member of my family

19. How much decision-making power do you have in your working environment? (if not working outside the home at present, use your last job as a basis for answering this question.)

a. more power than most members of my work team
b. as much power as any other member of my work team
c. less power than most members of my work team
d. less power than any other member of my work team

20. To what extent do you believe that events in your life are merely the result of luck, fate, or chance?

a. to very little extent
b. to little extent
c. to some extent
d. to a great extent

21. What is your best guess as to the extent and quality of contact you had with your parent(s) shortly after birth?

a. was given an above average amount of contact by happy parent(s)
b. was given an average amount of contact by happy parent(s)
c. was given an average amount of contact by unhappy (perhaps angry) parent(s)
d. was given a below average amount of contact by unhappy (perhaps angry) parent(s)

22. During your early childhood, to what extent was your mother both calm and generally permissive?

a. to a very great extent
b. to some extent
c. to little extent
d. to very little extent

23. How easily do you make friends in a strange situation?

a. very easily
b. easily
c. uneasily
d. very uneasily

24. When highly stressed, how likely are you to ask friends or relatives for help?

a. very likely
b. likely
c. unlikely
d. very unlikely

25. In comparison with other people, how likely are you to see others as threatening, uncooperative, or exploitative?

a. highly unlikely
b. unlikely
c. likely
d. highly likely

26. How often are you confused about the intentions of others toward you?

a. very infrequently
b. infrequently
c. frequently
d. very frequently

27. To what extent are you aware of practical, healthy ways of relaxing?

a. to a very great extent
b. to some extent
c. to little extent
d. to very little extent

28. How frequently do you pursue some highly relaxing practice?

a. daily or more often
b. once or twice a week
c. once or twice a month
d. seldom

29. How often do you engage in a spiritual practice such as prayer, mediation, or inspirational reading to enrich your interior life?

a. daily or more often
b. once or twice a week
c. once or twice a month
d. seldom

30. How connected do you feel to your conception of a higher power or to a worthy cause?
    a. to a very great extent
    b. to some extent
    c. to little extent
    d. to very little extent

31. To what extent do you believe your life has purpose?
    a. to a very great extent
    b. to some extent
    c. to little extent
    d. to very little extent

32. How much contact do you have with what you would consider a spiritual community?
    a. very much
    b. much
    c. very little
    d. none

Scoring Legend
Please note that the scoring legend has been derived rationally, not empirically. Nevertheless, you might find it interesting to compute your score for each of the scales below using the following legend: 'a' = 4; 'b'= 3; 'c' = 2; 'd' = 1.

Wellness Scale (sum of scores for questions 1-7 divided by 7) _____

Thought Control Scale (sum of scores for questions 8-13, divided by 6) _____

Active Coping Scale (sum of scores for questions 14-20, divided by7) _____

Social Ease Scale (sum of scores for questions 21-26, divided by 6) _____

Tension reduction Scale (sum of scores for questions 27-28, divided by 2) _____

Spiritual Practice Scale (sum of scores for questions 29-32 divided by 4) _____

Overall Score (sum of the scale scores above, divided by 6) _____

Interpreting Your Score. A perfect score on each scale would be 4. With this in mind, we might construct the following interpretive key:

An overall score of 3.5+ suggests you may be a superior stresscoper.

An overall score of 2.5-3.4 suggests you may be an above average stresscoper.

An overall score of 1.5-2.4 suggests you may be an average stresscoper.

An overall score of less than 1.5 suggests you may be a below average stresscoper.

Source: *'Write Your Own Prescription for Stress'*
Kenneth B. Matheny, Ph.D., ABPP and Christopher J. McCarthy, Ph.D. ~ 2000

SOCIAL RESPONSIVENESS SCALE AUTOSCORE™ FORM John N. Constantino, M.D. **PARENT REPORT**

DIRECTIONS

For each question, circle the number that best describes the child's behavior *over the past 6 months.*

ID
[redacted]: ______________________ Chronological Age: ____________

Gender (required): ☐ Female ☐ Male [redacted]: ______________________

[redacted]: ______________________ Administration Date: ____________

Relationship to Child: ☒ Mother ☐ Father ☐ Other ______________________

PLEASE PRESS HARD WHEN MARKING YOUR RESPONSES.

1 = NOT TRUE 2 = SOMETIMES TRUE 3 = OFTEN TRUE 4 = ALMOST ALWAYS TRUE

**1.** Seems much more fidgety in social situations than when alone.	1	2	3	4
**2.** Expressions on his or her face don't match what he or she is saying.	1	2	3	4
**3.** Seems self-confident when interacting with others.	1	2	3	4
**4.** When under stress, he or she shows rigid or inflexible patterns of behavior that seem odd.	1	2	3	4
**5.** Doesn't recognize when others are trying to take advantage of him or her.	1	2	3	4
**6.** Would rather be alone than with others.	1	2	3	4
**7.** Is aware of what others are thinking or feeling.	1	2	3	4
**8.** Behaves in ways that seem strange or bizarre.	1	2	3	4
**9.** Clings to adults, seems too dependent on them.	1	2	3	4
**10.** Takes things too literally and doesn't get the real meaning of a conversation.	1	2	3	4
**11.** Has good self-confidence.	1	2	3	4
**12.** Is able to communicate his or her feelings to others.	1	2	3	4
**13.** Is awkward in turn-taking interactions with peers (e.g., doesn't seem to understand the give-and-take of conversations).	1	2	3	4
**14.** Is not well coordinated.	1	2	3	4
**15.** Is able to understand the meaning of other people's tone of voice and facial expressions.	1	2	3	4
**16.** Avoids eye contact or has unusual eye contact.	1	2	3	4
**17.** Recognizes when something is unfair.	1	2	3	4
**18.** Has difficulty making friends, even when trying his or her best.	1	2	3	4
**19.** Gets frustrated trying to get ideas across in conversations.	1	2	3	4
**20.** Shows unusual sensory interests (e.g., mouthing or spinning objects) or strange ways of playing with toys.	1	2	3	4
**21.** Is able to imitate others' actions.	1	2	3	4
**22.** Plays appropriately with children his or her age.	1	2	3	4
**23.** Does not join group activities unless told to do so.	1	2	3	4
**24.** Has more difficulty than other children with changes in his or her routine.	1	2	3	4
**25.** Doesn't seem to mind being out of step with or "not on the same wavelength" as others.	1	2	3	4
**26.** Offers comfort to others when they are sad.	1	2	3	4
**27.** Avoids starting social interactions with peers or adults.	1	2	3	4
**28.** Thinks or talks about the same thing over and over.	1	2	3	4
**29.** Is regarded by other children as odd or weird.	1	2	3	4
**30.** Becomes upset in a situation with lots of things going on.	1	2	3	4
**31.** Can't get his or her mind off something once he or she starts thinking about it.	1	2	3	4
**32.** Has good personal hygiene.	1	2	3	4

Continue on back page...

Published by
WESTERN PSYCHOLOGICAL SERVICES
wps® 12031 Wilshire Boulevard
Los Angeles, CA 90025-1251
Publishers and Distributors

Copyright © 2005 by WESTERN PSYCHOLOGICAL SERVICES
Not to be reproduced in whole or in part without written permission of Western Psychological Services.
All rights reserved.
2 3 4 5 6 7 8 9 Printed in U.S.A.
W-399AP

PLEASE PRESS HARD WHEN MARKING YOUR RESPONSES.

1 = NOT TRUE 2 = SOMETIMES TRUE 3 = OFTEN TRUE 4 = ALMOST ALWAYS TRUE

**33.** Is socially awkward, even when he or she is trying to be polite.	1	2	3	4
**34.** Avoids people who want to be emotionally close to him or her.	1	2	3	4
**35.** Has trouble keeping up with the flow of a normal conversation.	1	2	3	4
**36.** Has difficulty relating to adults.	1	2	3	4
**37.** Has difficulty relating to peers.	1	2	3	4
**38.** Responds appropriately to mood changes in others (e.g., when a friend's or playmate's mood changes from happy to sad).	1	2	3	4
**39.** Has an unusually narrow range of interests.	1	2	3	4
**40.** Is imaginative, good at pretending (without losing touch with reality).	1	2	3	4
**41.** Wanders aimlessly from one activity to another.	1	2	3	4
**42.** Seems overly sensitive to sounds, textures, or smells.	1	2	3	4
**43.** Separates easily from caregivers.	1	2	3	4
**44.** Doesn't understand how events relate to one another (cause and effect) the way other children his or her age do.	1	2	3	4
**45.** Focuses his or her attention to where others are looking or listening.	1	2	3	4
**46.** Has overly serious facial expressions.	1	2	3	4
**47.** Is too silly or laughs inappropriately.	1	2	3	4
**48.** Has a sense of humor, understands jokes.	1	2	3	4
**49.** Does extremely well at a few tasks, but does not do as well at most other tasks.	1	2	3	4
**50.** Has repetitive, odd behaviors such as hand flapping or rocking.	1	2	3	4
**51.** Has difficulty answering questions directly and ends up talking around the subject.	1	2	3	4
**52.** Knows when he or she is talking too loud or making too much noise.	1	2	3	4
**53.** Talks to people with an unusual tone of voice (e.g., talks like a robot or like he or she is giving a lecture).	1	2	3	4
**54.** Seems to react to people as if they are objects.	1	2	3	4
**55.** Knows when he or she is too close to someone or is invading someone's space.	1	2	3	4
**56.** Walks in between two people who are talking.	1	2	3	4
**57.** Gets teased a lot.	1	2	3	4
**58.** Concentrates too much on parts of things rather than seeing the whole picture. For example, if asked to describe what happened in a story, he or she may talk only about the kind of clothes the characters were wearing.	1	2	3	4
**59.** Is overly suspicious.	1	2	3	4
**60.** Is emotionally distant, doesn't show his or her feelings.	1	2	3	4
**61.** Is inflexible, has a hard time changing his or her mind.	1	2	3	4
**62.** Gives unusual or illogical reasons for doing things.	1	2	3	4
**63.** Touches others in an unusual way (e.g., he or she may touch someone just to make contact and then walk away without saying anything).	1	2	3	4
**64.** Is too tense in social settings.	1	2	3	4
**65.** Stares or gazes off into space.	1	2	3	4
